# Longitudinal Molecular Epidemiology and Genomic Evolution of Porcine Reproductive and Respiratory Syndrome Virus in a Large‐Scale Swine Production System in Northern China, 2023–2026

**DOI:** 10.1155/tbed/6131183

**Published:** 2026-09-22

**Authors:** Tao Zhang, Shaofu Song, Hechao Zhu, Shixuan Zhu, Yanjiao Wang, Anding Zhang, Kui Hou, Rui Liu, Xujiao Ren, Junlong Zhao

**Affiliations:** ^1^ National Key Laboratory of Agricultural Microbiology, College of Veterinary Medicine, Huazhong Agricultural University, Wuhan 430070, Hubei, China, hzau.edu.cn; ^2^ COFCO Joycome (Chifeng) Co., Ltd., Chifeng 024000, Inner Mongolia, China; ^3^ COFCO Joycome Foods Limited, Beijing 100020, China; ^4^ CAS Key Laboratory of Pathogen Microbiology and Immunology, Institute of Microbiology, Chinese Academy of Sciences (CAS), Beijing 100101, China, cas.cn

**Keywords:** molecular epidemiology, NADC30-like PRRSV, porcine reproductive and respiratory syndrome virus, recombination, whole-genome sequencing

## Abstract

Porcine reproductive and respiratory syndrome virus (PRRSV) remains a major threat to swine production because of its persistent circulation, genetic diversity, and frequent recombination. However, longitudinal molecular surveillance within large‐scale integrated production systems remains limited. In this study, we investigated the epidemiological and genomic characteristics of PRRSV in a large‐scale swine production system in Chifeng, Inner Mongolia, northern China, from January 2023 to May 2026. A total of 380,050 PRRSV nucleic acid testing records were analyzed, and 36,474 were positive, yielding an overall positivity rate of 9.60%. PRRSV detection showed marked temporal fluctuations, with increased positivity from late 2025 to early 2026, and substantial heterogeneity among farms. Nursery pigs showed the highest positivity rate among production stages, followed by finishing pigs and gilts, whereas boars had the lowest positivity. Among sample types, castration/tail‐docking fluid, tongue‐tip fluid, and tissue/organ samples had relatively high positivity rates. ORF5‐based phylogenetic analysis revealed that all the study‐derived sequences belonged to PRRSV‐2, with sublineage 1.8 accounting for 53.74% and sublineage 1.5 accounting for 34.69%, indicating the predominance of lineage 1 strains. Whole‐genome phylogenetic analysis of the 11 successfully assembled PRRSV genomes confirmed that they were NADC30‐like PRRSVs and carried the characteristic 111 + 1 + 19 discontinuous deletion pattern in Nsp2. GP5 amino acid alignment revealed substitutions in antigenically relevant regions, including epitope A, epitope B, and predicted glycosylation‐related sites. Recombination analysis revealed potential recombination events involving lineage 8‐related fragments in CF‐k141‐31, CF‐k141‐560, and CF‐k141‐2190. These findings demonstrate that PRRSV circulation in this production system is characterized by temporal clustering, farm‐level heterogeneity, lineage 1 predominance, and genomic diversification of NADC30‐like strains. These results support the integration of routine real‐time reverse transcription quantitative PCR (RT‐qPCR) surveillance, ORF5 sequencing, and periodic whole‐genome sequencing for targeted PRRSV monitoring and control in large‐scale swine production systems.

## 1. Introduction

Porcine reproductive and respiratory syndrome (PRRS) remains one of the most economically important viral diseases affecting the global swine industry. This disease is characterized mainly by reproductive failure in sows, including abortion, stillbirth, mummified fetuses, and weak‐born piglets, as well as respiratory disease, poor growth performance, and increased mortality in nursery and growing pigs [1–3]. Since its first recognition in North America and Europe in the late 1980s and early 1990s, PRRS has become endemic in most major pig‐producing regions and continues to impose a substantial burden on commercial swine production. In China, PRRS virus (PRRSV) has circulated for more than two decades and remains difficult to control because of its extensive genetic diversity, frequent immune escape, variable pathogenicity, and incomplete cross‐protection provided by existing vaccines [4, 5].

PRRSV is an enveloped, positive‐sense, single‐stranded RNA virus belonging to the family Arteriviridae, with a genome of ~15 kb [6, 7]. ORF5 and Nsp2 are among the most variable genomic regions and are widely used for phylogenetic classification and molecular characterization [8, 9]. PRRSV‐2 predominates in Chinese swine herds, where lineages 1, 3, 5, and 8 have circulated successively or concurrently [4, 10]. In recent years, lineage 1 strains, particularly NADC30‐like and NADC34‐like PRRSVs, have become increasingly prevalent, further increasing the genetic complexity of circulating viruses [11, 12]. Frequent mutation and recombination among field, vaccine‐related, and heterologous lineage strains have generated mosaic genomes, and ORF5‐based classification alone may therefore be insufficient to define the complete genomic background of circulating PRRSVs [13, 14].

Although the prevalence and genetic diversity of PRRSV have been extensively investigated in China, most studies have relied on provincial or regional clinical submissions, outbreak‐associated samples, or relatively short surveillance periods [15–18]. Longitudinal molecular surveillance within large‐scale integrated swine production systems remains limited, particularly at the farm, production‐stage and sample‐type levels. Integrating routine nucleic acid detection with ORF5 sequencing and whole‐genome analysis may therefore provide a more comprehensive understanding of PRRSV circulation and evolution within interconnected production networks.

In this study, we conducted a longitudinal molecular epidemiological investigation of PRRSV in a large‐scale swine production system in northern China from 2023 to 2026. By integrating large‐scale nucleic acid detection, ORF5 sequence analysis, and whole‐genome sequencing, we characterized the spatiotemporal distribution, production‐stage and sample‐type patterns, lineage composition, and genomic evolution of circulating PRRSV strains. This study provides molecular evidence for optimizing targeted surveillance and control strategies in large‐scale swine production systems.

## 2. Materials and Methods

### 2.1. Study Design and Sample Collection

A longitudinal molecular epidemiological study was conducted in a large‐scale integrated swine production system located in Chifeng, Inner Mongolia, northern China, from January 2023 to May 2026. Detailed characteristics of individual production units are provided in Table S1. The surveillance dataset included routine diagnostic records from multiple farms, production stages, and sample types within the production system. The surveillance dataset was generated through routine herd health monitoring and diagnostic activities rather than through a predefined research sampling scheme with a fixed number of samples. Consequently, surveillance coverage and sampling intensity varied over time according to the PRRSV monitoring and control strategy implemented within the production system. In 2023, PRRSV surveillance was conducted at a relatively low routine frequency. During 2024 and 2025, surveillance coverage and testing frequency were progressively increased as the company strengthened its PRRSV monitoring and control program. In 2026, a more intensive PRRSV stabilization/elimination program was implemented, under which routine PRRSV testing was increased from predominantly monthly surveillance to weekly and/or production‐batch‐based testing. The production stages included gestation/mating sows, farrowing/lactation units, gilts, nursery pigs, finishing pigs, and boars. The sample types included throat swabs, oral fluid, serum, tail‐root blood/swabs, placenta or abortion‐related samples, semen, tissue/organ samples, castration/tail‐docking fluid, oronasal swabs, umbilical cord blood, tongue‐tip fluid, and other clinically relevant samples.

For each sample, information on the sampling date, farm, production stage, sample type, and PRRSV detection result was recorded. Samples with incomplete metadata or invalid detection results were excluded from the epidemiological analysis. All samples were collected as part of routine herd health monitoring and diagnostic surveillance. The samples were transported to the laboratory under cold‐chain conditions and processed immediately or stored at −80°C until further use.

### 2.2. Sample Processing and Viral RNA Extraction

Tissue and organ samples were homogenized in sterile phosphate‐buffered saline (PBS) or Dulbecco’s modified Eagle’s medium (DMEM; Gibco) and clarified by centrifugation. Swab samples were vortexed thoroughly in sterile PBS, and liquid samples, including serum, oral fluid, semen, and processing fluid, were processed according to the sample type. Viral RNA was extracted from processed samples using TRIzol reagent (Invitrogen, USA) according to the manufacturer’s instructions. The extracted RNA was either used immediately for downstream detection and amplification or stored at −80°C.

### 2.3. Real‐Time Reverse Transcription Quantitative PCR (RT‐qPCR) Detection of PRRSV

PRRSV nucleic acid detection was performed using an RT‐qPCR assay with the PRRSV (Universal) Real‐time RT‐qPCR Detection Kit (Beijing ComWin Biotech Co., Ltd., Beijing, China) according to the manufacturer’s instructions. The PRRSV‐specific primer and probe sequences used for RT‐qPCR detection are provided in Table S2. Amplification and fluorescence detection were performed using a ViiA 7 Real‐Time PCR System (Applied Biosystems, Grand Island, NY, USA). Samples were classified as PRRSV‐positive or PRRSV‐negative according to the cycle threshold (Ct) criteria specified by the manufacturer.

### 2.4. ORF5 Amplification and Sequencing

PRRSV‐positive samples with Ct values <30 and sufficient viral RNA concentrations for successful amplification were selected for ORF5 amplification. Reverse transcription was performed using extracted viral RNA as the template. The complete ORF5 gene was amplified by RT‐PCR using PRRSV‐specific primers (Table S2). The thermocycler conditions used for PCR were 5 min at 98°C, followed by 35 cycles of 10 s at 98°C, 5 s at 60°C, and 30 s at 72°C, and a final extension of 10 min at 72°C. PCR products were examined by agarose gel electrophoresis. Amplicons of the expected size were purified using a Universal DNA Purification Kit (Tiangen Biotech [Beijing] Co., Ltd.) and subjected to Sanger sequencing (Beijing Tsingke Biotech Co., Ltd.).

After quality control and harmonization of production unit identifiers, the final phylogenetic dataset comprised 441 study‐derived ORF5 sequences originating from 29 production units. Sequence metadata, including sampling date and production unit origin, were retained for temporal and production unit‐level analyses. The distribution of ORF5 sequences across production units and years is summarized in Table S3. The nucleotide sequences of all 441 study‐derived ORF5 genes included in the final phylogenetic and sequence analyses are provided in Data S1.

### 2.5. Whole‐Genome Sequencing by High‐Throughput Sequencing

A total of 21 PRRSV‐positive samples were subjected to whole‐genome sequencing. The samples used for downstream whole‐genome analyses were not preselected according to production unit origin, sampling time, or ORF5 phylogenetic classification. Of the 21 sequenced samples, 11 yielded complete or near‐complete PRRSV genome assemblies of sufficient quality for subsequent phylogenetic and genomic analyses. The remaining samples did not yield genome assemblies suitable for downstream whole‐genome analysis and were therefore excluded from the genomic dataset. Thus, inclusion of the 11 study‐derived genomes was determined by successful genome recovery rather than by an a priori lineage‐ or farm‐based selection scheme. Detailed metadata for these 11 genomes, including production unit origin, sampling date, production stage, sample type, and sampling reason, are provided in Table S4.

The sequencing libraries were prepared using the NEBNext Ultra II RNA Library Prep Kit for Illumina (New England Biolabs, Ipswich, MA, USA) according to the manufacturer’s instructions. After library quality control, the qualified libraries were pooled and subjected to paired‐end sequencing on an Illumina NovaSeq 6000 platform (Illumina, San Diego, CA, USA). The raw sequencing reads were quality‐filtered to remove adapters, low‐quality reads, and reads containing excessively ambiguous bases. Host‐derived reads were removed by mapping against the *Sus scrofa* reference genome. PRRSV‐related reads were assembled using de novo and/or reference‐guided assembly strategies. Candidate viral contigs were identified by BLAST comparison against the NCBI nucleotide database. Consensus sequences were generated after manual inspection of the assembly quality and ambiguous regions. The 11 successfully assembled complete or near‐complete genomes were used for phylogenetic, homology, amino acid variation, recombination, and within‐production‐unit longitudinal analyses.

### 2.6. Phylogenetic Analysis of PRRSV

Representative PRRSV reference sequences were retrieved from GenBank (Table 1). The reference dataset included PRRSV‐1 reference strains and representative PRRSV‐2 strains from major lineages circulating in China and worldwide, including NADC30‐like, NADC34‐like, QYYZ‐like, VR2332‐like, CH‐1a‐like, HP‐PRRSV‐like, and vaccine‐related strains. The ORF5 nucleotide sequences and complete genome sequences were aligned separately using ClustalW. Phylogenetic trees were constructed using the neighbor‐joining method with 1000 bootstrap replicates in MEGA X [19–22]. The phylogenetic trees were visualized and annotated using the iTOL online tool (https://itol.embl.de/).

**Table 1:** Information on the PRRSV reference strains used in this study.

Classification	Lineage	Strain	Area	Time	Accession no.
PRRSV‐1	—	Lelystad virus	Netherlands	1993	M96262.2
		EuroPRRSV	America	2003	AY366525.1
		LNEU12	Liaoning, China	2014	KM196101.1
		PRRSV1/CN/FJFQ‐1/2023	Fujian, China	2023	OR260421.1
		NMEU09‐1	Beijing, China	2009	GU047345.1
PRRSV‐2	Sublineage 1.5	NADC34	America	2014	MF326985.1
		CH2018NCV‐Anheal‐1	China	2018	MH370474.1
	Sublineage 1.8	NADC30	America	2008	MH500776.1
		SDQZ‐1609	Shandong, China	2016	MH651746.1
		LNCH‐1604	Liaoning, China	2016	MH651741.1
		JL580	Jilin, China	2013	KR706343.1
	Lineage 3	QYYZ	Guangdong, China	2011	JQ308798.1
		GM2	Guangdong, China	2011	JN662424.1
	Lineage 5	VR‐2332	America	2003	AY150564.1
		RespPRRS MLV	America	2005	AF066183.4
	Lineage 8	CH‐1a	Beijing, China	1996	AY032626.1
		CH‐1R	Heilongjiang, China	2008	EU807840.1
		XJ17‐5	Xinjiang, China	2017	MK759853.1
		TJbd14‐1	Tianjin, China	2014	KP742986.1
		JXA1	Jiangxi, China	2006	JX317649.1
		JXA1‐R	Jiangsu, China	2020	MT163314.1
		HuN	Hunan, China	2007	EF517962.1
		GD	Beijing, China	2008	EU825724.1
		TJ	Tianjin, China	2006	EU860248.1
		NT1	Shanghai, China	2014	KP179402.1

### 2.7. Homology Analysis and Sequence Alignment

Whole‐genome nucleotide identities were calculated among the 11 complete or near‐complete PRRSV genomes obtained in this study using MegAlign software. In addition, each study‐derived genome was compared with representative PRRSV reference strains from different lineages to evaluate its overall genomic relatedness. Pairwise whole‐genome nucleotide identities among the 11 study‐derived strains and between the study‐derived and reference strains were summarized in comprehensive identity matrices. The nucleotide and deduced amino acid identities of ORF1a, ORF1b, and ORF2–ORF7 between CF‐k141‐31 and representative PRRSV reference strains were calculated and visualized as heatmaps.

The GP5 sequences of the study‐derived and reference strains were aligned to identify amino acid substitutions in antigenically important regions, with emphasis on the decoy epitope A, neutralizing epitope B, and predicted N‐linked glycosylation sites. Nsp2 amino acid sequences were aligned with representative PRRSV strains from different lineages to identify lineage‐associated deletion patterns. Amino acid positions were numbered relative to VR‐2332 reference strain. The deletion patterns of the study‐derived strains were compared with those of the HP‐PRRSV‐like, NADC30‐like, and NADC34‐like strains to aid in the molecular characterization.

### 2.8. Recombination Analysis

Potential recombination events in the complete genome sequences were screened using RDP4 software [23]. Multiple algorithms implemented in RDP4, including RDP, GENECONV, BootScan, MaxChi, Chimaera, SiScan, and 3Seq, were used to detect recombination signals. Putative recombination events were considered reliable when supported by multiple methods with statistical significance. Recombination signals were further evaluated using SimPlot version 3.5 [24]. Similarity plot and bootscanning analyses were performed with a sliding window of 200 bp and a step size of 20 bp under the Kimura two‐parameter model, with gap‐stripping enabled. Putative major and minor parental strains were inferred on the basis of similarity profiles and phylogenetic relationships. To validate the predicted recombinant fragments, phylogenetic trees were constructed using the corresponding recombinant regions and compared with the complete‐genome phylogeny.

## 3. Results

### 3.1. Spatiotemporal and Production‐Stage Distribution of PRRSV Detection

**Figure 1:**
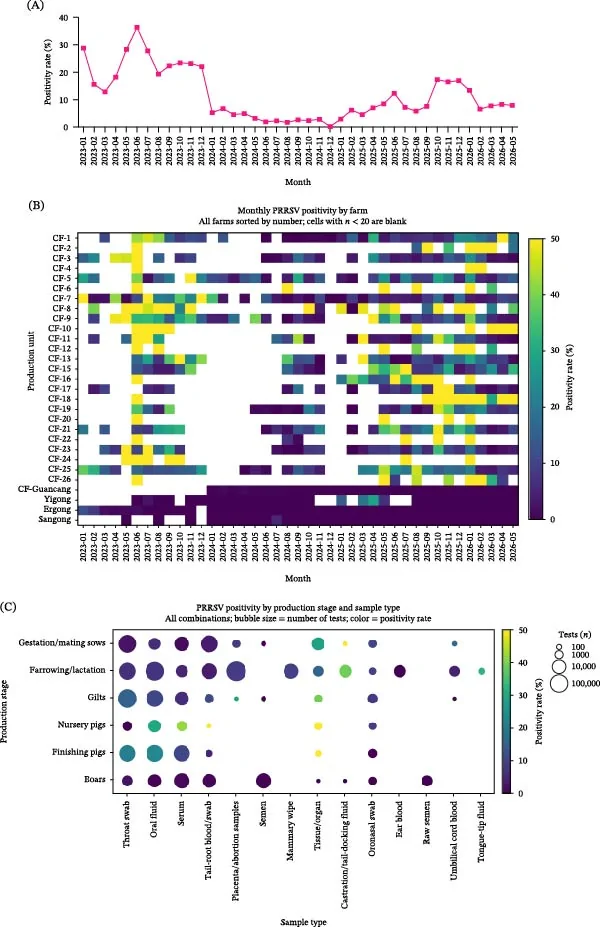
Spatiotemporal distribution and production‐stage/sample‐type patterns of PRRSV detection in a large‐scale swine production system from 2023 to 2026. (A) Monthly PRRSV positivity rate from January 2023 to May 2026 based on routine RT‐qPCR surveillance. (B) Temporal heterogeneity of PRRSV positivity across all farms. Farm‐by‐month heatmap showing PRRSV positivity rates across different production units. Each cell represents the positivity rate for a given farm and month; farm‐month combinations with fewer than 20 tests were treated as insufficiently sampled and left blank. (C) Complete distribution of PRRSV positivity across production stages and sample types. Bubble plot showing PRRSV positivity according to production stage and sample type. Bubble color represents the PRRSV positivity rate, and bubble size represents the number of tested samples.

From January 2023 to May 2026, 380,050 PRRSV nucleic acid testing records were analyzed, of which 36,474 were positive, yielding an overall positivity rate of 9.60%. The annual testing volume and positivity rate varied markedly during the study period, with 24.59% (2290/9311) in 2023, 2.30% (1115/48,463) in 2024, 11.79% (18,281/155,026) in 2025, and 8.84% (14,788/167,250) during January–May 2026 (Table 2). The progressive increase in testing volume reflected the strengthening of the routine PRRSV surveillance program during the study period. In particular, the substantial increase during January–May 2026 coincided with the implementation of an intensified PRRSV stabilization/elimination program, under which routine testing was increased from predominantly monthly surveillance to weekly and/or production‐batch‐based testing. Monthly analysis revealed that PRRSV detection remained relatively low during most of 2024 but increased from late 2025 to early 2026, with positivity rates of 17.25% (3066/17,779), 16.47% (4285/26,019), 16.89% (4935/29,214), and 13.39% (4145/30,958) in October 2025, November 2025, December 2025, and January 2026, respectively (Figure 1A). These results indicated that PRRSV circulated continuously within the production system and showed clear temporal clustering during the surveillance period.

**Table 2:** PRRSV RT‐qPCR testing results during routine surveillance from January 2023 to May 2026.

Period	Number of positive tests	Number of tests	Positivity rate (%)
January–December 2023	2290	9311	24.59
January–December 2024	1115	48,463	2.30
January–December 2025	18,281	155,026	11.79
January–May 2026	14,788	167,250	8.84
Total	36,474	380,050	9.60

Farm‐level analysis revealed substantial heterogeneity in PRRSV positivity among different production units (Table S5). The heatmap of the monthly PRRSV positivity revealed that PRRSV activity was not evenly distributed across farms. Several farms showed persistent or recurrent PRRSV detection during specific time windows, whereas others remained at relatively low positivity levels throughout most of the observation period (Figure 1B). Cumulative positivity rates ranged from 0.10% in CF‐Guancang to 90.55% in CF‐20. Several units, including CF‐20 (90.55%, 230/254), CF‐10 (84.75%, 706/833), CF‐4 (81.99%, 305/372), and CF‐12 (79.92%, 386/483), showed high cumulative test positivity; however, these units generally had substantially smaller testing denominators than heavily monitored units. In contrast, CF‐8 contributed the largest number of positive detections (5441/13,671, 39.80%), while CF‐7 and CF‐25 also contributed large numbers of positive tests. Because surveillance intensity, production function, sample composition, and testing frequency differed among production units, these results represent production‐unit‐level test positivity rather than estimates of infection prevalence.

PRRSV positivity also varied substantially among production stages. Nursery pigs had the highest positivity rate, followed by finishing pigs and gilts. In contrast, compared with the other production stages, the positive rate of boars was much lower. Specifically, the positivity rates were 28.46% (2978/10,463) in nursery pigs, 18.52% (9916/53,533) in finishing pigs, 12.66% (8112/64,068) in gilts, 8.60% (11,258/130,841) in farrowing/lactation units, 5.41% (3862/71,362) in gestation/mating sows, and 0.70% (348/49,783) in boars (Table 3). These results suggested that nursery and finishing pigs represented major stages for PRRSV amplification and detection within this production system.

**Table 3:** PRRSV positivity by production stage.

Production stage	Number of positive tests	Number of tests	Positivity rate (%)
Nursery pigs	2978	10,463	28.46
Finishing pigs	9916	53,533	18.52
Gilts	8112	64,068	12.66
Farrowing/lactation	11,258	130,841	8.60
Gestation/mating sows	3862	71,362	5.41
Boars	348	49,783	0.70

Distinct sample types also showed substantial differences in PRRSV positivity. The positivity rates were 39.14% (2287/5843) for castration/tail‐docking fluid, 32.49% (116/357) for tongue‐tip fluid, 30.16% (3058/10,138) for tissue/organ samples, 12.46% (10,260/82,356) for oral fluid, 10.47% (10,803/103,222) for throat swabs, 9.94% (1107/11,140) for mammary wipes, 8.61% (3142/36,510) for placenta/abortion samples, 6.54% (3610/55,215) for serum, 5.96% (325/5450) for oronasal swabs, 4.74% (116/2449) for umbilical cord blood, 3.48% (1575/45,229) for tail‐root blood/swabs, 0.52% (74/14,118) for semen, and 0.02% (1/4946) for ear blood, whereas no positive samples were detected in raw semen (0/3077) (Table 4). The bubble plot further demonstrated that high PRRSV positivity was associated with specific combinations of production stage and sample type, supporting the value of targeted surveillance in high‐risk populations and samples (Figure 1C).

**Table 4:** PRRSV positivity by sample type.

Sample type	Number of positive tests	Number of tests	Positivity rate (%)
Castration/tail‐docking fluid	2287	5843	39.14
Tongue‐tip fluid	116	357	32.49
Tissue/organ	3058	10,138	30.16
Oral fluid	10,260	82,356	12.46
Throat swab	10,803	103,222	10.47
Mammary wipe	1107	11,140	9.94
Placenta/abortion samples	3142	36,510	8.61
Serum	3610	55,215	6.54
Oronasal swab	325	5450	5.96
Umbilical cord blood	116	2449	4.74
Tail‐root blood/swab	1575	45,229	3.48
Semen	74	14,118	0.52
Ear blood	1	4946	0.02
Raw semen	0	3077	0

### 3.2. ORF5‐Based Phylogenetic Classification of Circulating PRRSV Strains

**Figure 2:**
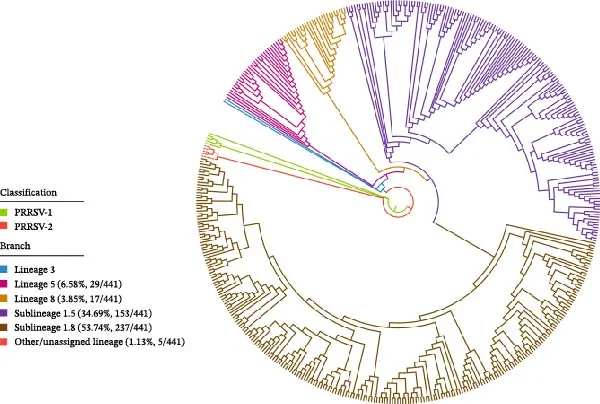
ORF5‐based phylogenetic analysis of the PRRSV strains detected in this study. A phylogenetic tree was constructed on the basis of the ORF5 nucleotide sequences obtained in this study and representative PRRSV reference strains. Branches are colored according to phylogenetic classification. All study‐derived sequences belonged to PRRSV‐2; the PRRSV‐1 cluster consisted only of reference strains. Among the 441 study‐derived sequences, 237 were assigned to sublineage 1.8, 153 to sublineage 1.5, 29 to lineage 5, and 17 to lineage 8, whereas five sequences formed a separate branch that could not be assigned to these established lineages/sublineages and were designated as other lineage.

To determine the genetic composition of circulating PRRSV strains, an ORF5‐based phylogenetic tree was constructed using the obtained ORF5 sequences and representative reference strains. All sequences obtained in this study belonged to PRRSV‐2; the PRRSV‐1 cluster shown in the tree consisted only of reference strains. Among the 441 ORF5 sequences analyzed, sublineage 1.8 was predominant, accounting for 53.74% (237/441), followed by sublineage 1.5 at 34.69% (153/441). Lineage 5 and lineage 8 accounted for 6.58% (29/441) and 3.85% (17/441), respectively. The remaining five sequences (1.13%, 5/441) formed a separate phylogenetic branch and were designated as an other/unassigned lineage (Figure 2).

The 441 ORF5 sequences originated from 29 production units and showed an uneven temporal distribution, with 59 sequences (13.38%) obtained in 2023, 20 (4.54%) in 2024, 238 (53.97%) in 2025, and 124 (28.12%) in 2026 (Table S3). The annual composition of the major ORF5‐defined lineages/sublineages also varied during the surveillance period (Figure S1A). Repeated sequencing from individual production units enabled a longitudinal assessment of circulating PRRSV populations. Twenty‐eight of the 29 production units were represented by sequences collected at multiple sampling time points, 26 were represented in at least two different years, and 21 were represented in at least 3 years. Notably, CF‐1, CF‐6, CF‐7, CF‐9, CF‐12, CF‐15, CF‐19, CF‐23, and CF‐25 were represented throughout all four study years. Production unit‐level analysis further revealed substantial heterogeneity in the lineage composition across the integrated production system (Figure S1B). Contemporaneous detection of genetically distinct PRRSV populations was identified on multiple sampling occasions within individual production units (Table S6). For example, sublineage 1.5 and lineage 5 were detected in CF‐2 on June 20, 2023, whereas sublineage 1.8 and lineage 8 were detected in CF‐4 on the same date. Sublineage 1.5 and lineage 8 were also detected in CF‐15 on June 20, 2023, and sublineage 1.5 and lineage 5 were detected in CF‐26 on June 22, 2023. These observations provide evidence of sustained production‐unit‐level PRRSV genetic heterogeneity and cocirculation during longitudinal surveillance.

To further characterize the genetic relationships of lineage 5 and lineage 8 viruses with vaccine‐related strains, ORF5 nucleotide identities were calculated against representative vaccine‐related reference strains (Table S7). The 29 lineage 5 sequences shared 97.4%–99.7% nucleotide identity with VR‐2332 and 97.4%–100.0% with RespPRRS MLV. The 17 lineage 8 sequences showed 92.7%–94.4% identity with CH‐1R but substantially higher identity with JXA1‐R, ranging from 97.5% to 100.0%. Pairwise ORF5 comparison within the major phylogenetic groups further revealed different degrees of intralineage diversity (Table S8). Nucleotide identities ranged from 83.3% to 100.0% within sublineage 1.8, 89.1%–100.0% within sublineage 1.5, 96.4%–100.0% within lineage 5, and 97.5%–100.0% within lineage 8. These results indicated relatively high sequence conservation within lineage 5 and lineage 8, whereas greater ORF5 diversity was observed among lineage 1 viruses.

### 3.3. Whole‐Genome Phylogenetic Analysis and Within‐Unit Genomic Variation

**Figure 3:**
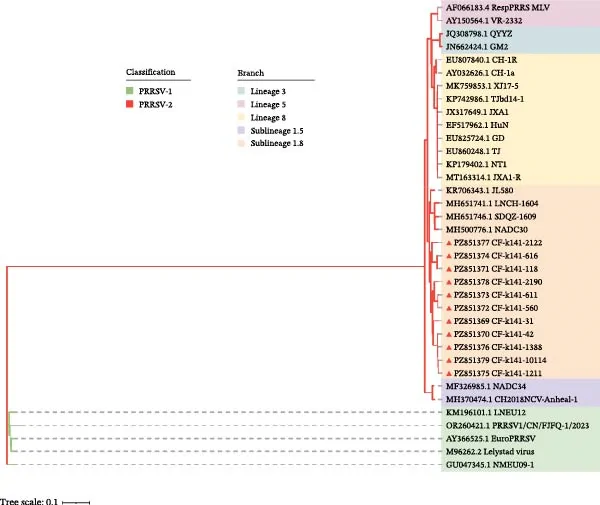
Whole‐genome phylogenetic analysis of the 11 successfully assembled PRRSV genomes obtained in this study. A phylogenetic tree was constructed using the complete genome sequences of 11 strains obtained in this study and reference PRRSV strains from different lineages. The PRRSV strains obtained in this study are indicated by red triangles. PRRSV‐1 reference strains were included for comparison and formed a separate branch.

Complete or near‐complete PRRSV genomes suitable for downstream analysis were successfully assembled from 11 of the 21 samples subjected to whole‐genome sequencing. The 11 genomes originated from six production units and were collected between August 27 and October 15, 2025 (Table S4). Whole‐genome phylogenetic analysis revealed that all 11 strains clustered within the PRRSV‐2 sublineage 1.8 and were closely related to the NADC30‐like reference strains (Figure 3). None of the study‐derived complete genomes clustered with PRRSV‐1, lineage 3, lineage 5, lineage 8, or NADC34‐like reference strains. Thus, the successfully recovered whole‐genome dataset consisted exclusively of NADC30‐like PRRSVs. Because these 11 genomes represented successful assemblies from the 21 samples subjected to sequencing rather than a lineage‐stratified sampling set, their lineage composition should not be interpreted as representative of the complete lineage distribution observed in the larger ORF5 dataset.

Serial whole‐genome sequences obtained at different sampling times were available from CF‐7, CF‐15, and CF‐25, allowing short‐term longitudinal comparison within individual production units (Table S9). In CF‐7, CF‐k141‐31, collected on August 27, 2025, and CF‐k141‐118, collected on October 4, 2025, shared 89.6% whole‐genome nucleotide identity over a 38‐day interval. In CF‐15, CF‐k141‐611 and CF‐k141‐616, collected 4 days apart, shared 89.9% identity. Three genomes were recovered from CF‐25 at different time points. CF‐k141‐1211 and CF‐k141‐560, collected 10 days apart, shared 90.2% identity, whereas CF‐k141‐560 and CF‐k141‐10114, collected 17 days apart, shared 90.1% identity. Notably, CF‐k141‐1211 and CF‐k141‐10114, collected 27 days apart, showed 100.0% whole‐genome nucleotide identity at the reported analytical precision. These comparisons revealed substantial short‐term genomic heterogeneity within individual production units. In particular, the ~90% identities observed between several serial genomes are more consistent with the sequential detection of genetically distinct NADC30‐like variants than with the simple short‐term accumulation of mutations within a single viral lineage. Conversely, the complete nucleotide identity observed between CF‐k141‐1211 and CF‐k141‐10114 suggests the persistence or repeated detection of a highly similar viral genome within CF‐25 over approximately 4 weeks. Two additional genomes, CF‐k141‐1388 and CF‐k141‐2122, were obtained from CF‐17 on the same date and shared 88.0% nucleotide identity, further illustrating the contemporaneous within‐unit genomic diversity, although this pair was not considered a longitudinal comparison.

### 3.4. Whole‐Genome Identity Analysis of PRRSV

To comprehensively evaluate the genomic relatedness of PRRSV strains obtained in this study, whole‐genome nucleotide identities were calculated among all 11 study‐derived strains and between these strains and representative PRRSV reference strains. The 11 study‐derived strains shared 87.3%–100.0% whole‐genome nucleotide identity with one another (Table S10), indicating substantial overall genetic relatedness but also appreciable genomic diversity among the NADC30‐like strains. Several strain pairs exhibited particularly high nucleotide identities, including CF‐k141‐1211 and CF‐k141‐10114 (100.0%), CF‐k141‐1211 and CF‐k141‐1388 (99.6%), CF‐k141‐1388 and CF‐k141‐10114 (99.6%), and CF‐k141‐118 and CF‐k141‐616 (99.4%).

Compared with representative PRRSV reference strains, the whole‐genome nucleotide identities of the 11 study‐derived strains ranged from 81.2% to 88.2% (Table S11). For each study‐derived strain, the highest whole‐genome nucleotide identity among the reference strains included in the analysis was observed with one of the NADC30‐like strains, including JL580, NADC30, or LNCH‐1604. This genomic similarity pattern was consistent with the whole‐genome phylogenetic analysis, which classified all 11 strains within PRRSV‐2 sublineage 1.8.

**Figure 4:**
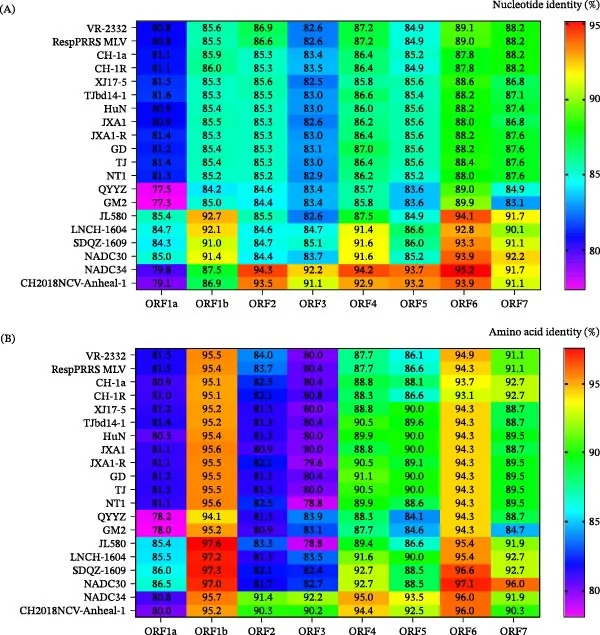
Nucleotide and amino acid identity analysis of the representative strain CF‐k141‐31. (A) Nucleotide identity heatmap comparing the ORF1a, ORF1b, and ORF2–ORF7 regions of CF‐k141‐31 with those of representative PRRSV reference strains. (B) Amino acid identity heatmap comparing the corresponding deduced proteins of CF‐k141‐31 with those of the reference strains. The color scale indicates the sequence identity percentage.

To further examine region‐specific genetic variation, CF‐k141‐31 was selected for detailed ORF‐level analysis because it exhibited heterogeneous similarity across different genomic regions. The nucleotide identity of CF‐k141‐31 with the reference strains varied across genomic regions, ranging from ~77.3% to 95.2% (Figure 4A). Similarly, the amino acid identity varied among different ORFs, ranging from ~78.0% to 97.6% (Figure 4B). CF‐k141‐31 showed relatively high similarity to lineage 1 reference strains in several genomic regions, which is consistent with its NADC30‐like classification. However, the highest identity was not consistently associated with a single reference strain across all the ORFs, suggesting region‐specific genetic divergence and a possible mosaic genomic background.

### 3.5. GP5 Amino Acid Variation in Major Antigenic Regions

**Figure 5:**
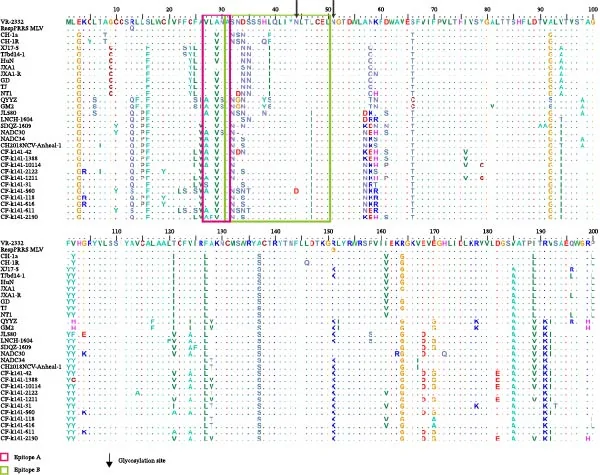
Alignment of the GP5 amino acid sequences of representative PRRSV strains. The deduced GP5 amino acid sequences of 11 representative strains obtained in this study were aligned with those of representative PRRSV reference strains. Amino acid positions are numbered relative to the VR‐2332 reference strain. The decoy epitope A, neutralizing epitope B, and predicted N‐linked glycosylation sites are indicated. Dots represent amino acids identical to the reference sequence, and substitutions are shown by single‐letter amino acid codes.

The deduced GP5 amino acid sequences of the 11 study‐derived strains were aligned with those of PRRSV reference strains to assess variation in antigenically relevant regions. Multiple amino acid substitutions were observed in the GP5 ectodomain, especially in the signal peptide region, decoy epitope A, neutralizing epitope B, and adjacent glycosylation‐related regions (Figure 5). Within epitope A, the sequence patterns of the study strains were more similar to those of the NADC30‐like/NADC34‐like lineage 1 strains than to those of the classical or HP‐PRRSV strains (V27‐A27). Several strains also exhibited amino acid substitutions in or near the neutralizing epitope B region (S32‐N32 and L47‐I47), indicating antigenic divergence from classical vaccine‐related strains. Overall, the GP5 amino acid alignment indicated that the circulating strains were antigenically divergent from the classical PRRSV and HP‐PRRSV reference strains and displayed lineage 1‐associated variation in key immune‐related regions. These mutations may contribute to differences in antigenicity, although their biological effects require further validation by neutralization or vaccine cross‐protection assays.

### 3.6. Nsp2 Deletion Patterns of Representative Strains

**Figure 6:**
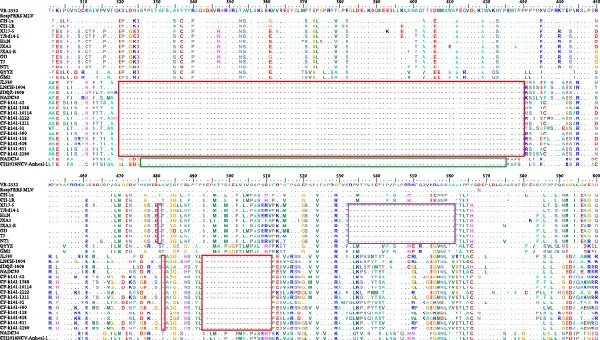
Alignment of Nsp2 amino acid sequences of representative PRRSV strains. The Nsp2 amino acid sequences of representative strains obtained in this study were aligned with those of reference strains from different PRRSV lineages. Amino acid positions are numbered relative to the VR‐2332 reference strain. The 11 study‐derived strains showed the characteristic 111 + 1 + 19 discontinuous deletion pattern of NADC30‐like PRRSV in the Nsp2 hypervariable region (red box), which differed from the 1 + 29 discontinuous deletion of HP‐PRRSV‐like strains (purple box) and the 100‐amino acid continuous deletion of NADC34‐like strains (green box).

To further characterize the molecular signatures of the representative strains, Nsp2 amino acid sequences were compared with those of reference strains from different PRRSV lineages. The 11 strains obtained in this study exhibited a large discontinuous deletion pattern (111 + 1 + 19) in the Nsp2 hypervariable region that was consistent with that of the NADC30‐like PRRSV. Specifically, the representative strains exhibited the characteristic NADC30‐like deletion pattern, including the major deletion region corresponding to the 111‐amino acid deletion and additional discontinuous deletions in the downstream Nsp2 region (Figure 6). This pattern was distinct from the 30‐amino acid discontinuous deletion characteristic (1 + 29) of HP‐PRRSV‐like strains and the 100‐amino acid continuous deletion typical of NADC34‐like strains. The Nsp2 deletion profile therefore supported the whole‐genome phylogenetic classification of these strains as NADC30‐like PRRSVs.

### 3.7. Recombination Analysis of PRRSV Complete Genomes

**Figure 7:**
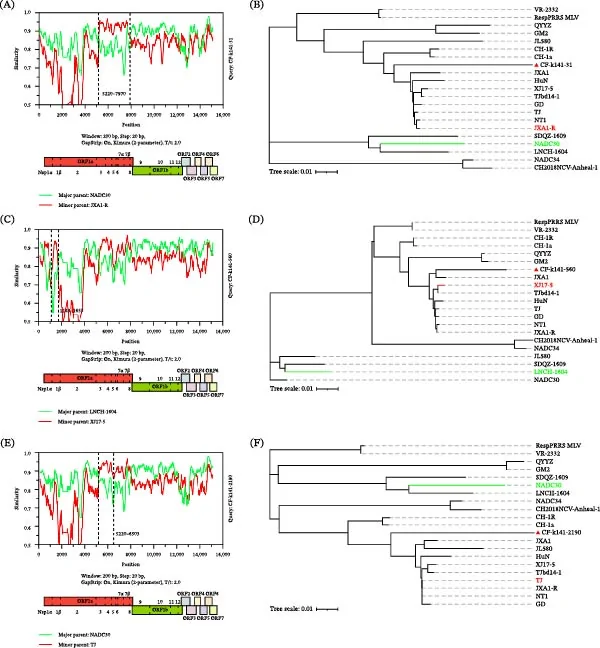
Recombination analysis of representative NADC30‐like PRRSV strains. Potential recombination events were screened using RDP4 and further analyzed using SimPlot 3.5. (A, C, and E) Similarity plots of CF‐k141‐31, CF‐k141‐560, and CF‐k141‐2190, respectively. The analyses were performed using a 200‐bp window and a 20‐bp step under the Kimura two‐parameter model with gap‐stripping enabled. The predicted recombinant regions are indicated above the genome structure. (B, D, and F) Phylogenetic trees constructed on the basis of the corresponding recombinant fragments. The target strains (CF‐k141‐31, CF‐k141‐560, and CF‐k141‐2190) are indicated by red triangles.

Potential recombination events were first screened using RDP4 software. Three study‐derived strains, namely, CF‐k141‐31, CF‐k141‐560, and CF‐k141‐2190, were identified as potential recombinant viruses (Table S12). To further validate the recombination signals and determine the recombination breakpoints, SimPlot 3.5 was used for similarity plot and recombination breakpoint analyses. For CF‐k141‐31, NADC30 was inferred as the putative major parent and JXA1‐R was inferred as the putative minor parent, with a predicted recombinant region at nt 5220–7970 (Figure 7A). Phylogenetic analysis of this region revealed that CF‐k141‐31 clustered closer to lineage 8‐related strains, supporting the recombination signal (Figure 7B). For CF‐k141‐560, LNCH‐1604, and XJ17‐5 were inferred as the putative major and minor parents, respectively, with a predicted recombinant region at nt 1143–1655 (Figure 7C). The phylogenetic tree based on this fragment showed a topology shift relative to that of the whole‐genome tree (Figure 7D). For CF‐k141‐2190, NADC30 and TJ were inferred as the putative major and minor parents, respectively, with a recombinant region at nt 5220–6503 (Figure 7E). The recombinant fragment tree placed CF‐k141‐2190 closer to the HP‐PRRSV‐related lineage 8 strains (Figure 7F).

Taken together, these results demonstrated that although the study‐derived complete‐genome strains were classified as NADC30‐like PRRSVs, several of them contained recombinant fragments derived from other PRRSV lineages, particularly HP‐PRRSV‐like or vaccine‐related lineage 8 strains. These findings indicated that recombination contributed to the genomic diversification of the NADC30‐like PRRSV circulating in this production system.

## 4. Discussion

PRRSV remains one of the most difficult swine pathogens to control because of its persistent circulation, extensive genetic diversity, immune evasion, and frequent recombination [1, 25, 26]. In China, the epidemiology of PRRSV has undergone several major transitions, from classical PRRSV to HP‐PRRSV and, more recently, to the increasing circulation of lineage 1 strains, including NADC30‐like and NADC34‐like PRRSVs [2, 27–31]. In the present study, we integrated routine large‐scale RT‐qPCR, ORF5‐based phylogenetic analysis, and whole‐genome sequencing to characterize PRRSV circulation in a large‐scale integrated swine production system in Chifeng, Inner Mongolia, northern China, from 2023 to 2026. The results revealed that PRRSV detection was characterized by temporal fluctuations, farm‐level heterogeneity, production‐stage‐ and sample‐type‐dependent positivity, a predominance of PRRSV‐2 lineage 1 strains, and the genomic diversification of representative NADC30‐like strains through GP5 variation and recombination.

A major feature of this study is the large longitudinal surveillance dataset from a single integrated production system. Previous studies have provided important information on PRRSV prevalence and genetic diversity in China, but most have focused on provincial, regional, or national surveillance datasets based mainly on clinical samples [15, 16, 18, 32, 33]. In contrast, our study analyzed 380,050 PRRSV nucleic acid testing records from multiple farms, production stages, and sample types within the same production network. This design allowed us to evaluate PRRSV dynamics at a more operationally relevant resolution. The overall PRRSV positivity rate was 9.60%, although both the testing volume and positivity varied considerably over time. Importantly, the surveillance intensity was not constant throughout the study period. PRRSV monitoring was conducted at a relatively low routine frequency in 2023, was progressively strengthened during 2024–2025, and was further intensified in 2026 following implementation of a PRRSV stabilization/elimination program when routine testing shifted from predominantly monthly surveillance to weekly and/or production‐batch‐based testing. Therefore, the annual positivity rates reported in this study represent the proportion of positive tests generated under routine herd health surveillance rather than population‐level prevalence estimates derived from a fixed sampling design. Direct quantitative comparisons of positivity rates among years should consequently be interpreted with caution. Although meteorological, pig flow, and management factors were not directly analyzed, the location of the production system in northern China implies that seasonal housing, ventilation constraints, and cold weather management may influence viral maintenance and amplification. Recent molecular epidemiological studies in northern China have also shown active circulation and genetic diversification of PRRSV lineage 1 strains, supporting the need for continued surveillance in this region [16, 18].

The results of the farm‐month heatmap further demonstrated that PRRSV positivity was not evenly distributed across production units. Several farms showed recurrent or concentrated positivity during specific time windows, suggesting that local amplification points existed within the integrated system. The production units were epidemiologically interconnected through movements of breeding, replacement, nursery, and finishing pigs, and their production functions, herd sizes, vaccination practices, and surveillance intensities differed. Consequently, elevated PRRSV detection in one unit may reflect both local viral circulation and epidemiological connections with upstream or downstream units. However, the present study did not formally model pig movements or management variables as causal predictors of PRRSV transmission; therefore, the observed production‐unit associations should not be interpreted as direct evidence of transmission between specific farms.

The results of the production‐stage analysis revealed that nursery pigs had the highest prevalence of PRRSV, followed by finishing pigs and gilts, whereas boars had the lowest prevalence. This pattern is consistent with the biology and management of PRRSV infection. Nursery pigs are frequently exposed to stress, waning maternal antibodies, mixing, and respiratory coinfections, which may facilitate viral amplification and transmission. The relatively high positivity in gilts also deserves attention because gilt acclimation and movement are key components of herd stability and may contribute to viral introduction or redistribution within production systems. In contrast, the low positivity in boars may reflect strict monitoring and biosecurity in boar‐related management, although semen surveillance remains necessary because PRRSV can be detected in boar semen and may pose reproductive and dissemination risks [34, 35].

Sample‐type analysis also provided practical implications for PRRSV monitoring. Castration/tail‐docking fluid, tongue‐tip fluid, and tissue/organ samples presented higher positivity rates than serum and semen‐related samples did. Processing fluids have been increasingly used for PRRSV surveillance because they provide practical litter‐ or group‐level samples for monitoring breeding herds [36, 37]. Oral fluids and related group‐level samples are also useful for population‐level PRRSV detection because they are less invasive and can represent multiple animals within a pen or group [34, 35]. Recent studies have further suggested that tongue fluid can be useful for detecting PRRSV vertical transmission and herd instability under certain conditions [38]. In this context, the high positivity observed in the processing fluid and tongue‐tip fluid in our study supports their value as high‐yield surveillance samples. Overall, these findings support a risk‐based surveillance strategy in which nursery pigs, finishing pigs, gilts, and farms with recurrent positivity are treated as key monitoring targets, while processing fluid, tongue‐tip fluid, oral fluid, and clinically affected tissue samples are selected according to the production stage and monitoring purpose. When PRRSV positivity increases, particularly during high‐risk seasonal periods, rapid measures such as restricting internal pig movement, strengthening all‐in/all‐out management, evaluating herd immune status, and tracing possible infection sources should be implemented to reduce the within‐system spread.

ORF5‐based phylogenetic analysis revealed that all sequences obtained in this study belonged to PRRSV‐2; the PRRSV‐1 cluster shown in the phylogenetic tree consisted only of reference strains. These results are consistent with the long‐standing predominance of PRRSV‐2 in Chinese swine herds [2, 28–30, 32]. In our ORF5 dataset, lineage 1 was dominant, with sublineage 1.8 accounting for 53.74% and sublineage 1.5 accounting for 34.69%. These results indicate that NADC30‐like and NADC34‐like PRRSVs were the major circulating lineages in this production system. This finding is consistent with recent reports showing the increased importance of lineage 1 PRRSV in China, especially in northern regions and other major pig‐producing regions [4, 9, 15, 16, 18, 39, 40]. The detection of lineage 5 and lineage 8 at lower proportions indicates that multiple PRRSV‐2 lineages cocirculate in the system, which may increase the likelihood of recombination when different lineages infect the same herd or production network.

The production unit‐resolved ORF5 analysis provided additional insight into the longitudinal dynamics of PRRSV within the integrated production system. Repeated recovery of ORF5 sequences from the same production units over multiple sampling time points and years indicated both the persistence of circulating viral populations and temporal changes in lineage composition. Moreover, genetically distinct PRRSV‐2 lineages were occasionally detected contemporaneously within the same production unit. Such within‐unit cocirculation increases the opportunity for genetically distinct viral populations to encounter one another and may provide an epidemiological context favorable for recombination. Nevertheless, production‐unit‐level codetection does not demonstrate simultaneous infection of individual pigs, and animal‐level sampling or deep sequencing approaches would be required to establish coinfection with multiple PRRSV strains.

Whole‐genome phylogeny further confirmed that all 11 study‐derived complete or near‐complete genome sequences belonged to the NADC30‐like PRRSV. The combined use of ORF5 analysis and whole‐genome sequencing is important because ORF5 alone may not fully reflect the genomic origin of recombinant strains [41, 42]. Whole‐genome sequencing has been shown to improve PRRSV genomic surveillance and provide more accurate information on viral transmission, recombination, and lineage assignment than single‐gene analysis does [41]. In the present study, ORF5 sequencing revealed the broader lineage composition of circulating PRRSV strains, whereas whole‐genome sequencing clarified the genomic background of representative lineage 1 strains. This integrated approach provides a more reliable framework for understanding PRRSV evolution within large‐scale production systems.

Short‐term longitudinal comparison of whole genomes recovered from the same production units provided additional insight into within‐unit viral diversity. Serial genomes from CF‐7 and CF‐15 shared only 89.6% and 89.9% nucleotide identity, respectively, despite being collected 38 and 4 days apart. Similarly, in CF‐25, CF‐k141‐560 shared ~90% whole‐genome identity with genomes obtained both before and after its sampling date, whereas CF‐k141‐1211 and CF‐k141‐10114, collected 27 days apart, were identical at the reported analytical precision. These patterns suggest that multiple genetically distinct NADC30‐like variants could circulate sequentially or concurrently within the same production unit rather than representing a simple stepwise evolution of a single viral population. At the same time, the persistence of a highly similar genome over several weeks indicates that individual viral variants may remain detectable within a production unit. Because the serial samples were not collected from the same animals, however, these observations should be interpreted as production‐unit‐level viral population dynamics rather than direct ancestor–descendant evolution.

Nsp2 amino acid alignment revealed that the 11 study‐derived strains shared the characteristic 111 + 1 + 19 discontinuous deletion pattern of NADC30‐like PRRSV. This deletion pattern is a recognized molecular signature of NADC30‐like strains and differs from the 1 + 29 discontinuous deletion of HP‐PRRSV‐like strains and the 100‐amino acid continuous deletion commonly observed in NADC34‐like PRRSV [4, 9, 39, 40]. The consistency between the whole‐genome phylogeny and Nsp2 deletion analysis results strengthens the classification of the study‐derived strains as NADC30‐like PRRSVs. However, Nsp2 deletion patterns should not be interpreted as the sole markers of pathogenicity because previous studies have shown that PRRSV pathogenicity is influenced by multiple genomic regions, recombination backgrounds, and host factors [4, 9, 40, 43].

GP5 amino acid analysis revealed substitutions in the ectodomain, particularly around decoy epitope A, neutralizing epitope B, and predicted N‐linked glycosylation sites. GP5 is an important structural protein involved in antigenicity and neutralizing antibody recognition. Epitope A has been described as a decoy epitope, whereas epitope B is associated with neutralizing antibody recognition [44]. N‐linked glycosylation in GP5 can influence virus infectivity and antigenicity and induce neutralizing antibodies, and glycan shielding has been proposed as one mechanism through which PRRSV reduces neutralizing antibody recognition [45, 46]. In this study, representative NADC30‐like strains presented lineage 1‐associated GP5 patterns but also displayed strain‐specific substitutions around immune‐related regions. These changes suggest ongoing antigenic diversification within the circulating NADC30‐like population. Nevertheless, sequence‐based prediction alone cannot be used to predict antigenic escape or vaccine failure. Neutralization assays, cross‐protection studies, and reverse genetics experiments are needed to clarify the biological significance of these changes in GP5 expression.

Recombination analysis revealed potential recombinant events in CF‐k141‐31, CF‐k141‐560, and CF‐k141‐2190. Although these strains were classified as NADC30‐like PRRSVs on the basis of whole‐genome phylogeny and Nsp2 signatures, SimPlot and fragment‐specific phylogenetic analyses suggested that some genomic fragments may have originated from lineage 8‐related strains, including HP‐PRRSV‐like or vaccine‐related strains. Recombination is a major driver of PRRSV diversity and has been repeatedly reported among NADC30‐like, HP‐PRRSV‐like, QYYZ‐like, NADC34‐like, and vaccine‐related strains [47, 48]. NADC30‐like strains appear to have particularly high recombination potential, and recombinant NADC30‐like viruses have been frequently reported in China [4, 39, 40, 47]. The detection of lineage 8‐related recombinant fragments in the present study indicates that historical or ongoing interactions between lineage 1 strains and lineage 8‐related viruses may contribute to the genomic mosaicism of PRRSV in this production system.

The possible involvement of vaccine‐related or HP‐PRRSV‐like fragments also has implications for PRRSV control. Modified live vaccines remain widely used for PRRS control, but their safety, recombination risk, and limited heterologous protection remain concerns, particularly in environments where genetically diverse field strains cocirculate [48]. The present study does not directly demonstrate vaccine‐derived transmission or vaccine‐strain reversion, and the parental assignments inferred by recombination analysis should be interpreted cautiously. However, the predominance of lineage 1 strains and the detection of recombinant NADC30‐like viruses indicate that vaccination and control programs should not rely solely on historical lineage information or ORF5‐based typing. Routine ORF5 sequencing should be complemented by periodic whole‐genome sequencing, especially when PRRSV positivity increases, clinical signs change, or vaccine performance appears unsatisfactory. Vaccine selection and immunization programs should be periodically reassessed according to currently circulating strains, while biosecurity, gilt acclimation, pig flow control, and rapid elimination of local infection sources should remain central components of PRRS control in large‐scale production systems.

This study provides a longitudinal, system‐level view of PRRSV circulation in a large‐scale swine production network in northern China by integrating routine RT‐qPCR surveillance, ORF5 phylogeny, and whole‐genome sequencing. This design allowed us to connect farm‐, time‐, production‐stage‐, and sample‐type‐specific detection patterns with viral lineage composition, Nsp2 deletion signatures, GP5 variation, and recombination signals, offering practical evidence for targeted surveillance rather than simple prevalence estimation. However, several limitations should be considered when interpreting the present findings. First, this study was conducted within a single integrated production system and relied primarily on routine diagnostic surveillance, resulting in uneven sampling intensity across years, production units, and sample types. Second, although production‐unit‐level pig flow and vaccination information was available, these management variables were not incorporated into a formal transmission model, and detailed animal‐level movement histories and longitudinal herd immune status data were unavailable. Third, the whole‐genome dataset had limited temporal and genetic coverage. Although 21 PRRSV‐positive samples were subjected to whole‐genome sequencing, only 11 yielded complete or near‐complete genomes suitable for downstream analysis, all of which belonged to sublineage 1.8 and were collected from August to October 2025. Therefore, the whole‐genome findings primarily characterize the successfully recovered NADC30‐like viruses and should not be considered fully representative of the broader PRRSV diversity observed in the ORF5 dataset. Finally, the absence of virus isolation, pathogenicity testing, and neutralization assays limited the interpretation of the biological and antigenic consequences of the observed genetic variation. Future studies integrating systematic serial whole‐genome sequencing with detailed animal movement networks, herd immune status, vaccination histories, and experimental validation are needed to more comprehensively define PRRSV evolution and transmission within large‐scale production systems.

In conclusion, this study demonstrated that PRRSV circulation in a large‐scale swine production system in Chifeng, Inner Mongolia, was characterized by temporal fluctuations, farm‐level heterogeneity, stage‐ and sample‐type‐dependent positivity, and a predominance of PRRSV‐2 lineage 1 strains. The 11 study‐derived whole‐genome sequences were identified as NADC30‐like PRRSVs and showed typical Nsp2 deletion signatures, GP5 antigenic region variation, and potential recombinant fragments involving lineage 8‐related strains. These findings support the integration of routine molecular detection, ORF5 sequencing, and whole‐genome surveillance for PRRSV control and highlight the need for targeted monitoring of high‐risk farms, production stages, and sample types within large‐scale swine production systems.

## Author Contributions

Junlong Zhao and Xujiao Ren conceived the project and supervised the overall study. Tao Zhang, Junlong Zhao, and Anding Zhang designed the study. Shaofu Song, Hechao Zhu, Shixuan Zhu, Yanjiao Wang, Kui Hou, Rui Liu, and Xujiao Ren performed the bench experiments and data analysis. Tao Zhang wrote the first draft of the manuscript. Tao Zhang, Shaofu Song, Hechao Zhu, Shixuan Zhu, Yanjiao Wang, Anding Zhang, Kui Hou, Rui Liu, Xujiao Ren, and Junlong Zhao critically revised and edited the manuscript.

## Funding

This work was supported by the China Postdoctoral Science Foundation (Certificate Number: 2024M753453).

## Disclosure

All the authors read and approved the final manuscript.

## Ethics Statement

This study was based on the retrospective analysis of diagnostic records and residual samples collected during routine herd health monitoring in a commercial swine production system. No animals were subjected to experimental procedures, additional sampling, treatment, or intervention for the purposes of this study. Therefore, institutional animal ethics approval was not required, and no approval ID was issued.

## Conflicts of Interest

The authors declare no conflicts of interest.

## Supporting Information

Additional supporting information can be found online in the Supporting Information section.

## Supporting information


**Supporting Information** The supporting information associated with this article includes Figure S1 and Tables S1–S12, which provide additional methodological, epidemiological, and molecular data supporting the analyses presented in the main text. Figure S1: Illustrates the temporal dynamics and production‐unit distribution of ORF5‐defined PRRSV‐2 lineages from 2023 to 2026. Tables S1–S6: Provide detailed information on production‐unit characteristics, pig‐flow and vaccination practices, primers and probes used for PRRSV detection and ORF5 amplification, the temporal and production‐unit distribution of the 441 ORF5 sequences, epidemiological metadata of the 11 successfully recovered whole genomes, production‐unit‐specific RT‐qPCR detection results, and contemporaneous detection of multiple PRRSV lineages/sublineages. Tables S7–S12: Present additional ORF5 and whole‐genome sequence identity analyses, short‐term longitudinal whole‐genome comparisons, comparisons with representative PRRSV reference strains, and recombination analysis of the study‐derived genomes. Data S1: Contains the nucleotide sequences of all 441 study‐derived ORF5 genes used for phylogenetic and sequence analyses.

## Data Availability

The 11 complete or near‐complete PRRSV genome sequences generated in this study have been deposited in GenBank under accession numbers PZ851369–PZ851379. The nucleotide sequences of all 441 study‐derived ORF5 genes used for phylogenetic and sequence analyses are provided in Data S1. Additional data supporting the findings of this study are available from the corresponding author upon reasonable request.
