# Supplementary material for: Longitudinal Molecular Epidemiology and Genomic Evolution of Porcine Reproductive and Respiratory Syndrome Virus in a Large‐Scale Swine Production System in Northern China, 2023–2026
Source: Transbound Emerg Dis. 2026 Sep 22;2026:6131183. doi: 10.1155/tbed/6131183 (PMC13594771; doi:10.1155/tbed/6131183)
Supplement: Supplementary file 1 [file TBED-2026-6131183-s001.zip › Supplementary Materials.docx]

**Supplementary Materials for**

**Longitudinal Molecular Epidemiology and Genomic Evolution of Porcine Reproductive and Respiratory Syndrome Virus in a Large-Scale Swine Production System in Northern China, 2023-2026**

Tao Zhang^1,2^, Shaofu Song^2^, Hechao Zhu^3^, Shixuan Zhu^3^, Yanjiao Wang^2^, Anding Zhang^1^, Kui Hou^2^, Rui Liu^2^, Xujiao Ren^4^, Junlong Zhao^1^

^1^National Key Laboratory of Agricultural Microbiology, College of Veterinary Medicine, Huazhong Agricultural University, Wuhan 430070, Hubei, China

^2^COFCO Joycome (Chifeng) Co., Ltd., Chifeng 024000, Inner Mongolia, China

^3^COFCO Joycome Foods Limited, Beijing 100020, China

^4^CAS Key Laboratory of Pathogen Microbiology and Immunology, Institute of Microbiology, Chinese Academy of Sciences (CAS), Beijing 100101, China

**Correspondence:** Xujiao Ren (renxujiao92@163.com); Junlong Zhao (zhaojunlong@mail.hzau.edu.cn)

**FIGURE S1** Temporal dynamics and production-unit distribution of ORF5-defined PRRSV-2 lineages from 2023 to 2026. (A) Annual proportional distribution of the 441 study-derived ORF5 sequences among sublineage 1.8, sublineage 1.5, lineage 5, lineage 8, and other/unassigned lineages. Numbers above the bars indicate the total number of ORF5 sequences obtained in each year. (B) Distribution of ORF5-defined PRRSV-2 lineages/sublineages across the 29 production units. Cell values indicate the number of sequences assigned to each lineage/sublineage within the corresponding production unit, with color intensity representing sequence abundance.

**TABLE S1** Characteristics, pig-flow patterns, animal introduction practices, and PRRSV vaccination status of the production units included in this study

| **Production unit** | **Production type** | **Major production stage(s)** | **Approx. herd/design capacity (head)** | **Main pig source(s)** | **Main destination(s)** | **Animal introduction/quarantine practice** | **PRRSV vaccination status** | **Vaccine/product and schedule** |
| --- | --- | --- | --- | --- | --- | --- | --- | --- |
| CF-1 | Commercial sow farm | Breeding; farrowing/lactation | 4,800 | CF-8; CF-16; CF-26 | CF-2 | 45-day quarantine; PRRSV-negative throat swab required before entry | Vaccinated | R98 vaccine; twice annually |
| CF-2 | Nursery/finishing unit | Nursery; finishing | 40,000 | CF-1 | Sale/slaughter | Not specified / not applicable | Not vaccinated | — |
| CF-3 | Commercial sow farm | Breeding; farrowing/lactation | 4,800 | CF-8; CF-16; CF-26 | CF-4 | 45-day quarantine; PRRSV-negative throat swab required before entry | Vaccinated | R98 vaccine; twice annually |
| CF-4 | Nursery/finishing unit | Nursery; finishing | 40,000 | CF-3 | Sale/slaughter | Not specified / not applicable | Not vaccinated | — |
| CF-5 | Commercial sow farm | Breeding; farrowing/lactation | 4,800 | CF-8; CF-16; CF-26 | CF-6 | 45-day quarantine; PRRSV-negative throat swab required before entry | Vaccinated | R98 vaccine; twice annually |
| CF-6 | Nursery/finishing unit | Nursery; finishing | 40,000 | CF-5 | Sale/slaughter | Not specified / not applicable | Not vaccinated | — |
| CF-7 | GP breeding/multiplication unit | Breeding; farrowing/lactation | 4,800 | CF-8; CF-Guancang | CF-8 | 45-day quarantine; PRRSV-negative throat swab required before entry | Vaccinated | R98 vaccine; twice annually |
| CF-8 | GP nursery/finishing multiplication unit | Nursery; finishing; replacement gilts | 40,000 | CF-7 | CF-1; CF-3; CF-5; CF-7; CF-9; CF-11; CF-13; sale | Not specified in field form | Vaccinated | R98 vaccine; four immunizations per batch |
| CF-9 | Commercial sow farm | Breeding; farrowing/lactation | 4,800 | CF-8; CF-16; CF-26 | CF-10 | 45-day quarantine; PRRSV-negative throat swab required before entry | Vaccinated | R98 vaccine; twice annually |
| CF-10 | Nursery/finishing unit | Nursery; finishing | 40,000 | CF-9 | Sale/slaughter | Not specified / not applicable | Not vaccinated | — |
| CF-11 | Commercial sow farm | Breeding; farrowing/lactation | 4,800 | CF-8; CF-16; CF-26 | CF-12 | 45-day quarantine; PRRSV-negative throat swab required before entry | Vaccinated | R98 vaccine; twice annually |
| CF-12 | Nursery/finishing unit | Nursery; finishing | 40,000 | CF-11 | Sale/slaughter | Not specified / not applicable | Not vaccinated | — |
| CF-13 | Commercial sow farm | Breeding; farrowing/lactation | 4,800 | CF-8 | CF-16 | 45-day quarantine; PRRSV-negative throat swab required before entry | Vaccinated | R98 vaccine; twice annually |
| CF-15 | Commercial sow farm | Breeding; farrowing/lactation | 4,800 | CF-8; CF-16 | Outdoor placement / transfer as recorded | 45-day quarantine; PRRSV-negative throat swab required before entry | Vaccinated | R98 vaccine; twice annually |
| CF-16 | Nursery/finishing unit | Nursery; finishing | 40,000 | CF-15; CF-13 | Sale/slaughter | Not specified / not applicable | Not vaccinated | — |
| CF-17 | Commercial sow farm | Breeding; farrowing/lactation; replacement gilts; nursery; finishing; boars | 4,800 | Internal CF-17; CF-Guancang | CF-18 | 90-day quarantine for animals from CF-Guancang; PRRSV-negative throat swab before entry | Vaccinated | R98 vaccine; twice annually |
| CF-18 | Nursery/finishing unit | Nursery; finishing | 40,000 | CF-17 | Sale/slaughter | Not specified / not applicable | Not vaccinated | — |
| CF-19 | Commercial sow farm | Breeding; farrowing/lactation; replacement gilts; nursery; finishing; boars | 4,800 | Internal CF-19; CF-Guancang | CF-20 | 90-day quarantine for animals from CF-Guancang; PRRSV-negative throat swab before entry | Vaccinated | R98 vaccine; twice annually |
| CF-20 | Nursery/finishing unit | Nursery; finishing | 40,000 | CF-19 | Sale/slaughter | Not specified / not applicable | Not vaccinated | — |
| CF-21 | Commercial sow farm | Breeding; farrowing/lactation; replacement gilts; nursery; finishing; boars | 4,800 | Internal CF-21; CF-Guancang | CF-22 | 90-day quarantine for animals from CF-Guancang; PRRSV-negative throat swab before entry | Vaccinated | R98 vaccine; twice annually |
| CF-22 | Nursery/finishing unit | Nursery; finishing | 40,000 | CF-21 | Sale/slaughter | Not specified / not applicable | Not vaccinated | — |
| CF-23 | Commercial sow farm | Breeding; farrowing/lactation; replacement gilts; nursery; finishing; boars | 4,800 | Internal CF-23; CF-Guancang | CF-24 | 90-day quarantine for animals from CF-Guancang; PRRSV-negative throat swab before entry | Vaccinated | R98 vaccine; twice annually |
| CF-24 | Nursery/finishing unit | Nursery; finishing | 40,000 | CF-23 | Sale/slaughter | Not specified / not applicable | Not vaccinated | — |
| CF-25 | GP breeding/multiplication unit | Breeding; farrowing/lactation; replacement gilts; nursery; finishing; boars | 4,800 | CF-Guancang | CF-26 | 90-day quarantine for animals from CF-Guancang; PRRSV-negative throat swab before entry | Vaccinated | R98 vaccine; twice annually |
| CF-26 | GP nursery/finishing multiplication unit | Nursery; finishing; replacement gilts | 40,000 | CF-25 | CF-1; CF-3; CF-5; CF-11; sale | Not specified in field form | Vaccinated | R98 vaccine; four immunizations per batch |
| CF-Guancang | GGP nucleus unit | Breeding; farrowing/lactation; replacement gilts; nursery; finishing; boars | 22,400 | Internal nucleus herd | CF-7; CF-17; CF-19; CF-21; CF-23; CF-25; internal nucleus; Yigong; Ergong; Sangong | 28-day quarantine/immunization period; two rounds of selection; PRRSV antigen and antibody testing before entry | Not vaccinated | — |
| Yigong | Boar station | Boars | 300 | CF-Guancang (field form also records boars introduced from Kepulixin) | Culling | Boars introduced from Kepulixin; 28-day quarantine/immunization; whole-group PRRSV antigen/antibody testing on days 1, 7, and 21 | Not vaccinated | — |
| Ergong | Boar station | Boars | 300 | CF-Guancang | Culling | Animals introduced from CF-Guancang; 28-day quarantine/immunization; whole-group PRRSV antigen/antibody testing on days 1, 7, and 21 | Not vaccinated | — |
| Sangong | Boar station | Boars | 300 | CF-Guancang | Culling | Animals introduced from CF-Guancang; 28-day quarantine/immunization; whole-group PRRSV antigen/antibody testing on days 1, 7, and 21 | Not vaccinated | — |

Note: “Not specified” indicates that the source form did not provide additional introduction/quarantine details for that unit.

**TABLE S2** Primers and probes used for PRRSV RT-qPCR detection and ORF5 amplification in this study

| **Primers** | **Sequence (5’ to 3’)** | **Purpose** |
| --- | --- | --- |
| PRRSV-NA-F | AGATGCCGTTTGTGCTTGCT | PRRSV-2 detection |
| PRRSV-NA-R | TGCCGCAATCGGATGAA |  |
| PRRSV-NA-P | ACATTCTGGCCCCTGC |  |
| PRRSV-EU-F | TYACTTCCAGATGCAGATTGTGT | PRRSV-1 detection |
| PRRSV-EU-R | CAGCGTATGCTCGGTTACCA |  |
| PRRSV-EU-P | TACATTCTGGCCCCTGCCCA |  |
| ORF5-705F | GAAAACGCCAAAAGCACCTT | ORF5 amplification |
| ORF5-705R | GTTTTAGCCTGTCTTTTTGCC |  |

**TABLE S3** Distribution of the 441 study-derived ORF5 sequences across production units and years

| **Production unit** | **Jan–Dec 2023** | **Jan–Dec 2024** | **Jan–Dec 2025** | **Jan–May 2026** | **Total** |
| --- | --- | --- | --- | --- | --- |
| CF-1 | 4 | 1 | 9 | 1 | 15 |
| CF-2 | 2 | 0 | 7 | 1 | 10 |
| CF-3 | 1 | 0 | 7 | 1 | 9 |
| CF-4 | 3 | 0 | 8 | 9 | 20 |
| CF-5 | 1 | 3 | 7 | 0 | 11 |
| CF-6 | 2 | 3 | 8 | 8 | 21 |
| CF-7 | 3 | 1 | 22 | 24 | 50 |
| CF-8 | 2 | 0 | 15 | 3 | 20 |
| CF-9 | 3 | 1 | 11 | 3 | 18 |
| CF-10 | 2 | 0 | 6 | 5 | 13 |
| CF-11 | 1 | 1 | 17 | 0 | 19 |
| CF-12 | 3 | 1 | 5 | 15 | 24 |
| CF-13 | 6 | 1 | 8 | 0 | 15 |
| CF-15 | 4 | 1 | 14 | 8 | 27 |
| CF-16 | 0 | 0 | 3 | 4 | 7 |
| CF-17 | 1 | 0 | 9 | 0 | 10 |
| CF-18 | 1 | 0 | 11 | 0 | 12 |
| CF-19 | 3 | 1 | 3 | 1 | 8 |
| CF-20 | 1 | 0 | 12 | 15 | 28 |
| CF-21 | 3 | 1 | 9 | 0 | 13 |
| CF-22 | 1 | 0 | 7 | 6 | 14 |
| CF-23 | 4 | 1 | 10 | 2 | 17 |
| CF-24 | 2 | 0 | 7 | 10 | 19 |
| CF-25 | 3 | 2 | 14 | 4 | 23 |
| CF-26 | 2 | 0 | 6 | 0 | 8 |
| CF-Guancang | 0 | 0 | 3 | 3 | 6 |
| Yigong | 0 | 0 | 0 | 1 | 1 |
| Ergong | 0 | 2 | 0 | 0 | 2 |
| Sangong | 1 | 0 | 0 | 0 | 1 |
| Total | 59 | 20 | 238 | 124 | 441 |

**TABLE S4** Epidemiological metadata of the 11 PRRSV genomes successfully recovered by whole-genome sequencing

| **Strain** | **GenBank accession no.** | **Production unit** | **Production stage** | **Sampling date** | **Sample type** | **Sampling reason** |
| --- | --- | --- | --- | --- | --- | --- |
| CF-k141-31 | PZ851369 | CF-7 | Gestating sow | 27 Aug 2025 | Tail-root blood | Abortion |
| CF-k141-42 | PZ851370 | CF-8 | Nursery pig | 18 Sep 2025 | Serum | Screening |
| CF-k141-118 | PZ851371 | CF-7 | Gestating sow | 4 Oct 2025 | Umbilical cord blood | Abortion |
| CF-k141-560 | PZ851372 | CF-25 | Gestating sow | 28 Sep 2025 | Umbilical cord blood | Abortion |
| CF-k141-611 | PZ851373 | CF-15 | Gestating sow | 28 Sep 2025 | Umbilical cord blood | Abortion |
| CF-k141-616 | PZ851374 | CF-15 | Farrowing sow | 2 Oct 2025 | Placenta | Screening |
| CF-k141-1211 | PZ851375 | CF-25 | Farrowing sow | 18 Sep 2025 | Placenta | Screening |
| CF-k141-1388 | PZ851376 | CF-17 | Nursery pig | 12 Oct 2025 | Oronasal swab | Screening |
| CF-k141-2122 | PZ851377 | CF-17 | Nursery pig | 12 Oct 2025 | Oronasal swab | Screening |
| CF-k141-2190 | PZ851378 | CF-18 | Nursery pig | 11 Oct 2025 | Tissue | Screening |
| CF-k141-10114 | PZ851379 | CF-25 | Gilt | 15 Oct 2025 | Tissue | Death |

Note: A total of 21 PRRSV-positive samples were subjected to whole-genome sequencing. The 11 strains listed here represent samples for which complete or near-complete PRRSV genome assemblies of sufficient quality were successfully recovered and used for downstream analyses.

**TABLE S5** Production-unit-specific PRRSV RT-qPCR detection results from January 2023 to May 2026

| **Production unit** | **Number of positive samples** | **Number of tests** | **Positivity rate** |
| --- | --- | --- | --- |
| CF-1 | 2,548 | 9,902 | 25.73% |
| CF-2 | 361 | 532 | 67.86% |
| CF-3 | 941 | 15,432 | 6.10% |
| CF-4 | 305 | 372 | 81.99% |
| CF-5 | 1,447 | 12,327 | 11.74% |
| CF-6 | 384 | 503 | 76.34% |
| CF-7 | 3,535 | 43,098 | 8.20% |
| CF-8 | 5,441 | 13,671 | 39.80% |
| CF-9 | 1,438 | 16,900 | 8.51% |
| CF-10 | 706 | 833 | 84.75% |
| CF-11 | 926 | 11,087 | 8.35% |
| CF-12 | 386 | 483 | 79.92% |
| CF-13 | 1,500 | 12,697 | 11.81% |
| CF-15 | 2,577 | 16,182 | 15.93% |
| CF-16 | 2,746 | 5,121 | 53.62% |
| CF-17 | 991 | 14,079 | 7.04% |
| CF-18 | 752 | 1,085 | 69.31% |
| CF-19 | 1,547 | 10,262 | 15.08% |
| CF-20 | 230 | 254 | 90.55% |
| CF-21 | 1,333 | 14,398 | 9.26% |
| CF-22 | 212 | 742 | 28.57% |
| CF-23 | 691 | 11,007 | 6.28% |
| CF-24 | 210 | 286 | 73.43% |
| CF-25 | 3,811 | 30,346 | 12.56% |
| CF-26 | 1,144 | 7,080 | 16.16% |
| CF-Guancang | 99 | 103,647 | 0.10% |
| Yigong | 174 | 10,220 | 1.70% |
| Ergong | 18 | 8,177 | 0.22% |
| Sangong | 21 | 9,327 | 0.23% |
| Overall | 36,474 | 380,050 | 9.60% |

**TABLE S6** Contemporaneous detection of multiple ORF5-defined PRRSV lineages/sublineages within individual production units

| **Production unit** | **Sampling date** | **Lineages/sublineages detected** | **Sample source** |
| --- | --- | --- | --- |
| CF-2 | 20 Jun 2023 | Sublineage 1.5; Lineage 5 | Nursery pig (Sublineage 1.5); Finishing pig (Lineage 5) |
| CF-2 | 20 May 2025 | Sublineage 1.8; Lineage 8 | Finishing pig |
| CF-2 | 12 Aug 2025 | Sublineage 1.5; Lineage 5 | Nursery pig |
| CF-4 | 20 Jun 2023 | Sublineage 1.8; Lineage 8 | Nursery pig (Sublineage 1.8); Finishing pig (Lineage 8) |
| CF-4 | 9 Aug 2025 | Sublineage 1.8; Lineage 5 | Nursery pig |
| CF-4 | 20 Jan 2026 | Sublineage 1.8; Sublineage 1.5 | NA |
| CF-6 | 24 Jan 2026 | Sublineage 1.8; Sublineage 1.5 | NA |
| CF-7 | 30 Jun 2025 | Sublineage 1.5; Lineage 8 | Farrowing sow |
| CF-7 | 30 Jul 2025 | Sublineage 1.8; Sublineage 1.5; Lineage 8 | Farrowing sow |
| CF-7 | 20 Jan 2026 | Sublineage 1.8; Sublineage 1.5 | NA |
| CF-7 | 28 Jan 2026 | Sublineage 1.8; Lineage 5 | NA |
| CF-8 | 29 Jan 2026 | Sublineage 1.8; Sublineage 1.5 | NA |
| CF-9 | 30 Apr 2025 | Sublineage 1.8; Sublineage 1.5 | Farrowing sow |
| CF-9 | 29 Jan 2026 | Sublineage 1.8; Other/unassigned | NA |
| CF-10 | 21 Aug 2025 | Sublineage 1.8; Sublineage 1.5 | Nursery pig |
| CF-11 | 17 Sep 2025 | Sublineage 1.8; Sublineage 1.5 | Farrowing sow |
| CF-12 | 19 Sep 2023 | Sublineage 1.5; Lineage 5 | Nursery pig |
| CF-12 | 9 Aug 2025 | Sublineage 1.8; Sublineage 1.5 | NA |
| CF-12 | 6 Jan 2026 | Sublineage 1.8; Sublineage 1.5 | Nursery pig |
| CF-12 | 20 Jan 2026 | Sublineage 1.8; Sublineage 1.5 | NA |
| CF-12 | 29 Jan 2026 | Sublineage 1.8; Sublineage 1.5 | NA |
| CF-15 | 20 Jun 2023 | Sublineage 1.5; Lineage 8 | Nursery pig |
| CF-15 | 20 Jan 2026 | Sublineage 1.8; Sublineage 1.5 | NA |
| CF-16 | 29 Jan 2026 | Sublineage 1.5; Lineage 5 | NA |
| CF-20 | 1 Aug 2025 | Sublineage 1.8; Sublineage 1.5; Lineage 5 | Finishing pig tissue (Sublineage 1.8, Sublineage 1.5); Nursery pig tissue (Lineage 5) |
| CF-20 | 29 Jan 2026 | Sublineage 1.8; Sublineage 1.5; Other/unassigned | NA |
| CF-20 | 30 Jan 2026 | Sublineage 1.8; Sublineage 1.5 | NA |
| CF-22 | 20 Jan 2026 | Sublineage 1.8; Sublineage 1.5 | NA |
| CF-24 | 20 Jun 2023 | Sublineage 1.5; Lineage 8 | Finishing pig |
| CF-24 | 21 Jul 2025 | Sublineage 1.8; Lineage 8 | Nursery pig |
| CF-24 | 29 Jan 2026 | Sublineage 1.8; Lineage 5 | NA |
| CF-25 | 20 Jan 2026 | Sublineage 1.8; Sublineage 1.5 | NA |
| CF-26 | 22 Jun 2023 | Sublineage 1.5; Lineage 5 | Nursery pig |

Note: Contemporaneous detection was defined as the identification of ORF5 sequences assigned to two or more different PRRSV lineages/sublineages from samples collected within the same production unit on the same sampling date. NA: not available.

**TABLE S7** ORF5 nucleotide and GP5 amino acid identities between lineage 5/lineage 8 field strains and representative PRRSV vaccine-related strains

| **Field group** | **Number of sequences** | **Vaccine/reference strain** | **ORF5 nucleotide identity range** |
| --- | --- | --- | --- |
| Lineage 5 | 29 | VR-2332 | 97.4%–99.7% |
|  |  | RespPRRS MLV | 97.4%–100.0% |
| Lineage 8 | 17 | CH-1R | 92.7%–94.4% |
|  |  | JXA1-R | 97.5%–100.0% |

**TABLE S8** Pairwise ORF5 nucleotide identities within the major PRRSV-2 lineages/sublineages identified in this study

| **Lineage/sublineage** | **Number of sequences** | **Number of pairwise comparisons** | **ORF5 nucleotide identity range (%)** |
| --- | --- | --- | --- |
| Sublineage 1.8 | 237 | 27,966 | 83.3%–100.0% |
| Sublineage 1.5 | 153 | 11,628 | 89.1%–100.0% |
| Lineage 5 | 29 | 406 | 96.4%–100.0% |
| Lineage 8 | 17 | 136 | 97.5%–100.0% |

**TABLE S9** Short-term longitudinal whole-genome comparisons of PRRSV strains obtained from the same production units at different sampling times

| **Production unit** | **Earlier strain** | **Sampling date** | **Later strain** | **Sampling date** | **Interval (days)** | **Whole-genome nucleotide identity** |
| --- | --- | --- | --- | --- | --- | --- |
| CF-7 | CF-k141-31 | 27 Aug 2025 | CF-k141-118 | 4 Oct 2025 | 38 | 89.6% |
| CF-15 | CF-k141-611 | 28 Sep 2025 | CF-k141-616 | 2 Oct 2025 | 4 | 89.9% |
| CF-25 | CF-k141-1211 | 18 Sep 2025 | CF-k141-560 | 28 Sep 2025 | 10 | 90.2% |
| CF-25 | CF-k141-560 | 28 Sep 2025 | CF-k141-10114 | 15 Oct 2025 | 17 | 90.1% |
| CF-25 | CF-k141-1211 | 18 Sep 2025 | CF-k141-10114 | 15 Oct 2025 | 27 | 100.0% |

Note: Whole-genome nucleotide identities were derived from pairwise comparisons of the 11 successfully assembled study-derived genomes. Serial samples were collected at the production-unit level and were not necessarily obtained from the same individual animals. Therefore, these comparisons reflect short-term changes in the viral populations detected within production units rather than direct within-host evolutionary trajectories.

**TABLE S10** Pairwise whole-genome nucleotide identities among the 11 PRRSV strains obtained in this study

| **Strain** | **CF-k141-31** | **CF-k141-42** | **CF-k141-118** | **CF-k141-560** | **CF-k141-611** | **CF-k141-616** | **CF-k141-1211** | **CF-k141-1388** | **CF-k141-2122** | **CF-k141-2190** | **CF-k141-10114** |
| --- | --- | --- | --- | --- | --- | --- | --- | --- | --- | --- | --- |
| **CF-k141-31** | — | 90.9% | 89.6% | 88.2% | 88.2% | 89.7% | 90.8% | 90.8% | 87.4% | 90.6% | 90.7% |
| **CF-k141-42** | 90.9% | — | 89.7% | 90.3% | 90.3% | 89.8% | 98.7% | 98.8% | 88.1% | 92.5% | 98.7% |
| **CF-k141-118** | 89.6% | 89.7% | — | 90.1% | 89.9% | 99.4% | 89.6% | 89.3% | 91.4% | 89.3% | 89.5% |
| **CF-k141-560** | 88.2% | 90.3% | 90.1% | — | 98.5% | 89.9% | 90.2% | 89.9% | 88.3% | 92.6% | 90.1% |
| **CF-k141-611** | 88.2% | 90.3% | 89.9% | 98.5% | — | 89.9% | 90.2% | 89.8% | 87.3% | 90.0% | 90.0% |
| **CF-k141-616** | 89.7% | 89.8% | 99.4% | 89.9% | 89.9% | — | 89.7% | 89.5% | 91.5% | 89.5% | 89.6% |
| **CF-k141-1211** | 90.8% | 98.7% | 89.6% | 90.2% | 90.2% | 89.7% | — | 99.6% | 88.0% | 92.3% | 100% |
| **CF-k141-1388** | 90.8% | 98.8% | 89.3% | 89.9% | 89.8% | 89.5% | 99.6% | — | 88.0% | 92.3% | 99.6% |
| **CF-k141-2122** | 87.4% | 88.1% | 91.4% | 88.3% | 87.3% | 91.5% | 88.0% | 88.0% | — | 87.6% | 88.0% |
| **CF-k141-2190** | 90.6% | 92.5% | 89.3% | 92.6% | 90.0% | 89.5% | 92.3% | 92.3% | 87.6% | — | 92.3% |
| **CF-k141-10114** | 90.7% | 98.7% | 89.5% | 90.1% | 90.0% | 89.6% | 100% | 99.6% | 88.0% | 92.3% | — |

Note: Diagonal self-comparisons are indicated by an em dash.

**TABLE S11** Whole-genome nucleotide identities between the 11 study-derived PRRSV strains and representative PRRSV-2 reference strains

| **Strain** | **Sublineage 1.8** | | | **Sublineage 1.5** | **Lineage 3** | **Lineage 5** | | **Lineage 8** | | | |
| --- | --- | --- | --- | --- | --- | --- | --- | --- | --- | --- | --- |
|  | **JL580** | **LNCH-1604** | **NADC30** | **NADC34** | **QYYZ** | **VR-2332** | **RespPRRS MLV** | **CH-1a** | **JXA1** | **JXA1-R** | **TJ** |
| **CF-k141-31** | 87.8% | 87.4% | 87.4% | 85.1% | 81.2% | 83.5% | 83.5% | 83.7% | 83.5% | 83.7% | 83.7% |
| **CF-k141-42** | 88.1% | 87.8% | 87.8% | 85.0% | 82.0% | 84.2% | 84.3% | 84.4% | 84.3% | 84.5% | 84.5% |
| **CF-k141-118** | 87.1% | 86.7% | 86.7% | 85.6% | 82.5% | 84.4% | 84.6% | 85.0% | 85.2% | 85.5% | 85.5% |
| **CF-k141-560** | 87.9% | 87.9% | 88.2% | 84.1% | 82.2% | 84.1% | 84.2% | 84.5% | 84.6% | 84.7% | 84.7% |
| **CF-k141-611** | 87.7% | 87.7% | 88.0% | 83.9% | 82.0% | 83.9% | 84.0% | 84.3% | 84.4% | 84.5% | 84.5% |
| **CF-k141-616** | 87.2% | 86.8% | 86.8% | 85.7% | 82.7% | 84.6% | 84.7% | 85.2% | 85.3% | 85.6% | 85.6% |
| **CF-k141-1211** | 88.0% | 87.7% | 87.7% | 85.0% | 81.9% | 84.2% | 84.2% | 84.3% | 84.2% | 84.5% | 84.4% |
| **CF-k141-1388** | 88.0% | 87.6% | 87.6% | 85.0% | 81.9% | 84.1% | 84.1% | 84.3% | 84.2% | 84.4% | 84.4% |
| **CF-k141-2122** | 86.8% | 87.8% | 87.7% | 85.1% | 82.7% | 84.6% | 84.6% | 84.9% | 84.6% | 85.0% | 85.0% |
| **CF-k141-2190** | 88.2% | 87.8% | 88.1% | 84.8% | 82.0% | 84.0% | 84.0% | 84.0% | 83.8% | 84.0% | 83.9% |
| **CF-k141-10114** | 88.0% | 87.7% | 87.7% | 85.0% | 82.0% | 84.2% | 84.2% | 84.3% | 84.3% | 84.5% | 84.5% |

**TABLE S12** Recombination events detection and analysis of the eleven PRRSV strains

| Recombinant | Major parent | Minor parent | Breakpoint position in alignment | Methods (Av. *P*-Val) | | | | | | | Recombinant score |
| --- | --- | --- | --- | --- | --- | --- | --- | --- | --- | --- | --- |
|  |  |  |  | RDP | GENECONV | BootScan | MaxChi | Chimaera | SiScan | 3Seq |  |
| CF-k141-31 | NADC30 | JXA1-R | 5,220-7,970 | 4.920×10^-68^ | 2.017×10^-15^ | 2.667×10^-68^ | 8.462×10^-25^ | 1.229×10^-28^ | 4.120×10^-30^ | 1.217×10^-12^ | 0.408 |
| CF-k141-560 | LNCH-1604 | XJ17-5 | 1,143-1,655 | 2.458×10^-26^ | 4.601×10^-07^ | 4.935×10^-23^ | 1.815×10^-10^ | 4.664×10^-12^ | 5.856×10^-13^ | 1.217×10^-12^ | 0.694 |
| CF-k141-2190 | NADC30 | TJ | 5,220-6,503 | 1.397×10^-28^ | 1.766×10^-10^ | 1.677×10^-27^ | 5.599×10^-13^ | 2.114×10^-09^ | 6.611×10^-16^ | 8.114×10^-13^ | 0.681 |
