# Supplementary material for: Longitudinal Molecular Epidemiology and Genomic Evolution of Porcine Reproductive and Respiratory Syndrome Virus in a Large‐Scale Swine Production System in Northern China, 2023–2026
Source: Transbound Emerg Dis. 2026 Sep 22;2026:6131183. doi: 10.1155/tbed/6131183 (PMC13594771; doi:10.1155/tbed/6131183)
Supplement: Supplementary file 1 [file TBED-2026-6131183-s001.zip › Supplementary Data S1.docx]

>CF-1chanfangzhuzuzhi20230619

ATGTTGGGGAGATGCTTGACCGCGGGCTGTTGCTCGCAATTGCCTTTTTTGTGGTATATCGTGCCGTTCTGTTTTGTTGTGCTCGTCAACGCCAACAACAGCAGCAGCTCCCATTTACAGTTGATTTATAACCTGACGATATGTGAGCTGAATGGCACAGATTGGCTAAATAAAAGTTTTGATTGGGCAGTGGAGACCTTTGTTATTTTCCCTGTGTTGACTCATATTGTCTCCTATGGCGCCCTAACCACCAGCCATTTCCTTGACACAGTTGGCCTGATCACCGTGTCTACCGCTGGGTATTATCACGGGCGGTATGTCCTGAGCAGCATTTACGCCGTCTGCGCCCTAGCTGCGTTAACTTGCTTTGTCATCAGGCTAACAAAAAATTGCATGTCCTGGCGTTACTCATGCACCAGATACACTAACTTTCTTCTGGACACCAAGGGCAAACTCTATCGTTGGCGGTCTCCCGTCATCGTGGAGAAAGGGGGTAAAGTTGAGGTCGCAGGCCACCTGATCGACCTCAAGAGAGTTGTGCTTGACGGTTCCGCGGCGACCCCTATAACCAAAGTTTCAGCGGAACAATGGGGTCGTCCTTAG

>CF-2baoyuzhuzuzhi20230620

ATGTTGGGGAGATGCTTGACCGCGGGCTGTTGCTCGCAATTGCCTTTTTTGTGGTATATCGTGCCGTTCTGTTTTGTTGTGCTCGTCAACGCCAACAACAGCAGCAGCTCCCATTTACAGTTGATTTATAACCTGACGATATGTGAGCTGAATGGCACAGATTGGCTAAATAAAAGTTTTGATTGGGCAGTGGAGACCTTTGTTATTTTCCCTGTGTTGACTCATATTGTCTCCTATGGCGCCCTAACCACCAGCCATTTCCTTGACACAGTTGGCCTGATCRCCGTGTCTACCGCTGGGTATTATCACGGGCGGTATGTCCTGAGCAGCATTTACGCCGTCTGCGCCCTAGCTGCGTTAACTTGCTTTGTCATCAGGCTAACAAAAAATTGCATGTCCTGGCGTTACTCATGCACCAGATACACTAACTTTCTTCTGGACACCAAGGGCAAACTCTATCGTTGGCGGTCTCCCGTCATCGTGGAGAAAGGGGGTAAAGTTGAGGTCGAAGGCCACCTGATCGACCTCAAGAGAGTTGTGCTTGACGGTTCCGCGGCGACCCCTATAACCAAAGTTTCAGCGGAACAATGGGGTCGTCCTTAG

>CF-2yufeizhuzuzhi20230620

ATGTTGGGGAGATGCTTGATCGCGGGCTGCTGCTCGCAATTGCTTTTTTTGTGGTGTATCGTGCCGTTCTGTTTTGTTGCGCTCGTCAACGCCAACAACAGCAGCAGCTCCCATCTACAGCTGATTTACAACTTGACGCTATGTGAGCTGAACGGCACAGATTGGCTAGCTGACAAATTTGATTGGGCAGTGGAGAGTTTTGTCATCTTTCCCGTTTTGACTCACATTGTCTCCTATGGTGCCCTCACTACCAGCCATTTCCTTGACACAGTCGCTTTAGTCACTGTGTCTACCGCCGGGTTTGTTCACGGGCGGTATGTCCTAAGTAGCATCTACGCGGTCTGTGCCCTGGCTGCGTTGACTTGCTTCGTCATTAGGTTTGCAAAGAATTGCATGTCCTGGCGCTATGCGTGTACCAGATATACCAACTTTCTTCTGGACACTAAGGGCAGACTCTATCGTTGGCGGTCGCCTGTCATCATAGAGAAAATGGGCAAAGTTGAGGTCGAAGGTCATCTGATCGACCTCAAAAGAGTTGTGCTTGATGGTTCCGTGGCAACCCCTATAACCAGAGTTTCAGCGGAACAATGGGGTCGTCCTTAG

>CF-3chanfangzhuzuzhi20230628

ATGTTGGGGAGATGCTTGACCGCGGGCTGTTGCTCGCAATTGCCTTTTTTGTGGTATATCGTGCCGTTCTGTTTTGTTGTGCTCGCCAACGCCAACAACAGCAGCAGCTCCCATTTACAGTTGATTTATAACCTGACGATATGTGAGCTGAACGGCACAAATTGGCTAAATGAAAGTTTTGATTGGGCAGTGGAGACCTTTGTTATTTTCCCTGTGTTGACTCATATTGTCTCCTATGGCGCCCTAACCACCAGCCATTTCCTTGACACAGTTGGCCTGATCACCGTGTCTACCGCTGGATATTATCACGGGCGGTATGTCCTGAGCAGCATTTACGCCGTCTGCGCCCTAGCTGCGTTAACTTGCTTTGTCATCAGGCTAACAAAAAATTGCATGTCCTGGCGTTACTCATGCACCAGATACACTAACTTTCTTCTGGACACCAAGGGCAAACTCTATCGTTGGCGGTCTCCCGTCATCGTGGAGAAAGGGGGTAAAGTTGAGGTCGAAGGCCACCTGATCGACCTCAAGAGAGTTGTGCTTGACGGTTCCGCGGCGACCCCTATAACCAAAATTTCAGCGGAACAATGGGGTCGTCCTTAG

>CF-4baoyuzhuzuzhi20230620

ATGTTGGGGAAATGCTTGACCGCGGGTTATTGCTCACAATCGCTTTTTTTGTGGTGTATCGTGCCATTCTGTTTTGCTGTGCTCGTCAACGCCAGCGACAACAGCAGCTCCCACTTACAGTTGATCTATAACCTGACGATATGTGAGCTGAATGGCACAGACTGGTTGAACCAAAAATTTGACTGGGCAGTAGAGACTTTCGTCATTTTTCCTGTCTTGACCCATATTGTCTCTTACGGCGCCCTTACCACTAGCCATTTTCTTGACACGGTCGGCCTAATCACCGTATCCACCGCTGGGTATTATCATAAGCGGTATGTATTGAGTAGCATCTACGCTGTCTGTGCCCTGGCCGCGTTGGTTTGCTTCGCCATTAGGTTGGCAAAAAATTGTATGTCCTGGCGCTACTCATTCACCAGATATACTAATTTTCTTCTGGACTCTAAGGGCAAACTCTACCGTTGGCGGTCACCCGTCATCATAGAGAAAGAGGGCAAAGTTGATGTGGGGGGTCAGTTAATCGACCTCAAGAGAGTTGTGCTTGATGGTTCCGCGGCGACCCCTGTAACCAAGATTTCAGCGGAACAATGGGGTCGTCCATAG

>CF-4yufeizhuzuzhi20230620

ATGTTGGGGAAGTGCTTGACCGCGTGCTGTTGCTCGCGATTGCTTTTTTTGTGGTGTATCGTGCCGTCCTATCTTGCTGTGCTCGTCAACGCCAGCAACAACTACAGCTCTCATATTCAGTTGATTTATAACTTAACGCTATGTGAGCTGAATGGCACAGATTGGCTGGCACAAAAATTTGACTGGGCAGTGGAGACTTTTGTCATCTTCCCCGTGTTGACTCACATTGTTTCCTATGGGGCACTCACCACCAGCCATTTCCTTGACACAGTTGGTCTGGCCACTGTGTCCACCGCCGGATATTATCACGGGCGGTATGTCTTGAGTAGCATTTACGCAGTCTGTGCTCTGGCTGCGCTGATTTGCTTTGTCATTAGGCTTGCGAAGAACTGCATGTCCTGGCGCTACTCTTGTACCAGATATACCAACTTCCTTCTGGACACTAAGGGCAAACTCTATCGTTGGCGGTCGCCCGTCATTGTGGAGAAAGGGGGTAAGGTTGAGGTCGAAGGTCACCTGATCGACCTCAAGAGAGTTGTGCTTGATGGTTCCGCGGCAACCCCTTTAACCAGAGTTTCAGCGGAACGATGGGGTCGTCTCTAG

>CF-7chanfangzizhuzuzhiye20230721

ATGTTGGAGAAATGCTTGACCGCGGGCTGTTGCTCGCAATTGCTTTCTTTGTGGTGTATCGTGCCGTTCTGTTTTGCTGTGCTCGCCAACGCCAGCAACGACAGCAGCTCCCATCTACAGCTGATTTACAACTTGACGCTATGTGAGCTGAATGGCACAGATTGGCTAGCTAACAAATTTGATTGGGCAGTGGAGAGTTTTGTCATCTTTCCCGTTTTGACTCACATTGTCTCCTATGGTGCCCTCACTACCAGCCATTTCCTTGACACAGTCGCTTTAGTCACTGTGTCTACCGCCGGGTTTGTTCACGGGCGGTATGTCCTAAGTAGCATCTACGCGGTCTGTGCCCTGGCTGCGTTGACTTGCTTCGTCATTAGGTTTGCAAAGAATTGCATGTCCTGGCGCTACGCGTGTACCAGATATACCAACTTTCTTCTGGACACTAAGGGCGGACTCTATCGTTGGCGGTCGCCTGTCATCATAGAGAAAAGGGGCAAAGTTGAGGTCGAAGGTCATCTGATCGACCTCAAAAGAGTTGTGCTTGATGGTTCCGTGGCAACCCCTATAACCAGAGTTTCAGCGGAACAATGGGGTCGTCCTTAG

>CF-8baoyuzhuzuzhi20230620

ATGTTGGGGAAATGCTTGACCGCGGGTTATTGCTCACAATCGCTTTTTTTGTGGTGTATCGTGCCATTCTGTTTTGCTGTGCTCGTCAACGCCAGCGGCAACAGCAGCTCCCACTTACAGTTGATCTATAACCTGACGATATGTGAGCTGAATGGCACAGACTGGTTGAACCAAAAATTTGACTGGGCAGTAGAGACTTTCGTCATTTTTCCTGTCTTGACCCATATTGTCTCTTACGGCGCCCTTACCACTAGCCATTTTCTTGACACGGTCGGCCTAATCACCGTATCCACCGCTGGGTATTATCATAAGCGGTATGTATTGAGTAGCATCTACGCTGTCTGTGCCCTGGCCGCGTTGGTTTGCTTCGCCATTAGGTTGGCAAAAAATTGTATGTCCTGGCGCTACTCATTCACCAGATATACGAATTTTCTTCTGGACTCTAAGGGCAAACTCTACCGTTGGCGGTCACCCGTCATCATAGAGAAAGAGGGCAAAGTTGATGTGGGGGGTCAGTTAATCGACCTCAAGAGAGTTGTGCTTGATGGTTCCGCGGCGACCCCTGTAACCAAGATTTCAGCGGAACAATGGGGTCGTCCATAG

>CF-9chanfangzizhuzuzhi20230704

ATGTTGGGGAAATGCTTGACCGCGGGCTACTGCTCGCAATTGCTTTTTTTGTGGTGTATCGTGCCGTCCTGTTTAGTTGCGCTCGTCAACGCTCAAAGAAACAGCAGCTCCCATTTACAGTTGATTTATAACCTGACGATATGTGAGCTGAATGGTACAGATTGGCTAAATGAAACATTTGATTGGGCGGTGGAGACCTTTGTTATTTTTCCTGTGCTGACTCATATTGTCTCCTATGGCGCCCTAACCACCAGCCATTTCCTTGACACAGTCGGCCTGATCACCGTGTCTGCCGCCGGATATTGCCACGAGCGGTATGTTTTGAGCAGCATTTACGCCGTCTGCGCCCTAGCTGCGTTAACTTGCTTCGTCATCAGGCTAACAAAAAATTGCATGTCCTGGCGTTACTCATGCACCAGATACACTAACTTTCTTCTGGACACCAAGGGCAAACTCTATCGTTGGCGGTCTCCTGTCATCGTAGAGAAAGGGGGTAAAGTCGAGGTCGGAGGCCACCTGATCGACCTCAAGAGGGTTGTGCTTGACGGTTCCGCGGCGACCCCTATAACCAAAGTTTCAGCGGAACAATGGGGTCGTCCTTAG

>CF-9chanfangzizhuzuzhi20230704(2)

ATGTTGGAGAAATGCTTGACCGCGGGCTGTTGCTCGCAATTGCTTTCTTTGTGGTGTATCGTGCCGTTCTGTTTTGCTGTGCTCGCCAACGCCAGCAACGACAGCAGCTCCCATCTACAGCTGATTTACAACTTGACGCTATGTGAGCTGAATGGCACAGATTGGCTAGCTAACAAATTTGATTGGGCAGTGGAGAGTTTTGTCATCTTTCCCGTTTTGACTCACATTGTCTCCTATGGTGCCCTCACTACCAGCCATTTCCTTGACACAGTCGCTTTAGTCACTGTGTCTACCGCCGGGTTTGTTCACGGGCGGTATGTCCTAAGTAGCATCTACGCGGTCTGTGCCCTGGCTGCGTTGACTTGCTTCGTCATTAGGTTTGCAAAGAATTGCATGTCCTGGCGCTACGCGTGTACCAGATATACCAACTTTCTTCTGGACACTAAGGGCGGACTCTATCGTTGGCGGTCGCCTGTCATCATAGAGAAAAGGGGCAAAGTTGAGGTCGAAGGTCATCTGATCGACCTCAAAAGAGTTGTGCTTGATGGTTCCGTGGCAACCCCTATAACCAGAGTTTCAGCGGAACAATGGGGTCGTCCTTAG

>CF-10baoyuzhuzuzhi20230620

ATGTTGGGGAAATGCTTGACCGCGGGCTACTGCTCGCAATTGCTTTTTTTGTGGTGTATCGTGCCGTCCTGTTTAGTTGCGCTCGTCAACGCTCAAAGAAACAGCAGCTCCCATTTACAGTTGATTTATAACCTGACGATATGTGAGCTGAATGGCACAGATTGGCTAAATGAAACATTTGATTGGGCGGTGGAGACCTTTGTTATTTTTCCTGTGCTGACTCATATTGTCTCCTATGGCGCCCTAACCACCAGCCATTTCCTTGACACAGTCGGCCTGATCACCGTGTCTGCCGCCGGATATTGCCACGGGCGGTATGTTTTGAGCAGCATTTACGCCGTCTGCGCCCTAGCTGCGTTAACTTGCTTCGTCATCAGGCTAACAAAAAATTGCATGTCCTGGCGTTACTCATGCACCAGATACACTAACTTTCTTCTGGACACCAAGGGCAAACTCTATCGTTGGCGGTCTCCTGTCATCGTAGAGAAAGGGGGTAAAGTCGAGGTCGGAGGCCACCTGATCGACCTCAAGAGGGTTGTGCTTGACGGTTCCGCGGCGACCCCTATAACCAAAGTTTCAGCGGAACAATGGGGTCGTCCTTAG

>CF-11chanfangzizhuzuzhiye20230619

ATGTTGGTGAAATGCTTGACCGCGGGCTACTGCTCGCAATTGCTTTTTTTGTGGTGTATCGTGCCGTTCTGTTTAGTTGCGCTCGTCAACGCTCAAAAAAACAGCAGCTCCCATTTACAGTTGATTTATAACCTGACGATATGTGAGCTGAATGGCACAGATTGGCTAAATGAAACATTTGATTGGGCGGTGGAGACCTTTGTTATTTTTCCTGTGCTGACTCATATTGTCTCCTATGGCGCCCTAACCACCAGCCATTTCCTTGACACAGTCGGCCTGATCGCCGTGTCTGCCGCCGGATATTACCACGGGCGGTATGTTTTGAGCAGCATTTACGCCGTCTGCGCCCTAGCTGCGTTAACTTGCTTCGTCATCAGGCTAACAAAAAATTGCATGTCCTGGCGTTATTCATGCACCAGATACACTAACTTTCTTCTGGACACCAAGGGCAAACTCTATCGTTGGCGGTCTCCTGTCATCGTAGAGAAAGGGGGTAAAGTCGAGGTCGGAGGCCACCTGATCGACCTCAAGAGGGTTGTGCTTGACGGTTCCGCGGCGACCCCTATAACCAAAGTTTCAGCGGAACAATGGGGTCGTCCTTAG

>CF-12baoyuzhuzuzhi20230620

ATGTTGGTGAAATGCTTGACCGCGGGCTACTGCTCGCAATTGCTTTTTTTGTGGTGTATCGTGCCGTTCTGTTTAGTTGCGCTCGTCAACGCTCAAAAAAACAGCAGCTCCCATTTACAGTTGATTTATAACCTGACGATATGTGAGCTGAATGGCACAGATTGGCTAAATGAAACATTTGATTGGGCGGTGGAGACCTTTGTTATTTTTCCTGYGCTGACTCATATTGTCTCCTATGGCGCCCTAACCACCAGCCATTTCCTTGACACAGTCGGCCTGATCGCCGTGTCTGCCGCCGGATATTACCACGGGCGGTATGTTTTGAGCAGCATTTACGCCGTCTGCGCCCTAGCTGCGTTAACTTGCTTCGTCATCAGGCTAACAAAAAATTGCATGTCCTGGCGTTACTCATGCACCAGATACACTAACTTTCTTCTGGACACCAAGGGCAAACTCTATCGTTGGCGGTCTCCTGTCATCGTAGAGAAAGGGGGTAAAGTCGAGGTCGGAGGCCACCTGATCGACCTCAAGAGGGTTGTGCTTGACGGTTCCGCGGCGACCCCTATAACCAAAGTTTCAGCGGAACAATGGGGTCGTCCTTAG

>CF-13chanfangzhuzuzhi20230710

ATGTTGGGGAAATGCTTGACCGCGGGCTGCTGCTCGCAATTGCTTTTTTTGTGGTGTATCGTGCCGTTCTGTTTAGTTTCGCTCGTCAACGCCAACAACAGCAGCAGCTCCCATTTACAGTTGATTTATAACCTGACGATATGTGAGCTGAATGGCACAGGTTGGCTAGATAGCAATTTTGATTGGGCAGTGGAGACCTTCGTTATTTTTCCTGTGTTGACTCATATCGTCTCCTATGGCGCCCTAACCACCAGCCACTTCCTTGACACAGTCGGCCTGATCACCGTGTCTACTGCTGGATATTGCCACGGGCGGTATGTCTTGAGCAGTATTTACGCCGTCTGCGCCCTAGCTGCACTAACTTGCTTCGTCATCAGGCTAACAAAAAATTGCATGTCCTGGCGTTACTCATGCACCAGATACACTAATTATCTTCTGGACACCAAGGGAAAACTCTATCGTTGGCGGTCTCCCGTCATCGTAGAAAAAGGGGGTAAAGTTGAGGTCGAAGGCCACCTCATCGACCTCAAGAAAGTTGTGCTTGACGGTTCCGCGGCGACCCCTATAACCAAAGTTTCAGCGGAACAATGGGGTCGTCCTTAG

>CF-13houbeishezhuzuzhi20230804

ATGTTGGGGAAATGCTTGACCGCGGGCTGCTGCTCGCAATTGCTTTTTTTGTGGTGTATCGTGCCGTTCTGTTTAGTTTCGCTCGTCAACGCCAACAACAGCAGCAGCTCCCATTTACAGTTGATTTATAACCTGACGATATGTGAGCTGACTGGCACAGATTGGCTAGGTAGCAATTTTGATTGGGCAGTGGAGACCTTCGTTATTTTTCCTGTGTTGACTCATATCGTCTCCTATGGCGCCCTAACCACCAGCCACTTCCTTGACACAGTCGGCCTGATCACCGTGTCTACTGCTGGATATTGCCGCGGGCGGTATGTCTTGAGCAGTATTTACGCCGTCTGCGCCCTAGCTGCGCTAACTTGCTTCGTCATCAGGCTAACAAAAAATTGCATGTCCTGGCGTTACTCATGCACCAGATACACTAATTTTCTTCTGGACACCAAGGGAAAACTCTATCGTTGGCGGTCTCCCGTCATCGTAGAAAAAGGGGGTAAAGTTGAGGTCGAAGGCCACCTCATCGACCTCAAGAAAGTTGTGCTTGACGGTTCCGCGGCGACCCCTATAACCAAAGTTTCAGCGGAACAATGGGGTCGTCCTTAG

>CF-14yiqubaoyuzhuzuzhi20230620

ATGTTGGGGAAATGCTTGACCGCGGGCTGCTGCTCGCAATTGCTTTTTTTGTGGTGTATCGTGCCGTTCTGTTTAGTTTCGCTCGTCAACGCCAACGACAGCAGCAGCTCCCATTTACAGTTGATTTATAACCTGACGATATGTGAGCTGAATGGCACAGATTGGCTAGATAGCAATTTTGATTGGGCAGTGGAGACCTTCGTTATTTTTCCTGTGTTGACTCATATCGTCTCCTATGGCGCCCTAACCACCAGCCACTTCCTTGACACAGTCGGCCTGATCACCGTGTCTACTGCTGGATATTGCCACGGGCGGTATGTCTTGAGCAGTATTTACGCCGTCTGCGCCCTAGCTGCGCTAATTTGCTTCGTCATCAGGCTAACAAAAAATTGCATGTCCTGGCGTTACTCATGCACCAGATACACTAATTTTCTTCTGGACACCAAGGGAAAACTCTATCGTTGGCGGTCTCCCGTCATCGTAGAAAAAGGGGGTAAAGTTGAGGTCGAAGGCCAYCTCATCGACCTCAAGAAAGTTGTGCTTGACGGTTCCGCGGCGACCCCTATAACCAAAGTTTCAGCGGAACAATGGGGTCGTCCTTAG

>CF-14erqubaoyuzhuzuzhi20230620

ATGTTGGGGAAGTGCTTGACCGCGTGCTGTTGCTCGCGATTGCTTTTTTTGTGGTGTATCGTGCCGTCCTATCTTGCTGTGCTCGTCAACGCCAGCAACAACAACAGCTCTCATATTCAGTTGATTTATAACTTAACGCTATGTGAGCTGAATGGCACAGATTGGCTGGCACAAAAATTTAACTGGGCAGTGGAGACTTTTGTCATCTTCCCCGTGTTGACTCACATTGTTTCCTATGCGGCACTCACCACCAGCCATTTCCTTGACACAGTTGGTCTGGCCACTGTGTCCACCGCCGGATATTATCACGGGCGGTATGTCTTGAGTAGCATTTACGCAGTCTGTGCTCTGGCTGCGCTGATTTGCTTTGTCATTAGGCTTGCGAAGAACTGCATGTCCTGGCGCTACTCTTGTACCAGATATACCAACTTCCTTCTGGACACTAAGGGCAAACTCTATCGTTGGCGGTCGCCCGTCATTGTGGAGAAAGGGGGTAAGGTTGAGGTCGAAGGTCACCTGATCGACCTCAAGAGAGTTGTGCTTGATGGTTCCGCGGCAACCCCTTTAACCAGAGTTTCAGCGGAACGATGGGGTCGTCTCTAG

>CF-14pici2023001zhuzuzhi20230804

ATGTTGGGGAAGTGCTTGACCGCGTGCTGTTGCTCGCGATTGCTTTTTTTGTGGTGTATCGTGCCGTCCTATCTTGCTGTGCTCGTCAACGCCAGCAACAACAACAGCTCTCATATTCAGTTGATTTATAACTTAACGCTATGTGAGCTGAATGGCACAGATTGGCTGGCACAAAAATTTGACTGGGCAGTGGAGACTTTTGTCATCTTCCCCGTGTTGACTCACATTGTTTCCTATGCGGCACTCACCACCAGCCATTTCCTTGACACAGTTGGTCTGGCCATTGTGTCCACCGCCGGATATTATCACGGGCGGTATGTCTTGAGCAGCATTTACGCAGTCTGTGCTCTGGCTGCGCTGATTTGCTTTGTCATTAGGCTTGCGAAGAACTGCATGTCCTGGCGCTACTCTTGTACCAGATATACCAACTTCCTTCTGGACACTAAGGGCAAACTCTATCGTTGGCGGTCGCCCGTCATTGTGGAGAAAGGGGGTAAGGTTGAGGTCGAAGGTCACCTGATCGACCTCAAGAGAGTTGTGCTTGATGGTTCCGCGGCAACCCCTTTAACCAGAGTTTCAGCGGAACGATGGGGTCGTCTCTAG

>CF-17nanquyufeizhuzuzhi20230721

ATGTTGGGGAAATGCTTGATCGCGGGCTGCTGCTCGCAATTGCCTTTTTTGTGGTGTATCGTGCCGTTCTGTTTTGTTGCGCTCGTCAACGCCAGCAACAGCAGCAGCTCCCATTTACAGTTGATTTATAACCTGACAATATGTGAGCTGAATGGCACAGATTGGCTGAATGAAAGATTTGATTGGGCGGTGGAGACCTTTGTTATTTTTCCTGTGTTGACTCACATTGTCTCCTATGGCGCCCTAACCACCAGCCATTTCCTTGACACAGTCGGCCTGATCACCGTGTCTACCGCCGGATATTATCACAGGCGGTATGTCTTGAGCAGCATCTACGCTGTCTGCGCCCTAGCTGCGCTAACTTGCTTCGTCATCAGGCTAACAAAAAATTGCATGTCCTGGCGTTACTCATGTACCAGATACACTAACTTTCTTCTGGACACCAAGGGCAAACTCTATCGTTGGCGGTCTCCCGTCATCGTAGAGAAAGGGGGTAAAGTTGAGGTCGAAGGCCATTTAATCGACCTCAAGAGAGTTGTGCTTGACGGTTCCGCGGCGACCCCTTTAACCAAAGTTTCAGCGGAACAATGGGGTCGTCCTTAG

>CF-18yufeizhuxueqing20230620

ATGTTGGGGAAATGCTTGACCGCGGGTTATTGCTCACAATTGCTTTTTTTGTGGTGTATCGTGCCGTTCTGTTTTGCTGTGCTCGTCAACGCCAGCGACAACAACAGCTCCCATTTACAGTTGATTTATAACCTGACGATATGTGAGCTGAATGGCACAGACTGGTTGAACCAAAAATTTGACTGGGCAGTAGAGACTTTCGTCATTTTTCCTGTGTTGACTCACATTGTCTCCTATGGCGCCCTAACCACCAGCCATTTCCTTGACACAGTCGGCCTGATCACCGTGTCTACCGCCGGATATTATCACGGGCGGTATGTCTTGAGCAGCATTTACGCTGTCTGCGCCCTAGCTGCGCTAACTTGCTTCGCCATTAGGTTGGCAAAAAATTGTATGTCCTGGCGCTACTCATTCACCAGATATACTAATTTTCTTCTGGACTCTAAGGGCAAACTCTACCGTTGGCGGTCACCCGTCATCATAGAAAAAGAGGGCAAAGTTGATGTGGGGGGTCAGTTAATTGACCTCAAGAGAGTTGTGCTTGATGGTTCCGCGGCAACCCCTGTAACCAAGATTTCAGCGGAACAATGGGGTCGTCCATAG

>CF-20yufeizhuzuzhi20230620

ATGTTGGGGAAATGCTTGACCGCGGGCTGCTGCTCGCAATTTCTTTTTTTGTGGTGTATCGTGCCGTTCTGTTTAGTTGCGCTCGTCAACGCCAACAACAGCAGCAGCTCCCACTTACAGTTGATTTATAACCTGACGATATGTGAGCTGAATGGCACAGATTGGCTGAATAAAAGTTTTGATTGGGCAGTGGAGACCTTTGTTATTTTTCCTGTGTTGACTCATATTGTCTCCTATGGTGCCCTAACCACCAGCCATTTCCTTGATACAGTCGGCCTGATCACCGTGTCTACCGCTGGATATTACCACGGGCGGTATGTCTTGAGCAGCATTTACGCCGTCTGCGCCCTAGCTGCATTAACTTGCTTCGTCATCAGGCTAACAAAAAATTGCATGTCCTGGCGTTACTCATGCACCAGATACACTAACTTTCTTCTGGACACCAAGGGCAAACTCTATCGCTGGCGGTCTCCCGTCATCGTAGAGAAAGGGGGTAAGGTTGAGGTCGAAGGCCACCTGATCGACCTCAAGAGAGTTGTGCTAGACGGCTCCGCGGCGACTCCCATAACCAAAGTTTCAGCGGAGCAATGGGGTCGTCCTTAG

>CF-21xiquchanfangzizhuzuzhiye20230804

ATGTTGGGGAAATGCTTGACCGCGGGCTGCTGCTCGCAATTTCTTTTTTTGTGGTGTATCGTGCCGTTCTGTTTAGTTGCGCTCGTCAACGCCAACAACAGCAGCAGCTCCCACTTACAGTTGATTTATAACCTGACGATATGTGAGCTGAATGGCACAGATTGGCTAAATAAAAGTTTTGATTGGGCAGTGGAGACCTTTGTTATTTTTCCTGTGTTGACTCATATTGTCTCCTATGGTGCCCTAACCACCAGCCATTTCCTTGATACAGTCGGCCTGATCACCGTGTCTACCGCTGGATATTACCACGGGCGGTATGTCTTGAGCAGCATTTACGCCGTCTGCGCCCTAGCTGCATTAACTTGCTTCGTCATCAGGCTAACAAAAAATTGCATGTCCTGGCGTTACTCATGCACCAGATACACTAACTTTCTTCTGGACACCAAGGGCAAACTCTATCGCTGGCGGTCTCCCGTCATCGTAGAGAAAGGGGGTAAGGTTGAGGTCGAAGGCCACCTGATCGACCTCAAGAGAGTTGTGCTAGACGGTTCCGCGGCGACTCCCATAACCAAAGTTTCAGCGGAGCAATGGGGTCGTCCTTAG

>CF-22beiquyufeixueqing20230620

ATGTTGGGGAAATGCTTGACCGCGGGTTATTGCTCACAATTGCCTTTTTTGTGGTGTATCGTGCCGTTCTGTTTTGCTGTGCTCGTCAACGCCAGCGACAACAACAGCTCCCATTTACAGTTGATTTATAACCTGACGATATGTGAGCTGAATGGCACAGACTGGTTGAACCGAAAATTTGACTGGGCAGTAGAGACTTTCGTCATTTTTCCTGTGTTGACCCATATTGTCTCTTACGGCGCCCTTACCACTAGCCATTTTCTTGACACGGTCGGCCTAATCACCGTGTCCACCGCTGGGTATTATCATGAGCGGTATGTATTGAGTAGCATCTACGCTGTCTGTGCCCTGGCCGCGTTGGTTTGCTTCGCCATTAGGTTGGCAAAAAATTGTATGTCCTGGCGCTACTCATTCACCAGATATACTAATTTTCTTCTGGACTCTAAGGGCAAACTCTACCGTTGGCGGTCACCCGTCATCATAGAGAAAGAGGGCAAAGTTGATGTGGGGGGTCAGTTAATTGACCTCAAGAGAGTTGTGCTTGATGGTTCCGCGGCGACCCCTGTAACCAAGATTTCAGCGGAACAATGGGGTCGTCCCTAG

>CF-23GDUzhuzuzhi20230717

ATGTTGGGGAAATGCTTGACCGCGGGTTACTGCTCATCATTGCTTTTTTTGTGGTGTATCGTGCCATCCTGTTTTGTTGCACTCGTCAACGCCAGCAACAACACCAGCTCTCATTTACAGTTGATTTATAACTTGACGATATGTGAGCTGAATGGCACAGATTGGCTAAATAGAAAGTTTGACTGGGCAGTGGAGACTTTCGTCATCTTTCCTGTACTGACTCATATTGTCTCTTATGGCGCCCTTACCACTAGCCATTTTCTTGACACGGTCGGCCTGCTCACTGTGTCCACAGCTGGTTATTTACACAAGCGGTACGTATTGAGTAGCATCTACGTTGTTTGTGCCCTGGCTGCGTTTGTCTGCTTCGCCATTAGGTTGGCGAAAAATTGCATGTCTTGGCGCTACTCATGCACCAGATATACCAACTTTCTCCTGGACACTAAGGGCAAACTCTATCGCTGGCGGTCACCCGTCATCATAGAGAAGGGGGGCAAAGTTGACGTGGGGGGTCATTTAATTGACCTCAAGAGAGTCGTGCTTGATGGTTCCGCGGCGACCCCTATAACCAAGATTTCAGCGGAACAATGGGGTCGTCCATAG

>CF-24yufeizhuxueqing20230620

ATGTTGGGGAAGTGCTTGACCGCATGCTGTTGCTCGCGATTGCTTTTTTTGTGGTGTATCGTGCCGTCCTATCTCATTGTGCCCGTCAACGCCAGCAACAACAACAGCTCTCATATTCAGTTGATTTATAACTTAACGCTATGTGAGCTGAATGGTACAGATTGGCTGGCACAAAAATTTGACTGGGCAGTGGAGACTTTTGTCATCTTCCCCGTGTTGACTCACATTGTTTCCTATGGGGCACTCACCACCAGCCATTTCCTTGACACAGTTGGTCTGGCCACTGTGTCTACCGCCGGATATTATCACGAGCGGTATGTCTTGAGTAGCATTTACGCAGTCTGTGCTCTGGCTGCGCTGATTTGCTTTGTCATTAGGCTTGCGAAGAACTGCATGTCCTGGCGCTACTCTTGTACCAGATATACCAACTTTCTTCTGGACACTAAGGGCAAACTCTATCGTTGGCGGTCGCCCGTCATTGTGGAGAAAGGGGGTAAGGTTGAGGTCGAAGGTCACCTGATCGACCTCAAGAGAGTTGTGCTTGATGGTTCCGCGGCAACCCCTTTAACCAGAGTTTCAGCGGAACGATGGGGTCGTCTCTAG

>CF-24yufeizhuzuzhi20230620

ATGTTGGGGAAATGCTTGATCGCGGGCTGCTGCTCGCAATTGCCTTTTTTGTGGTGTATCGTGCCGTTCTGTTTTGTTGCGCTCGTCAACGCCAACAACAGCAGCAGCTCCCATTTACAGTTGATTTATAACCTGACAATATGTGAGCTGAATGGCACAGATTGGCTGAATGAAAGTTTTGATTGGGCGGTGGAGACCTTTGTTATTTTTCCTGTGTTGACTCATATTGTCTCCTATGGAGCCCTAACCACCAGCCATTTCCTTGACACAGTCGGCCTGATCACCGTGTCTACCGCCGGATATTATCACGGGCGGTATGTCTTGAGCAGCATTTACGCTGTCTGCGCCCTAGCTGCGCTAACTTGCTTCGTCATCAGGCTAACAAAAAACTGCATGTCCTGGCGTTACTCATGTACCAGATACACTAATTTTCTTCTTGACACCAAGGGCAAACTCTATCGTTGGCGGTCTCCCGTCATCGTAGAGAAAGGGGGTAGAGTTGAGATCGAAGGCCATTTAATCGACCTCAAGAGAGTTGTGCTTGACGGTTCCGCTGCGACCCCTATAACCAAAGTTTCAGCGGAACAATGGGGTCGTCCTTAG

>CF-25GDUzhuzuzhi20230620

ATGTTGGGGAGATGCTTGACCGCGGGCTGTTGCTCGCAATTACCTTTTTTGTGGTATATCGTGCCGTTCTGTTTTGTTGTGCTCGTCAACGCCAACAACAGCAGCAGCTCCCATTTACAGTTGATTTATAACCTGACGATATGTGAGCTGAATGGCACAGATTGGCTAAATGAAAATTTTGATTGGGCAGTGGAGACCTTTGTTATTTTCCCTGTGTTGACTCATATTGTCTCCTATGGCGCCCTAACCACCAGCCATTTCCTTGACACAGTTGGCCTGATCACCGTGTCTACCGCTGGATATTATCACGGGCGGTATGTCCTGAGCAGCATTTACGCCGTCTGCGCCCTAGCTGCGTTAACTTGCTTTGTAATCAGGCTAACAAAAAATTGCATGTCCTGGCGTTACTCTTGCACCAGATACACTAGCTTTCTTCTGGACACCAAGGGCAAACTCTATCGTTGGCGGTCTCCCGTCATCGTGGAGAAAGGGGGTAAAGTTGAGGTCGAAGGCCACCTGATCGACCTCAAGAGAGTTGTGCTTGACGGTTCCGCGGCGACCCCTATAACCAAAGTTTCAGCGGAACAATGGTGTCGTCCTTAG

>CF-26beiqubaoyuzhuzuzhiB9-20230622

ATGTTGGAGAAATGCTTGACCGCGGGCTGTTGCTCGCAATTGCTTTCTTTGTGGTGTATCGTGCCGTTCTGTTTTGCTGTGCTCGCCAACGCCAGCAACAACAGCAGCTCCCATCTACAGCTGATTTACAACTTGACGCTATGTGAGCTGAATGGCACAGATTGGCTAGCTAACAAATTTGATTGGGCAGTGGAGAGTTTTGTCATCTTTCCCGTTTTGACTCACATTGTCTCCTATGGTGCCCTCACTACCAGCCATTTCCTTGACACAGTCGCTTTAGTCACTGTGTCTACCGCCGGGTTTGTTCACGGGCGGTATGTCCTAAGTAGCATCTACGCGGTCTGTGCCCTGGCTGCGTTGACTTGCTTCGTCATTAGGTTTGCAAAGAATTGCATGTCCTGGCGCTACGCGTGTACCAGATATACCAACTTTCTTCTGGACACTAAGGGCAGACTCTATCGTTGGCGGTCGCCTGTCATCATAGAGAAAAGGGGCAAAGTTGAGGTCGAAGGTCATCTGATCGACCTCAAAAGAGTTGTGCTTGATGGTTCCGTGGCAACCCCTATAACCAGAGTTTCAGCGGAACAATGGGGTCGTCCTTAG

>CF-26beiqubaoyuzhuzuzhiB15-20230622

ATGTTGGGGAAATGCTTGACCGCGGGCTGCTGCTCGCAATTTCTTTTTTTGTGGTGTATCGTGCCGTTCTGTTTAGTTGCGCTCGTCAACGCCAACAACAGCAGCAGCTCCCACTTACAGTTGATTTATAACCTGACGATATGTGAGCTGAATGGCACAGATTGGCTAAATAAAAGTTTTGATTGGGCAGTGGAGACCTTTGTTGTTTTTCCTGTGTTGACTCATATTGTCTCCTATGGTGCCCTAACCACCAGCCATTTCCTTGATACAGTCGGCCTGATCACCGTGTCTACCGCTGGATACTACCACGGGCGGTATGTCTTGAGCAGCATTTACGCTGTCTGCGCCCTAGCTGCATTAACTTGCTTCGTCATCAGGCTAACAAAAAATTGCATGTCCTGGCGTTATTCATGCACCAGATACACTAACTTTCTTCTGGACACCAAGGGCAAACTCTATCGCTGGCGGTCTCCCGTCATCGTAGAGAAAGGGGGTAAGGTTGAGGTCGAAGGCCACCTGATCGACCTCAAGAGAGTTGTGCTAGACGGTTCCGCGGCGACTCCTATAACCAAAGTTTCAGCGGAGCAATGGGGTCGTCCTTAG

>CF-9houbei2dong06pici20230909

ATGTTGGGGAAATGCTTGACCGCGGGCTACTGCTCGCAATTGCTTTTTTTGTGGTGTATCGTGCCGTCCTGTTTAGTTGCGCTCGTCAACGCTCAAAGAAACAGCAGCTCCCATTTACAGTTGATTTATAACCTGACGATATGTGAGCTGAATGGCACAGATTGGCTAAATGAAACATTTGATTGGGCGGTGGAGACCTTTGTTATTTTTCCTGTGCTGACTCATATTGTCTCCTATGGCGCCCTAACCACCAGCCATTTCCTTGACACAGTCGGCCTGATCACCGTGTCTGCCGCCGGATATTGCCACGGGCGGTATGTTTTGAGCAGCATTTACGCCGTCTGCGCCCTAGCTGCGTTAACTTGTTTCTTCATCAGGCTAACAAAAAATTGCATGTCCTGGCGTTACTCATGCACCAGATACACTAACTTTCTTCTGGACACCAAGGGCAAACTCTATCGTTGGCGGTCTCCTGTCATCGTAGAGAAAGGGGGTAAAGTCGAGGTCGGAGGCCACCTGATCGACCTCAAGAGGGTTGTGCTTGACGGTTCCGCGGCGACCCCTATAACCAAAGTTTCAGCGGAACAATGGGGTCGTCCTTAG

>CF-1houbeimuzhu20230828

ATGTTGGGGAGATGCTTGACCGCGGGCTGTTGCTCGCAATTGCCTTTTTTGTGGTATATCGTGCCGTTCTGTTTTGTTGTGCTCGTCAACGCCAACAACAGCAGCAGCTCCCATTTACAGTTGATTTATAACCTGACGATATGTGAGCTGAATGGCACAGATTGGCTAAATAAAAGTTTTGATTGGGCAGTGGAGACCTTTGTTATTTTCCCTGTGTTGACTCATATTGTCTCCTATGGCGCCCTAACCACCAGCCATTTCCTTGACACAGTTGGCCTGATCACCGTGTCTACCGCTGGGTATTATCACGGGCGGCATGTCCTGAGCAGCATTTACGCCGTCTGTGCCCTAGCTGCGTTAACTTGCTTTGTCATCAGGCTAACAAAAAATTGCATGTCCTGGCGTTACTCATGCACCAGATACACTAACTTTCTTCTGGACACCAAGGGCAAACTCTATCGTTGGCGGTCTCCCGTCATCGTGGAGAAAGGGGGTAAAGTTGAGGTCGAAGGCCACCTGATCGACCTCAAGAGAGTTGTGCTTGACGGTTCCGCGGCGACCCCTATAACCAAAGTTTCAGCGGAACAATGGGGTCGTCCTTAG

>CF-7chanfangzuzhiye20230901

ATGTTGGAGAAATGCTTGACCGCGGGCTGTTGCTCGCAATTGCTTTCTTTGTGGTGTATCGTGCCGTTCTGTTTTGCTGTGCTCGCCAACGCCAGCAACGACAGCAGCTCCCATCTACAGCTGATTTACAACTTGACGCTATGTGAGCTGAATGGCACAGATTGGCTAGCTAACAAATTTGATTGGGCAGTGGAGAGTTTTGTCATCTTTCCCGTTTTGACTCACATTGTCTCCTATGGTGCCCTCACTACCAGCCATTTCCTTGACACAGTCGCTTTAGTCACTGTGTCTACCGCCGGGTTTGTTCACGGGCGGTATGTCCTAAGTAGCATCTACGCGGTCTGTGCCCTGGCTGCGTTGACTTGCTTCGTCATTAGGTTTGCAAAGAATTGCATGTCCTGGCGCTACGCGTGTACCAGATATACCAACTTTCTTCTGGACACTAAGGGCGGACTCTATCGTTGGCGGTCGCCTGTCATCATAGAGAAAAGGGGCAAAGTTGAGGTCGAAGGTCATCTGATCGACCTCAAAAGAGTTGTGCTTGATGGTTCCGTGCCAACCCCTATAACCAGAGCTTCAGCGCAACAATGCAGTCGACCTTAG

>CF-1houbeizhuzuzhi20230823

ATGTTGGGGAGATGCTTGACCGCGGGCTGTTGCTCGCAATTGCCTTTTTTGTGGTATATCGTGCCGTTCTGTTTTGTTGTGCTCGTCAACGCCAACAACAGCAGCAGCTCCCATTTACAGTTGATTTATAACCTGACGATATGTGAGCTGAATGGCACAGATTGGCTAAATAAAAGTTTTGATTGGGCAGTGGAGACCTTTGTTATTTTCCCTGTGTTGACTCATATTGTCTCCTATGGCGCCCTAACCACCAGCCATTTCCTTGACACAGTTGGCCTGATCACCGTGTCTACCGCTGGGTATTATCACGGGCGGCATGTCCTGAGCAGCATTTACGCCGTCTGTGCCCTAGCTGCGTTAACTTGCTTTGTCATCAGGCTAACAAAAAATTGCATGTCCTGGCGTTACTCATGCACCAGATACACTAACTTTCTTCTGGACACCAAGGGCAAACTCTATCGTTGGCGGTCTCCCGTCATCGTGGAGAAAGGGGGTAAAGTTGAGGTCGAAGGCCACCTGATCGACCTCAAGAGAGTTGTGCTTGACGGTTCCGCGGCGACCCCTATAACCAAAGTTTCAGCGGAACAATGGGGTCGTCCTTAG

>CF-21chanfangzuzhiye20230901

ATGTTGGGGAAATGCTTGACCGCGGGCTGCTGCTCGCAATTTCTTTTTTTGTGGTGTATCGTGCCGTTCTGTTTAGTTGCGCTCGTCAACGCCAACAACAGCAGCAGCTCCCACTTACAGTTGATTTATAACCTGACGATATGTGAGCTGAATGGCACAGATTGGCTAAATAAAAGTTTTGATTGGGCAGTGGAGACCTTTGTTATTTTTCCTGTGTTGACTCATATTGTCTCCTATGGTGCCCTAACCACCAGCCATTTCCTTGATACAGTCGGCCTGATCACCGTGTCTACCGCTGGATATTACCACGGGCGGTATGTCTTGAGCAGCATTTACGCCGTCTGCGCCCTAGCTGCATTAACTTGCTTCGTCATCAGGCTAACAAAAAATTGCATGTCCTGGCGTTACTCATGCACCAGATACACTAACTTTCTTCTGGACACCAAGGGCAAACTCTATCGCTGGCGGTCTCCCGTCATCGTAGAGAAAGGGGGTAAGGTTGAGGTCGAAGGCCACCTGATCGACCTCAAGAGAGTTGTGCTAGACGGTTCCGCGGCGACTCCCATAACCAAAGTTTCAGCGGAGCAATGGGGTCGTCCTTAG

>CF-8baoyu2023002picizuzhi20230901

ATGTTGGGGAAATGCTTGACCGCGGGCTGCTGCTCGCAATTGCTTTTTTTGTGGTGTATCGTGCCGTTCTGTTTAGTTTCGCTCGTCAACGCCAACAACAGCAGCAGCTCCCATTTACAGTTGATTTATAACCTGACGATATGTGAGCTGAATGGCACAGATTGGCTAGATAGCAATTTTGATTGGGCAGTGGAGACCTTCGTTATTTTTCCTGTGTTGACTCATATCGTCTCCTATGGCGCCCTAACCACCAGCCACTTCCTTGACACAGTCGGCCTGATCACCGTGTCTACTGCTGGATATTGCCACGGGCGGTATGTCTTGAGCAGTATTTACGCCGTCTGCGCCCTAGCTGCGCTAACTTGCTTCGTCATCAGGCTAACAAAAAATTGCATGTCCTGGCGTTACTCAGGCACCAGATACACTAATTATCTTCTGGACACCAAGGGAAAACTCTATCGTTGGCGGTCTCCCGTCATCGTAGAAAAAGGGGGTAAAGTTGAGGTCGAAGGCCACCTCATCGACCTCAAGAGAGTTGTGCTTGACGGTTCCGCGGCGACCCCTATAACCAAAGTTTCAGCGGAACAATGGGGTCGTCCTTAG

>CF-4baoyuzhuzuzhi20230918

ATGTTGGGGAGATGCTTGACCGCGGGCTATTGCTCGCAATTGCCTTTTTTGTGGTATATCGTGCCGTTCTGTTTTGTTGTGCTCGTCAACGCCAACAACAGCAGCAGCTCCCATTTACAGTTGATTTATAACCTGACGATATGTGAGCTGAACGGCACAAATTGGCTAAATGAAAGTTTTGATTGGGCAGTGGAGACCTTTGTTATTTTCCCTGTGTTGACTCATATTGTCTCCTATGGCGCCCTAACCACCAGCCATTTCCTTGACACAGTTGGCCTGATCACCGTGTCTACCGCTGGATATTATCACGGGCGGTATGTCCTGAGCAGCATTTACGCCGTCTGCGCCCTAGCTGCGTTAACTTGCTTTGTCATCAGGCTAACAAAAAATTGCATGTCCTGGCGTTACTCATGCACCAGATACACTAACTTTCTTCTGGACTCCAAGGGCAAACTCTATCGTTGGCGGTCTCCCGTCATCGTGGAGAAAGGGGGTAAAGTTGAGGTCGAAGGCCACCTGATCGACCTCAAGAGAGTTGTGCTTGACGGTTCCGCGGCGACCCCTATAACCAAAATTTCAGCGGAACAATGGGGTCGTCCTTAG

>CF-4baoyuzhuzuzhi20230918(2)

ATGTTGGGGAAATGCTTGACCGCGGGTTATTGCTCACAATCGCTTTTTTTGTGGTGTATCGTGCCATTCTGTTTTGCTGTGCTCGTCAACGCCAGCGACAACAGCAGCTCCCACTTACAGTTGATCTATAACCTGACGATATGTGAGCTGAATGGCACAGACTGGTTGAACCAAAAATTTGACTGGGCAGTAGAGACTTTCGTCATTTTTCCTGTCTTGACCCATATTGTCTCTTACGGCGCCCTTACCACTAGCCATTTTCTTGACACGGTCGGCCTAATCACCGTATCCACCGCTGGGTATTATCATAAGCGGTATGTATTGAGTAGCATCTACGCTGTCTGTGCCCTGGCCGCGTTGGTTTGCTTCGCCATTAGGTTGGCAAAAAATTGTATGTCCTGGCGCTACTCATTCACCAGATATACTAATTTTCTTCTGGACTCTAAGGGCAAACTCTACCGTTGGCGGTCACCCGTCATCATAGAGAAAGAGGGCAAAGTTGATGTGGGGGGTCAGTTAATCGACCTCAAGAGAGTTGTGCTTGATGGTTCCGCGGCGACCCCTGTAACCAAGATTTCAGCGGAACAATGGGGTCGTCCATAG

>CF-10baoyuzhuzuzhi20230918

ATGTTGGGGAAATGCTTGACCGCGGGCTACTGCTCGCAATTGCTTTTTTTGTGGTGTATCGTGCCGTCCTGTTTAGTTGCGCTCGTCAACGCTCAAAGAAACAGCAGCTCCCATTTACAGTTGATTTATAACCTGACGATATGTGAGCTGAATGGCACAGATTGGCTAAATGAAACATTTGATTGGGCGGTGGAGACCTTTGTTATTTTTCCTGTGCTGACTCATATTGTCTCCTATGGCGCCCTAACCACCAGCCATTTCCTTGACACAGTCGGCCTGATCACCGTGTCTGCCGCCGGATATTGCCACGGGCGGTATGTTTTGAGCAGCATTTACGCCGTCTGCGCCCTAGCTGCGTTAACTTGCTTCGTCATCAGGCTAACAAAAAATTGCATGTCCTGGCGTTACTCATGCACCAGATACACTAACTTTCTTCTGGACACCAAGGGCAAACTCTATCGTTGGCGGTCTCCTGTCATCGTAGAGAAAGGGGGTAAAGTCGAGGTCGGAGGCCACCTGATCGACCTCAAGAGGGTTGTGCTTGACGGTTCCGCGGCGACCCCTATAACCAAAGTTTCAGCGGAACAATGGGGTCGTCCTTAG

>CF-9peihuaimuzhu20230912

ATGTTGGGGAAATGCTTGACCGCGGGCTACTGCTCGCAATTGCTTTTTTTGTGGTGTATCGTGCCGTCCTGTTTAGTTGCGCTCGTCAACGCTCAAAGAAGCAGCAGCTCCCATTTACAGTTGATTTATAACCTGACGATATGTGAGCTGAATGGCACAGATTGGCTAAATGAAACATTTGATTGGGCGGTGGAGACCTTTGTTATTTTTCCTGTGCTGACTCATATTGTCTCCTATGGCGCCCTAACCACCAGCCATTTCCTTGACACAGTCGGCCTGATCACCGTGTCTGCCGCCGGATATTGCCACGGGCGGTATGTTTTGAGCAGCATTTACGCCGTCTGCGCCCTAGCTGCGTTAACTTGCTTCGTCATCAGGCTAACAAAAAATTGCATGTCCTGGCGTTACTCATGCACCAGATACACTAACTTTCTTCTGGACACCAAGGGCAAACTCTATCGTTGGCGGTCTCCTGTCATCGTAGAGAAAGGGGGTAAAGTCGAGGTCGGAGGCCACCTGATCGACCTCAAGAGGGTTGTGCTTGACGGTTCCGCGGCGACCCCTATAACCAAAGTTTCAGCGGAACAATGGGGTCGTCCTTAG

>CF-13peihuaimuzhu20230917

ATGTTGGGGAAGTGCTTGACCGCGTGCTGTTGCTCGCGATTGCTTTTTTTGTGGTGTATTGTGCCGTCCTATCTTGCTGTGCTCGTCAACGCCAGCAACAACAACAGCTCTCATATTCAGTTGATTTATAACTTAACGCTATGTGAGCTGAATGGCACAGATTGGCTGGCACAAAAATTTGACTGGGCAGTGGAGACCTTTGTCATCTTCCCCGTGTTGACTCACATTGTTTCCTATGCGGCACTCACCACCAGCCATTTCCTTGACACAGTTGGTCTGGCCACTGTGTCCACCGCCGGATATTATCACGGGCGGTATGTCTTGAGTAGCATTTACGCAGTCTGTGCTCTGGCTGCGCTGATTTGCTTTGTCATTAGGCTTGCGAAGAACTGCATGTCCTGGCGCTACTCTTGTACCAGATATACCAACTTCCTTCTGGACACTAAGGGCAAACTCTATCGTTGGCGGTCGCCCGTCATTGTGGAGAAAGGGGGTAAGGTTGAGGTCGAAGGTCACCTGATCGACCTCAAGAGAGTTGTGCTTGATGGTTCCGCGGCAACCCCTTTAACCAGAGTTTCAGCGGAACGATGGGGTCGTCTCTAG

>CF-23chanfangzhuzuzhiye20230914

ATGTTGGGGAAATGCTTGACCGCGGGTTACTGCTCATCATTGCTTTTTTTGTGGTGTATCGTGCCATCCTGTTTTGTTGCACTCGTCAACGCCAGCAACAACACCAGCTCTCATTTACAGTTGATTTATAACTTGACGATATGTGAGCTGAATGGCACAGATTGGCTAAATAGAAAGTTTGACTGGGCAGTGGAGACTTTCGTCATCTTTCCTGTACTGACTCATATTGTCTCTTATGGCGCCCTTACCACTAGCCATTTTCTTGACACGGTCGGCCTGCTCACTGTGTCCACAGCTGGTTATTTACACAAGCGGTACGTATTGAGTAGCATCTACGTTGTTTGTGCCCTGGCTGCGTTTGTCTGCTTCGCCATTAGGTTGGCGAAAAATTGCATGTCTTGGCGCTACTCATGCACCAGATATACCAACTTTCTCCTGGACACTAAGGGCAAACTCTATCGCTGGCGGTCACCCGTCATCATAGAGAAGGGGGGCAAAGTTGACGTGGGGGGTCATTTAATTGACCTCAAGAGAGTCGTGCTTGATGGTTCCGCGGCGACCCCTATAACCAAGATTTCAGCGGAACAATGGGGTCGTCCATAG

>CF-12baoyuzhuzuzhi20230919

ATGTTGGAGAAATGCTTGACCGCGGGCTGTTGCTCGCAATTGCTTTCTTTGTGGTGTATCGTGCCGTTCTGTTTTGCTGTGCTCGCCAACGCCAGCAACGTCAGCAGCTCCCATCTACAGCTGATTTACAACTTGACGCTATGTGAGCTGAATGGCACAGATTGGCTAGCTAACAAATTTGATTGGGCAGTGGAGAGTTTTGTCATCTTTCCCGTTTTGACTCACATTGTCTCCTATGGTGCCCTCACTACCAGCCATTTCCTTGACACAGTCGCTTTAGTCACTGTGTCTACCGCCGGGTTTGTTCACGGGCGGTATGTCCTAAGTAGCATCTACGCGGTCTGTGCCCTGGCTGCGTTGACTTGCTTCGTCATTAGGTTTGCAAAGAATTGCATGTCCTGGCGCTACGCGTGTACCAGATACACCAACTTTCTTCTGGACACTAAGGGCATACTCTATCGTTGGCGGTCGCCTGTCATCATAGAGAAAAGGGGCAAAGTTGAGGTCGAAGGTCATCTGATCGACCTCAAAAGAGTTGTGCTTGATGGTTCCGTGGCAACCCCTATAACCAGAGTTTCAGCGGAACAATGGGGTCGTCCTTAG

>CF-12baoyuzhuzuzhi-20230919

ATGTTGGTGAAATGCTTGACCGCGGGCTACTGCTCGCAATTGCTTTTTTTGTGGTGTATCGTGCCGTTCTGTTTAGTTGCGCTCGTCAACGCTCAMAAAAACAGCAGCTCCCATTTACAGTTGATTTATAACCTGACGATATGTGAGCTGAATGGCACAGATTGGCTAAATGAAACATTTGATTGGGCGGTGGAGACCTTTGTTATTTTTCCTGTGCTGACTCATATTGTCTCCTATGGCGCCCTAACCACCAGCCATTTCCTTGACACGGTYGGCCTGATCGCCGTGTCTGCCGCCGGATATTACCACGGGCGGTATGTTTTGAGCAGCATTTACGCCGTCTGCGCCCTAGCTGCGTTAACTTGCTTCGTCATCAGGCTAACAAAAAATTGCATGTCCTGGCGTTAYTCATGCACCAGATACACTAACTTTCTTCTGGACACCAAGGGCAAACTCTATCGTTGGCGGTCTCCTGTCATCGTAGAGAAAGGGGGTAAAGTCGAGGTCGGAGGCCACCTGATCGACCTCAAGAGGGTTGTGCTTGACGGTTCCGCGGCGACCCCTATAACCAAAGTTTCAGCGGAACAATGGGGTCGTCCTTAG

>CF-6yufeizhuzuzhi2023002pici-20230922

ATGTTGGGGAAATGCTTGACCGCGGGCTACTGCTCGCAATTGCTTTTTTTGTGGTGTATCGTGCCGTCCTGTTTAGTTGCGCTCGTCAACGCTCAAARAAACAGCAGCTCCCATTTACAGTTGATTTATAACCTGACGATATGTGAGCTGAATGGCACAGATTGGCTAAATGAAACATTTGATTGGGCGGTGGAGACCTTTGTTATTTTTCCTGTGCTGACTCATATTGTCTCCTATGGCGCCCTAACCACCAGCCATTTCCTTGACACAGTCGGCCTGATCACCGTGTCTGCCGCCGGATATTGCCACGGGCGGTATGTTTTGAGCAGCATTTACGCCGTCTGCGCCCTAGCTGCGTCAACTTGCTTCGTCATCAGGCTAACAAAAAATTGTATGTCCTGGCGTTACTCATGCACCAGATACACTAACTTTCTTCTGGACACCAAGGGCAAACTCTATCGTTGGCGGTCTCCTGTCATCGTAGAGAAAGGGGGTAAAGTCGAGGTCGGAGGCCACCTGATCGACCTCAAGAGGGTTGTGCTTGACGGTTCCGCGGCGACTCCTKTAACCAAAGTTTCAGCGGAACAATGGGGTCGTCCTTAG

>CF-6baoyuzuzhi2023003pici-20230929

ATGTTGGGGAAATGCTTGACCGCGGGCTACTGCTCGCAATTGCTTTTTTTGTGGTGTATCGTGCCGTCCTGTTTAGTTGCGCTCGTCAACGCTCAAAGAAACAGCAGCTCCCATTTACAGTTGATTTATAACCTGACGATATGTGAGCTGAATGGCACAGATTGGCTAAATGAAACATTTGATTGGGCGGTGGAGACCTTTGTTATTTTTCCTGTGCTGACTCATATTGTCTCCTATGGCGCCCTAACCACCAGCCATTTCCTTGACACAGTCGGCCTGATCACCGTGTCTGCCGCCGGATATTGCCACGGGCGGTATGTTTTGAGCAGCATTTACGCCGTCTGCGCCCTAGCTGCGTTAACTTGCTTCGTCATCAGGCTAACAAAAAATTGTATGTCCTGGCGTTACTCATGCACCAGATACACTAACTTTCTTCTGGACACCAAAGGCAAACTCTATCGTTGGCGGTCTCCTGTCATCGTAGAGAAAGGGGGTAAAGTCGAGGTCGGAGGCCACCTGATCGACCTCAAGAGGGTTGTGCTTGACGGTTCCGCGGCGACTCCTATAACCAAAGTTTCAGCGGAACAATGGGGTCGTCCTTAG

>CF-7houbeizhuxueqing20230927

ATGTTGGGGAAGTGCTTGACCGCGTGCTGTTGCTCGCGATTGCTTTTTTTGTGGTGTATTGTGCCGTCCTATCTTGCTGTGCTCGTCAACGCCAGCAACAACAACAGCTCTCATATTCAGTTGATTTATAACTTAACGCTATGTGAGCTGAATGGCACAGATTGGCTGGCACAAAAATTTGACTGGGCAGTGGAGACCTTTGTCATCTTCCCCGTGTTGACTCACATTGTTTCCTATGCGGCACTCACCACCAGCCATTTCCTTGACACAGTTGGTCTGGCCACTGTGTCCACCGCCGGATATTATCACGGGCGGTATGTCTTGAGTAGCATTTACGCAGTCTGTGCTCTGGCTGCGCTGATTTGCTTTGTCATTAGGCTTGCGAAGAACTGCATGTCCTGGCGCTACTCTTGTACCAGATATACCAACTTCCTTCTGGACACTAAGGGCAAACTCTATCGTTGGCGGTCGCCCGTCATTGTGGAGAAAGGGGGTAAGGTTGAGGTCGAAGGTCACCTGATCGACCTCAAGAGAGTTGTGCTTGATGGTTCCGCGGCAACCCCTTTAACCAGAGTTTCAGCGGAACGATGGGGTCGTCTCTAG

>CF-19nanqupeihuaixueqing20230925

ATGTTGGGGAAATGCTTGACCGCGGGTTACTGCTCATCATTGCTTTTTTTGTGGTGTATCGTGCCATCCTGTTTTGTTGCACTCGTCAACGCCAGCAACAACACCAGCTCTCATTTACAGTTGATTTATAACTTGACGATATGTGAGCTGAATGGCACAGATTGGCTAAATAGAAAGTTTGACTGGGCAGTGGAGACTTTCGTCATCTTTCCTGTACTGACTCATATTGTCTCTTATGGCGCCCTTACCACTAGCCATTTTCTTGACACGGTCGGCCTGCTCACTGTGTCCACTGCTGGTTATTTACACAAGCGGTACGTATTGAGTAGCATCTACGTTGTTTGTGCCCTGGCTGCGTTTGTCTGCTTCGCCATTAGGTTGGCGAAAAATTGCATGTCTTGGCGCTACTCATGCACCAGATATACCAACTTTCTCCTGGACACTAAGGGCAAACTCTATCGCTGGCGGTCACCCGTCATCATAGAGAAGGGGGGCAAAGTTGACGTGGGGGGTCATTTAATTGACCTCAAGAGAGTCGTGCTTGATGGTTCCGCGGCGACCCCTATAACCAAGATTTCAGCGGAACAATGGGGTCGTCCATAG

>CF-21xiqufenmianzizhuduanweiye20230929

ATGTTGGGGAAATGCTTGACCGCGGGCTGCTGCTCGCAATTTCTTTTTTTGTGGTGTATCGTGCCGTTCTGTTTAGTTGCGCTCGTCAACGCCAACAACAACAGCAGCTCCCACTTACAGTTGATTTATAACCTGACGATATGTGAGCTGAATGGCACAGATTGGCTAAATAAAAGTTTTGATTGGGCAGTGGAGACCTTTGTTATTTTTCCTGTGTTGACTCATATTGTCTCCTATGGTGCCCTAACCACCAGCCATTTCCTTGATACAGTCGGCCTGATCACCGTGTCTACCGCTGGATATTACCACGGGCGGTATGTCTTGAGCAGCATTTACGCCGTCTGCGCCCTAGCTGCATTAACATGCTTCGTCATCAGGCTAACAAAAAATTGCATGTCCTGGCGTTACTCATGCACCAGATACACTAACTTTCTTCTGGACACCAAGGGCAAACTCTATCGCTGGCGGTCTCCCGTCATCGTAGAGAAAGGGGGTAAGGTTGAGGTCGAAGGCCACCTGATCGACCTCAAGAGAGTTGTGCTAGACGGTTCCGCGGCGACTCCCATAACCAAAGTTTCAGCGGAGCAATGGGGTCGTCCTTAG

>CF-23nanqufenmianzizhuduanweiye20231004

ATGTTGGGGAAATGCTTGACCGCGGGTTACTGCTCATCATTGCTTTTTTTGTGGTGTATCGTGCCATCCTGTTTTGTTGCACTCGTCAACGCCAGCAACAACACCAGCTCTCATTTACAGTTGATTTATAACTTGACGATATGTGAGCTGAATGGCACAGATTGGCTAAATAGAAAGTTTGACTGGGCAGTGGAGACTTTCGTCATCTTTCCTGTACTGACTCATATTGTCTCTTATGGCGCCCTTACCACTAGCCATTTTCTTGACACGGTCGGCCTGCTCACTGTGTCCACAGCTGGTTATTTACACAAGCGGTACGTATTGAGTAGCATCTACGTTGTTTGTGCCCTGGCTGCGTTTGTCTGCTTCGCCATTAGGTTGGCGAAAAATTGCATGTCTTGGCGCTACTCATGCACCAGATATACCAACTTTCTCCTGGACACTAAGGGCAAACTCTATCGCTGGCGGTCACCCGTCATCATAGAGAAGGGGGGCAAAGTTGACGTGGGGGGTCATTTAATTGACCTCAAGAGAGTCGTGCTTGATGGTTCCGCGGCGACCCCTATAACCAAGATTTCAGCGGAACAATGGGGTCGTCCATAG

>CF-13peihuai3dongliuchanzhu20231004

ATGTTGGGGAAATGCTTGACCGCGGGCTGCTGCTCGCAATTGCTTTTTTTGTGGTGTATCGTGCCGTTCTGTTTGGTTTCGCTCGTCAACGCCAACGACGGCAGCAGCTCCCATTTACAGTTGATTTATAACCTGACGATATGTGAGCTGAATGGCACAGATTGGCTAGATAGCAATTTTGATTGGGCAGTGGAGACCTTCGTTCTTTTTCCTGTGTTGACTCACATCGTCTCCTATGGCGCCCTAACCACCAGCCACTTCCTTGACACAGTCGGCCTGATCACCGTGTCTACTGCTGGATATTGCCACGGGCGGTATGTCTTGAGCAGTATTTACGCCGTCTGCGCCCTAGCTGCGCTAATTTGCTTCGTCATCAGGTTAACAAAAAATTGCATGTCCTGGCGTTACTCATGCACCAGATACACTAATTTTCTTCTGGACACCAAGGGAAAACTCTATCGTTGGCGGTCTCCCGTCATCGTAGAAAAAGGGGGTAAAGTTGAGGTCGAAGGCCACCTCATCGACCTCAAGAAAGTTGTGCTTGACGGTTCCGCGGCGACCCCTATAACCAAAGTTTCAGCGGAACAATGGGGTCGTCCTTAG

>CF-23beiqufenmianzhuzuzhi20231013

ATGTTGGGGAAATGCTTGACCGCGGGATGTTGCTCGCAATTGCTTTTTTTGTGGTATATCGTGCCATTCTGTTTTGTTGTGCTCGTCAACGCCAACA---ACAGCAGCTCCCATTTACAGTTGATTTATAACCTGACGATATGTGAGCTGAATGGCACAGAATGGCTGGATGGACATTTTAGCTGGGCAGTGGAGACTTTCGTCATCTTTCCTGCGTTGACTCACATTGTCTCTTACGGTGCTGTTACCACCAGCCATCTGCTTGACGCAGCCGGCCTGACCATTGTGTCCACCGCCGGTTATGTGCACAAGCGGTATGTATTGAGTAGCATTTACGCTGTCTGTGCTTTGGCTGCGTTGATTTGCTTTGCTATTAGGTTGGCAAAAAATTGCATGTCCTGGCGCTACTCATGCACCAGATACACCAATTTTCTTCTGGATACTAAAGGCAAACTCTACCGATGGCGGTCATCTGTCATCATAGAAAAAGGGGGTAAGGTTGACATWGGGGGTCATTTGATTGACCTCAAAAGAGTTGTGCTTGATGGTTCCGCGGCAGCCCCTGTAACCAAGATTTCAGCGGAACAATGGGGTCATCCATAG

>CF-5chanfangzizhuduanweiye20231013

ATGTTGGGGAAATGCTTGACCGCGGGTTACTGCTCATCATTGCTTTTTTTGTGGTGTATCGTGCCATCCTGTTTTGTTGCACTCGTCAACGCCAGCAACAACACCAGCTCTCATTTACAGTTGATTTATAACTTGACGATATGTGAGCTGAATGGCACAGATTGGCTAWATAGAAAGTTTGACTGGGCAGTGGAGACTTTCGTCATCTTTCCTGTACTGACTCATATTGTCTCTTATGGCGCCCTTACCACTAGCCATTTTCTTGACACGGTCGGCCTGCTCACTGTGTCCACAGCTGGTTATTTACACAAGCGGTACGTATTGAGTAGCATCTACGTTGTTTGTGCCCTGGCTGCGTTTGTCTGCTTCGTCATTAGGTTGGCGAAAAATTGCATGTCTTGGCGCTACTCATGCACCAGATATACCAACTTTCTCCTGGACACTAAGGGCAAACTCTATCGCTGGCGGTCACCCGTCATCATAGAGAAGGGGGGCAAAGTTGACGTGGGGGGTCATTTAATTGACCTCAAGAGAGTCGTGCTTGATGGTTCCGCGGCGACCCCTATAACCAAGATTTCAGCGGAACAATGGGGTCGTCCATAG

>CF-15chanfangzizhuduanweiye20231019

ATGTTGGGGAAATGCTTGACCGCGGGCTGCTGCTCGCAATTGCTTTTTTTGTGGTGTATCGTGCCGTTCTGTTTAGTTTCGCTCGTCAACGCCAACAACAGAAGCAGCTCCCATTTACAGTTGATTTATAACCTGACGATATGTGAGCTGAATGGCACAGATTGGCTAGATAGCAATTTTGATTGGGCAGTGGAGACCTTCGTTATTTTTCCTGTGTTGACTCATATCGTCTCCTATGGCGCCCTAACCACCAGCCACTTCCTTGACACAGTCGGCCTGATCACCGTGTCTACTGCTGGATATTACCACGGGCGGTATGTCTTGAGCAGTATTTACGCCGTCTGCGCCCTAGCTGCGCTAACTTGCTTCGTCATCAGGCTAACAAAAAATTGCATGTCCTGGCGTTACTCATGCACCAGATACACTAATTTTCTTCTGGACACCAAGGGAAAACTCTATCGTTGGCGGTCTCCCGTCATTGTAGAAAAAGGGGGTAAAGTTGAGGTCGAAGGCCACCTCATCGACCTCAAGAAAGTTGTGCTTGACGGTTCCGCGGCGACCCCTATAACCAAAGTTTCAGCGGAACAATGGGGTCGTCCTTAG

>CF-13houbeishezhubiantaoti20231020

ATGTTGGGGAAGTGCTTGACCGCGTGCTGTTGCTCGCGATTGCTTTTTTTGTGGTGTATTGTGCCGTCCTATCTTGCTGTGCTCGTCAACGCCAGCAACAACAACAGCTCTCATATTCAGTTGATTTATAACTTAACGCTATGTGAGCTGAATGGCACAGATTGGCTGGCACAAAAATTTGACTGGGCAGTGGAGACCTTTGTCATCTTCCCCGTGTTGACTCACATTGTTTCCTATGCGGCACTCACCACCAGCCATTTCCTTGACACAGTTGGTCTGGCCACTGTGTCCACCGCCGGATATTATCACGGGCGGTATGTCTTGAGTAGCATTTACGCAGTCTGTGCTCTGGCTGCGCTGATTTGCTTTGTCATTAGGCTTGCGAAGAACTGCATGTCCTGGCGCTACTCTTGTACCAGATATACCAACTTCCTTCTGGACACTAAGGGCAAACTCTATCGTTGGCGGTCGCCCGTCATTGTGGAGAAAGGGGGTAAGGTTGAGGTCGAAGGTCACCTGATCGACCTCAAGAGAGTTGTGCTTGATGGTTCCGCGGCAACCCCTTTAACCAGAGTTTCAGCGGAACGATGGGGTCGTCTCTAG

>CF-19nanqufenmianzizhuduanweiye20231018

ATGCTGGGGAAATGCTTGACCGCGGCTTACTGCTCATCATTGCTTTTTTTGTGGTGTATCGTGCCATCCTGTTTTGTTGCACTCGTCAACGCCAGCAACAACACCAGCTCTCCTTTACTGTTGATTTATAACTTGACGATATGTGAGCTGAATGGCACAGATTGGCTAAATAGAAAGTTTGACTGGGCAGTGGAGATTTTCGTCATCTTTCCTGTACTGACTCATATTGTCTCTTATGGCGCCCTTACCACTAGCCATTTTCTTGACACGGTCGGCCTGCTCACTGTGTCCACTGCTGGTTATTTACACAAGCGGTACGTATTGAGCAGCATCTACGTTGTTTGTGCCCTGGCTGCGTTTGTCTGCTTCGCCATTAGGTTGGCGAAAAATTGCATGTCTTGGCGCTACTCATGCACCAGATATACCAACTTTCTCCTGGACACTAAGGGCAAACTCTATCGCTGGCGGTCACCCGTCATCATAGAGAAGGGGGGCAAAGTTGACGTGGGGGGTCATTTAATTGACCTCAAGAGAGTCGTGCTTGATGCTTCCGCGGCGACCCCTATAACCAAGATTTCAGCGGAACAATGTGGCCGTCCATAG

>CF-25yiqufenmianzhuzuzhi20231019

ATGTTGGGGAGATGCTTGACCGCGGGCTGTTGCTCGCAATTGCCTTTTTTGTGGTATATCGTGCCGTTCTGTTTTGTTGTGCTCGTCAACGCCAACGACAGCAGCAGCTCCCATTTACAGTTGATTTACAACCTGACGATATGTGAGCTGAATGGCACAGATTGGCTAAATGAAAATTTTGATTGGGCAGGGGAGACCTTTGTTATTTTCCCTGTGTTGACTCATATTGTCTCCTATGGCGCCCTAACCACCAGCCATTTCCTTGACACAGTTGGTCTGATCACCGTGTCTACCGCTGGATATTATCACGGGCGGTATGTCCTGAGCAGCATTTACGCCGTCTGCGCCCTAGCTGCGTTAACTTGCTTTGTAATCAGGCTAACGAAAAATTGCATGTCCTGGCGTTATTCTTGCACCAGATACACTAGCTTTCTTCTGGACACCAAGGGCAAACTCTATCGTTGGCGATCTCCCGTCATCGTGGAGAAAGGGGGTAAAGTTGAGGTCGAAGGCCACCTGATCGACCTCAAGAGAGTTGTGCTTGACGGTTCCGCGGCGACCCCTGTAACCAAAATTCCAGCGGAACAATGGGGTCGTCCTTAG

>CF-25GDUzhuzuzhi20231020

ATGTTGGGGAGATGCTTGACCGCGGGCTGTTGCTCGCAATTGCCTTTTTTGTGGTATATCGTGCCGTTCTGTTTTGTTGTGCTCGTCAACGCCAACGACAGCAGCAGCTCCCATTTACAGTTGATTTACAACCTGACGATATGTGAGCTGAATGGCACAGATTGGCTAAATAAAAGTTTTGATTGGGCAGTGGAGACCTTTGTTATTTTCCCTGTGTTGACTCATATTGTCTCCTATGGCGCCCTAACCACCAGCCATTTCCTTGACACAGTTGGTCTGATCACCGTGTCTACCGCTGGATATTATCACGGGCGGTATGTCCTGAGCAGCATTTACGCCGTCTGCGCCCTAGCTGCGTTAACTTGCTTTGTAATCAGGCTAACGAAAAATTGCATGTCCTGGCGTTATTCTTGCACCAGATACACTAGCTTTCTTCTGGACACCAAGGGCAAACTCTATCGTTGGCGATCTCCCGTCATCGTGGAGAAAGGGGGTAAAGTTGAGGTCGAAGGCCACCTGATCGACCTCAAGAGAGTTGTGCTTGACGGTTCCGCGGCGACCCCTGTAACCAAAATTGCAGCGGAACAATGGGGTCGTCCTTAG

>Sangong-zhuzuzhi20231021

ATGTTGGGGAGATGCTTGACCGCGGACTGCTGCTCGCACTTGCTTTTTTTGTGGTATATCGTGCCGTTTTGTTTTGTTGCGCTCGTCAACGCCAACAACAACGACAGCTCCCACTTACAATTGATTTATAACCTGACGATATGTGAGCTGAATGGCACAGATTGGCTAAATAGAAGTTTTGATTGGGCAGTGGAGACCTTTGTTATTTTCCCTGTGTTGACTCATATTGTCTCCTATGGCGCCCTAACCACCAGCCATTTCCTTGACACAGTTGGCCTGATCACCGTGTCTACCGCTGGATATTGTCACGGGCGGTATGTCTTGAGCAGCATTTACGCCGTCTGCGCCCTAGCTGCGTTAACTTGCTTCGTCATCAGGCTAACAAAAAATTTCATGTCCTGGCGTTATTCATGCACCAGATATACTAACTTTCTTCTGGACACCAAGGGCAAACTCTATCGTTGGCGGTCTCCCGTCATCGTAGAGAAAGGGGGTAAAGTTGAGGTCGAAGGCCACCTGATCGACCTCAAGAGAGTTGTGCTTGACGGTTCCGCGGCGACCCCTATAACCAAAGTTTCAGCGGAACAATGGGGTCGTCCTTAG

>CF-19nanqupeihuaizhuxueqing20231024

ATGTTGGGGAAATGCTTGACCGCGGGTTACTGCTCATCATTGCTTTTTTTGTGGTGTATCGTGCCATCCTGTTTTGTTGCACTCGTCAACGCCAGCAACAACACCAGCTCTCATTTACAGTTGATTTATAACTTGACGATATGTGAGCTGAATGGCACAGATTGGCTAAATAGAAAGTTTGACTGGGCAGTGGAGACTTTCGTCATCTTTCCTGTACTGACTCATATTGTCTCTTATGGCGCCCTTACCACTAGCCATTTTCTTGACACGGTCGGCCTGCTCACTGTGTCCACTGCTGGTTATTTACACAAGCGGTACGTATTGAGTAGCATCTACGTTGTTTGTGCCCTGGCTGCGTTTGTCTGCTTCGCCATTAGGTTGGCGAAAAATTGCATGTCTTGGCGCTACTCATGCACCAGATATACCAACTTTCTCCTGGACACTAAGGGCAAACTCTATCGCTGGCGGTCACCCGTCATCATAGAGAAGGGGGGCAAAGTTGACGTGGGGGGTCATTTAATTGACCTCAAGAGAGTCGTGCTTGATGGTTCCGCGGCGACCCCTATAACCAAGATTTCAGCGGAACAATGGGGTCGTCCATAG

>CF-1houbeizhuxueqing20231025

ATGTTGGGGAGATGCTTGACCGCGGGCTGTTGCTCGCAATTGCCTTTTTTGTGGTATATCGTGCCGTTCTGTTTTGTTGTGCTCGTCAACGCCAACAACAGCAGCAGCTCCCATTTACAGTTGATTTATAACCTGACGATATGTGAGCTGAATGGCACAGATTGGCTAAATAAAAGTTTTGATTGGGCAGTGGAGACCTTTGTTATTTTCCCTGTGTTGACTCATATTGTCTCCTATGGCGCCCTAACCACCAGCCATTTCCTTGACACAGTTGGCCTGATCACCGTGTCTACCGCTGGGTATTATCACGGGCGGTATGTCCTGAGCAGCATTTACGCCGTCTGCGCCCTAGCTGCGTTAACTTGCTTTGTCATCAGGCTAACAAAAAATTGCATGTCCTGGCGTTACTCATGCACCAGATACACTAACTTTCTTCTGGACACCAAGGGCAAACTCTATCGTTGGCGGTCTCCCGTCATCGTGGAGAAAGGGGGTAAAGTTGAGGTCGAAGGCCACCTGATCGACCTCAAGAGAGTTGTGCTTGACGGTTCCGCGGCGACCCCTATAACCAAAGTTTCAGCGGAACAATGGGGTCGTCCTTAG

>CF-13chanfangzizhuduanweiye20231024

ATGTTGGGGAAATGCTTGACCGCGGGCTGCTGCTCGCAATTGCTTTTTTTGTGGTGTATCGTGCCGTTCTGTTTAGTTTCGCTCGTCAACGCCAGCAACAGCAGCAGCTCCCATTTACAGTTGATTTATAACCTGACGATATGTGAGCTGAATGGCACAGATTGGCTAGATAGCAATTTTGATTGGGCAGTGGAGACCTTCGTTATTTTTCCTGTGTTGACTCATATCGTCTCCTATGGCGCCCTAACCACCAGCCACTTCCTTGACACAGTCGGCCTGATCACCGTGTCTACTGCTGGATATTGCCACGGGCGGTATGTCTTGAGCAGTATTTACGCCGTCTGCGCCTTAGCTGCGCTAATTTGCTTCGTCATCAGGCTAACAAAAAATTGCATGTCCTGGCGTTACTCATGCACCAGATACACTAATTTTCTTCTGGACACCAAGGGAAAACTCTATCGTTGGCGGTCTCCCGTCATCGTAGAAAAAGGGGGTAAAGTTGAGGTCGAAGGCCACCTCATCGACCTCAAGAAAGTTGTGCTTGACGGTTCCGCGGCGACCCCTATAACCAAAGTTTCAGCGGAACAATGGGGTCGTCCTTAG

>CF-1houbeishezhuxueqing20240302

ATGTTGGGGAGATGCTTGACCGCGGGCTGTTGCTCGCAATTGCCTTTTTTGTGGTATATCGTGCCGTTCTGTTTTGTTGTGCTCGTCAACGCCAACAACAGCAGCAGCTCCCATTTACAGTTGATTTATAACCTGACGATATGTGAGCTGAATGGCACAGATTGGCTAAATAAAAGTTTTGATTGGGCAGTGGAGACCTTTGTTATTTTCCCTGTGTTGACTCATATTGTCTCCTATGGCGCCCTAACTACCAGCCATTTCCTTGACACAGTTGGCCTGATCACCGTGTCTACCGCTGGGTATTATCACGGGCGGTATGTCCTGAGCAGCATTTACGCCGTCTGCGCCCTAGCTGCGTTAACTTGCTTTGTCATCAGGCTAACAAAAAATTGCATGTCCTGGCGTTACTCATGCACCAGATACACTAACTTTCTTCTGGACACCAAGGGCAAACTCTATCGTTGGCGGTCTCCCGTCATCGTGGAGAAAGGGGGTAAAGTTGAGGTCGAAGGCCACCTGATCGACCTCAAGAGAGTTGTGCTTGACGGTTCCGCGGCGACCCCTATAACCAAAGTTTCAGCGGAACAATGGGGTCGTCCTTAG

>CF-25peihuaizhuzuzhi20240521

ATGTTGGGGAAATGCTTGACCGCGGGTTATTGCTCACAATCGCTTTTTTTGTGGTGTATCGTGCCATTCTGTTTTGCTGTGCTCGTCAACGCCGGCAACAACAGCAGCTCCCACTTACAGTTGATCTATAACCTGACGATATGTGAGCTGAATGGCACAGACTGGTTGAACCAAAAATTTGACTGGGCAGTAGAGACTTTCGTCATTTTTCCAGTCTTGACCCATATTGTCTCTTACGGCGCCCTTACCACTAGCCATTTTCTTGATACGGTCGGCCTAATCACCGTATCCACCGCTGGGTATTATCATAAGCGGTATGTATTGAGTAGCATTTACGCTGTCTGTGCCCTGGCCGCGTTGGTTTGCTTCGCCATTAGGTTGGCAAAAAATTGTATGGCCTGGCGCTACTCATTCACCAGACATACTAATTTTCTTCTGGACTCTAAGGGCAAACTCTACCGTTGGCGGTCACCCGTCATCATAGAGAAAGAGGGCAAAGTTGATGTGGGGGGTCAGTTGATCGACCTCAAGAGAGTTGTGCTTGATGGTTCCGCGGCGACCCCTGTAACCAAGATTTCAGCGGAACAATGGGGTCGTCCATAG

>CF-5chanfangzizhuduanweiye20240503

ATGTTGGGGAAATGCTTGACCGCGGGTTACTGCTCATCATTGCTTTTTTTGTGGTGTATCGTGCTATCCTGTTCTGTTGCGCTCGTCAACGCCAACAGCAACACCAGCTCTCATTTACAGTTGATTTATAACCTGACGATATGTGAGCTGAATGGCACGGATTGGTTGGACGAAAGATTTGACTGGGCAGTAGAGACTTTCGTCATTTTTCCTGTTCTGACCCATATTGTCTCTTACGGCGCCCTCACCACTAGCCATTTTCTTGACACGGTCGGCCTAATCACTGTGTCCACCGCTGGGTATTACCATAAGCGGTATGTATTGAGTAGCATCTATGCTGTCTGTGCCCTGGCCGCGTTGGCTTGCTTCGCCATCAGGTTGGCGAAAAATTGCATGTCCTGGCGCTACTCATGCACCAGATACACCAACTTTCTCCTGGACACTAAAGGCAAACTCTACCGCTGGCGGTCACCCGTCATCATAGAGAAGGGGGGCAAAGTTGATGTGGGGGGTCATTTAATTGACCTCAAGAGAGTCGTGCTTGATGGTTCCGCGGCGACCCCTATAACCAAGATTTCAGCGGAACAATGGGGTCGTCCATAG

>CF-6baoyuzhuzuzhi20240503

ATGTTGGGGAAATGCTTGACCGCGGGTTACTGCTCATCATTGCTTTTTTTGTGGTGTATCGTGCTATCCTGTTCTGTTGCGCTCGTCAACGCCGACAGCAACACCAGCTCTCATTTACAGTTGATTTATAACCTGACGATATGTGAGCTGAATGGCACGGATTGGTTGAACAAAAGATTTGACTGGGCAGTAGAGACTTTCGTCATTTTTCCTGTTCTGACCCATATTGTCTCTTACGGCGCCCTCACCACTAGCCATTTTCTTGACACGGTCGGCCTAATTACTGTGTCCACCGCTGGGTATTACCATAAGCGGTATGTATTGAGTAGCATCTATGCTGTCTGTGCCCTGGCCGCGTTGGCTTGCTTCGCCATCAGGTTGGCGAAAAATTGCATGTCCTGGCGCTACTCATGCACCAGATACACCAACTTTCTCCTGGACACTAAAGGCAGACTCTACCGCTGGCGGTCACCCGTCATCATAGAGAAGGGGGGCAAAGTTGATGTGGGGGGTCATTTAATTGACCTCAAGAGAGTCGTGCTTGATGGTTCCGCGGCGACCCCTATAACCAAGATTTCAGCGGAACAATGGGGTCGTCCATAG

>CF-11chanfangzizhuduanweiye20240503

ATGTTGGGGAAATGCTTGACCGCGGGTTATTGCTCACAATCGCTTTTTTTGTGGTGTATCGTGCCATTCTGTTTTGCTGTGCTCGTCAACGCCAGCAACAACAGCGGCTCCCACTTACAGTTGATCTATAACCTGACGATATGTGAGCTGAATGGCACAGACTGGTTGAACCAAAAATTTGACTGGGCAGTAGAGACTTTCGTCATTTTTCCTGTCTTGACCCATATTGTCTCTTACGGCGCCCTTACCACTAGCCATTTTCTTGACACGGTCGGACTAATCACCGTATCCACCGCTGGGTATTATCATGAGCGGTATGTATTGAGTAGCATCTACGCTGTCTGTGCCCTGGCCGCGTTGGTTTGCTTCGCCATTAGGTTGGCAAAAAATTGTATGTCCTGGCGCTACTCATTCACCAGATATACCAATTTTCTTCTGGACTCTAAGGGCAAACTCTACCGTTGGCGGTCACCCGTCATCATAGAGAAAGAGGGCAAAGTTGATGTGGGGGGTCAGTTAATCGACCTCAAGAGAGTAGTGCTTGATGGTTCCGCGGCGACCCCTGTAACCAAGATTTCAGCGGAACAATGGGGTCGTCCATAG

>CF-9chanfangzizhuduanweiye20240516

ATGTTGGGGAAATGCTTGACCGCGGGCTACTGCTCGCAATTGCTTTTTTTGTGGTGTATCGTGCCGTCCTGTTTAGTTGCGCTCGTCAACGCTCAAAGAAACAGCAGCTCCCATTTACAGTTGATTTATAACCTGACGATATGTGAGCTGAATGGCACAGATTGGCTAAATGAAACATTTGATTGGGCGGTGGAGACCTTTGTTATTTTTCCTGTGCTGACTCATATTGTCTCCTATGGCGCCCTAACCACCAGCCATTTCCTTGACACAGTCGGCCTGATCACCGTGTCTGCCGCCGGATATTGCCACGGGCGGTATGTTTTGAGCAGCATTTACGCCGTCTGCGCCCTAGCTGCGTTAACTTGCTTCGTCATCAGGCTAACAAAAAATTGTATGTCCTGGCGTTACTCATGCACCAGATACACTAACTTTCTTCTGGACACCAAGGGCAAACTCTATCGTTGGCGGTCTCCTGTCATCGTAGAGAAAGGGGGTAAAGTCGAGGTCGGAGGCCACCTGATCGACCTCAAGAGGGTTGTGCTTGACGGTTCCGCGGCGACTCCTATAACCAAAGTTTCAGCGGAACAATGGGGTCGTCCTTAG

>CF-5chanfangzizhuduanweiye20240529

ATGTTGGGGAAATGCTTGACCGCGGGCTACTGCTCGCAATTGCTTTTTTTGTGGTGTATCGTGCCGTCCTGTTTAGTTGCGCTCGTCAACGCTCAAAGAAACAGCAGCTCCCATTTACAGTTGATTTATAACCTGACGATATGTGAGCTGAATGGCACAGATTGGCTAAATGAAACATTTGATTGGGCGGTGGAGACCTTTGTTATTTTTCCTGTGCTGACTCATATTGTCTCCTATGGCGCCCTAACCACCAGCCATTTCCTTGACACAGTCGGCCTGATCACCGTGTCTGCCGCCGGATATTGCCACGGGCGGTATGTTTTGAGCAGCATTTACGCCGTCTGCGCCCTAGCTGCGTTAACTTGCTTCGTCATCAGGCTAACAAAAAATTGTATGTCCTGGCGTTACTCATGCACCAGATACACTAACTTTCTTCTGGACACCAAGGGCAAACTCTATCGTTGGCGGTCTCCTGTCATCGTAGAGAAAGGGGGTAAAGTCGAGGTCGGAGGCCACCTGATCGACCTCAAGAGGGTTGTGCTTGACGGTTCCGCGGCGACTCCTATAACCAAAGTTTCAGCGGAACAATGGGGTCGTCCTTAG

>CF-6baoyuzuzhi20240720

ATGTTGGGGAAATGCTTGACCGCGGGTTACTGCTCATCATTGCTTTTTTTGTGGTGTATCGTGCTATCCTGTTCTGTTGCGCTCGTCAACGCCGACAGCAACACCAGCTCTCATTTACAGTTGATTTATAACTTGACGATATGTGAGCTGAATGGCACGGATTGGTTGAACGAAAGATTTGACTGGGCAGTAGAGACTTTCGTCATTTTTCCTGTTCTGACCCATATTGTCTCTTACGGCGCCCTCACCACTAGCCATTTTCTTGACACGGTCGGCCTGATCACTGTGTCCACCGCTGGGTATTGCCATAAGCGGTATGTATTGAGTAGCATCTATGCTGTCTGTGCCCTGGCCGCGTTGGCTTGCTTCGCCATCAGGTTGGCGAAAAATTGCATGTCCTGGCGCTACTCATGCACCAGATACACCAACTTTCTCCTGGACACTAAAGGCAAACTCTACCGCTGGCGGTCACCCGTCATCATAGAGAAGGGGGGCAAAGTTGATGTGGGGGGTCATTTAATTGACCTCAAGAGAGTCGTGCTTGATGGTTCCGCGGCGACCCCTATAACCAAGATTTCAGCGGAACAATGGGGTCGTCCATAG

>CF-19chanfangzizhuduanweiye20240717

ATGTTGGGGAAATGCTTGACCGCGGGTTACTGCTCATCATTGCTTTTTTTGTGGTGTATCGTGCCATCCTGTTTTGTTGCACTCGTCAACGCCAGCAACAACACCAGCTCTCATTTACAGTTGATTTATAACTTGACGATATGTGAGCTGAATGGCACAGATTGGCTAACAAAAAGGTTTGACTGGGCAGTGGAGACTTTCGTCATCTTTCCTGTACTGACTCATATTGTCTCTTATGGCGCCCTTACCACTAGCCATTTTCTTGACACGGTCGGCCTGCTCACTGTGTCCACTGCTGGTTATTTACACAAGCGGTACGTATTGAGTAGCATCTACGTTGTTTGTGCCCTGGCTGCGTTTGTCTGCTTCGCCATTAGGTTGGCGAAAAATTGCATGTCTTGGCGCTACTCATGCACCAGATATACCAACTTTCTCCTGGACACTAAGGGCAAACTCTATCGCTGGCGGTCACCCGTCATCATAGAGAAGGGGGGCAAAGCTGACGTGGGGGGTCATTTAATTGACCTCAAGAGAGTCGTGCTTGATGGTTCCGCGGCGACCCCTATAACCAAGATTTCAGCGGAACAATGGGGTCGTCCATAG

>CF-12baoyuzuzhi20240717

ATGTTGGGGAAATGCTTGACCGCGGGTTACTGCTCATCATTGCTTTTTTTGTGGTGTATCGTGCTATCCTGTTCTGTTGCGCTCGTCAACGCCGACAGCAACACCAGCTCTCATTTACAGTTGATTTATAACCTGACGATATGTGAGCTGAATGGCACGGATTGGTTGAACAAAAGGTTTGACTGGGCAGTAGAGACTTTCGTCATTTTTCCTGTTCTGACCCATATTGTCTCTTACGGCGCCCTCACCACTAGCCATTTTCTTGACACGGTCGGCCTAATTACTGTGTCCACCGCTGGGTATTACCATAAACGGTATGTATTGAGTAGCATCTATGCTGTCTGTGCCCTGGCCGCGTTGGCTTGCTTCGCCATCAGGTTGGCGAAAAATTGCATGTCCTGGCGCTACTCATGCACCAGATACACCAACTTTCTCCTGGACACTAAAGGCAGACTCTACCGCTGGCGGTCACCCGTCATCATAGAGAAGGGGGGCAAAGTTGATGTGGGGGGTCATTTAATTGACCTCAAGAGAGTCGTGCTTGATGGTTCCGCAGCGACCCCTATAACCAAGATTTCAGCGGAACAATGGGGTCGTCCATAG

>Ergong-xueqing20240801

ATGTTGGGGAAATGCTTGACCGCGGGCTGCTGCTCGCAATTGCCTTTTTTGTGGTGTATCGTGCCGTTCTGTTTAGTTGCGCTCGTCAACGCCGACGACAACAGCAGCTCCCACTTACAGTTGATTTATAACCTAACGATATGTGAGCTGAATGGCACAGATTGGCTGAAAGAACACTTTTCTTGGGCGGTGGAGACTTTCGTTATTTTTCCTGTGTTAACTCATGTTGTCTCTTACGGTGCCCTCACCACTAGCCATTTTCTTGACACGGTCGGTCTTATCACTGTGTCCACCGCCGGGTATCATCGCGGGCGGTATGTACTGAGTAGCATCTACGCTGTCTGCGCCCTAGCTGCGCTTGTTTGCTTCGCCATTAGGTTGGTAAAGAATTGCATGTCCTGGCGCTACTCTTGCACCAGATATACTAATTTTCTTCTGGACACTAAGGGAAAACTCTATCGTTGGCGGTCACCCGTCATCATAGAGAAGGGGGGCAAGGTGGATGTTGGGGGTCATTTAATCGACCTAAAGAGAGTTGTGCTTGAAGGTTCCGCGGCAACCCCTGTAACCAAGATTTCAGCGGAACAATGGGGTCGTCCATAG

>Ergong-xueqing20241111

ATGTTGGGGAAATGCTTGACCGCGGGTTATTGCTCGCAATCGCTTTTTTTGTGGTGTATCGTGCCGTTCTGTTTTGCTGTGCTCGTCAACGCCAGCAACAACAGCAGCTCCCACTTACAGTTGATTTATAACCTGACGATATGTGAGCTGAATGGCACAGATTGGCTAGATAAAAGTTTTGATTGGGCAGTGGAGACCTTTGTTATTTTCCCTGTGTTGACTCATATTGTCTCCTATGGCGCCCTAACTACCAGCCATTTCCTTGACACAGTTGGCCTGATCACCGTGTCTACCGCTGGGTATTATCACGGGCGGTATGTCCTGAGCAGCATTTACGCCGTCTGCGCCCTAGCTGCGTTAACTTGCTTTGTCATCAGGCTAACAAAAAATTGCATGTCCTGGCGTTACTCATGCACCAGATACACTAACTTTCTTCTGGACACCAAGGGCAAACTCTATCGTTGGCGGTCTCCCGTCATCGTGGAGAAAGGGGGTAAAGTTGAGGTCGAAGGCCACCTGATCGACCTCAAGAGAGTTGTGCTTGACGGTTCCGCGGCGACCCCTATAACCAAAGTTTCAGCGGAACAATGGGGTCGTCCTTAG

>CF-15chanfangduanweiye20240924

ATGTTGGGGAAATGCTTGACCGCGGGCTGCTGCTCGCAATTGCCTTTTTTGTGGTGTATCGTGCCGTTCTGTTTAGTTGCGCTCGTCAACGCCAACGACAACAGCAGCTCCCACTTACAGTTGATTTATAACCTAACGATATGTGAGCTGAATGGCACAGATTGGCTGAAAGAACACTTTTCTTGGGCGGTGGAGACTTTCGTTATTTTTCCTGTGTTAACTCATGTTGTCTCTTACGGTGCCCTCACCACTAGCCATTTTCTTGACACGGTCGGTCTTATCACTGTGTCCACCGCCGGGTATTATCATGGGCGGTATGTACTGAGTAGCATCTACGCTGTCTGCGCCCTAGCTGCGCTTGTTTGCTTCGCCATTAGGTTGGTAAAGAATTGCATGTCCTGGCGCTACTCTTGCACCAGATATACTAATTTTCTTCTGGACACTAAGGGAAAACTCTATCGTTGGCGGTCACCCGTCATCATAGAGAAGGGGGGCAAGGTGGATGTTGGGGGTCATTTAATCGACCTAAAGAGAGTTGTGCTTGAAGGTTCCGCGGCAACCCCTGTAACCAAGATTTCAGCGGAACAATGGGGTCGTCCATAG

>CF-21chanfangduanweiye20240920

ATGTTGGGGAAATGCTTGACCGCGGGTTATTGCTCACAATCGCTTTTTTTGTGGTGTATCGTGCCATTCTGTTTTGCTGTGCTCGTCAACGCCAGCGCCAACAGCAGCTCCCACTTACAGTTGATCTATAACCTGACGATATGTGAGCTGAATGGCACAGACTGGTTGGACCAAAAATTTGACTGGGCAGTAGAGACTTTCGTCATTTTTCCTGTCTTGACCCATATTGTCTCTTACGGCGCCCTTACCACTAGCCATTTTCTTGACACGGTCGGCCTAATCACCGTATCCACCGCTGGGTATTATCATAAGCGGTATGTATTGAGTAGCATCTACGCTGTCTGTGCCCTGGCCGCGCTGGTTTGCTTCGCCATTAGGTTGGCAAAAAATTGTATGTCCTGGCGCTACTCATTCACTAGATATACTAATTTTCTTCTGGACTCTAAGGGCAAACTCTACCGTTGGCGGTCACCCGTCATCATAGAGAAAGAGGGCAAAGTTGATGTGGGGGGTCAGTTAATCGACCTCAAGAGAGTTGTGCTTGACGGTTCCGCGGCGACCCCTGTAACCAAGATTTCAGCGGAACAATGGGGTCGTCCATAG

>CF-5peihuaimuzhuzuzhi20240920

ATGTTGGGGAAATGCTTGACCGCGGGTTACTGCTCATCATTGCTTTTTTTGTGGTGTATCGTGCTATCCTGTTCTGTTGCGCTCGTCAACGCCGACAGCAACACCAGCTCTCATTTACAGTTGATTTATAACTTGACGATATGTGAGCTGAATGGCACGGATTGGTTGAACGAAAGATTTGACTGGGCAGTAGAGACTTTCGTCATTTTTCCTGTTCTGACCCATATTGTCTCTTACGGCGCCCTCACCACTAGCCATTTTCTTGACACGGTCGGCCTAATCACTGTGTCCACCGCTGGGTATTGCCATAAGCGGTATGTATTGAGTAGCATCTATGCTGTCTGTGCCCTGGCCGCGTTGGCTTGCTTCGCCATCAGGTTGGCGAAAAATTGCATGTCCTGGCGCTACTCATGCACCAGATACACCAACTTTCTCCTGGACACTAAAGGCAAACTCTACCGCTGGCGGTCACCCGTCATCATAGAGAAGGGGGGCAAAGTTGATGTGGGGGGTCATTTAATTGACCTCAAGAGAGTCGTGCTTGATGGTTCCGCGGCGACCCCTATAACCAAGATTTCAGCGGAACAATGGGGTCGTCCATAG

>CF-25peihuaimuzhuzuzhi20241011

ATGTTGGGGAA-TGCTTGACCGCGGGCTGCTGCTCGCAATTGCTTTTTTTGTGGTGTATCGTGCCGTTCTGTTTAGTTTCGCTCGTCAACGCCAGCAACAGCAGCAGCTCCCATTTACAGTTGATTTATAACCTGACGATATGTGAGCTGAATGGCACAGATTGGCTAAATCGCACTTTTGATTGGGCAGTGGAGACCTTCGTTATTTTTCCTGTGTTGACTCATATCGTCTCCTATGGCGCCCTAACCACCAGCCACTTCCTTGACACAGTCGGCCTGATCACCGTGTCTACTGCTGGATATTGCCACGGGCGGTATGTCTTGAGCAGTATTTACGCCGTCTGCGCCTTAGCTGCGCTAATTTGCTTCGTCATCAGGCTAACAAAAAATTGCATGTCCTGGCGTTACTCATGCACCAGATACACTAATTTTCTTCTGGACACCAAGGGAAAACTCTATCGTTGGCGGTCTCCCGTCATCGTGGAAAAAGGGGGTAAAGTTGAGGTCGAAGGCCACCTCATCGACCTCAAGAAGGTTGTGCTTGATGGTTCCGCGGCGACCCCTATAACCAAAGTTTCAGCGGAACAATGGGGTCGTCCTTAG

>CF-7peihuaimuzhuzuzhi20241011

ATGTTGGGGAAATGCTTGACCGCGGGCTGCTGCTCGCAATTGCTTTTTTTGTGGTGTATCGTGCCGTTCTGTTTAGTTTCGCTCGTCAACGCCAGCAACAGCAGCAGCTCCCATTTACAGTTGATTTATAACCTGACGATATGTGAGCTGAATGGCACAGATTGGCTAAATGGCAATTTTAATTGGGCAGTGGAGACCTTCGTTATTTTTCCTGTGTTGACCCATATCGTCTCCTATGGCGCCCTAACCACCAGCCACTTCCTTGACACAGTCGGCCTGGTCACCGTGTCTACTGCTGGATATTACCACGGGCGGTATGTCTTGAGCAGTATTTACGCCGTCTGTGCCTTAGCTGCGCTAATTTGCTTCGTCATCAGGCTAACAAAAAATTGCATGTCCTGGCGTTACTCATGCACCAGATACACTAATTTTCTTCTGGACACCAAGGGAAAACTCTATCGTTGGCGGTCTCCCGTCATCGTAGAAAAAGGGGGTAAAGTTGAGGTCGAAGGCCACCTCATCGACCTCAAGAAAGTTGTGCTTGACGGTTCCGCGGCGACCCCTATAACCAAAGTTTCAGCGGAACAATGGGGTCGTCCTTAG

>CF-13peihuaimuzhu20240827

ATGTTGGGGAAATGCTTGACCGCGGGCTGCTGCTCGCAATTGCTTTTTTTGTGGTGTATCGTGCCGTTCTGTTTAGTTTCGCTCGTCAACGCCAGCAACAGCAGCAGCTCCCATTTACAGTTGATTTATAACCTGACGATATGTGAGCTGAATGGCACAGATTGGCTAAATCGCACTTTTGATTGGGCAGTGGAGACCTTCGTTATTTTTCCTGTGTTGACTCATATCGTCTCCTATGGCGCCCTAACCACCAGCCACTTCCTTGACACAGTCGGCCTGATCACCGTGTCTACTGCTGGATATTGCCACGGGCGGTATGTCTTGAGCAGTATTTACGCCGTCTGCGCCTTAGCTGCGCTAATTTGCTTCGTCATCAGGCTAACAAAAAATTGCATGTCCTGGCGTTACTCATGCACCAGATACACTAATTTTCTTCTGGACACCAAGGGAAAACTCTATCGTTGGCGGTCTCCCGTCATCGTGGAAAAAGGGGGTAAAGTTGAGGTCGAAGGCCACCTCATCGACCTCAAGAAAGTTGTGCTTGATGGTTCCGCGGCGACCCCTATAACCAAAGTTTCAGCGGAACAATGGGGTCGTCCTTAG

>CF-23chanfangzizhu20240802

ATGTTGGGGAAATGCTTGACCGCGGGCTGCTGCTCGCAATTTCTTTTTTTGTGGTGTATCGTGCCGTTCTGTTTAGTTGCGCTCGTCAACGCCAACAACAGCAGCAGCTCCCACTTACAGTTGATTTATAACCTGACGATATGTGAGCTGAATGGCACAGATTGGCTAAATAAAAGTTTTGATTGGGCAGTGGAGACCTTTGTTATTTTTCCTGTGTTGACTCATATTGTCTCCTATGGTGCCCTAACCACCAGCCATTTCCTTGATACAGTCGGCCTGATCACCGTGTCTACCGCTGGATATTACCACGGGCGGTATGTCTTGAGCAGCATTTACGCCGTCTGCGCCCTAGCTGCATTAACTTGCTTCGTCATCAGGCTAACAAAAAATTGCATGTCCTGGCGTTACTCATGCACCAGATACACTAACTTTCTTCTGGACACCAAGGGCAAACTCTATCGCTGGCGGTCTCCCGTCATCGTAGAGAAAGGGGGTAAGGTTGAGGTCGAAGGCCACCTGATCGACCTCAAGAGAGTTGTGCTAGACGGTTCCGCGGCGACTCCCATAACCAAAGTTTCAGCGGAGCAATGGGGTCGTCCTTAG

>CF-6baoyuzhuzuzhi20240903

ATGTTGGGGAAATGCTTGACCGCGGGTTACTGCTCATCATTGCTTTTTTTGTGGTGTATCGTGCTATCCTGTTCTGTTGCGCTCGTCAACGCCGACAGCAACACCAGCTCTCATTTACAGTTGATTTATAACTTGACGATATGTGAGCTGAATGGCACGGATTGGTTAAACGAAAGATTTGACTGGGCAGTAGAGACTTTCGTCATTTTTCCTGTTCTGACCCATATTGTCTCTTACGGCGCCCTCACCACTAGCCATTTTCTTGACACGGTCGGCCTGATCACTGTGTCCACCGCTGGGTATTGCCATAAGCGGTATGTATTGAGTAGCATCTATGCTGTCTGTGCCCTGGCCGCGTTGGCTTGCTTCGCCATCAGGTTGGCGAAAAATTGCATGTCCTGGCGCTACTCATGCACCAGATACACCAACTTTCTCCTGGACACTAAAGGCAAACTCTACCGCTGGCGGTCACCCGTCATCATAGAGAAGGGGGGCAAAGTTGATGTGGGGGGTCATTTAATTGACCTCAAGAGAGTCGTGCTTGATGGTTCCGCGGCGACCCCTATAACCAAGATTTCAGCGGAACAATGGGGTCGTCCATAG

>CF-16yufeixueqing20250228

ATGTTGGGGAAATGCTTGACCGCGGGTTATTGCTCACAATCGCTTTTTTTGTGGTGTATCGTGCCATTCTGTTTTGCTGTGCTCGTCAACGCCAGCGACAACAACAGCTCCCACTTACAGTTGATCTATAACCTGACGATATGTGAGCTGAATGGCACAGACTGGTTGAACCAAAAATTTGACTGGGCAGTAGAGACTTTCGTCATTTTTCCTGTCTTGACCCATATTGTCTCTTACGGCGCCCTTACCACTAGCCATTTTCTTGACACGGTCGGCCTAATCACCGTATCCACCGCTGGGTATTATCATAAGCGGTATGTGTTGAGTAGCATCTACGCTGTCTGTGCCCTGGCCGCGTTGGTTTGCTTCGCCATTAGGTTGGCAAAAAATTGCATGTCCTGGCGCTACTCATTCACCAGATATACTAATTTTCTTCTGGACTCTAAGGGCAAACTCTACCGTTGGCGGTCACCCGTCATCATAGAGAAAGAGGGCAAAGTTGATGTGGGGGGTCAGTTAATCGAACTCAAGAGAGTTGTGCTTGATGGTTCCGCGGCGACCCCTGTAACCAAGATTTCAGCGGAACAATGGGGTCGTCCATAG

>CF-1houbeixueqing20250311

ATGTTGGGGAAATGCTTGACCGCGGGCTGCTGCTCGCAATTGCCTTTTTTGTGGTGTATCGTGCCGTTCTGTTTAGTTGCGCTCGTCAACGCCAACGACAACAGCAGCTCCCACTTACAATTGATTTATAACCTAACGATATGTGAGCTGAATGGCACAGATTGGCTGAAAGACCACTTTTCTTGGGCGGTGGAGACTTTCGTTATTTTTCCTGTGTTAACTCATATTGTCTCTTACGGTGCCCTCACCACTAGCCATTTTCTTGACACGGTCGGTCTTATCACTGTGTCCACCGCCGGGTATTATCACGGGCGGTATGTATTGAGTAGCATCTACGCTGTCTGCGCCCTAGCTGCGCTTGTTTGCTTCGCCATTAGGTTGGTAAAGAATTGCATGTCCTGGCGCTACTCTTGCACCAGATATACTAATTTTCTTCTGGACACTAAGGGAAAACTCTATCGTTGGCGGTCACCCGTCATCATAGAGAAGGGAGGCAAGGTGGATGTTGGTGGTCATTTAATCGACCTAAAGAGAGTTGTGCTTGAAGGTTCCGCGGCAACCCCTGTAACCAAGATTTCAGCGGAACAATGGGGTCGTCCATAG

>CF-8yufeixueqing20250317

ATGTTGGGGAAATGCTTGACCGCGGGCTGCTGCTCGCAATTGCTTTTTTTGTGGTGTATCGTGCCGTTCTGTTTAGTTTCGCTCGTCAACGCCAGCAACAGCAGCAGCTCCCATTTACAGTTGATTTATAACCTGACGATATGTGAGCTGAATGGCACAGATTGGCTAAATCGCACTTTTGATTGGGCAGTGGAGACCTTCGTTATTTTTCCTGTGTTGACTCATATCGTCTCCTATGGCGCCCTAACCACCAGCCACTTCCTTGACACAGTCGGCCTGATCACCGTGTCTACTGCTGGATATTACCACGGGCGGTATGTCTTGAGCAGTATTTACGCCGTCTGCGCCTTAGCTGCGCTAATTTGCTTCGTCATCAGGCTAACAAAAAATTGCATGTCCTGGCGTTACTCATGCACCAGATACACTAATTTTCTTCTGGACACCAAGGGAAAACTCTATCGTTGGCGGTCTCCCGTCATCGTGGAAAAAGGGGGTAAAGTTGAGGTCGAAGGCCACCTCATCGACCTCAAGAAAGTTGTGCTTGATGGTTCCGCGGCGACCCCTATAACCAAAGTTACAGCGGAACAATGGGGTCGTCCTTAG

>CF-25erquchanfangduanweiye20250421

ATGTTGGGGAAATGCTTGACCGCGGGCTGCTGCTCGCAATTGCTTTTTTTGTGGTGTATCGTGCCGTTCTGTTTAGTTTCGCTCGTCAACGCCAGCAGCAGCAGCAGCTCCCATTTACAGTTGATTTATAACCTGACGATATGTGAGCTGAATGGCACAGATTGGCTAAATTCCACTTTTGATTGGGCAGTGGAGACCTTCGTTATTTTTCCTGTGTTGACTCATATCGTCTCCTATGGCGCCCTAACCACCAGCCACTTCCTTGACACAGTCGGCCTGATCACCGTGTCTACTGCTGGATATTGCCACGGGCGGTATGTCTTGAGCAGTATTTACGCCGTCTGCGCCTTAGCTGCGCTAATTTGCTTCGTCATCAGGCTAACAAAAAATTGCATGTCCTGGCGTTACTCATGCACCAGATACACTAATTTTCTTCTGGACACCAAAGGAAAACTCTATCGTTGGCGGTCTCCCGTCATCGTGGAGAAAGGGGGTAAAGTTGAGGTCGAAGGCCACCTCATCGACCTCAAGAAAGTTGTGCTTGATGGTTCCGCGGCGACCCCTATAACCAAAGTTTCAGCGGAACAATGGGGTCGTCCTTAG

>CF-9chanfang2dongduanweiye20250430

ATGTTGGGGAAATGCTTGACCGCGGGCTACTGCTCGCAATTGCTTTTTTTGTGGTGTATCGTGCCGTCCTGTTTAGTTGCGCTCGTCAACGCTCAAAGAAACAGCAGCTCCCATTTACAGTTGATTTATAACCTGACGATATGTGAGCTGAATGGCACAGATTGGCTAAATGAAACATTTGATTGGGCGGTGGAGACCTTTGTTATTTTTCCTGTGCTGACTCATATTGTCTCCTATGGCGCCCTAACCACCAGCCATTTCCTTGACACAGTCGGCCTGATCACCGTGTCTGCCGCCGGATATTACCACGGGCGGTATGTTTTGAGCAGCATTTACGCCGTCTGCGCCCTAGCTGCGTCAACTTGCTTCGTCATCAGGCTAACAAAAAATTGTATGTCCTGGCGTTACTCATGCACCAGATACACTAACTTTCTTCTGGACACCAAGGGCAAACTCTATCGTTGGCGGTCTCCTGTCATCGTAGAGAAAGGGGGTAAAGTCGAGGTCGGAGGCCACCTGATCGACCTCAAGAGGGTTGTGCTTGACGGTTCCGCGGCGACTCCTATAACCAAAGTTTCAGCGGAACAATGGGGTCGTCCTTAG

>CF-9chanfang4dongduanweiye20250430

ATGTTGGGGAAATGCTTGACCGCGGGCTGCTGCTCGCAATTGCCTTTTTTGTGGTGTATCGTGCCGTTCTGTTTAGTTGCGCTCGTCAACGCCAACGAAAACAGCAGCTCCCACTTACAATTGATTTATAACCTAACGATATGTGAGCTGAATGGCACAGATTGGCTGAATGAACACTTTTCTTGGGCGGTGGAGACTTTCGTTATTTTTCCTGTGTTAACTCATATTGTCTCTTACGGTGCCCTCACCACTAGCCATTTTCTTGACACGGTCGGTCTTATCACTGTGTCCACCGCCGGGTATTATCACGGGCGGTATGTATTGAGTAGCATCTACGCTGTCTGCGCCCTAGCTGCGCTTGGTTGCTTCGTCATTAGGTTGGTAAAAAATTGCATGTCCTGGCGCTACTCTTGCACCAGATATACTAATTTTCTTCTGGACACTAAGGGAAAACTCTATCGTTGGCGGTCACCCGTCATCATAGAGAAGGGGGGCAAGGTGGATGTTGGTGGTCATTTAATCGACCTAAAGAGAGTTGTGCTTGAAGGTTCCGCGGCAACCCCTGTAACCAAGATTTCAGCGGAACAATGGGGTCGTCCATAG

>CF-16yufeiyiquxueqing20250504

ATGTTGGGGAAATGCTTGACCGCGGGCTGCTGCTCGCAATTGCTTTTTTTGTGGTGTATCGTGCCGTTCTGTTTAGTTTCGCTCGTCAACGCCAGCAACAGCAGCAGCTCCCATTTACAGTTGATTTATAACCTGACGATATGTGAGCTGAATGGCACGGATTGGCTGAATCGCACTTTTGATTGGGCAGTGGAGACCTTCGTTATTTTTCCTGTGTTGACTCATATCGTCTCCTACGGCGCCCTAACCACCAGCCACTTCCTTGACACAGTCGGCCTGATCACCGTGTCTACTGCTGGATATTACCACGGGCGGTATGTCTTGAGCAGTATTTACGCCGTCTGCGCCTTAGCTGCGCTAATTTGCTTCGTCATCAGGCTAACAAAAAATTGCATGTCCTGGCGTTACTCATGCACCAGATACACTAATTTTCTTCTGGACACCAAGGGAAAACTCTATCGTTGGCGGTCTCCCGTCATCGTGGAAAAAGGGGGTAAAGTTGAGGTCGAAGGCCACCTCATCGACCTCAAGAAAGTTGTGCTTGATGGTTCCGCGGCGACCCCTATAACCAAAGTTACAGCGGAACAATGGGGTCGTCCTTAG

>CF-25GDUxueqing20250504

ATGTTGGGGAAATGCTTGACCGCGGGCTGCTGCTCGCAATTGCCTTTTTTGTGGTGTATCGTGCCGTTCTGTTTAGTTGCGCTCGTCAACGCCAACGACAACAACAGCTCCCACTTACAATTGATTTATAACCTAACGATATGTGAGCTGAATGGCACAGATTGGCTGAAAGACCACTTTTCTTGGGCGGTGGAGACTTTCGTTATTTTTCCTGTGTTAACTCATATTGTCTCTTACGGTGCCCTCACCACTAGCCATTTTCTTGACACGGTCGGTCTTATCACTGTGTCCACCGCCGGGTATTATCACGGGCGGTATGTATTGAGTAGCATCTACGCTGTCTGCGCCCTAGCTGCGCTTGTTTGCTTCGCCATTAGGTTGGTAAAGAATTGCATGTCCTGGCGCTACTCTTGCACCAGATATACTAATTTTCTTCTGGACACTAAGGGAAAACTCTATCGTTGGCGGTCACCCGTCATCATAGAGAAGGGGGGCAAGGTGGATGTTGGTGGTCATTTAATCGACCTAAAGAGAGTTGTGCTTGAAGGTTCCGCGGCAACCCCTGTAACCAAGATTTCAGCGGAACAATGGGGTCGTCCATAG

>CF-5chanfangtaiyixue20250507

ATGTTGGGGAAATGCTTGACCGCGGGCTGCTGCTCGCAATTGCCTTTTTTGTGGTGTATCGTGCCGTTCTGTTTAGTTGCGCTCGTCAACGCCAACAACAACAGCAGCTCCCACTTACAGTTGATTTATAGACTAACGATATGTGAGCTGAATGGCACAGACTGGCTGGATAAACATTTTTCTTGGGCAGTGGAGACTTTCGTTATCTTTCCTGTGTTAACTCATATTGTCTCTTACGGTGCCCTCACCACTAGCCATTTTCTTGACACGGTCGGCCTAATCGCTGTGTCCACCGCCGGGTATTATCACGGGCGGCATGTACTGAGTAGCATCTACGCTGTCTGCGCCCTAGCTGCACTTGTTTGCTTCGCCATTAGGTTGGTAAAAAATTGCATGTCCTGGCGCTACTCTTGCACCAGATATACTAATTTTCTTCTGGACACTAAGGGAAAACTCTATCGCTGGCGGTCACCCGTCATCATAGAGAAGGGGGGCAAGGTGGATGTTGGGGGTCATTTAATTGACCTAAAGAGAGTTGTGCTTGAAGGTTCCGCGGCGACTCCTGTAACCAAGATTTCAGCGGAACAATGGGGTCGTCCATAG

>CF-24nanqubaoyukoushizi20250509

ATGTTGGGGAAGTGCTTGACCGCGTGCTGTTGCTCGCGATTGCTTTTTTTGTGGTGTATCGTGCCGTCCTATCTTGCTGTGCTCGTCAACGCCAGCAACAACAACAGCTCTCATATTCAGTTGATTTATAACTTAACGCTATGTGAGCTGAATGGCACAGATTGGCTGGCACAAAAATTTGACTGGGCAGTGGAGACTTTCGTCATCTTCCCCGTGTTGACTCACATTGTTTCCTATGCGGCACTCACCACCAGCCATTTCCTTGACACAGTTGGTCTGGCCACTGTGTCCACCGCCGGATATTATCACGGGCGGTATGTCTTGAGTAGCATTTACGCAGTCTGTGCTCTGGCTGCGCTGATTTGCTTTGTCATTAGGCTTGCGAAGAACTGCATGTCCTGGCGCTACTCTTGTACCAGATATACCAACTTCCTTCTGGACACTAAGGGCAAACTCTATCGTTGGCGGTCGCCCGTCATTGTGGAGAAAGGGGGTAAGGTTGAGGTCGAAGGTCACCTGATCGACCTCAAGAGAGTTGTGCTTGATGGTTCCGCGGCAACCCCTTTAACCAGAGTTTCAGCGGAACGATGGGGTCGTCTCTAG

>CF-8erquyufeixueqing20250510

ATGTGGGGGAAATGCTTGACCGCGGGCTGCTGCTCGCAATTGCCTTTTTTGTGGTGTATCGTGCCGTTCTGTTTAGTTGCGCTCGTCAACGCCAACGACAACAGCAGCTCCCACTTACAGTTGATTTATAACCTAACAATATGTGAGCTGAATGGCACAGATTGGCTGAAAGAACACTTTTCTTGGGCGGTGGAGACTTTCGTTATTTTTCCTGTGTTAACTCATGTTGTCTCTTACGGTGCCCTCACCACTAGCCATTTTCTTGACACGGTCGGTCTTATCACTGTGTCCACCGCCGGGTATTATCACGGGCGGTATGTACTGAGTAGTATTTACGCTGTCTGCGCCCTAGCTGCGCTTGTTTGCTTCGCCATTAGGTTGGTAAAGAATTGCATGTCCTGGCGCTACTCTTGCACCAGATATACTAATTTTCTTCTGGACACTAAGGGAAAACTCTATCGTTGGCGGTCACCCGTCATCATAGAGAAGGGGGGCAAGGTGGATGTCGGGGGTCATTTAATCGACCTAAAGAGAGTTGTGCTTGAAGGTTCCGCGGCAACCCCTGTAACCAAGATTTCAGCGGAACAATGGGGTCGTCCATAG

>CF-25yiquchanfangtaiyixue20250513

ATGTTGGGGAAATGCTTGACCGCGGGCTGCTGCTCGCAATTGCCTTTTTTGTGGTGTATCGTGCCGTTCTGTTTAGTTGCGCTCGTCAACGCCAACGACAACAGCAGCTCCCACTTACAATTGATTTATAACCTAACGATATGTGAGCTGAATGGCACAGATTGGCTGAAAGACCACTTTTCTTGGGCGGTGGAGACTTTCGTTATTTTTCCTGTGTTAACTCATATTGTCTCTTACGGTGCCCTCACCACTAGCCATTTTCTTGACACGGTCGGTCTTATCACTGTGTCCACCGCCGGGTATTATCACGGGCGGTATGTATTGAGTAGCATCTACGCTGTCTGCGCCCTAGCTGCGCTTGTTTGCTTCGCCATTAGGTTGGTAAAGAATTGCATGTCCTGGCGCTACTCTTGCACCAGATATACTAATTTTCTTCTGGACACTAAGGGAAAACTCTATCGTTGGCGGTCATCCGTCATCATAGAGAAGGGGGGCAAGGTGGATGTTGGTGGTCATTTAATCGACCTAAAGAGAGTTGTGCTTGAAGGTTCCGCGGCAACCCCTGTAACCAAGATTTCAGCGGAACAATGGGGTCGTCCATAG

>CF-25GDUxueqing20250511

ATGTTGGGGAAATGCTTGACCGCGGGCTGCTGCTCGCAATTGCCTTTTTTGTGGTGTATCGTGCCGTTCTGTTTAGTTGCGCTCGTCAACGCCAACGACAACAACAGCTCCCACTTACAATTGATTTATAACCTAACGATATGTGAGCTGAATGGCACAGATTGGCTGAAAGACCACTTTTCTTGGGCGGTGGAGACTTTCGTTATTTTTCCTGTGTTAACTCATATTGTCTCTTACGGTGCCCTCACCACTAGCCATTTTCTTGACACGGTCGGTCTTATCACTGTGTCCACCGCCGGGTATTATCACGGGCGGTATGTATTGAGTAGCATCTACGCTGTCTGCGCCCTAGCTGCGCTTGTTTGCTTCGCCATTAGGTTGGTAAAGAATTGCATGTCCTGGCGCTACTCTTGCACCAGATATACTAATTTTCTTCTGGACACTAAGGGAAAACTCTATCGTTGGCGGTCACCCGTCATCATAGAGAAGGGGGGCAAGGTGGATGTTGGTGGTCATTTAATCGACCTAAAGAGAGTTGTGCTTGAAGGTTCCGCGGCAACCCCTGTAACCAAGATTTCAGCGGAACAATGGGGTCGTCCATAG

>CF-7chanfangduanweiye20250512

ATGTTGGGGAAATGCTTGACCGCGGGCTGCTGCTCGCAATTGCTTTTTTTGTGGTGTATCGTGCCGTTCTGTTTAGTTTCGCTCGTCAACGCCAGCAACAGCAGCAGCTCCCATTTACAGTTGATTTATAACCTGACGATATGTGAGCTGAATGGCACAGATTGGCTAAATCGCACTTTTGATTGGGCAGTGGAGACCTTCGTCATTTTTCCTGTGTTGACCCATATCGTCTCCTATGGCGCCCTAACCACCAGCCACTTCCTTGACACAGTCGGCCTGATCACCGTGTCTATTGCTGGATATTACCACGGGCGGTATGTCTTGAGCAGTATTTACGCCGTCTGCGCCTTAGCTGCGCTAATTTGCTTCGTCATCAGGCTAACAAAAAATTGCATGTCCTGGCGTTACTCATGCACCAGATACACTAATTTTCTTCTGGACACCAAGGGAAAACTCTATCGTTGGCGGTCTCCCGTCATCGTGGAAAAAGGGGGTAAAGTTGAGGTCGAAGGCCACCTCATCGACCTCAAGAAAGTTGTGCTTGATGGTTCCGCGGCGACCCCTATAACCAAAGTTACAGCGGAACAATGGGGTCGTCCTTAG

>CF-5chanfangduanweiye20250512

ATGTTGGGGAAATGCTTGACCGCGGGCTGCTGCTCGCAATTGCCTTTTTTGTGGTGTATCGTGCCGTTCTGTTTAGTTGCGCTCGTCAACGCCAGCGACTACAGCAGCTCCCACTTACAATTGATTTATAACCTAACGATATGTGAGCTGAATGGCACAGATTGGCTGAAAGACCACTTTTCTTGGGCGGTGGAGACTTTCGTTATTTTTCCTGTGTTAACTCATATTGTCTCTTACGGTGCCCTCACCACTAGCCATTTTCTTGACACGGTCGGTCTTATCACTGTGTCCACCGCCGGGTATTATCACGGGCGGTATGTATTGAGTAGCATCTACGCTGTCTGCGCCCTAGCTGCGCTTGTTTGCTTCGCCATTAGGTTGGTAAAGAATTGCATGTCCTGGCGCTACTCTTGCACCAGATATACTAATTTTCTTCTGGACACTAAGGGAAAACTCTATCGTTGGCGGTCACCCGTCATCATAGAGAAGGGGGGCAAGGTGGATGTTGGTGGTCATTTAATCGACCTAAAGAGAGTTGTGCTTGAAGGTTCCGCGGCAACCCCTGTAACCAAGATTTCGGCGGAACAATGGGGTCGTCCATAG

>CF-22beiqubaoyuzuzhi20250512

ATGTTGGGGAAATGCTTGACCGCGGGCTGCTGCTCGCAATCGCTTTTTTTGTGGTGTATCGTGCCGTTCTGTTTAGTTGCGTTCGTCAACGCCAACAACAGCAGCAGCTCCCACTTACAGTTGATTTATGACCTAACGATATGTGAGCTGAATGGCACAGATTGGCTGGATAAACATTTTTCTTGGGCAGTGGAGACTTTCGTTATTTTTCCTGTATTAACTCACATTGTCTCTTACGGTGCCCTCACCACTAGCCATTTTCTTGACACGGTCGGCCTAATCACTGTGTCCACCGCCGGGTATTGTCACGGGCGGTATGTACTGAGTAGCATCTACGCTGTCTGCGCCCTAGCTGCGCTTGTTTGCTTCGCCATTAGGTTGGTAAAAAATTGCATGTCCTGGCGCTACTCTTGCACTAGATATACTAATTTCCTTCTGGACACTAAGGGAAAACTCTATCGTTGGCGGTCACCCGTCATCATAGAGAAAGGGGGCAAGGTGGATGTTGGGGGTCATTTAATTGACCTGAAGAGAGTTGTGCTTGAAGGTTCCGCGGCGACCCCTGTAACCAAGATTTCAGCGGAACAATGGGGTCGTCCATAG

>CF-18beiquyufeizuzhi20250515

ATGTTGGGGAAATGCTTGACCGCGGGTTACTGCTCATCATTGCTTTTTTTGTGGTGTATCGTGCTATCCTGTTCTGTTGCGCTCGTCAACGCCAACAGCAACACCAGCTCTCATTTACAGTTGATTTATAACCTGACGATATGTGAGCTGAATGGCACGGATTGGTTGAACAAAAGATTTGACTGGGCAGTAGAGACTTTCGTCATTTTTCCTGTTCTGACCCATATTGTCTCGTACGGTGCCCTCACCACTAGCCATTTTCTTGACACGGTCGGCCTAATCACTGTGTCCACCGCTGGGTATTACCATAAGCGGTATGTATTGAGCAGCATCTATGCTGTCTGTGCCCTGGCTGCGTTGGCTTGCTTCGCCATCAGGTTGGCGAAAAATTGCATGTCCTGGCGCTACTCATGCACCAGATACACCAACTTTCTCCTTGACACTAAAGGCAAACTCTACCGCTGGCGGTCACCCGTCATCATAGAGAAGGGGGGCAAAGTTGATGTGGGGGGTCATTTAATTGACCTCAAGAGAGTCGTGCTTGATGGTTCCGCAGCGACCCCTATAACCAAGATTTCAGCGGAACAATGGGGTCGTCCATAG

>CF-9chanfang3dongtaiyixue20250519

ATGTTGGGGAAATGCTTGACCGCGGGTTGTTGCTCGCAATTGCTTTTTTTGTGGTGTATCGTGCCATCCTGTTTTGTTGCGCTCGTCAGCGCCAACA---ACAGCAGCTCCCACTTACAGTTGATTTATAACCTGACGATATGTGAGCTGAATGGCACAGATTGGCTAAACGAAAATTTTTCTTGGGCAGTGGAGACTTTCGTCATCTTTCCTGTGTTGACTCACATTGTTTCTTACGGTGCCCTCACCACTAGCCATTTTCTTGACACGGTCGCCCTGATCACTGTGTCCACCGCCGGATATTATCACCGGCGGTATGTATTGAGTAGCATCTATGCTGTCTGTGCCCTGGCTGCGTTGTCTTGCTTTGCCATCAGATTGACAAAAAATTGCATGTCCTGGCGCTACTCATGTACTAGATACACCCATTTTCTTCTGGACACTAAAGGGAGACTCTATCGTTGGCGGTCACCCGTCATCATAGAGAGAGGGGGTAAAGCTGATGTGGGAGGTCACTTAATTGATCTCAAGAGAGTTGTGCTTGATGGTTCCGCGGCAACCCCCGTAACCAAGATTTCAGCGGAACAATGGGGTCGTCCATAG

>CF-2nanquyufeizuzhi20250520

ATGTTGGGGAAATGCTTGACCGCGGGTTGTTGCTGGCAATTGCTTTTTTTGTGGTGTATCGTGCCATCTTGTTTTGTTGCGCTCGTCAGCGCCAACA---ACAGCAGCTCCCACTTACAGTTGATTTATAACCTGACGATATGTGAGCTGAATGGCACAGATTGGCTAAACGAAAATTTTTCTTGGGCAGTGGAGACTTTCGTCATCTTTCCTGTGTTGACTCACATTGTTTCTTACGGTGCCCTCACCACTAGCCATTTTCTTGACACGGTCGCCCTGATCACTGTGTCCACCGCCGGATATTATCACCGGCGGTATGTATTGAGTAGCATCTATGCTGTCTGTGCCCTGGCTGCGTTGTCTTGCTTTGCCATCAGATTGACAAAAAATTGCATGTCCTGGCGCTACTCATGTACTAGATACACCCATTTTCTTCTGGACACTAAAGGGAGACTCTATCGTTGGCGGTCACCCGTCATCATAGAGAGAGGGGGTAAAGCTGATGTGGGAGGTCACTTAATTGACCTCAAGAGAGTTGTGCTTGATGGTTCCGCGGCAACCCCCGTAACCAAGATTTCAGCGGAACAATGGGGTCGTCCATAG

>CF-2beiquyufeizuzhi20250520

ATGTTGGGGAAGTGCTTGACCGCGTGCTGTTGCTCGCGATTGCTTTTTTTGTGGTGTATCGTGCCGTCCTATCTTGCTGTGCTCGTCAACGCCAGCAACAACAACAGCTCTCATATTCAGTTGATTTATAACTTAACGCTATGTGAGCTGAATGGCACAGATTGGCTGGCAAAAAATTTTGACTGGGCAGTGGAGACTTTTGTCATCTTCCCCGTGTTGACTCACATTGTTTCCTATGGGGCACTCACCACCAGCCATTTCCTTGACACAATTGGTCTGGCCACTGTGTCCACCGCCGGATATTATCACAGGCGGTATGTCTTGAGTAGCATTTACGCAGTCTGTGCTTTGGCTGCGCTGCTTTGCTTTGTCATTAGGCTTGCGAAGAACTGCATGTCCTGGCGCTACTCTTGTACCAGATATACCAACTTCCTTCTGGACACTAAGGGCAAACTCTATCGTTGGCGGTCGCCCGTCATTGTGGAGAAAGGGGGTAAGGTTGAGGTCGAAGGTCACCTGATCGACCTCAAGAGAGTTGTGCTTGATGGTTCCGCGGCAACCCCTTTAACCAGAGTTTCAGCGGAACGATGGGGTCGTCTCTAG

>CF-4yiquyufeizuzhi20250520

ATGTTGGGGAAATGCTTGACCGCGGGCTGCTGCTCGCAATTGCTTTTTTTGTGGTGTATCGTGCCTTTCTGTTTAGTTGCGCTCGTCAACGCCAACGACAACAGCAGCTCCCACTTACAGTTGATTTATAACCTAACGATATGTGAGCTGAATGGCACAGATTGGCTGAATGAACACTTTTCTTGGGCAGTGGAGACTTTCGTTATTTTTCCTGTGTTAACTCATATTGTCTCTTACGGTGCCCTCACCACTAGCCATTTTCTTGACACGGCCGGTCTGATCACTGTGTCCACCGCCGGGTATTATCACGGGCGGTATGTGCTGAGTAGCATCTACGCTGTCTGCGCCCTAGCTGCGCTTGTTTGCTTCGCCATTAGGTTGGTGAAAAATTGCATGTCCTGGCGCTACTCTTGCACCAGATATACTAATTTTCTTCTGGATACCAAGGGAAAACTCTATCGTTGGCGGTCACCCGTCATCATAGAGAAGGGGGGCAAGGTGGATGTTGGGGGTCATTTAATTGACCTGAAGAGAGTTGTGCTTGAGGGTTCCGCGGCGACCCCTGTAACCAAGATTTCAGCGGAACAATGGGGTCGTCCATAG

>CF-7gongzhushexueqing20250520

ATGTTGGGGAAATGCTTGACCGCGGGCTGCTGCTCGCAATTGCTTTTTTTGTGGTGTATCGTGCCGTTCTGTTTAGTTTCGCTCGTCAACGCCAGCAACAACAGCAGCTCCCATTTACAGTTGATTTATAACCTGACGATATGTGAGCTGAATGGCACAGATTGGCTAAATCGCACTTTTGATTGGGCAGTGGAGACCTTCGTTATTTTTCCTGTGTTGACTCATATCGTCTCCTATGGCGCCCTAACCACCAGCCACTTCCTTGACACAGTCGGCCTGATCACCGTGTCTACTGCTGGATATTACCACGGGCGGTATGTCTTGAGCAGTATTTACGCCGTCTGCGCCTTAGCTGCGCTAATCTGCTTCGTCATCAGGCTAACAAAAAATTGCATGTCCTGGCGTTACTCATGCACCAGATACACTAATTTTCTTCTGGACACCAAGGGAAAACTCTATCGTTGGCGGTCTCCCGTCATCGTGGAAAAAGGGGGTAAAGTTGAGGTCGAAGGCCACCTCATCGACCTCAAGAAAGTTGTGCTTGATGGTTCCGCGGCGACCCCTATAACCAAAGTTACAGCGGAACAATGGGGTCGTCCTTAG

>CF-18nanquyufeizuzhi20250520

ATGTTGGGGAAATGCTTGACCGCGGGCTGCTGCTCGCAATTGCTTTTTTTGTGGTGTATCGTGCCGTTCTGTTTAGTTGCGTTCGTCAACGCCAACAACAGCAGCAGCTCCCACCTACAGTTGATTTATAACCTAACGATATGTGAGCTGAATGGCACAGATTGGCTGAATAAACATTTTTCTTGGGCAGTGGAGACTTTCGTTATTTTTCCTGTGTTAACTCACATTGTCTCTTACGGTGCCCTCACCACTAGCCATTTTCTTGACACGGTCGGCCTGATCACTGTGTCCACCGCCGGGTATCGTCACGGGCGGTATGTATTGAGTAGCATCTACGCTGTCTGCGCCCTAGCTGCGCTTGTTTGCTTCGCCATTAGGTTGGTAAAAAATTGCATGTCCTGGCGCTACTCTTGCACCAGACATACTAATTTCCTTCTGGACACTAAGGGAAAACTCTATCGTTGGCGGTCACCCGTCATCATAGAGAAAGGGGGCAAGGTGGATGTTGGGGGTCATTTAATTGACCTGAAAAGAGTTGTGCTTGAAGGTTCCGCGGCGACTCCTGTAACCAAGATTTCAGCGGAACAATGGGGTCGTCCATAG

>CF-25yiquchanfangduanweiye20250522

ATGTTGGGGAAATGCTTGACCGCGGGCTGCTGCTCGCAATTGCCTTTTTTGTGGTGTATCGTGCCGTTCTGTTTAGTTGCGCTCGTCAACGCCAACGACAACAGCAGCTCCCACTTACAATTGATTTATAACCTAACGATATGTGAGCTGAATGGCACAGATTGGCTGAAAGACCACTTTTCTTGGGCGGTGGAGACTTTCGTTATTTTTCCTGTGTTAACTCATATTGTCTCTTACGGTGCCCTCACCACTAGCCATTTTCTTGACACGGTCGGTCTTATCACTGTGTCCACCGCCGGGTATTATCACGGGCGGTATGTATTGAGTAGCATCTACGCTGTCTGCGCCCTAGCTGCGCTTGTTTGCTTCGCCATTAGGTTGGTAAAGAATTGCATGTCCTGGCGCTACTCTTGCACCAGATATACTAATTTTCTTCTGGACACTAAGGGAAAACTCTATCGTTGGCGGTCATCCGTCATCATAGAGAAGGGGGGCAAGGTGGATGTTGGTGGTCATTTAATCGACCTAAAGAGAGTTGTGCTTGAAGGTTCCGCGGCAACCCCTGTAACCAAGATTTCAGCGGAACAATGGGGTCGTCCATAG

>CF-10baoyuzuzhi20250523

ATGTTGGAGAAATGCTTGACCGCGGGCTGTTGCTCGCAATTGCTTTCTTTGTGGTGTATCGTGCCGTTCTGTTTTGCTGTGCTCGCCAACGCCAGCAGCGACAGCAGCTCCCATCTACAGCTGATTTACAACTTGACGCTATGTGAGCTGAATGGCACAGATTGGCTAGCTAACAAATTTGATTGGGCAGTGGAGAGTTTTGTCATCTTTCCCGTTTTGACTCACATTGTCTCCTATGGTGCCCTCACTACCAGCCATTTCCTTGACACAGTCGCTTTAGTCACTGTGTCTACCGCCGGGTTTGTTCACGGGCGGTATGTCCTAAGTAGCATCTACGCGGTCTGTGCCCTGGCTGCGTTGACTTGCTTCGTCATTAGGTTTGCAAAGAATTGCATGTCCTGGCGCTACGCGTGTACCAGATATACCAACTTTCTTCTGGACACTAAGGGCGGACTCTATCGTTGGCGGTCGCCTGTCATCATAGAGAAAAGGGGCAAAGTTGAGGTCGAAGGTCATCTGATCGACCTCAAAAGAGTTGTGCTTGATGGTTCCGTGGCAACCCCTATAACCAGAGTTTCAGCGGAACAATGGGGTCGTCCTTAG

>CF-26beiqubaoyukouqiangye20250526

ATGTTGGGGAGATGCTTGACCGCGGGTTGCTGCTCATCATTGCTTTTTTTGTGGTGTATCGTGCTATCTTGTTCTGTTGCGCTCGTCAACGCCGACAGCAACACCAGCTCTCATTTACAGTTGATTTATAACCTGACGATATGTGAGCTGAATGGCACGGATTGGTTGGACAAAAGATTTAACTGGGCAGTAGAGACTTTCGTCATTTTTCCTGTTCTAACCCATATTGTCTCTTACGGCGCCCTCACCACTAGCCATTTTCTTGACACGGTCGGCCTAATTACTGTGTCCACCGCTGGGTATTACCATAAGCGGTATGTATTGAGTAGCATCTATGCTGTCTGTGCCCTGGCCGCGTTGGCTTGCTTCGCCATCAGGTTGGCGAAAAATTGCATGTCCTGGCGCTACTCATGCACCAGATACACCAACTTTCTCCTGGACACTAAAGGCAGACTCTACCGCTGGCGGTCACCCGTCATCATAGAGAAGGGGGGTAAAGTTGATGTGGGGGGTCATTTAGTTGACCTCAAGAGAGTCGTGCTTGATGGTTCCGCGGCGACCCCTATAACCAAGATTTCAGCGGAACAATGGGGTCGTCCATAG

>CF-12baoyuzuzhi20250527

ATGTCGGGGAAATGCTTGACCGCGGGCTGTTGCTCGCAATTGCCTTTTTTGTGGTATATCGTGCCGTTCTGTTTTGTTGTGCTCGTCAACGCCAACAACAGCAGCAGCTCCCATTTACAGTTGATTTATAACCTGACGATATGTGAGCTGAATGGCACAGATTGGCTAAATAAAAGTTTTGATTGGGCAGTGGAGACCTTTGTTATTTTCCCTGTGTTGACTCATATTGTCTCCTATGGCGCCCTAACCACCAGCCATTTCCTTGACACAGTTGGCCTGATCACCGTGTCTACCGCTGGGTATTACCACGGGCGGTATGTCCTGAGCAGCATTTACGCCGTCTGCGCCCTAGCTGCGTTAACTTGCTTTGTCATCAGGCTAACAAAAAATTGCATGTCCTGGCGTTACTCATGCACCAGATACACTAACTTTCTTCTGGACACCAAGGGCAAACTCTATCGTTGGCGGTCTCCCGTCATCGTTGAGAAAGGGGGTAAAGTTGAGGTCGCAGGCCACCTGATCGACCTCAAGAGAGTTGTGCTTGACGGTTCCGCGGCAACCCCTATAACCAAAGTTTCAGCGGAACAATGGGGTCGTCCTTAG

>CF-15peihuai2dongzuzhi20250527

ATGTCGGGGAAATGCTTGACCGCGGGCTGCTGCTCGCAATTGCTTTTTTTGTGGTGTATCGTGCCGTTCTGTTTAGTTTCGCTCGTCAACGCCAGCAACAGCAGCAGCTCCCATTTACAGTTGATTTATAACCTGACGATATGTGAGCTGAATGGCACGGATTGGCTGAATCGCACTTTTGATTGGGCAGTGGAGACCTTCGTTATTTTTCCTGTGTTGACTCATATCGTCTCCTACGGCGCCCTAACCACCAGCCACTTCCTTGACACAGTCGGCCTGATCACCGTGTCTACTGCTGGATATTACCACGGGCGGTATGTCTTGAGCAGTATTTACGCCGTCTGCGCCTTAGCTGCGCTAATTTGCTTCGTCATCAGGCTAACAAAAAATTGCATGTCCTGGCGTTACTCATGCACCAGATACACTAATTTTCTTCTGGACACCAAGGGAAAACTCTATCGTTGGCGGTCTCCCGTCATCGTGGAAAAAGGGGGTAAAGTTGAGGTCGAAGGCCACCTCATCGACCTCAAGAAAGTTGTGCTTGATGGTTCCGCGGCGACCCCTATAACCAAAGTTACAGCGGAACAATGGGGTCGTCCTTAG

>CF-20dongqubaoyuzuzhi20250528

ATGTTGGGGAGATGCTTGACCGCGGGCTGTTGCTCGCAATTGCCTTTTTTGTGGTATATCGTGCCGTTCTGTTTTGTTGTGCTCGTCAACGCCAACAACGACAGCAGCTCCCATTTACAGTTGATTTATAACCTGACGATATGTGAGCTGAATGGCACAGATTGGCTAAATAAAAGTTTTGATTGGGCCGTGGAGACCTTTGTTATTTTCCCTGTGTTGACTCATATTGTCTCCTATGGTGCCCTAACCACCAGCCATTTCCTTGACACAGTTGGCCTGGTCACCGTGTCTACCGCTGGATATTGTCACGGGCGGTATGTCCTGAGCAGCATTTACGCCGTCTGCGCCCTGGCTGCGTTAACTTGCTTTGTAATCAGGCTAACAAAAAATTGCATGTCCTGGCGTTACTCTTGCACCAGATACACTAGCTTTCTTTTGGACACCAAGGGCAAACTCTATCGTTGGCGATCTTCCGTCATCGTGGAGAAAGGGGGTAAAGTTGAGGTCGAAGGCCACCTGATCGACCTCAAGAGAGTTGTGCTTGATGGTTCCGCGGCGACCCCTGTAACCAAAATTTCAGCGGAACAATGGGGTCGTCCTTAG

>CF-26beiqubaoyuzuzhi20250528

ATGTTGGGGAAGTGCTTGACCGCGTGCTGTTGCTCGCGATTGCTTTTTTTGTGGTGTATCGTGCCGTCCTATCTTGTTGTGCTCGTCAACGCCAGCAACAACAACAGCTCTCATATTCAGTTGATTTATAACTTAACGCTATGTGAGCTGAATGGCACAGATTGGCTGGCACAAAAATTTGACTGGGCAGTGGAGACTTTCGTCATCTTCCCCGTGTTGACTCACATTGTTTCCTATGCGGCACTCACCACCAGCCATTTCCTTGACACAGTTGGTCTGGCCACTGTGTCCACCGCCGGATATTATCACGGGCGGTATGTCTTGAGTAGCATTTACGCAGTCTGTGCTCTGGCTGCGCTGATTTGCTTTGTCATTAGGCTTGCGAAGAACTGCATGTCCTGGCGCTACTCTTGTACCAGATATACCAACTTCCTTCTGGACACTAAGGGCAAACTCTATCGTTGGCGGTCGCCCGTCATTGTGGAGAAAGGGGGTAAGGTTGAGGTCGAAGGTCACCTGATCGACCTCAAGAGAGTTGTGCTTGATGGTTCCGCAGCAACCCCTTTAACCAGAGTTTCAGCGGAACGATGGGGTCGTCTCTAG

>CF-9fenmian3dongduanweiye20250528

ATGTTGGGGAAATGCTTGACCGCGGGCTGCTGCTCGCAATTGCCTTTTTTGTGGTGTATCGTGCCGTTCTGTTTAGTTGCGCTCGTCAACGCCAACGAAAACAGCAGCTCCCACTTACAATTGATTTATAACCTAACGATATGTGAGCTGAATGGCACAGATTGGCTGAATGGACACTTTTCTTGGGCGGTGGAGACTTTCGTTATTTTTCCTGTGTTAACTCATATTGTCTCTTACGGTGCCCTCACCACTAGCCATTTTCTTGACACGGTCGGTCTTATCACTGTGTCCACCGCCGGGTATTGTCACGGGCGGTATGTATTGAGTAGCATCTACGCTGTCTGCGCCCTAGCTGCGCTTGGTTGCTTCGCCATTAGGTTGGTAAAAAATTGCATGTCCTGGCGCTACTCTTGCACCAGATATACTAATTTTCTTCTGGACACTAAGGGAAAACTCTATCGTTGGCGGTCACCCGTCATCATAGAGAAGGGGGGCAAGGTGGATGTTGGTGGTCATTTAATCGACCTAAAGAGAGTTGTGCTTGAAGGTTCCGCGGCAACCCCTGTAACCAAGATTTCAGCGGAACAATGGGGTCGTCCATAG

>CF-15peihuai2dongzuzhi20250530

ATGTTGGGGAAATGCTTGACCGCGGGCTGCTGCTCGCAATTGCCTTTTTTGTGGTGTATCGTGCCGTTCTGTTTAGTTGCGCTCGTCAACGCCAACGACGACAGCAGCTCCCACTTGCAATTGATTTATAACCTAACGATATGTGAGCTGAATGGCACAGATTGGCTGAAAGAACACTTTTCTTGGGCGGTGGAGACTTTCGTTATTTTTCCTGTGTTAACTCATGTTGTCTCTTACGGTGCCCTCACCACTAGCCATTTTCTTGACACGGTCGGTCTTATCACTGTGTCCACCGCCGGGTATTATCACGGGCGGTATGTATTGAGTAGCATCTACGCTGTTTGCGCCCTAGCTGCGCTTGTTTGCTTCGCCATTAGGTTGGTAAAGAATTGCATGTCCTGGCGCTTCTCTTGCACCAGATATACTAATTTTCTTCTGGACACTAAGGGAAAACTCTATCGTTGGCGGTCACCCGTCATCATAGAGAAGGGGGGCAAGGTGGATGTTGGGGGTCATTTAATCGACCTAAAGAGAGTTGTGCTTGAAGGTTCCGCGGCAACCCCTGTAACCAAGATTTCAGCGGAACAATGGGGTCGTCCATAG

>CF-6yufeizuzhi20250530

ATGTTGGGGAAATGCTTGATCGCGGGTTATTGCTCGCAATTGCTTTTTTTGTGGTGTATCGTGCCATTCTGTTTTGTTGCACCCGTCAACGCCAACAACAGCAGCAGCTCCCATTTCCAGTTGATTTATAACTTAACGATATGTGAGCTGAATGGCACAGATTGGCTGACCAAAAATTTTTCTTGGCCAGTAGAGACTTTGGTCATCTTTCCGGTGTTAACTCATATTGTTTCTTACGGTGCCCTCACCACTAGTCATTTTCTTGACACGGCCGCCTTGATCACTGTGTCCCCCGCCGGGTATTTTCACGGGCGGTATGTATTGAGTAGCATCTATGCGGTCTGTGCCCTAGCTGCGTTGGTTTGCTTCGCCATTAGATTGGCAAAAAATTGCATGTCTTGGCGCTACTCCTGCACCAGATATACCAATTTTCTTCTGGACACTAAGGGCAAACTCTATCGTTGGCGGTCACCCGTCATCATAGAGAAGGGGGGCAAAGTGGACGTTGGAGGTCATTTAATCGACCTCAAGAAAGTTGTGCTTGAAGGTTCCGCGGCAACCCCTGTAACCAAAATTTCAGCGGAACTATGGGGTCGTCCATAG

>CF-21dongqupeihuaizuzhi20250530

ATGTTGGGGAAATGCTTGACCGCGGGCTGCTGCTCGCAATTGCTTTTTTTGTGGTGTATCGTGCCGTTCTGTTTAGTTGCGCTCGTCAACGCCAGCAACAGCAGCAGCTCTCATTTACAGTTGATTTATAACCTGACGATATGTGAGCTGAATGGCACAGATTGGCTAAATCGCAATTTTGATTGGGCAGTGGAGACCTTCGTTATTTTTCCTGTGTTGACTCATATCGTCTCCTATGGCGCCCTAACCACCAGCCACTTCCTTGACACAGTCAGCCTGATCACCGTGTCTACTGCTGGATATTGCCACGGGCGGTATGTCTTGAGCAGTATTTACGCCGTCTGCGCCTTAGCTGCGCTAATTTGCTTCGTCATCAGGCTAACAAAAAATTGCATGTCCTGGCGCTACTCATGCACCAGATACACTAATTTTCTTCTGGACACCAAGGGAAAACTCTATCGTTGGCGGTCTCCCGTCATCGTGGAAAAAGGGGGTAAAGTTGAGGTCGAAGGCCACCTCATCGACCTCAAGAAAGTTGTGCTTGATGGTTCCGCGGCGACCCCTATAACCAAAGTTTCAGCGGAACAATGGGGTCGTCCTTAG

>CF-6yufeizuzhi20250531

ATGTTGGGGAAATGCTTGACCGCGGGCTGCTGCTCGCAATTGCTTTTTTTGTGGTGTATCGTGCCGTTCTGTTTAGTTGCGCTCGTCAACGCCAACGACAACAGCAGCTCCCACTTACAATTGATTTATAACCTAACGATATGTGAGCTGAATGGCACAGATTGGCTGAAAGACCACTTTTATTGGGCGGTGGAGACTTTCGTTATTTTTCCTGTGTTAACTCATATTGTCTCTTACGGTGCCCTCACCACTAGCCATTTTCTTGACACGGTCGGTCTTATCACTGTGTCCACCGCCGGGTATTATCACGGGCGGTATGTATTGAGTAGCATCTACGCTGTCTGCGCCCTAGCTGCGCTTGTTTGCTTCGCCACTAGGTTGGTAAAGAATTGCATGTCCTGGCGTTACTCTTGCACCAGATATACTAATTTTCTTCTGGACACCAAGGGAAAACTCTATCGTTGGCGGTCACCCGTCATCATAGAGAAGGGGGGCAAGGTGGATGTTGGTGGTCACTTAATCGACCTAAAGAGAGTTGTGCTTGAAGGTTCCGCGGCAACCCCTGTAACCAAGATTTCAGCGGAACAATGGGGTCGTCCATAG

>CF-11fenmiantaiyixue20250602

ATGTTGGGGAAATGCTTGACCGCGGGCTGCTGCTCGCAATTGCCTTTTTTGTGGTTTATCGTGCCGTTCTGTTTAGTTGCGCTCGTCAACGCCAACGACAACAGCAGCTCCCACTTACAATTGATTTATAACCTAACGATATGTGAGCTGAATGGCACAGATTGGCTGAAAGACCACTTTTCTTGGGCGGTGGAGACTTTCGTTATTTTTCCTGTGTTAACTCATATTGTCTCTTACGGTGCCCTCACCACTAGCCATTTTCTTGACACGGTCGGTCTTATCACTGTGTCCACCGCCGGGTATTATCACGGGCGGTATGTATTGAGTAGCATCTACGCTGTCTGCGCCCTAGCTGCGCTTGTTTGCTTCGCCATTAGGTTGGTAAAGAATTGCATGTCCTGGCGCTACTCTTGCACCAGATATACTAATTTTCTTCTGGACACTAAGGGAAAACTCTATCGTTGGCGGTCACCCGTCATCATAGAGAAGGGGGGCAAGGTGGATGTTGGTGGTCATTTAATCGACCTAAAGAGAGTTGTGCTTGAAGGTTCCGCGGCAACCCCTGTAACCAAGATTTCAGCGGAACAATGGGGTCGTCCATAG

>CF-3fenmian20250604

ATGTTGGAGAAATGCTTGACCGCGGGCTGTTGCTCGCAATTGCTTTCTTTGTGGTGTATCGTGCCGTTCTGTTTTGCTGTGCTCGCCAACGCCAGCAACGACAGCAGCTCCCATCTACAGCTGATTTACAACTTGACGCTATGTGAGCTGAATGGCACAGATTGGCTAGCTAACAAATTTGATTGGGCAGTGGAGAGTTTTGTCATCTTTCCCGTTTTGACTCACATTGTCTCCTATGGTGCCCTCACTACCAGCCATTTCCTTGACACAGTCGCTTTAGTCACTGTGTCTACCGCCGGGTTTGTTCACGGGCGGTATGTCCTAAGTAGCATCTACGCGGTCTGTGCCCTGGCTGCGTTGACTTGCTTCGTCATTAGGTTTGCAAAGAATTGCATGTCCTGGCGCTACGCGTGTACCAGATATACCAACTTTCTTCTGGACACTAAGGGCGGACTCTATCGTTGGCGGTCGCCTGTCATCATAGAGAAAAGGGGCAAAGTTGAGGTCGAAGGTCATCTGATCGACCTCAAAAGAGTTGTGCTTGATGGTTCCGTGGCAACCCCTATAACCAGAGTTTCAGCGGAACAATGGGGTCGTCCTTAG

>CF-15fenmianweigenxue20250604

ATGTTGGGGAAATGCTTGACCGCGGGCTGCTGCTCGCAATTGCCTTTTTTGTGGTGTATCGTGCCGTTCTGTTTGGTTGCGCTCGTCAACGCCAACGACAACAGCAGCTCCCACTTACAATTGATTTATAACCTAACGATATGTGAGCTGAATGGCACAGATTGGCTGAAAGAACACTTTTCTTGGGCGGTGGAGACTTTCGTTATTTTTCCTGTGTTAACTCATGTTGTCTCTTACGGTGCCCTCACCACTAGCCATTTTCTTGACACGGTCGGTCTTATCACTGTGTCCACCGCCGGGTATTATCACGGGCGGTATGTATTGAGTAGCATCTACGCTGTCTGCGCCCTAGCTGCGCTTGTTTGCTTCGCCATTAGGTTGGCAAAGAATTGCATGTCCTGGCGCTACTCTTGCACCAGATATACTAATTTTCTTCTGGACACTAAGGGAAAACTCTATCGTTGGCGGTCACCCGTCATCATAGAGAAGGGGGGCAAGGTGGATGTTGGGGGTCATTTAATCGACCTAAAGAGAGTTGTGCTTGAAGGTTCCGCGGCAACCCCTGTAACCAAGATTTCAGCGGAACAATGGGGTCGTCCATAG

>CF-7fenmiankouqiangye20250604

ATGTTGGGGAAATGCTTGACCGCGGGCTGCTGCTCGCAATTGCTTTTTTTGTGGTGTATCGTGCCGTTCTGTTTAGTTTCGCTCGTCAACGCCAACAACAACAGCAGCTCCCATTTACAGTTGATTTATAACCTGACGATATGTGAGCTGAATGGCACAGATTGGCTAAATCACACTTTTGATTGGGCGGTGGAGACCTTCGTTATTTTTCCTGTGTTGACCCATATCGTCTCCTATGGCGCCCTAACCACCAGCCATTTCCTTGACACAGTCGGCCTGATCACCGTGTCTATCGCTGGATATTACCACGGGCGGTATGTCTTGAGCAGCATTTACGCCGTCTGCGCCCTAGCTGCGCTAATTTGCTTCGTCATCAGGCTAACAAAAAATTGCATGTCCTGGCGTTACTCATGCACCAGATACACTAACTTTCTTCTGGACACCAAGGGAAAACTCTATCGTTGGCGGTCTCCCGTCATCGTGGAAAAAGGGGGTAAAGTTGAGGTCGAAGGCCACCTCATCGACCTCAAGAAAGTTGTGCTTGACGGCTCCGCGGCGACCCCTATAACCAAAGTTACAGCGGAACAATGGGGTCGTCCTTAG

>CF-17nanqufenmiantaiyixue20250605

ATGTTGGGGAAATGCTTGACCGCGGGCTGCTGCTCGCAATTGCTTTTTTTGTGGTGTATCGTGCCGTTCTGTTTAGTTGCGTTCGTCAACGCCAACAACAGCAACAGCTCCCACTTACAGTTGATTTATAGCCTAACGATATGTGAGCTGAATGGCACAGAGTGGCTGAATAAACATTTTTCTTGGGCAGTGGAGACTTTCGTTATTTTTCCTGTGTTAACCCACATTGTCTCTTACGGTGCCCTCACCACTAGCCATTTTCTTGACACGGTCGGCCTAATCACTGTGTCCACCGCCGGGTATTGTCACGGGCGGCATGTACTGAGTAGCATCTACGCTGTCTGCGCCCTAGCTGCGCTTGTTTGCTTCGCCATTAGGTTGGTAAAAAATTGCATGTCCTGGCGCTACTCTTGCACCAGACACACTAATTTCCTTCTGGACACTAAGGGAAAACTCTATCGTTGGCGGTCACCCGTCATCATAGAGAAAGGGGGCAAGGTGGATGTTGGGGGTCATTTAATTGACCTGAAGAGAGTTGTGCTTGAAGGTTCCGCGGCGACTCCTGTAACCAAGATTTCAGCGGAACAATGGGGTCGTCCATAG

>CF-15houbeixueqing20250609

ATGTTGGGGAAATGCTTGACCGCGGGCTGCTGCTCGCAATTGCCTTTTTTGTGGTGTATCGTGCCGTTCTGTTTAGTTGCGCTCGTCAACGCCAACAACGACAGCAGCTCCCACTTACAATTGATTTATAACCTAACGATATGTGAGCTGAATGGCACAGATTGGCTGAACAAACACTTTCCTTGGGCGGTGGAGACTTTCGTTATTTTTCCTGTGTTAACGCATGTTGTCTCTTGTGGTGCCCTCACCACTAGCCATTTTCTTGACACGGTCGGTCTTATCACTGTGTCCACCGCCGGGTATTATCACGGGCGGTATGTATTGAGTAGCATCTACGCTGTCTGCGCCCTAGCTGCGCTTGTTTGCTTCGCCATTAGGTTGGTAAAGAATTGCATGTCCTGGCGCTACTCTTGCACCAGATATACTAATTTTCTTCTGGACACAAAGGGAAAACTCTATCGTTGGCGGTCACCCGTCATCATAGAGAAGGGGGGCAAGGTGGATGTTGGGGGTCATTTAATCGACCTAAAGAGAGTTGTGCTTGAAGGTTCCGCGGCAACCCCTGTAACCAAGATTTCAGCGGAACAATGGGGTCGTCCATAG

>CF-15fenmianzizhuxueqing20250612

ATGTTGGGGAAATGCTTGACCGCGGGCTGCTGCTCGCAATTGCCTTTTTTGTGGTGTATCGTGCCGTTCTGTTTGGTTGCGCTCGTCAACGCCAACGACAACAGCAGCTCCCACTTACAATTGATTTATAACCTAACGATATGTGAGCTGAATGGCACAGATTGGCTGAAAGAACACTTTTCTTGGGCGGTGGAGACTTTCGTTATTTTTCCTGTGTTAACTCATGTTGTCTCTTACGGTGCCCTCACCACTAGCCATTTTCTTGACACGGTCGGTCTTATCACTGTGTCCACCGCCGGGTATTATCACGGGCGGTATGTATTGAGTAGCATCTACGCTGTCTGCGCCCTAGCTGCGCTTGTTTGCTTCGCCATTAGGTTGGCAAAGAATTGCATGTCCTGGCGCTACTCTTGCACCAGATATACTAATTTTCTTCTGGACACTAAGGGAAAACTCTATCGTTGGCGGTCACCCGTCATCATAGAGAAGGGGGGCAAGGTGGATGTTGGGGGTCATTTAATCGACCTAAAGAGAGTTGTGCTTGAAGGTTCCGCGGCAACCCCTGTAACCAAGATTTCAGCGGAACAATGGGGTCGTCCATAG

>CF-3fenmian2dongtaiyixue20250613

ATGTGGGGGAAATGCTTGACCGCGGGCTGCTGCTCGCAATTGCCTTTTTTGTGGTGTATCGTGCCGTTCTGTTTAGTTGCGCTCGTCAACGCCAACGACAACAGCAGCTCCCACTTACAATTGATTTATAACCTAACGATATGTGAGCTGAATGGCACAGATTGGCTGAAAGACCACTTTTCTTGGGCGGTGGAGACTTTCGTTATTTTTCCTGTGTTAACTCATATTGTCTCTTACGGTGCCCTCACCACTAGCCATTTTCTTGACACGGTCGGTCTTATCACTGTGTCCACCGCCGGGTATTATCACGGGCGGTATGTATTGAGTAGCATCTACGCTGTCTGCGCCCTAGCTGCGCTTGTTTGCTTCGCCATTAGGTTGGTAAAGAATTGCATGTCCTGGCGCTACTCTTGCACCAGATATACTAATTTTCTTCTGGACACTAAGGGAAAACTCTATCGTTGGCGGTCACCCGTCATCATAGAGAAGGGGGGCAAGGTGGATGTTGGAGGTCATTTAATCGACCTAAAGAGAGTTGTGCTTGAAGGTTCCGCGGCAACCCCTGTAACCAAGATTTCAGCGGAACAATGGGGTCGTCCATAG

>CF-8yiqubaoyuxueqing20250614

ATGTTGGAGAAATGCTTGACCGCGGGCTGTTGCTCGCAATTGCTTTCTTTGTGGTGTATCGTGCCGTTCTGTTTTGCTGTGCTCGCCAACGCCAGCAACGACAACAGCTCCCATCTACAGCTGATTTACAACTTGACGCTATGTGAGCTGAATGGCACAGATTGGCTAGCTAACAAATTTGATTGGGCAGTGGAGAGTTTTGTCATCTTTCCCGTTTTGACTCACATTGTCTCCTATGGTGCCCTCACTACCAGCCATTTCCTTGACACAGTCGCTTTAGTCACTGTGTCTACCGCCGGGTTTGTTCACGGGCGGTATGTCCTAAGTAGCATCTACGCGGTCTGTGCCCTGGCTGCGTTGACTTGCTTCGTCATTAGGTTTGCAAAGAATTGCATGTCCTGGCGCTACGCGTGTACCAGATATACCAACTTTCTTCTGGACACTAAGGGCAGACTCTATCGTTGGCGGTCGCCTGTCATCATAGAGAAAAGGGGCAAAGTTGAGGTCGAAGGTCATCTGATCGACCTCAAAAGAGTTGTGCTTGATGGTTCCGTGGCAACCCCTATAACCAGAGTTTCAGCGGAACAATGGGGTCGTCCTTAG

>CF-26erqubaoyuzuzhi20250620

ATGTTGGAGAAATGCTTGACCGCGGGCTGTTGCTCGCAATTGCTTTCTTTGTGGTGTATCGTGCCGTTCTGTTTTGCTGTGCTCGCCAACGCCAGCAACGACAGCAGCTCCCATCTACAGCTGATTTACAACTTGACGCTATGTGAGCTGAATGGCACAGATTGGCTAGCTAACAAATTTGATTGGGCAGTGGAGAGTTTTGTCATCTTTCCCGTTTTGACTCACATTGTCTCCTATGGTGCCCTCACTACCAGCCATTTCCTTGACACAGTCGCTTTAGTCACTGTGTCTACCGCCGGGTTTGTTCACGGGCGGTATGTCCTAAGTAGCATCTACGCGGTCTGTGCCCTGGCTGCGTTGACTTGCTTCGTCATTAGGTTTGCAAAGAATTGCATGTCCTGGCGCTACGCGTGTACCAGATATACCAACTTTCTTCTGGACACTAAGGGCGGACTCTATCGTTGGCGGTCGCCTGTCATCATAGAGAAAAGGGGCAAAGTTGAGGTCGAAGGTCATCTGATCGACCTCAAAAGAGTTGTGCTTGATGGTTCCGTGGCAACCCCTATAACCAGAGTTTCAGCGGAACAATGGGGTCGTCCTTAG

>CF-15houbeixueqing20250626

ATGTTGGGGAAATGCTTGACCGCGGGCTGCTGCTCGCAATTGCTTTTTTTGTGGTGTATCGTGCCGTTCTGTTTAGTTTCGCTCGTCAACGCCAGCAACAGCAGCAGCTCCCATTTACAGTTGATTTATAACCTGACGATATGTGAGCTGAATGGCACGGATTGGCTGAATCGCACTTTTGATTGGGCAGTGGAGACCTTCGTTATTTTTCCTGTGTTGACTCATATCGTCTCCTACGGCGCCCTAACCACCAGCCACTTCCTTGACACAGTCGGCCTGATCACCGTGTCTACTGCTGGATATTACCACGGGCGGTATGTCTTGAGCAGTATTTACGCCGTCTGCGCCTTAGCTGCGCTAATTTGCTTCGTCATCAGGCTAACAAAAAATTGCATGTCCTGGCGTTACTCATGCACCAGATACACTAATTTTCTTCTGGACACCAAGGGAAAACTCTATCGTTGGCGGTCTCCCGTCATCGTGGAAAAAGGGGGTAAAGTTGAGGTCGAAGGCCACCTCATCGACCTCAAGAAAGTTGTGCTTGATGGTTCCGCGGCGACCCCTATAACCAAAGTTACAGCGGAACAATGGGGTCGTCCTTAG

>CF-8erqubaoyuxueqing20250630

ATGTTGGGGAAATGCTTGACCGCGGGCTGCTGCTCGCAATTGCTTTTTTTGTGGTGTATCGTGCCGTTCTGTTTAGTTGCGCTCGTCAACGCCAGCAACAACAGCAGCTCCCATTTACAGTTGATTTATAACCTGACGATATGTGAGCTGAATGGCACAGATTGGCTAAATAAAAATTTTGATTGGGCAGTGGAGACTTTCGTTATTTTTCCTGTGTTGACTCATATTGTCTCCTATGGTGCCCTCACCACCAGCCATTTCCTTGACACAGTCGGTCTGATCACTGTGTCTACCGCCGGGTATTATCACGGGCGGTATGTCTTGAGTAGCATCTACGCTGTCTGCGCCCTAGCTGCGCTTATTTGCTTCGTCATTAGGTTGGCAAAGAATTGCATGTCCTGGCGCTACTCTTGCACCAGATA-ACTAATTTTCTTCTGGAC-CTAAGGGAAAACTCTATCGTTGGCGGTC-CCCGTCATCATAGAAAAAGGGGGAAAG--TGAGGTTGAAGGTCA-TTGATCGACCTAAAAAAG--TGTGCTTGAAGGT-CCGCGGC-ACCCCTATA-CCAAAATTCC-GCGGAACAATGGGGTCGCCT-TAG

>CF-7fenmianerdongduanweiye20250630

ATGTTGGTGAAATGCTTGACCGCGGGCTGCTGCTCGCAATTGCTTTTTTTGTGGTGTATCGTGCCGTTCTGTTTAGTTTCGCTCGTCAACGCCAGCGACAGCAGCAGCTCCCATTTACAGTTGATTTATAACCTGACGATATGTGAGCTGAATGGCACAGATTGGCTAAATCACACTTTTGATTGGGCAGTGGAGACCTTCGTTATTTTTCCTGTGTTGACTCATATCGTCTCCTATGGCGCCCTGACCACCAGCCACTTCCTTGACACAGTCGGCCTGATCACCGTGTCCACTGCTGGATATTACCACGGGCGGTATGTTTTGAGCAGTATTTACGCCGTCTGCGCCTTGGCTGCGCTAATTTGCTTCATCATCAGGCTAACAAAAAATTGCATGTCCTGGCGTTACTCGTGCACCAGATACACTAATTTTCTTCTGGACACCAAGGGAAAACTCTATCGTTGGCGGTCTCCCGTCATCGTGGAAAAAGGGGGTAAAGTTGAGGTCGAAGGCCACCTCATTGACCTCAAGAAAGTTGTGCTTGATGGTTCCGCGGCGACCCCTATAACCAAAGTTACAGCGGAACAATGGGGTCGTCCTTAG

>CF-7fenmiansidongduanweiye20250630

ATGTTGGGGAAGTGCTTGACCGCGTGCTGTTGCTCGCGATTGCTTTTTTTGTGGTGTATCGTGCCGTTCTATCTTGCTGTGCTCGTCAACGCCAGCAACAACAACAGCTCTCATATTCAGTTGATTTATAACTTGACGCTATGTGAGCTGAATGGCACAGATTGGCTGGCACAAAATTTTGACTGGGCAGTGGAGACTTTTGTCATCTTCCCCGTGTTGACTCACATTGTTTCCTATGGGGCACTCACCACCAGCCATTTCCTTGACACAGTTGGTCTGGCCACTGTGTCCACCGCCGGATATTATCACGGGCGGTATGTCTTGAGTAGCATTTACGCAGTCTGTGCTCTGGCTGCGCTGATTTGCTTTGTCATTAGGCTTGCGAAGAACTGCATGTCCTGGCGCTACTCTTGTACCAGATATACCAACTTCCTTCTGGACACTAAGGGCAGACTCTATCGTTGGCGGTCGCCCGTCATTGTGGAGAAAAGGGGTAAGGTTGAGGTCGAAGGTCACCTGATCGACCTCAAGAGAGTTGTGCTTGATGGTTCCGCGGCAACCCCTTTAACCAGAGTTTCAGCGGAACTATGGGGTCGTCTCTAG

>CF-1fenmianerdongzizhuxueqing20250712

ATGTTGGGGAAATGCTTGACCGCGGGCTGCTGCTCGCAATTGCTTTTTTTGTGGTGTATCGTGCCGTTCTGTTTAGTTGCGCTCGTCAACGCCGACAACAACAGCAGCTCCCACTTACAGTTGATTTATAACCTAACGATATGTGAGCTGAATGGCACAGATTGGCTGAAGGAACATTTTTCTTGGGCAGTAGAGACTTTCGTTATATTTCCTGTGTTAACTCATATTGTCTCTTACGGTGCCCTCACCACTAGCCATTTTCTTGACACGGTTGGCCTGATCACTGTGTCCACTGCCGGGTATTATCACAGGCGGTATGTACTGAGTAGCATCTACGCTGTCTGCGCCCTAGCTGCGCTTGTTTGCTTCACCATTAGGTTCGTAAAAAATTGCATGTCCTGGCGCTACTCTTGCACCAGATATACTAACTTTCTTCTGGACACTAAGGGAAAACTCTATCGTTGGCGGTCACCTGTCATCATAGAGAAGGGGGGCAAGGTGGATGTTGGTGGTCATTTAATTGACCTAAAGAGAGTTGTGCTTGAAGGTTCCGCGGCGACTCCTGTAACCAGGATTTCAGCGGAACAATGGGGTCGTCCATAG

>CF-23peihuaierdongmuzhuweigenxue20250718

ATGTTGGGGAAGTGCTTGACCGCGTGCTGTTGCTCGCGATTGCTTTTTTTGTGGTGTATCGTGCCGTCCTATCTTGCTGTGCTCGTCAACGCCAGCAACAACAACAGCTCTCATATTCAGTTGATTTATAACTTAACGCTATGTGAGCTGAATGGCACAGATTGGCTGGCACAAAAATTTGACTGGACAGTGGAGACTTTCGTCATCTTCCCCGTGTTGACTCACATTGTTTCCTATGCGGCACTCACCACCAGCCATTTCCTTGACACAGTTGGTCTGGCCACTGTGTCCACCGCCGGATATTATCACGGGCGGTATGTCTTGAGTAGCATTTACGCAGTCTGTGCTCTGGCTGCGCTGATTTGCTTTGTCATTAGGCTTGCGAAGAACTGCATGTCCTGGCGCTACTCTTGTACCAGATATACCAACTTCCTTCTGGACACTAAGGGCAAACTCTATCGTTGGCGGTCGCCCGTCATTGTGGAGAAAGGGGGTAAGGTTGAGGTCGAAGGTCACCTGATCGACCTCAAGAGAGTTGTGCTTGATGGTTCCGCGGCAACCCCTTTAACCAGAGTTTCAGCGGAACGATGGGGTCGTCTCTAG

>CF-16yiqubaoyuzhuxueqing20250722

ATGTTGGGGAAATGCTTGACCGCGGGCTGCTGCTCGCAATTGCTTTTTTTGTGGTGTATCGTGCCGTTCTGTTTAGTTTCGCTCGTCAACGCCAACTACAGCAGCAGCTCCCATTTACAGTTGATTTATAACCTGACGATATGTGAGCTGAATGGCACGGATTGGCTAAATCGCACTTTTGATTGGGCGGTGGAGACCTTTGTTATTTTTCCTGTGTTGACTCATATCGTTTCCTATGGCGCCCTAACCACCAGCCACTTCCTTGACACAGTCGGCCTGATCACCGTGTCTACTGCTGGATATTACCACGGGCGGTATGTCTTGAGCAGTATTTACGCCGTCTGCGCCTTAGCTGCGCTAATTTGCTTCGTCATCAGGCTAACAAAAAATTGCATGTCCTGGCGTTACTCATGCACCAGATACACTAATTTTCTTCTGGACACCAAGGGAAAACTCTATCGTTGGCGGTCTCCCGTCATCGTGGAAAAAGGGGGTAAAGTTGAGGTCGAAGGCCACCTCATCGACCTCAAGAAAGTTGTGCTTGATGGTTCCGCGGCGACCCCTATAACCAAAGTTACAGCGGAACAATGGGGTCGTCCTTAG

>CF-24baoyu9jianxueqing20250721

ATGTTGGGGAAGTGCTTGACCGCGTGCTGTTGCTCGCGATTGCTTTTTTTGTGGTGTATCGTGCCGTCCTATCTTGCTGTGCTCGTCAACGCCAGCAACAACAACAGCTCTCATATTCAGTTGATTTATAACTTAACGCTATGTGAGCTGAATGGCACAGATTGGCTGGCACAAAAATTTGACTGGACAGTGGAGACTTTCGTCATCTTCCCCGTGTTGACTCACATTGTTTCCTATGCGGCACTCACCACCAGCCATTTCCTTGACACAGTTGGTCTGGCCACTGTGTCCACCGCCGGATATTATCACGGGCGGTATGTCTTGAGTAGCATTTACGCAGTCTGTGCTCTGGCTGCGCTGATTTGCTTTGTCATTAGGCTTGCGAAGAACTGCATGTCCTGGCGCTACTCTTGTACCAGATATACCAACTTCCTTCTGGACACTAAGGGCAAACTCTATCGTTGGCGGTCGCCCGTCGTTGTGGAGAAAGGGGGTAAGGTTGAGGTCGAAGGTCACCTGATCGACCTCAAGAGAGTTGTGCTTGATGGTTCCGCGGCAACCCCTTTAACCAGAGTTTCAGCGGAACGATGGGGTCGTCTCTAG

>CF-24baoyu15jianxueqing20250721

ATGTTGGGGAAATGCTTGACCGCGGGCTGCTGCTCGCAATTACTTTTTTTGTGGTGTATCGTGCCGTTCTGTTTAGTTGCGTTCGTCAACGCCAACAACAGCAGCAGCTCCCACTTACAGTTGATTTATAACCTAACGATATGTGAGCTGAATGGCACAGATTGGCTGAAAGAACATTTTTCTTGGGCAGTGGAGACTTTCGTTATTTTTCCTGTGTTAACCCACATTGTCTCTTACGGTGCCCTCACCACTAGCCATTTTCTTGACACGGTCGGCCTAATCACTGTGTCCACCGCCGGGTATTATCACGGGCGGTATGTACTGAGTAGCATCTATGCTGTCTGCGCCCTAGCTGCGCTTGTTTGCTTCGCCATTAGGTTGGTAAAAAATTGCATGTCCTGGCGCTACTCTTGCACCAGATATACTAATTTTCTTCTGGACACTAAGGGAAAACTCTATCGTTGGCGGTCACCCGTCATCATAGAGAAAGGGGGCAAGGTGGATGTTGGGGGTCATTTAATTGACCTGAAGAGAGTTGTGCTTGAAGGTTCCGCGGCGACTCCTGTAACCAAGATTTCAGCGGAACAATGGGGACATCCATAG

>CF-22baoyuxueqing20250727

ATGTTGGGGAGATGCTTGACCGCGGGCTGTTGCTCGCAATTGCCTTTTTTGTGGTATATCGTGCCGTTCTGTTTTGTTGTGCTCGTCAACGCCAACAACAGCAGCAGCTCCCATTTACAGTTGATTTATAACCTGACGATATGTGAGCTGAATGGCACAGATTGGCTAAATAAAAGTTTTGATTGGGCAGTGGAGACCTTTGTTATTTTCCCTGTGTTGACTCATATTGTCTCCCATGGCGCCCTAACCACCAGCCATTTCCTTGACACAGTTGGCCTGATCACCGTGTCTACCGCTGGGTATTACCACGGGCGGTATGTCCTGAGCAGCATTTACGCCGTCTGCGCCCTAGCTGCGTTAACTTGCTTTGTCATCAGGCTAACAAAAAATTGCATGTCCTGGCGTTACTCATGCACCAGATACACTAACTTTCTTCTGGACACCAAGGGCAAACTCTATCGTTGGCGGTCTCCCGTCATCGTGGAGAAAGGGGGTAAAGTTGAGGTCGCAGGCCACCTGATCGACCTCAAGAGAGTTGTGCTTGACGGTTCCGCGGCAACCCCTATAACCAAAGTTTCAGCGGAACAATGGGGTCGTCCTTAG

>CF-26baoyu5jianzuzhi20250727

ATGTTGGGGAAATGCTTGACCGCGGGCTGCTGCTCGCAATTGCCTTTTTTGTGGTGTATCGTGCCGTTCTGTTTAGTTGCGCTCGTCAACGCCAACGACAACAGCAGCTCCCACTTACAATTGATTTATAACCTAACGATATGTGAGCTGAATGGCACAGATTGGCTGAAAGACCACTTTTCTTGGGCGGTGGAGACTTTCGTTATTTTTCCTGTGTTAACTCATATTGTCTCTTACGGTGCCCTCACCACTAGCCATTTTCTTGACACGGTCGGTCTTATCACTGTGTCCACCGCCGGGTATTATCGCGGGCGGTATGTATTGAGTAGCATCTACGCTGTCTGCGCCCTAGCTGCGCTTGTTTGCTTCGCCATTAGGTTGGTAAAGAATTGCATGTCCTGGCGCTACTCTTGCACCAGATATACTAATTTTCTTCTGGACACTAAGGGAAAACTCTATCGTTGGCGGTCACCCGTCATCATAGAGAAGGGGGGCAAGGTGGATGTTGGTGGTCATTTAATCGACCTAAAGAGAGTTGTGCTTGAAGGTTCCGCGGCAACCCCTGTAACCAAGATTTCAGCGGAACAATGGGGTCGTCCATAG

>CF-7chanfangduanweiye20250730

ATGTTGGGGAAATGCTTGACCGCGGGCTGCTGCTCGCAATTCCCTTTTTTGTGGTGTATCGTGCCGTTCTGTTTAGTTGCGCTCGTCAACGCCAACGACAACAGCAGCTCCCACTTACAATCGATTTATAACCTAACGATATGTGAGCTGAATGGCACAGATTGGCTGAAAGAACACTTTTCTTGGGCGGTGGAGACTTTCGTTATTTTTCCTGTGTTAACTCATGTTGTCTCTTACGGTGCCCTCACCACTAGCCATTTTCTTGATACGGTCGGTCTTATCACTGTGTCCACCGCCGGGTATTATCACGGGCGGTATGTATTGAGTAGCATCTACGCTGTCTGCGCCCTAGCTGCGCTTGTTTGCTTCGCCATTAGGTTGGTAAAGAATTGCATGTCCTGGCGCTACTCTTGCACCAGATATACTAATTTTCTTCTGGACACTAAGGGAAAACTCTATCGTTGGCGGTCGCCCGTCATCATAGAGAAGGGGGGCAAGGTGGATGTTGGGGGTCATTTAATCGACCTAAAGAGAGTTGTGCTTGAAGGTTCCGCGGCAACCCCTGTAACCAAGATTTCAGCGGAACAATGGGGTCGTCCATAG

>CF-20yiquyufeizuzhi20250801

ATGTTGGGGAGATGCTTGACCGCGGGCTGTTGCTCGCAATTGCTTTTTTTGTGGTATATCGTGCCGTTCTGTTTTGTTGCGCTCGTCAACGCCAACAACGGCAGCAGCTCCCATTTACAGTTGATTTATAACCTGACGATATGTGAGCTGAATGGCACAGATTGGCTAAATAAAAGTTTCGATTGGGCAGTGGAGACCTTTGTTATTTTCCCTGTGTTGACTCATATTGTCTCCTATGGCGCCCTAACCACCAGCCATTTCCTTGACACAGTTGGCCTGATCACCGTGTCTACCGCTGGATATTATCACGGGCGGTATGTCCTGAGCAGCATTTACGCCGTCTGCGCCCTAGCTGCGTTGACTTGCTTTGTCATCAGGCTAACAAAAAATTGCATGTCCTGGCGTTACTCATGCACCAGACACACTAACTTTCTTCTGGACACCAAGGGCAAACTCTATCGTTGGCGGTCTCCCGTCATCGTAGAGAAAGGGGGTAAAGTTGAGGTCGAAGGCCACCTGATCGACCTCAAGAGAGTTGTGCTTGATGGTTCCGCGGCGACCCCTATAACCAAAGTTTCAGCGGAACAATGGGGTCGTCCTTAG

>CF-20erqubaoyu12jianzuzhi20250801

ATGTTGGAGAAATGCTTGACCGCGGGCTGTTGCTCGCAATTGCTTTCTTTGTGGTGTATCGTGCCGTTCTGTTTTGCTGTGCCCGCCAACGCCAGCAACGACAGCAGCTCCCATCTACAGCTGATTTACAACTTGACGCTATGTGAGCTGAATGGCACAGATTGGCTAGCTAACAAATTTGATTGGGCAGTGGAGAGTTTTGTCATCTTTCCCGTTTTGACTCACATTGTCTCCTATGGTGCCCTCACTACCAGCCATTTCCTTGACACAGTCGCTTTAGTCACTGTGTCTACCGCCGGGTTTGTTCACGGGCGGTATGTCCTAAGTAGCATCTACGCGGTCTGTGCCCTGGCTGCGTTGACTTGCTTCGTCATTAGGTTTGCAAAGAATTGCATGTCCTGGCGCTACGCGTGTACCAGATATACCAACTTTCTTCTGGACACTAAGGGCAGACTCTATCGTTGGCGGTCGCCTGTCATCATAGAGAAAAGGGGCAAAGTTGAGGTCGAAGGTCATCTGATCGACCTCAAAAGAGTTGTGCTTGATGGTTCCGTGGCAACCCCTATAACCAGAGTTTCAGCGGAACAATGGGGTCGTCCTTAG

>CF-20erqubaoyu9jianzuzhi20250801

ATGTTGGGGAAATGCTTGACCGCGGGCTGCTGCTCGCAATTGCTTTTTTTGTGGTGTATCGTGCCGTTCTGTTTAGTTGCGTTCGTCAACGCCAACAACAGCAGCAGCTCCCACTTACAGTTGATTTATAACCTAACGATATGTGAGCTGAATGGCACAGATTGGCTGAATGAACATTTTTCTTGGGCAGTGGAGACTTTCGTTATTTTTCCTGTGTTAACTCACATTGTCTCTTACGGTGCTCTCACCACTAGCCATTTTCTTGACACGGTCGGCCTAATCACTGTGTCCACCGCCGGGTATTGTCACGGGCGGTATGTACTGAGTAGCATCTACGCTGTCTGCGCCCTAGCTGCACTTGTTTGCTTCGCCATTAGGTTGGTAAAAAATTGCATGTCCTGGCGCTACTCTTGCACCAGATATACTAATTTCCTTCTGGACACTAAGGGAAAACTCTATCGTTGGCGGTCACCCGTCATCATAGAGAAAGGGGGCAAGGTGGATGTTGGGGGTCATTTAATTGACCTGAAGAGAGTTGTGCTTGAAGGTTCCGCGGCGACTCCTGTAACCAAGATTTCAGCGGAACAATGGGGTCGTCCATAG

>CF-20erquyufeizuzhi20250801

ATGTTGGGGAAATGCTTGACCGCGGGCTGCTGCTCGCAATTGCTTTTTTTGTGGTGTATCGTGCCGTTCTGTTTAGTTGCGTTCGTCAACGCCAACAACAGCAGCAGCTCCCACTTACAGTCGATTTATAACCTAACGATATGTGAGCTGAATGGCACAGATTGGCTGAATGAACATTTTTCTTGGGCAGTGGAGACTTTCGTTATTTTTCCTGTGTTAACTCACATTGTCTCTTACGGTGCCCTCACCACTAGCCATTTTCTTGACACGGTCGGCCTAATCACTGTGTCCACCGCCGGGTATCATCACAGGCGGTATGTACTGAGTAGCATCTACGCTGTCTGCGCCCTAGCTGCGCTTGTTTGCTTCGCCATTAGGTTGGTGAAAAATTGCATGTCCTGGCGCTACTCTTGCACCAGATATACTAACTTCCTTCTGGACACTAAGGGAAAACTCTATCGTTGGCGGTCACCCGTCATCATAGAGAAAGGGGGCAAGGTGGATGTTGGGGGTCATTTAATTGACCTGAAGAGAGTTGTGCTTGAAGGTTCCGCGGCGACTCCTGTAACCAAGATTTCAGCGGAACAATGGGGTCGTCCATAG

>CF-15chanfangduanweiye20250802

ATGTTGGGGAAATGCTTGACCGCGGGCTGCTGCTCGCAATTGCCTTTTTTGTGGTGTATCGTGCCGTTCTGTTTAGTTGCGCTCGTCAACGCCAACGACAACAGCAGCTCCCACTTGCAATCGATTTATAACCTAACGATATGTGAGCTGAATGGCACAGATTGGCTGAAAGAACACTTTTCTTGGGCGGTGGAGACTTTCGTTATTTTTCCTGTGTTAACTCATGTTGTCTCTTACGGTGCCCTCACCACTAGCCATTTTCTTGACACGGTCGGTCTTATCACTGTGTCCACCGCCGGGTATTATCACGGGCGGTATGTATTGAGTAGCATCTACGCTGTTTGCGCCCTAGCTGCGCTTGTTTGCTTCGCCATTAGGTTGGTAAAGAACTGCATGTCCTGGCGCTTCTCTTGCACCAGATATACTAATTTTCTTCTGGACACTAAGGGAAAACTCTATCGTTGGCGGTCACCCGTCATCATAGAGAAGGGGGGCAAGGTGGATGTTGGGGGTCATTTAATCGACCTAAAGAGAGTTGTGCTTGAAGGTTCCGCGGCAACCCCTGTAACCAAGATTTCAGCGGAACAATGGGGTCGTCCATAG

>CF-23yiquchanfangduanweiye20250803

ATGTTGGGGAAATGCTTGACCGCGGGCTGCTGCTCGCAATTGCCTTTTTTGTGGTGTATCGTGCCGTTCTGTTTAGTTGCGCTCGTCAACGCCAACGACAGCAGCAGCTCCCACTTACAATTGATTTATAACCTAACGATATGTGAGCTGAATGGCACAGATTGGCTGAAAGAACACTTTCCTTGGGCGGTGGAGACTTTCGTTATTTTTCCTGTGTTAACTCATGTTGTCTCTTACGGTGCCCTCACCACTAGCCATTTTCTTGACACGGTCGGTCTTATCACTGTGTCCACCGCCGGGTATTATCACGGGCGGTATGTATTGAGTAGCATCTACGCTGTCTGCGCCCTAGCTGCGCTTGTCTGCTTTGCCATTAGGTTGGTAAAGAACTGCATGTCCTGGCGCTACTCTTGCACCAGATATACTAATTTTCTTCTGGACACTAAGGGAAAACTCTATCGTTGGCGGTCACCCGTCATCATAGAGAAGGGGGGCAAGGTGGACGTTGGGGGTCATTTAATCGACCTAAAGAGAGTTGTGCTTGAAGGTTCCGCGGCAGCCCCTGTAACCAAGATTTCAGCGGAACAATGGGGTCGTCCATAG

>CF-5chanfangduanweiye20250807

ATGTTGGGGAAATGCTTGACCGCGGGTTATTGCTCACAATCGGTTTTTTTGTGGTGTATCGTGCCATTCTGTTTTGCTGTGCTCGTCAACGCCAGCGGCAACAGCAGCTCCCGCTTACAATTGATCTATAACCTGACGATATGTGAGCTGAATGGCACAGACTGGTTGAACCAAAGATTTGACTGGGCAGTGGAGACTTTCGTCATTTTTCCTGTCTTGACCCATATCGTCTCTTACGGCGCTCTTACCACTAGCCATTTTCTTGACACGGTCGGCCTAATCACCGTGTCCACTGCTGGGTATTATCATAAGCGGTATGTATTGAGTAGCATCTACGCTGTCTGTGCCCTAGCCGCGTTGGTTTGCTTCGCCATTAGGTTGGCAAAAAATTGTATGTCCTGGCGCTACTCATTCACCAGATATACTAATTTTCTTCTGGACTCTAAGGGCAAACTCTACCGTTGGCGGTCACCCGTCATCATAGAGAAAGAGGGCAAAGTTGATGTAAGGGGTCAGTTAATCGACCTCAAGAGAGTTGTGCTTGATGGTTCCGCGGCGACCCCTGTAACCAAGATTTCAGCGGAACAATGGGGTCGTCCATAG

>CF-18erqubaoyuzuzhi20250808

ATGTTGGGGAAATGCTTGACCGCGGGCTGCTGCTCGCAATTGCTTTTTTTGTGGTGTATCGTGCCGTTCTGTTTAGTTGCGTTCGTCAACGCCAACGACAACAACAGCTCCCACTTACAGTTGATTTATAACCTAACGATATGTGAGCTGAATGGCACAGAGTGGCTGAATAAACATTTTTCTTGGGCAGTGGAGACTTTCGTTATTTTTCCTGTGTTAACCCACATTGTCTCTTACGGTGCCCTCACCACTAGCCATTTTCTTGACACGGTCGGCCTAATCACTGTGTCCACCGCCGGGTATTGTCACGGGCGGTATGTACTGAGTAGCATCTACGCTGTCTGCGCCCTAGCTGCGCTTGTTTGCTTCGCCATTAGGTTGGTAAAAAATTGCATGTCCTGGCGCTACTCTTGCACCAGACACACTAATTTCCTTCTGGACACTAAGGGAAAACTCTATCGTTGGCGGTCACCCGTCATCATAGAGAAAGGGGGCAAGGTGGATGTTGGGGGTCATTTAATTGACCTGAAGAGAGTTGTGCTTGAAGGTTCCGCGGCGACTCCTGTAACCAAGATTTCAGCGGAACAATGGGGTCGTCTATAG

>CF-18yiquyufeizuzhi20250808

ATGTTGGGGAAATGCTTGACCGCGGGCTACTGCTCGCCATTGCTTTTTTTGTGGTGTATCGTGCCGTTCTGTTTAGTTGCGTTCGTCAACGCCAACAACAGCACCAGCTCCCACTTACAGTTGATTTATAACCTAACGATATGTGAGCTGAATGGCACAGATTGGCTGAATGAACATTTTTCTTGGGCAGTGGAGACTTTTGTTATTTTTCCTGTGTTAACTCACATTGTCTCTTACGGTGCCCTCACCACCAGCCATTTTCTTGACACGGTCGGCCTAATCACTGTGTCCACCGCCGGGTATTGCCACGGGCGGTATGTATTGAGTAGCATCTACGCTGTCTGCGCCCTAGCTGCGCTTGTTTGCTTCGCCATTAGGTTGGTAAAAAATTGCATGTCCTGGCGCTACTCTTGCACCAGACATACTAATTTCCTTCTGGACACTAAGGGAAAACTCTATCGTTGGCGGTCACCCGTCATCATAGAGAAAGGGGGCAAGGTGGATGTTGGGGGCCATTTAATTGACCTGAAGAGAGTTGTGCTTGAAGGTTCCGCGGCGACTCCTGTAACCAAGATTTCAGCGGAACAATGGGGTCGTCCATAG

>CF-4baoyu1jianzuzhi20250809

ATGTTGGGGAAATGCTTGACCGCGGGTTATTGCTCGCAATCGCTTTTTTTGTGGTGTATCGTGCCATTCTGTTTTGCTGTGCTCGTCAACGCCAGCAACGACAGCAGCTCCCACTTACAGTTGATCTATAACCTGACAATATGTGAGCTGAATGGCACAGACTGGTTGAACAAAAAATTTGACTGGGCAGTAGAGACCTTCGTTATTTTTCCTGTCTTGACCCATATTGTCTCTTACGGCGCCCTTACCACTAGCCATTTTCTTGACACGGTCGGCCTAATCACCGTATCCACCGCTGGGTATTATCATAAGCGGTATGTATTGAGTAGCATCTACGCTGTCTGTGCCCTGGCCGCGTTGGTTTGTTTCGCCATTAGGTTGGCAAAAAATTGTATGTCCTGGCGCTACTCATGCACCAGATATACCAATTTTCTTCTGGACTCTAAGGGCAGACTCTACCGTTGGCGGTCACCCGTCATCATAGAGAAAGAGGGCAAAGTTGATGTGGGGGGTCAGTTAATCGACCTCAAGAGAGTAGTGCTTGATGGTTCCGCGGCGACCCCTGTAACCAAGATTTCAGCGGAACAATGGGGTCGTCCATAG

>CF-4baoyu6jianzuzhi20250809

ATGTTGGAGAAATGCTTGACCGCGGGCTGTTGCTCGCAATTGCTTTCTTTGTGGTGTATCGTGCCGTTCTGTTTTGCTGTGCTCGCCAACGCCAGCAACGACAGCAGCTCCCATCTACAGCTGATTTACAACTTGACGCTATGTGAGCTGAATGGCACAGATTGGCTAGCTAACAAATTTGATTGGGCAGTGGAGAGTTTTGTCATCTTTCCCGTTTTGACTCACATTGTCTCCTATGGTGCCCTCACTACCAGCCATTTCCTTGACACAGTCGCTTTAGTCACTGTGTCTACCGCCGGGTTTGTTCACGGGCGGTATGTCCTAAGTAGCATCTACGCGGTCTGTGCCCTGGCTGCGTTGACTTGCTTTGTCATTAGGTTTGCAAAGAATTGCATGTCCTGGCGCTACGCGTGTACCAGATATACCAACTTTCTTCTGGACACTAAGGGCATACTCTATCGTTGGCGGTCGCCTGTCATCATAGAGAAAAGGGGCAAAGTTGAGGTCGAAGGTCATCTGATCGACCTCAAAAGAGTTGTGCTTGATGGTTCCGTGGCAACCCCTATAACCAGAGTTTCAGCGGAACAATGGGGTCGTCCTTAG

>CF-9chanfangzizhuxueqing20250811

ATGTTGGGGAAATGCTTGACCGCGGGTTATTGCTCACAATCGCTTTTTTTGTGGTGTATCGTGCCATTCTGTTTTGCTGTGCTCGTCAACGCCAGCGACAACAACAGCTCCCACTTACAGTTGATCTATAACCTGACGATATGTGAGCTGAATGGCACAGACTGGTTGAAACAAAAATTTGACTGGGCAGTAGAGACTTTCGTCATTTTTCCTGTCTTGACCCATATTGTCTCTTACGGCGCCCTTACCACTAGCCATTTTCTTGACACGGTCGGCCTAATCACCGTATCCGCCGCTGGGTATTATCATAAGCGGTATGTGTTGAGTAGCATCTACGCTGTCTGTGCCCTGGCCGCGTTGGTTTGCTTCGCCATTAGGTTGGCAAAAAATTGCATGTCCTGGCGCTACTCATTCACCAGATATACTAATTTTCTTCTGGACTCTAAGGGCAAACTCTACCGTTGGCGGTCACCCGTCATCATAGAGAAAGAGGGCAAAGTTGATGTGGGGGGTCAGTTAATCGAACTCAAGAGAGTTGTGCTTGATGGTTCCGCGGCGACCCCTGTAACCAAGATTTCAGCGGAACAATGGGGTCGTCCATAG

>CF-21erquchanfangzizhuxueqing20250811

ATGTTGGGGAAATGCTTGACCGCGGGCTGCTGCTCGCAATTGCTTTTTTTGTGGTGTATCGTGCCGTTCTGTTTAGTTGCGTTCGTCAACGCCAACAACAGCAGCAGCTCCCACTTACAGTTGATTTATAACCTAACGATATGTGAGCTGAATGGCACAGATTGGCTGAATGAACATTTTTCTTGGGCAGTGGAGACTTTCGTTATTTTTCCTGTGTTAACTCACATTGTCTCTTACGGTGCCCTCACCACTAGCCATTTTCTTGACACGGTCGGCCTAATCACTGTGTCCACCGCCGGGTATTGTCGCGGGCGGTATGTATTGAGTAGCATCTACGCTGTCTGCGCCCTAGCTGCACTTGTTTGCTTCGCCATTAGGTTGGTAAAAAATTGCATGTCCTGGCGCTACTCTTGCACCAGACATACTAATTTCCTTCTGGACACTAAGGGAAAACTCTATCGTTGGCGGTCACCCGTCATCATAGAGAAAGGGGGCAAGGTGGATGTTGGGGGCCATTTAATTGACCTGAAGAGAGTTGTGCTTGAAGGTTCCGCGGCGACTCCTGTAACCAAGATTTCAGCGGAACAATGGGGTCGTCCATAG

>CF-2baoyu9jianxueqing20250812

ATGTTGGGGAAATGCTTGACCGCGGGCTGCTGCTCGCAATTGCTTTTTTTGTGGTGTATCGTGCCGTTCTGTTTAGTTTCGCTCGTCAACGCCAGCAACAGCAACAGCTCCCATTTACAGTTGATTTATAACCTGACGATATGTGAGCTGAATGGCACAGATTGGCTAAATCGCACTTTTGATTGGGCAGTGGAGACCTTCGTTATTTTTCCTGTATTGACTCATATCGTCTCCTATGGCGCCCTAACCACCAGCCACTTCCTTGACACAGTCGGCCTGATCACCGTGTCTACTGCTGGATATTACCACGGGCGGTATGTCTTGAGCAGTATTTACGCCGTCTGCGCCTTAGCTGCGCTAATTTGCTTCGTCATCAGGCTAACAAAAAATTGCATGTCCTGGCGTTACTCATGCACCAGATACACTAATTTTCTTCTGGACACCAAGGGAAAACTCTATCGTTGGCGGTCTCCCGTCATCGTAGAAAAAGGGGGTAAAGTTGAGGTCGAAGGCCACCTCATCGACCTCAAGAAAGTTGTGCTTGATGGTTCCGCGGCGACCCCTATAACCAAAGTTTCAGCGGAACAATGGGGTCGTCCTTAG

>CF-2baoyu15jianxueqing20250812

ATGTTGGAGAAATGCTTGACCGCGGGCTGTTGCTCGCAATTGCTTTCTTTGTGGTGTATCGTGCCGTTCTGTTTTGCTGTGCTCGCCAACGCCAGCAACGACAGCAGCTCCCATCTACAGCTGATTTACAACTTGACGCTATGTGAGCTGAATGGCACAGATTGGCTAGCTAACAAATTTGATTGGGCAGTGGAGAGTTTTGTCATCTTTCCCGTTTTGACTCACATTGTCTCCTATGGTGCCCTCACTACCAGCCATTTCCTTGACACAGTCGCTTTAGTCACTGTGTCTACCGCCGGGTTTGTTCACGGGCGGTATGTCCTAAGTAGCATCTACGCGGTCTGTGCCCTGGCTGCGTTGACTTGCTTCGTCATTAGGTTTGCAAAGAATTGCATGTCCTGGCGCTACGCGTGTACCAGATATACCAACTTTCTTCTGGACACTAAGGGCATACTCTATCGTTGGCGGTCGCCTGTCATCATAGAGAAAAGGGGCAAAGTTGAGGTCGAAGGTCATCTGATCGACCTCAAAAGAGTTGTGCTTGATGGTTCCGTGGCAACCCCTATAACCAGAGTTTCAGCGGAACAATGGGGTCGTCCTTAG

>CF-25yiquchanfangduanweiye20250814

ATGTTGGGGAGATGCTTGACCGCGGGCTGTTGCTCGCAATTGCCTTTTTTGTGGTATATCGTGCCGTTCTGCTTTGTTGTGCTCGTCAACGCCAACAACAGCAGCAGCTCCCATTTACAGTTGATTTATAACCTAACGATATGTGAGCTGAACGGCACAGATTGGCTAAATAAAAGTTTTGATTGGGCAGTGGAGACCTTTGTTTTTTTCCCTGTGTTGACTCATATTGTTTCTTAGGGCGCCCTAACCACCAGCCATTTCCTTGACACAGTTGGCCTGATCACCGTGTCTACCGCTGGATATTATCACGAGCGGTATGTCCTGAGCAGCATTTACGCCCCCTGCGCCCTAGCTGCGTTAACTTGCTTTGTCATCAGGCTAACAAAAAATTGCATGTCCGGGCGTTACTCATGCACCAGATACACTAACTTTCTTCTGGACACCAAGGGCAAACTCTATCGCTGGCGGTCTCCCGTCATCGTGGAGAAAGGGGGTAAAGTTGAGGTCGAAGGCCACCTGATCGACCTCAAGAGAGTTGTGCTTGACGGTTCCGCGGCGACCCCTATAACCAAAGTTTCAGCGGAACAATGGGGTCGTCCTTAG

>CF-6baoyuxueqing20250820

ATGTCGGGGAAATGCTTGACCGCGGGCTGTTGCTCGCAATTGCCTTTTTTGTGGTATATCGTGCCGTTCTGTTTTGTTGTGCCCGTCAACGCCAACAACAGCAGCAGCTCCCATTTACAGTTGATTTATAACCTGACGATATGTGAGCTGAATGGCACAGATTGGCTAAATAAGAGTTTTGATTGGGCAGTGGAGACCTTTGTTATCTTCCCTGTGTTGACTCATATTGTCTCCTATGGCGCCCTAACCACCAGCCATTTCCTTGACACAGTTGGCCTGATCACCGTGTCTACCGCTGGGTATTGCCACGGGCGGTATGTCCTGAGCAGCATTTACGCCGTCTGCGCCCTAGCTGCGTTAACTTGCTTTGTCATCAGGCTAACAAAAAATTGCATGTCCTGGCGTTACTCATGCACCAGATACACCAACTTTCTTCTGGACACCAAGGGCAAACTCTATCGTTGGCGGTCTCCCGTCATCGTGGAGAAAGGGGGTAAAGTTGAGGTCGCAGGCCACCTGATCGACCTCAAGAGAGTTGTGCTTGACGGTTCCGCGGCAACCCCTATAACCAAAGTTTCAGCGGAACAATGGGGTCGTCTTTAG

>CF-12baoyuxueqing20250819

ATGTTGGGGAAATGCTTGACCGCGGGCTGCTGCTCGCAATTGCCTTTTTTGTGGTTTATCGTGCCGTTCTGTTTAGTTGCGCTCGTCAACGCCAACGACAACAGCAGCTCCCACTTACAATTGATTTATAACCTAACGATATGTGAGCTGAATGGCACAGATTGGCTGAAAGACCACTTTTCTTGGGCGGTGGAGACTTTCGTTATTTTTCCTGTGTTAACTCATATTGTCTCTTACGGTGCCCTCACCACTAGCCATTTTCTTGACACGGTCGGCCTTATCACTGTGTCCACCGCCGGGTATTATCACGGGCGGTATGTATTAAGTAGCATCTACGCTGTCTGCGCCCTAGCTGCGCTTGTTTGCTTCGCCATTAGGTTGGTAAAGAATTGCATGTCCTGGCGCTACTCTTGCACCAGATATACTAATTTTCTTCTGGACACTAAGGGAAAACTCTATCGTTGGCGGTCACCCGTCATCATAGAGAAGGGGGGCAAGGTGGATGTTGGTGGTCATCTAATCGACCTAAAGAGAGTTGTGCTTGAAGGTTCCGCGGCAACCCCTGTAACCAAGATTTCAGCGGAACAATGGGGTCGTCCATAG

>CF-10baoyu9jianzuzhi20250821

ATGTCGGGGAAATGCTTGACCGCGGGCTGTTGCTCGCAATTGCTTTTTTTGTGGTATATCGTGCCGTTCTGTTTTGTTGTGCCCGTCAACGCCAACAACAGCAGCAGCTCCCATTTACAGTTGATTTATAACCTGACGATATGTGAGCTGAATGGCACAGATTGGCTAAATAAAAGTTTTGATTGGGCAGTGGAGACCTTTGTTATTTTCCCTGTGTTGACTCATATTGTCTCCTATGGCGCCCTAACCACCAGCCATTTCCTTGACACAGTTGGCCTGATCACCGTGTCTACCGCTGGGTATTACCACGGGCGGTATGTCCTGAGCAGCATCTACGCCGTCTGCGCCCTAGCTGCGTTAACTTGCTTTGTCATCAGGCTAACAAAAAATTGCATGTCCTGGCGTTACTCATGCACCAGATACACTAACTTTCTTCTGGACACCAAGGGCAAACTCTATCGTTGGCGGTCTCCCGTCATCGTGGAGAAAGGGGGTAAAGTTGAGGTCGCAGGCCACCTGATCGACCTCAAGAGAGTTGTGCTTGACGGTTCCGCGGCAACCCCTATAACCAAAGTTTCAGCGGAACAATGGGGTCGTCCTTAG

>CF-10baoyu12jianzuzhi20250821

ATGTTGGGGAAATGCTTGACCGCGGGCTGCTGCTCGCAATTGCCTTTTTTGTGGTGTATCGTGCCGTTCTGTTTAGTTGCGCTCGTCAACGCCAACGAAAACAGCAGCTCCCACTTACAATTGATTTATAACCTAACGATATGTGAGCTGAATGGCACAGATTGGCTGAATGAACACTTTTCTTGGGCGGTGGAGACTTTCGTCATTTTTCCCGTGTTGACTCATATTGTCTCTTACGGTGCCCTCACCACTAGCCATTTTCTTGACACGGTCGGTCTTATCACTGTGTCCACCGCCGGGTATTACCACGGGCGGTATGTATTGAGTAGCATCTACGCTGTCTGCGCCCTAGCTGCGCTTGTTTGCTTCGCCATTAGGTTGGTAAAAAATTGCATGTCCTGGCGCTACTCTTGCACCAGATATACTAATTTTCTTCTGGACACTAAGGGAAAACTCTATCGTTGGCGGTCACCCGTCATCATAGAGAAGGGGGGCAAGGTGGATGTTGGTGGTCATTTAATCGACCTAAAGAGAGTTGTGCTTGAAGGTTCCGCGGCAACCCCTGTAACCAAAATTTCAGCGGAACAATGGGGTCGTCCATAG

>CF-26erqubaoyuxueqing20250824

ATGTTGGAGAAATGCTTGACCGCGGGCTGTTGCTCGCAATTGCTTTCTTTGTGGTGTATCGTGCCGTTCTGTTTTGCTGTGCTCGCCAACGCCAGCAACGACAGCAGCTCCCATCTACAGCTGATTTACAACTTGACGCTATGTGAGCTGAATGGCACAGATTGGCTAGCTAACAAATTTGATTGGGCAGTGGAGAGTTTTGTCATCTTTCCCGTTTTGACTCACATTGTCTCCTATGGTGCCCTCACTACCAGCCATTTCCTTGACACAGTCGCTTTAGCCACTGTGTCTACCGCCGGGTTTGTTCACGGGCGGTATGTCCTAAGTAGCATCTACGCGGTCTGTGCCCTGGCTGCGTTGACTTGCTTCGTCATTAGGTTTGCAAAGAATTGCATGTCCTGGCGCTACGCGTGTACCAGATACACCAACTTTCTTCTGGACACTAAGGGCATACTCTATCGTTGGCGGTCGCCTGTCATCATAGAGAAAAGGGGCAAAGTTGAGGTCGAAGGTCATCTGATCGACCTCAAAAGAGTTGTGCTTGATGGTTCCGTGGCAACCCCTATAACCAGAGTTTCAGCGGAACAATGGGGTCGTCCTTAG

>CF-17erqufenmianzizhuxueqing20250825

ATGTTGGAGAAATGCTTGACCGCGGGCTGTTGCTCGCAATTGCTTTCTTTGTGGTGTATCGTGCCGTTCTGTTTTGCTGTGCTCGCCAACGCCAGCAACGACAGCAGCTCCCATCTACAGCTGATTTACAACTTGACGCTATGTGAGCTGAATGGCACAGATTGGCTAGCTAACAAATTTGATTGGGCAGTGGAGAGTTTTGTCATCTTTCCCGTTTTGACTCACATTGTCTCCTATGGTGCCCTCACTACCAGCCATTTCCTTGACACAGTCGCTTTAGTCACTGTGTCTACCGCCGGGTTTGTTCACGGGCGGTATGTCCTAAGTAGCATCTACGCGGTCTGTGCCCTGGCTGCGTTGACTTGCTTCGTCATTAGGTTTGCAAAGAATTGCATGTCCTGGCGCTACGCGTGTACCAGATATACCAACTTTCTTCTGGACACTAAGGGCGGACTCTATCGTTGGCGGTCGCCTGTCATCATAGAGAAAAGGGGCAAAGTTGAGGTCGAAGGTCATCTGATCGACCTCAAAAGAGTTGTGCTTGATGGTTCCGTGGCAACCCCTATAACCAGAGTTTCAGCGGAACAATGGGGTCGTCCTTAG

>CF-13fenmianzizhuxueqing20250827

ATGTTGGGGAAATGCTTGACCGCGGGCTGCTGCTCGCAATTGCCTTTTTTGTGGTGTATCGTGCCGTTCTGTTTAGTTGCGCTCGTCAACGCCAACAACGACAGCAGCTCCCACTTACAATTGATTTATAACCTAACGATATGTGAGCTGAATGGCACAGATTGGCTGAACAAACACTTTCCTTGGGCGGTGGAGACTTTCGTTATTTTTCCTGTGTTAACGCATGTTGTCTCTTACGGTGCCCTCACCACTAGCCATTTTCTTGACACGGTCGGTCTTATCACTGTGTCCACCGCCGGGTATTATCACGGGCGGTATGTATTGAGTAGCATCTACGCTGTCTGCGCCCTAGCTGCGCTTGTTTGCTTCGCCATTAGGTTGGTAAAGAATTGCATGTCCTGGCGCTACTCTTGCACCAGATATACTAATTTTCTTCTGGACACAAAGGGAAAACTCTATCGTTGGCGGTCACCCGTCATCATAGAGAAGGGGGGCAAGGTGGATGTTGGGGGTCATTTAATCGACCTAAAGAGAGTTGTGCTTGAAGGTTCCGCGGCAACCCCTGTAACCAAGATTTCAGCGGAACAATGGGGTCGTCCATAG

>CF-21erqupeihuaimuzhu20250906

ATGTTGGGGAAATGCTTGACCGCGGGCTGCTGCTCGCAATTGCCTTCTTTGTGGTGTATCGTGCCGTTCTGTTTAGTTGCGCTCGTCAACGCCAACCACGACAGCAGCTCCCACTTACAATTGATTTATAACCTAACGATATGTGAGCTGAATGGCACAGATTGGCTGAAAGAACACTTTCCTTGGGCGGTGGAGACTTTCGTTATTTTTCCTGTGTTAACTCATGTTGTCTCTTACGGTGCCCTCACCACTAGCCATTTTCTTGACACGGTCGGTCTTATCACTGTGTCCACCGCCGGGTATTATCACGGGCGGTATGTACTGAGTAGCATCTACGCTGTCTGCGCCCTAGCTGCGCTTGCTTGCTTCGCCATTAGGTTGGTAAAGAATTGCATGTCCTGGCGCTACTCTTGCACCAGATATACTAATTTTCTTCTGGACACCAAGGGAAAACTCTATCGTTGGCGGTCACCCGTCATCATAGAGAAGGGGGGCAAGGTGGATGTTGGGGGTCATTTAATCGACCTAAAGAGAGTTGTGCTTGAAGGTTCCGCGGCAACCCCTGTAACCAAGATTTCAGCGGAACAATGGGGTCGTCCATAG

>CF-11fenmian13jianzizhuzuzhi20250917

ATGTCGGGGAAATGCTTGACCGCGGGCTGTTGCTCGCAATTGCCTTTTTTGTGGTATATCGTGCCGTTCTGTTTTGTTGTGCTCGTCAACGCCAACAACAGCAGCAGCTCCCATTTACAGTTGATTTACAACCTGACGATATGTGAGCTGAATGGCACAGATTGGCTAAATAGAAGTTTTGATTGGGCAGTGGAGACCTTTGTTATTTTCCCTGTGTTGACTCATATTGTCTCCTATGGCGCCCTAACCACCAGCCATTTTCTTGACACAGTTGGCCTGATCACCGTGTCTACCGCTGGGTATTACCACGGGCGGTATGTCCTGAGCAGCATTTACGCCGTCTGCGCCCTAGCTGCGTTAACTTGCTTTGTCATCAGGCTAACAAAAAATTGCATGTCCTGGCGTTACTCATGCACCAGATACACTAACTTTCTTCTGGACACCAAGGGCAAACTCTATCGTTGGCGGTCTCCCGTCATCGTGGAGAAAGGGGGTAAAGTTGAGGTCGCAGGCCACCTGATCGACCTCAAGAGAGTTGTGCTTGACGGTTCCGCGGCAACCCCTATAACCAAAGTTTCAGCGGAACAATGGGGTCGTCCTTAG

>CF-11fenmian7jianzizhuzuzhi20250917

ATGTTGGGGAAATGCTTGACCGCGGGTTATTGCTCACAATCGGTTTTTTTGTGGTGTATCGTGCCATTCTGTTTTGCTGTGCTCGTCAACGCCAGCGGCAACAGCAGCTCTCACTTACAATTGATCTATAACCTGACGATATGTGAGCTGAATGGCACAGACTGGTTGAACCAAAGATTTGACTGGGCAGTGGAGACTTTCGTCATTTTTCCTGTCTTGACCCATATCGTCTCTTACGGCGCCCTTACCACTAGCCATTTTCTTGACACGGTCGGCCTAATCACCGTGTCCACCGCTGGGTATTATCATAAGCGGTATGTATTGAGTAGCATCTACGCTGTCTGTGCCCTAGCCGCGTTGGTTTGCTTCGCCATTAGGTTGGCAAAAAATTGTATGTCCTGGCGCTACTCATTCACCAGATATACTAATTTTCTTCTGGACTCTAAGGGCAAACTCTACCGTTGGCGGTCACCCGTCATCATAGAGAAAGAGGGCAAAGTTGATGTGGGGGGTCAGTTAATCGACCTCAAGAGAGTTGTGCTTGATGGTTCCGCGGCGACCCCTGTAACCAAGATTTCAGCGGAACAATGGGGTCGTCCATAG

>CF-8yiqubaoyuzhuxueqing20250918

ATGTTGGAGAAATGCTTGACCGCGGGCTGTTGCTCGCAATTGCTTTCTTTGTGGTGTATCGTGCCGTTCTGTTTTGCTGTGCTCGCCAACGCCAGCAACGACAGCAGCTCCCATCTACAGCTGATTTACAACTTGACGCTATGTGAGCTGAATGGCACAGATTGGCTAGCTAACAAATTTGATTGGGCAGTGGAGAGTTTTGTCATCTTTCCCGTTTTGACTCACATTGTCTCCTATGGTGCCCTCACTACTAGCCATTTCCTTGACACAGTCGCTTTAGTCACTGTGTCTACCGCCGGGTTTGTTCACGGGCGGTATGTCCTAAGTAGCATCTACGCGGTCTGTGCCCTGGCTGCGTTGACTTGCTTCGTCATTAGGTTTGCAAAGAATTGCATGTCCTGGCGCTACGCGTGTACCAGATATACCAACTTTCTTCTGGACACTAAGGGCAGACTCTATCGTTGGCGGTCGCCTGTCATCATAGAGAAAAGGGGCAAAGTTGAGGTCGAAGGTCATCTGATCGACCTCAAAAGAGTTGTGCTTGATGGTTCCGTGGCAACCCCTATAACCAGAGTTTCAGCGGAACAATGGGGTCGTCCTTAG

>CF-7houbeimuzhukouqiangye20250916

ATGTTGGGGAAATGCTTGACCGCGGGCTGCTGCTCGCAATTGCCTTTTTTGTGGTGTATCGTGCCGTTCTGTTTAGTTGCGCTCGTCAACGCCAACGACAACAGCAGCTCCCACTTACAATTGATTTATAACCTAACGATATGTGAGCTGAATGGCACAGATTGGCTGAAAGAACACTTTTCTTGGGCGGTGGAGACTTTCGTTATTTTTCCTGTGTTAACTCATGTTGTCTCTTACGGTGCCCTCACCACTAGCCATTTTCTTGACACGGTCGGTCTTATCACTGTGTCCACCGCCGGGTATTATCACGGGCGGTATGTATTGAGTAGCATCTACGCTGTCTGCGCCCTAGCTGCGCTTGTTTGCTTCGCCATTAGGTTGGTAAAGAATTGCATGTCCTGGCGCTACTCTTGCACCAGATATACTAATTTTCTTCTGGACACTAAGGGAAAACTCTATCGTTGGCGGTCACCCGTCATCATAGAGAAGGGGGGCAAGGTGGATGTTGGGGGTCATTTAATCGACCTAAAGAGAGTTGTGCTTGAAGGTTCCGCGGCAACCCCTGTAACCAAGATTTCAGCGGAACAATGGGGTCGTCCATAG

>CF-7fenmian1dongzizhutaiyixue20250918

ATGTTGGGGAAATGCTTGACCGCGGGCTGCTGCTCGCAATTGCTTTTTTTGTGGTGTATCGTGCCGTTCTGTTTAGTTTCGCTCGTCAACGCCAGCGACAGCAGCAGCTCCCATTTACAGTTGATTTATAACCTGACGATATGTGAGCTGAATGGCACAGATTGGCTAAATCGCACTTTTGATTGGGCAGTGGAGACCTTCGTTATTTTTCCTGTGTTGACTCATATCGTCTCCTATGGCGCCCTAACCACCAGCCACTTCCTTGACACAGTCGGCCTGATCACCGTGTCTACTGCTGGATATTACCACGGGCGGTATGTCTTGAGCAGTATTTACGCCGTCTGCGCCTTAGCTGCGCTAATTTGCTTCGTCATCAGGCTAACAAAAAATTGCATGTCCTGGCGTTACTCATGCACCAGATACACTAATTTTCTTCTGGACACCAAGGGAAAACTCTATCGTTGGCGGTCTCCCGTCATCGTGGAAAAAGGGGGTAAAGTTGAGGTCGAAGGCCACCTCATCGACCTCAAGAAAGTTGTGCTTGATGGTTCCGCGGCGACCCCTATAACCAAAGTTACAGCGGAACAATGGGGTCGTCCTTAG

>CF-25yiqufenmianzizhuduanweiye20250913

ATGTTGGGGAAATGCTTGACCGCGGGTTGCTGCTCATCATTGCTTTTTTTGTGGTGTATCGTGCTATCTTGTTCTGTTGCGCTCGTCAACGCCGACAGCAACACCAGCTCTCATTTACAGTTGATTTATAACCTGACGATATGTGAGCTGAATGGCACGGATTGGTTGGACAAAAAATTTGACTGGGCAGTAGAGACTTTCGTCATTTTTCCTGTTCTAACCCATATTGTCTCTTACGGCGCCCTCACCACTAGCCATTTTCTTGACATGGTCGGCCTAATTACTGTGTCCACCGCTGGGTATTACCATGAGCGGTATGTATTGAGTAGCATCTATGCTGTCTGTGCCCTGGCCGCGTTGGCTTGCTTCGCCATCAGGTTGGCGAAAAATTGCATGTCCTGGCGCTACTCATGCACCAGATACACCAACTTTCTCCTGGACACTAAAGGCAGACTCTACCGCTGGCGGTCACCCGTCATCATAGAGAAGGGGGGCAAAGTTGATGTGGGGGGTCATTTAATTGACCTCAAGAGAGTCGTGCTTGATGGTTCCGCGGCGACCCCTATAACCAAGATTTCAGCGGAACAATGGGGTCGTCCATAG

>CF-18baoyu12dongzuzhi20250927

ATGTTGGGGAAATGCTTGACCGCGGGCTGCTGCTCGCAATTGCTTTTTTTGTGGTGTATCGTGCCGTTCTGTTTAGTTGCGTTCGTCAACGCCAACAACAGCAGCAGCTCCCACTTACAGTTGATTTATAACCTAACGATATGTGAGCTGAATGGCACAGATTGGCTGAATGAACATTTTTCTTGGGCAGTGGAGACTTTCGTTATTTTTCCTGTGTTAACTCATATTGTCTCTTACGGTGCCCTCACCACTAGCCATTTTCTTGACACGGTCGGCCTAATCACTGTGTCCACCGCCGGGTATTATCACGGGCGGTATGTATTGAGTAGCATCTACGCTGTCTGCGCCCTAGCTGCGCTTGTTTGCTTCGCCATTAGGTTGGTAAAAAATTGCATGTCCTGGCGCTACTCTTGCACCAGACATACTAATTTCCTTCTGGACACTAAGGGAAAACTCTATCGTTGGCGGTCACCCGTCATCATAGAGAAAGGGGGCAAGGTGGATGTTGGGGGCCATTTAATTGACCTGAAGAGAGTTGTGCTTGAAGGTTCCGCGGCGACTCCTGTAACCAAGATTTCAGCGGAACAATGGGGTCGTCCATAG

>CF-7houbeizhukouqiangye20250921

ATGTTGGGGAAATGCTTGACCGCGGGCTGCTGCTCGCAATTGCCTTTTTTGTGGTGTATCGTGCCGTTCTGTTTAGTTGCGCTCGTCAACGCCAACGACAACAGCAGCTCCCACTTACAATCGATTTATAACCTAACGATATGTGAGCTGAATGGCACAGATTGGCTGAAAGAACACTTTTCTTGGGCGGTGGAGACTTTCGTTATTTTTCCTGTGTTAACTCATGTTGTCTCTTACGGTGCCCTCACCACTAGCCATTTTCTTGACACGGTCGGTCTTATCACTGTGTCCACCGCCGGGTATTATCACGGGCGGTATGTATTGAGTAGCATCTACGCTGTCTGCGCCCTAGCTGCGCTTGTTTGCTTCGCCATTAGGTTGGTAAAGAATTGCATGTCCTGGCGCTACTCTTGCACCAGATATACTAATTTTCTTCTGGACACTAAGGGAAAACTCTATCGTTGGCGGTCACCCGTCATCATAGAGAAGGGGGGCAAGGTGGATGTTGGGGGTCATTTAATCGACCTAAAGAGAGTTGTGCTTGAAGGTTCCGCGGCAACCCCTGTAACCAAGATTTCAGCGGAACAATGGGGTCGTCCATAG

>CF-15fenmian105taiyixue20250921

ATGTTGGGGAAATGCTTGACCGCGGGCTGCTGCTCGCAATTGCTTTTTTTGTGGTGTATCGTGCCGTTCTGTTTAGTTTCGCTCGTCAACGCCAGCTACAGCAGCAGCTCCCATTTACAGTTGATTTATAACCTGACGATATGTGAGCTGAATGGCACGGATTGGCTGGATCGCACTTTTGATTGGGCAGTGGAGACCTTCGTTATTTTTCCTGTGTTGACTCATATCGTCTCCTACGGCGCCCTAACCACCAGCCACTTCCTTGACACAGTCGGCCTGATCACCGTGTCTACTGCTGGATATTACCACGGGCGGTATGTCTTGAGCAGTATTTACGCCGTTTGCGCCTTAGCTGCGCTAATTTGCTTCGTCATCAGGCTAACAAAAAATTGCATGTCCTGGCGTTACTCATGCACCAGATACACTAATTTTCTTCTGGACACCAAGGGAAAACTCTATCGTTGGCGGTCTCCCGTCATCGTGGAAAAAGGGGGTAAAGTTGAGGTCGAAGGCCACCTCATCGACCTCAAGAAAGTTGTGCTTGATGGTTCCGCGGCGACCCCTATAACCAAAGTTACAGCGGAACAATGGGGTCGTCCTTAG

>CF-15fenmian314taiyixue20250921

ATGTTGGGGAAATGCTTGACCGCGGGCTGCTGCTCGCAATTGCTTTTTTTGTGGTGTATCGTGCCGTTCTGTTTAGTTTCGCTCGTCAACGCCAGCAACAACAGCAGCTCCCATTTACAGTTGATTTATAACCTGACGATATGTGAGCTGAATGGCACGGATTGGCTGAATCGCACTTTTGATTGGGCAGTGGAGACCTTCGTTATTTTTCCTGTGTTGACTCATATCGTCTCCTACGGCGCCCTAACCACCAGCCACTTCCTTGACACAGTCGGCCTGATCACCGTGTCTACTGCTGGATATTACCACGGGCGGTATGTCTTGAGCAGTATTTACGCCGTCTGCGCCTTAGCTGCGCTAATTTGCTTCGTCATCAGGCTAACAAAAAATTGCATGTCCTGGCGTTACTCATGCACCAGATACACTAATTTTCTTCTGGACACCAAGGGAAAACTCTATCGTTGGCGGTCTCCCGTCATCGTGGAAAAAGGGGGTAGAGTTGAGGTCGAAGGCCACCTCATCGACCTCAAGAAAGTTGTGCTTGATGGTTCCGCGGCGACCCCTATAACCAAAGTTACAGCGGAACAATGGGGTCGTCCTTAG

>CF-11peihuai2dongliuchanzhuweigenxue20250923

ATGTTGGGGAAATGCTTGACCGCGGGCTGCTGCTCGCAATTGCCTTTTTTGTGGTTTATCGTGCCGTTCTGTTTAGTTGCGCTCGTCAACGCCAACGACAACAGCAGCTCCCACTTACAATTGATTTATAACCTAACAATATGTGAGCTGAATGGCACAGATTGGCTGAAAGACCACTTTTCTTGGGCGGTGGAGACTTTCGTTATTTTTCCTGTGTTAACTCATATTGTCTCTTACGGTGCCCTCACCACTAGCCATTTTCTTGACACGGTCGGTCTTATCACTGTGTCCACCGCCGGGTATTATCACGGGCGGTATGTATTGAGTAGCATCTACGCTGTCTGCGCCCTAGCTGCGCTTGTTTGCTTCGCCATTAGGTTGGTAAAGAATTGCATGTCCTGGCGCTACTCTTGCACCAGATATACTAATTTTCTTCTGGACACTAAGGGAAAACTCTATCGTTGGCGGTCACCCGTCATCATAGAGAAGGGGGGCAAGGTGGATGTTGGTGGTCATTTAATCGACCTAAAGAGAGTTGTGCTTGAAGGTTCCGCGGCAACCCCTGTAACCAAGATTTCAGCGGAACAATGGGGTCGTCCATAG

>CF-15peihuai3dongliuchanzhu20250927

ATGTTGGGGAAATGCTTGACCGCGGGCTGCTGCTCGCAATTGCCTTTTTTGTGGTGTATCGTGCCGTTCTGTTTAGTTGCGCTCGTCAACGCCAACATCGACAGCAGCTCCCACTTACAATTGATTTATAACCTAACGATATGTGAGCTGAATGGCACAGATTGGCTGAACAAACACTTTCCTTGGGCGGTGGAGACTTTCGTTATTTTTCCTGTGTTAACGCATGTTGTCTCTTGTGGTGCCCTCACCACTAGCCATTTTCTTGACACGGTCGGTCTTATCACTGTGTCCACCGCCGGGTATTATCACGGGCGGTATGTATTGAGTAGCATCTACGCTGTCTGCGCCCTAGCTGCGCTTGTTTGCTTCGCCATTAGGTTGGTAAAGAATTGCATGTCCTGGCGCTACTCTTGCACCAGATATACTAATTTTCTTCTGGACACAAAGGGAAAACTCTATCGTTGGCGGTCACCCGTCATCATAGAGAAGGGGGGCAAGGTGGATGTTGGGGGTCATTTAATCGACCTAAAGAGAGTTGTGCTTGAAGGTTCCGCGGCAACCCCTGTAACCAAGATTTCAGCGGAACAATGGGGTCGTCCATAG

>CF-11peihuai2dongliuchanzhuweigenxue20250928

ATGTTGGGGAAATGCTTGACCGCGGGCTGCTGCTCGCAATTGCCTTTTTTGTGGTTTATCGTGCCGTTCTGTTTAGTTGCGCTCGTCAACGCCAACGACAACAGCAGCTCCCACTTACAATTGATTTATAACCTAACAATATGTGAGCTGAATGGTACAGATTGGCTGAAAGACCACTTTTCTTGGGCGGTGGAGACTTTCGTTATTTTTCCTGTGTTAACTCATATTGTCTCTTACGGTGCCCTCACCACTAGCCATTTTCTTGACACGGTCGGTCTTATCACTGTGTCCACCGCCGGGTATTATCACGGGCGGTATGTATTGAGTAGCATCTACGCTGTCTGCGCCCTAGCTGCGCTTGTTTGCTTCGCCATTAGGTTGGTAAAGAATTGCATGTCCTGGCGCTACTCTTGCACCAGATATACTAATTTTCTTCTGGACACTAAGGGAAAACTCTATCGTTGGCGGTCACCCGTCATCATAGAGAAGGGGGGCAAGGTGGATGTTGGTGGTCATTTAATCGACCTAAAGAGAGTTGTGCTTGAAGGTTCCGCGGCAACCCCTGTAACCAAGATTTCAGCGGAACAATGGGGTCGTCCATAG

>CF-23erqufenmianduanweiye20250928

ATGTTGGGGAAATGCTTGACCGCGGGCTGCTGCTCGCAATTGCTTTTTTTGTGGTGTATCGTGCCGTTCTGTTTAGTTTCGCTCGTCAACGCCAGCAACAGCAGCAGCTCCCATTTACAGTTGATTTATAACCTGACGATATGTGAGCTGAATGGCACAGATTGGCTAAATCGCACTTTTGATTGGGCAGTGGAGACCTTCGTTATTTTTCCTGTGTTGACTCATATCGTCTCCTATGGCGCCCTAACCACCAGCCACTTCCTTGACACAGTCGGCCTGATCACCGTGTCTACTGCTGGATATTACCACGGGCGGTATGTCTTGAGCAGTATTTACGCCGTCTGCGCCTTAGCTGCGCTAATTTGCTTCGTCATCAGGCTAACAAAAAATTGCATGTCCTGGCGTTACTCATGCACCAGATACACTAATTTTCTTCTGGACACCAAGGGAAAACTCTATCGTTGGCGGTCTCCCGTCATCGTGGAAAAAGGGGGTAAAGTTGAGGTCGAAGGCCACCTCATCGACCTCAAGAAAGTTGTGCTTGATGGTTCCGCGGCGACCCCTATAACCAAAGTTATAGCGGAACAATGGGGTCGTCCTTAG

>CF-15fenmiantaiyixue20250928

ATGTTGGGGAAATGCTTGACCGCGGGCTGCTGCTCGCAATTGCTTTTTTTGTGGTGTATCGTGCCGTTCTGTTTAGTTTCGCTCGTCAACGCCAGCAACAGCAGCAGCTCCCATTTACAGTTGATTTATAACCTGACGATATGTGAGCTGAATGGCACGGATTGGCTGAATCGCAATTTTGATTGGGCAGTGGAGACCTTCGTTATTTTTCCTGTGTTGACTCATATCGTCTCCTACGGCGCCCTAACCACCAGCCACTTCCTTGACACAGTCGGCCTGATCACCGTGTCTACTGCTGGATATTGCCACGGGCGGTATGTCTTGAGCAGTATTTACGCCGTCTGCGCCTTAGCTGCGCTAATTTGCTTCGTCATCAGGCTAACAAAAAATTGCATGTCCTGGCGTTACTCATGCACCAGATACACTAATTTTCTTCTGGACACCAAGGGAAAACTCTATCGTTGGCGGTCTCCCGTCATCGTGGAAAAAGGGGGTAAAGTTGAGGTCGAAGGCCACCTCATCGACCTCAAGAAAGTTGTGCTTGATGGTTCCGCGGCGACCCCTATAACCAAAGTTACAGCGGAACAATGGGGTCGTCCTTAG

>CF-15peihuai3dongliuchanzhu20250927(2)

ATGTTGGGGAAATGCTTGACCGCGGGCTGCTGCTCGCAATTGCCTTTTTTGTGGTGTATCGTGCCGTTCTGTTTAGTTGCGCTCGTCAACGCCAACAACGACAGCAGCTCCCACTTACAATTGATTTATAACCTAACGATATGTGAGCTGAATGGCACAGATTGGCTGAACAAACACTTTCCTTGGGCGGTGGAGACTTTCGTTATTTTTCCTGTGTTAACGCATGTTGTCTCTTATGGTGCCCTCACCACTAGCCATTTTCTTGACACGGTCGGTCTTATCACTGTGTCCACCGCCGGGTATTATCACGGGCGGTATGTATTGAGTAGCATCTACGCTGTCTGCGCCCTAGCTGCGCTTGTTTGCTTCGCCATTAGGTTGGTAAAGAATTGCATGTCCTGGCGCTACTCTTGCACCAGATATACTAATTTTCTTCTGGACACAAAGGGAAAACTCTATCGTTGGCGGTCACCCGTCATCATAGAGAAGGGGGGCAAGGTGGATGTTGGGGGTCATTTAATCGACCTAAAGAGAGTTGTGCTTGAAGGTTCCGCGGCAACCCCTGTAACCAAGATTTCAGCGGAACAATGGGGTCGTCCATAG

>CF-17erqufenmian116jianweigenxue20251001

ATGTTGGGGAAATGCTTGACCGCGGGTTACTGCTCATCATTGCTTTTTTTGTGGTGTATCGTGCTATCCTGTTCTGTTGCGCTCGTCAACGCCAACAGCAACACCAGCTCTCATTTACAGTTGATTTATAACCTGACGATATGTGAGCTGAATGGCACGGATTGGTTGAATGAAAGATTTGACTGGGCAGTGGAGACTTTCGTCATTTTTCCTGTTCTGACCCATATTGTCTCGTACGGTGCCCTCACCACTAGCCATTTTCTTGACACGGTCGGTCTAATCACTGTGTCCACCGCTGGGTATTACCATAAGCGGTATGTATTGAGTAGCATCTATGCTGTCTGTGCCCTAGCCGCGCTGGCTTGCTTCGCCATCAGGTTGGCGAAAAATTGCATGTCCTGGCGCTACTCATGCACCAGATACACCAACTTTCTCCTTGACACTAAAGGCAAACTCTACCGCTGGCGGTCACCCGTCATCATAGAGAAGGGGGGCAAAGTTGATGTGGGGGGTCATTTAATTGACCTCAAGAGAGTCGTGCTTGATGGTTCCGCGGCGACCCCTATAACCAAGATTTCAGCGGAACAATGGGGTCGTCCATAG

>CF-7peihuai4dongliuchanzuzhi20251005

ATGTTGGGGAAATGCTTGACCGCGGGCTGCTGCTCGCAATTGCTTTTTTTGTGGTGTATCGTGCCGTTCTGTTTAGTTTCGCTCGTCAACGCCAGCAACAGCAGCAGCTCCCATTTACAGTTGATTTATAACCTGACGATATGTGAGCTGAATGGCACAGATTGGCTAAATCGCACTTTTGATTGGGCAGTGGAGACCTTCGTTATTTTTCCTGTGTTGACTCATATCGTCTCCTATGGCGCCCTAACCACCAGCCACTTCCTTGACACAGTCGGCCTGATCACCGTGTCTACTGCTGGATATTACCACGGGCGGTATGTCTTGAGCAGTATTTACGCCGTCTGCGCCTTAGCTGCGCTAATTTGCTTCGTCATCAGGCTAACAAAAAATTGCATGTCCTGGCGTTACTCATGCACCAGATACACTAATTTTCTTCTGGACACCAAGGGAAAACTCTATCGTTGGCGGTCTCCCGTCATCGTGGAAAAAGGGGGTAAAGTTGAGGTCGAAGGCCACCTCATCGACCTCAAGAAAGTTGTGCTTGATGGTTCCGCGGCGACCCCTATAACCAAAGTTACAGCGGAACAATGGGGTCGTCCTTAG

>CF-11fenmianduanweiye20251005

ATGTTGGGGAAATGCTTGACCGCGGGCTGCTGCTCGCAATTGCCTTTTTTATGGTTTATCGTGCCGTTCTGTTTAGTTGCGCTCGTCAACGCCAACGACAACAGCAGCTCCCACTTACAATTGATTTATAACCTAACGATATGTGAGCTGAATGGCACAGATTGGCTGAAAGACCACTTTTCTTGGGCGGTGGAGACTTTCGTTATTTTTCCTGTGTTAACTCATATTGTCTCTTACGGTGCCCTCACCACTAGCCATTTTCTTGACACGGTCGGCCTTATCACTGTGTCCACCGCCGGGTATTATCACGGGCGGTATGTATTGAGTAGCATCTACGCTGTCTGCGCCCTAGCTGCGCTTGTTTGCTTCGCCATTAGGTTGGTAAAGAATTGCATGTCCTGGCGCTACTCTTGCACCAGATATACTAATTTTCTTCTGGACACTAAGGGAAAACTCTATCGTTGGCGGTCACCCGTCATCATAGAGAAGGGGGGCAAGGTGGATGTTGGTGGTCATTTAATCGACCTAAAGAGAGTTGTGCTTGAAGGTTCCGCGGCAACCCCTGTAACCAAGATTTCAGCGGAACAATGGGGTCGTCCATAG

>CF-25yiqufenmiantaiyixue20251006

ATGTTGGGGAAATGCTTGACCGCGGGTTATTGCTCACAATCGGTTTTTTTGTGGTGTATCGTGCCATTCTGTTTTGCTGTGCTGGTCAACGCCAGGGGCAGCAGCAGTTCCCACTTACAATTGATCTATAACCTGACGATATGTGAGCTGAATGGCACAGACTGGTTGAACCAAAGATTTGACTGGGCAGTGGAGACTTTCGTCATTTTTCCTGTCTTGACCCATATCGTTTCTTACGGCGCCCTTACCACTAGCCATTTTCTTGACACGGTCGGCCTAATCACCGTGTCCACCGCTGGGTATTATCATAAGCGGTATGTATTGAGTAGCATTTACGCTGTCTGTGCCCTAGCCGCGTTGGTTTGCTTCGCCATTAGGTTGGCAAAAAATTGTATGTCCTGGCGCTACTCATTCACCAGATATACTAATTTTCTTCTGGACTCTAAGGGCAAACTCTACCGTTGGCGGTCACCCGTCATCATAGAGAAAGAGGGCAAAGTTGATGTGGGGGGTCAGTTAATCGACCTCAAGAGAGTTGTGCTTGATGGTTCCGCGGCGACCCCTGTAACCAAGATTTCAGCGGAACAATGGGGTCGTCCATAG

>CF-25yiqufenmiantaiyixue20251008

ATGTTGGGGAAATGCTTGACCGCGGGTTGCTGCTCATCATTGCTTTTTTTGTGGTGTATCGTGCTATCTTGTTCTGTTGCGCTCGTCAACGCCGACAGCAACACCAGCTCTCATTTACAGTTGATTTATAACCTGACGATATGTGAGCTGAATGGCACGGATTGGTTGGAAAAAAAATTTGACTGGGCAGTAGAGACTTTCGTCATTTTTCCTGTTCTAACCCATATTGTCTCTTACGGCGCCCTCACCACTAGCCATTTTCTTGACATGGTCGGCCTAATTACTGTGTCCACCGCTGGGTATTACCATAAGCGGTATGTATTGAGTAGCATCTATGCTGTCTGTGCCCTGGCCGCGTTGGCTTGCTTCGCCATCAGGTTGGCGAAAAATTGCATGTCCTGGCGCTACTCATGCACCAGATACACCAACTTTCTCCTGGACACTAAAGGCAGACTCTACCGCTGGCGGTCACCCGTCATCATAGAGAAGGGGGGCAAAGTTGATGTGGGGGGTCATTTAATTGACCTCAAGAGAGTCGTGCTTGATGGTTCCGCGGCGACCCCTATAACCAAGATTTCAGCGGAACAATGGGGTCGTCCATAG

>CF-7peihuai4K141dongtaiyixue20251007

ATGTTGGGGAGATGCTTGATCGCGGGCTGTTGCTCGCAATTGCCTTTTTTGTGGTATATCGTGCCGTTCTGCTTTGTTGTGCTCGTCAACGCCAACAACAGCAGCAGCTCCCATTTACAGTTGATTTATAACCTGACGATATGTGAGCTGAATGGCACAGATTGGCTAAATAAAAGTTTTGATTGGGCAGTGGAGACCTTTGTTATTTTCCCTGTGTTGACTCATATTGTCTCCTATGGCGCCCTAACCACCAGCCATTTCCTTGACACAGTTGGCCTGATCACCGTGTCTACCGCTGGATATTATCACGGGCGGTATGTCCTGAGCAGCATTTACGCCGCCTGCGCCCTAGCTGCGTTAACTTGCTTTGTCATCAGGCTAACAAAAAATTGCATGTCCTGGCGTTACTCATGCACCAGATACACTAACTTTCTTCTGGACACCAAGGGCAAACTCTATCGTTGGCGGTCTCCCGTCATCGTGGAGAAAGGGGGTAAAGTTGAGGTCGAAGGCCACCTGATCGACCTCAAGAGAGTTGTGCTTGACGGTTCCGCGGCGACCCCTATAACCAAAGTTTCAGCGGAACAATGGGGTCGTCCTTAG

>CF-7peihuai4KN93dongtaiyixue20251007

ATGTTGGGGAGATGCTTGATCGCGGGCTGTTGCTCGCAATTGCCTTTTTTGTGGTATATCGTGCCGTTCTGCTTTGTTGTGCTCGTCAACGCCAACGACAGCAGCAGCTCCCATTTACAGTTGATTTATAACCTGACGATATGTGAGCTGAATGGCACAGATTGGCTAAATAAAAGTTTTGATTGGGCAGTGGAGACCTTTGTTATTTTCCCTGTGTTGACTCATATTGTCTCCTATGGCGCCCTAACCACCAGCCATTTCCTTGACACAGTTGGCCTGATCACCGTGTCTACCGCTGGATATTATCACGGGCGGTATGTCCTGAGCAGCATTTACGCCGCCTGCGCCCTAGCTGCGTTAACTTGCTTTGTCATCAGGCTAACAAAAAATTGCATGTCCTGGCGTTACTCATGCACCAGATACACTAACTTTCTTCTGGACACCAAGGGCAAACTCTATCGTTGGCGGTCTCCCGTCATCGTGGAGAAAGGGGGTAAAGTTGAGGTCGAAGGCCACCTGATCGACCTCAAGAGAGTTGTGCTTGACGGTTCCGCGGCGACCCCTATAACCAAAGTTTCAGCGGAACAATGGGGTCGTCCTTAG

>CF-11peihuai3dongliuchanzhu20251008

ATGTTGGGGAAATGCTTGACCGCGGGCTGCTGCTCGCAATTGCCTTTTTTGTGGTTTATCGTGCCGTTCTGTTTAGTTGCGCTCGTCAACGCCAACGACAACAGCAGCTCCCACTTACAATTGATTTATAACCTAACAATATGTGAGCTGAATGGCACAGATTGGCTGAAAGACCACTTTTCTTGGGCGGTGGAGACTTTCGTTATTTTTCCTGTGTTAACTCATATTGTCTCTTACGGTGCCCTCACCACTAGCCATTTTCTTGACACGGTCGGTCTTATCACTGTGTCCACCGCCGGGTATTATCACGGGCGGTATGTATTGAGTAGCATCTACGCTGTCTGCGCCCTAGCTGCGCTTGTTTGCTTCGCCATTAGGTTGGTAAAGAATTGCATGTCCTGGCGCTACTCTTGCACCAGATATACTAATTTTCTTCTGGACACTAAGGGAAAACTCTATCGTTGGCGGTCACCCGTCATCATAGAGAAGGGGGGCAAGGTGGATGTTGGTGGTCATTTAATCGACCTAAAAAGAGTTGTGCTTGAAGGTTCCGCGGCAACCCCTGTAACCAAGATTTCAGCGGAACAATGGGGTCGTCCATAG

>CF-21peihuai3dongliuchanzhu20251012

ATGTTGGGGAGATGCTTGACCGCGGGCTGTTGCTCGCAATTGCCTTTTTTGTGGTATATCGTGCCGTTCTGTTTTGTTGCGCTCGTCAACGCCAACAACAGCAGCAGCTCCCATTTACAGTTGATTTATAACCTGACGATATGTGAGCTGAATGGCACAGATTGGCTAAATAAAAGTTTTGATTGGGCAGTGGAGACCTTTGTTATTTTCCCTGTGTTGACTCATATTGTCTCCTATGGCGCCCTAACCACCAGCCATTTCCTTGACACAGTTGGCCTGATCACCGTGTCTACCGCTGGGTATTACCACGGGCGGTATGTCCTGAGTAGCATTTACGCCGTCTGCGCCCTAGCTGCGTTAACTTGCTTTGTCATCAGGCTAACAAAAAATTGCATGTCCTGGCGTTACTCATGCACCAGATACACTAACTTTCTTCTGGACACCAAGGGCAAACTCTATCGTTGGCGGTCTCCCGTCATCGTGGAGAAAGGGGGTAAAGTTGAGGTCGCAGGCCACCTGATCGACCTCAAGAGAGTTGTGCTTGACGGTTCCGCGGCAACCCCTATAACCAAAGTTTCAGCGGAACAATGGGGTCGTCCTTAG

>CF-19erqufenmianduanweiye20251016

ATGTTGGGGAGATGCTTGACCGCGGGCTGTTGCTCGCAATTGCCTTTTTTGTGGTATATCGTGCCGTTCTGTTTTGTTGCGCTCGTCAGCGCCAACAACGGCAGCAGCTCCCATTTACAGTTGATTTATAACCTAACGATATGTGAGCTGAATGGCACAGACTGGCTGAATGAACATTTTTCTTGGGCAGTGGAGACTTTCGTTATTTTTCCTGTGTTAACTCACATTGTCTCTTACGGTGCCCTCACCACTAGCCATTTTCTTGACACGGTCGGCCTAATCACTGTGTCCACCGCCGGGTATTATCACGGGCGGTATGTACTGAGTAGCATATACGCTGTCTGCGCCCTAGCTGCGCTTGTTTGCTTCGCCATTAGGTTGGTAAAAAATTGCATGTCCTGGCGCTACTCTTGCACCAGATATACTAACTTCCTTCTGGACACTAAGGGAAAACTCTATCGTTGGCGGTCACCCGTCATCATAGAGAAAGGGGGCAAGGTGGATGTTGGGGGTCATTTAATTGACCTGAAGAGAGTTGTGCTTGAAGGTTCCGCGGCGACTCCTGTAACCAAGATTTCAGCGGAACAATGGGGTCGTCCATAG

>CF-23peihuai7dongzuzhi20251025

TGTTTTAGCCTGTCTTTTTGCCATTCTGCTGGCAATTTGAATGTTCAGGTATGTTGGGGAAATGCTTGACCGCGGGCTGCTGCTCGCAATTGCCTTTTTTGTGGTGTATCGTGCCGTTCTGTTTAGTTGCGCTCGTCAACGCCAACAACGACAGCAGCTCCCACTTACAATTGATTTATAACCTAACGATATGTGAGCTGAATGGCACAGATTGGCTGAAAGAACACTTTTCTTGGGCGGTGGAGACTTTCGTTATTTTTCCTGTGTTGACTCATATCGTCTCCTATGGCGCCCTAACCACCAGCCACTTCCTTGACACAGTCGGCCTGATCACCGTGTCTACTGCTGGATATTACCACGGGCGGTATGTCTTGAGCAGTATTTACGCCGTCTGCGCCTTAGCTGCGCTAATTTGCTTCGTCATCAGGCTAACAAAAAATTGCATGTCCTGGCGCTACTCTTGCACCAGATATACTAATTTTCTTCTGGACACTAAGGGAAAACTCTATCGCTGGCGGTCACCCGTCATCATAGAGAAGGAGGGCAAGGTGGACGTTGGGGGTCATTTAATCGACTTAAAGAGAGTTGTGCTTGAAGGTTCCGCGGCAACCCCTGTAACCAAGATTTCAGCGGAACAATGGGGCCGTCCATAGACGACTTCTGCAATGACAGCACGGCTGTACAAAAGGTGCTTTTGGCGTTTTCA

>CF-23peihuai3dongzuzhi20251028

TGTTTTAGGCCTGTCTTTTTGCCATTCTGCTGGCAATTTGAATGTTCAGGTATGTTGGGGAAATGCTTGACCGCGGGCTGCTGCTCGCAATTGCCTTTTTTGTGGTGTATCGTGCCGTTCTGTTTAGTTGCGCTCGTCAACGCCAACGACAACAGCAGCTCCCACTTACAATTGATTTATAACCTAACGATATGTGAGCTGAATGGCACAGATTGGCTGAAAGACCACTTTTCTTGGGCGGTGGAGACTTTCGTTATTTTTCCTGTGTTAACTCATATTGTCTCTTACGGTGCCCTCACCACTAGCCATTTTCTTGACACGGTCGGTCTTATCACTGTGTCCACCGCCGGGTATTATCACGGGCGGTATGTATTGAGTAGCATTTACGCTGTCTGCGCCTTAGCTGCGCTTGTTTGCTTCGCCATTAGGTTGGTAAAGAATTGCATGTCCTGGCGCTACTCTTGCACCAGATATACCAATTTTCTTCTGGACACTAAGGGAAAACTCTATCGTTGGCGGTCATCCGTCATCATAGAGAAGGGGGGCAAGGTGGATGTTGGTGGTCATTTAATCGACCTAAAGAGAGTTGTGCTTGAAGGTTCCGCGGCAACCCCTGTAACCAAGATTTCAGCGGAACAATGGGGCCGTCCATAGACGACTTCTGCAATGACAGCACGGCTGTACAAAAGGTGCTTTGGCC

>CF-11peihuai4dongzuzhi20251024

CCCCGCTTTTTGCCATTCTGCTGGCAATTTGAATGTTCAGGTATGTCGGGGAAATGCTTGACCGCGGGCTGTTGCTCGCAATTGCCTTTTTTGTGGTATATCGTGCCGTTCTGTTTTGTTGTGCTCGTCAACGCCAACAACAGCAACAGCTCCCATTTACAGTTGATTTATAACCTGACGATATGTGAGCTGAATGGCACAGATTGGCTAGATAAAAATTTTGATTGGGCAGTGGAGACCTTTGTTATTTTCCCCGTGTTGACTCATATTGTCTCCTATGGCGCCCTAACCACCAGCCATTTCCTTGACACAGTTGGCCTGATCACCGTGTCTACCGCTGGGTATTACCACGGGCGGTATGTCCTGAGCAGCATTTACGCCGTCTGCGCCCTAGCTGCGTTAACTTGCTTTGTCATCAGGCTAACAAAAAATTGCATGTCCTGGCGCTACTCTTGCACCAGATATACTAATTTTCTTCTGGACACTAAGGGAAAACTCTATCGCTGGCGGTCACCCGTCATCATAGAGAAGGGGGGCAAGGTGGATGTTGGGGGTCACTTAATTGACCTAAAGAGAGTTGTGCTTGAAGGTTCCGCGGCGACTCCTGTAACCAAGATTTCAGCGGAACAATGGGGTCGTCCATAGACGACTTCTGCAATGACAGCACGGCTGTACAAAAGGTGCTTGGGG

>CF-15chanfang312taiyixue20251018

TGTTTTAGCCTGTCTTTTTGCCATTCTGCTGGCAGTTTAAATGTTCAGGTATGTTGGGGAAATGCTTGACCGCGGGCTGCTGCTCGCAATTGCTTTTTTTGTGGTGTATCGTGCCGTTCTGTTTAGTTTCGCTCGTCAACGCCAGCAACAACAGCAGCTCCCATTTACAGTTGATTTATAACCTGACGATATGTGAGCTGAATGGCACGGATTGGCTGAATAGCAATTTTGATTGGGCAGTGGAGACCTTCGTTATTTTTCCTGTGTTGACTCATATCGTCTCCTACGGCGCCCTAACCACCAGCCACTTCCTTGACACAGTCGGCCTGATCACCGTGTCTACTGCTGGATATTACCACGGGCGGTATGTCTTGAGCAGTATTTACGCCGTCTGCGCCTTAGCTGCGCTAATTTGCTTCGTCATCAGGCTAACAAAAAATTGCATGTCCTGGCGTTACTCATGCACCAGATACACTAATTTTCTTCTGGACACCAAGGGAAAACTCTATCGTTGGCGGTCTCCCGTCATCGTGGAAAAAGGGGGTAAAGTTGAGGTCGAAGGCCACCTCATCGACCTCAAGAAAGTTGTGCTTGATGGTTCCGCGGCGACCCCTATAACCAAAGTTACAGCGGAACAATGGGGTCGTCCTTAGACGACTTCTGCTATGACAGCACAGCCCCGCAAAAGGTGCTTTTGGCGTTTTCA

>CF-21peihuai3dongweigenxue20251024

TGTTTTAGCCTGTCTTTTTGCCATTCTGTTGGCAATTTGAATGTTTAGATATGTTGGGGAAATGCTTGACCGCGGGTTATTGCTCACAATTGCTTTTTTTGTGGTGTATCGTGCCATTCTGTTCTGCTGTGCTCGTCAACGCCAGCGGCAACAGCAGCTCCCACTTACAGTTGATCTATAACCTGACGATATGTGAGCTGAATGGCACAGACTGGTTGAACCAAAAATTTGACTGGGCAGTAGAGACTTTCGTCATTTTTCCTGTCTTGACCCATATTGTCTCTTACGGCGCCCTTACCACTAGCCATTTTCTTGACACGGTCGGCCTAATCACCGTATCCACCGCTGGGTATTATCATAAGCGGTATGTGTTGAGTAGCATCTACGCTGTCTGTGCCCTGGCCGCGTTGGTTTGCTTCGCCATTAGGTTGGCAAAAAATTGCATGTCCTGGCGCTACTCATTCACCAGATATACTAATTTTCTTCTGGACTCTAAGGGCAAACTCTACCGTTGGCGGTCACCCGTCATCATAGAGAAAGAGGGCAAAGTTGATGTGGGGGGTCAGTTAATCGACCTAAAGAGAGTTGTGCTTGAAGGTTCCGCGGCAACCCCTGTAACCAAGATTTCAGCGGAACAATGGGGTCGTCCATAGACGACTTCTGCAATGACAGCACGGCTGTACAAAAGGTGCTTTTGGCGTTTTCA

>CF-22baoyu12jianxueqing20251020

-TGTTTTAGGCCTGTCTTTTTGCCATTCTGCTAGCAATTTGAATGTTCAGGTATGTTGGGGAAATGCTTGACCGCGGGCTGCTGCTCGCAATTGCTTTTTTTGTGGTGTATCGTGCCGTTCTGTTTAGTTGCGTTCGTCAACGCCAACAACAGCAGCAGCTCCCACTTACAGTTGATTTATAACCTAACGATATGTGAGCTGAATGGCACAGATTGGCTGAATGAACATTTTTCTTGGGCAGTGGAGACTTTCGTTATTTTTCCTGTGTTAACTCACATTGTCTCTTACGGTGCCCTCACCACTAGCCATTTTCTTGACATGGTCGGCCTAATCACTGTGTCCACCGCCGGGTATTGTCACGGGCGGTATGTACTGAGTAGCATCTACGCTGTCTGCGCCCTAGCTGCGCTTGTTTGCTTCGCCATTAGGTTGGTAAAAAATTGCATGTCCTGGCGCTACTCTTGCACCAGATATACTAATTTCCTTCTGGACACTAAGGGAAAACTCTATCGTTGGCGGTCACCCGTCATCATAGAGAAAGGGGGCAAGGTGGATGTTGGGGGTCATTTAATTGACCTGAAGAGAGTTGTGCTTGAAGGTTCCGCGGCGACTCCTGTAACCAAGATTTCAGCGGAACAATGGGGTCGTCCATAGACGACTTCTGCAATGACAGCACGGCTGTACAAAAGGTGCTTTTGGCGTTTTCA

>CF-24baoyu1jianxueqing20251020

CCCTTTTTGCCATTCTGCTAGCAATTTGAATGTTCAGGTATGTTGGGGAAATGCTTGACCGCGGGCTGCTGCTCGCAATTACTTTTTTTGTGGTGTATCGTGCCGTTCTGTTTAGTTGCGTTCGTCAACGCCAACAACAGCAGCAGCTCCCACTTACAGTTGATTTATAACCTAACGATATGTGAGCTGAATGGCACAGACTGGCTGAAAGAACATTTTTCTTGGGCAGTGGAGACTTTCGTTATTTTTCCTGTGTTAACCCACATTGTCTCTTACGGTGCCCTCACCACTAGCCATTTTCTTGACACGGTCGGCCTAATCACTGTGTCCACCGCCGGGTATTATCACGGGCGGTATGTACTGAGTAGCATCTATGCTGTCTGCGCCCTAGCTGCGCTTGTTTGCTTCGCCATTAGGTTGGTAAAAAATTGCATGTCCTGGCGCTACTCTTGCACCAGATATACTAATTTTCTTCTGGACACTAAGGGAAAACTCTATCGTTGGCGGTCACCCGTCATCATAGAGAAAGGGGGCAAGGTGGATGTTGGGGGTCATTTAATTGACCTGAAGAGAGTTGTGCTTGAAGGTTCCGCGGCGACTCCTGTAACCAAGATTTCAGCGGAACAATGGGGACATCCATAGACGACTTCTGCAATGACAGCACGGCTGTGCAAAAGGTGCTGGG

>CF2-baoyuxueqing20251101

TGTTTTAGGCCCGTCTTTTTGCCATTCTGTTGGCAATTTGAATGTTTAAGTATGTTGGAGAAATGCTTGACCGCGGGCTGTTGCTCGCAATTGCTTTCTTTGTGGTGTATCGTGCCGTTCTGTTTTGCTGTGCTCGCCAACGCCAGCAACGACAGCAGCTCCCATCTACAGCTGATTTACAACTTGACGCTATGTGAGCTGAATGGCACAGATTGGCTAGCTAACAAATTTGATTGGGCAGTGGAGAGTTTTGTCATCTTTCCCGTTTTGACTCACATTGTCTCCTATGGTGCCCTCACTACCAGCCATTTCCTTGACACAGTCGCTTTAGTCACTGTGTCTACCGCCGGGTTTGTTCACGGGCGGTATGTCCTAAGTAGCATCTACGCGGTCTGTGCCCTGGCTGCGTTGACTTGCTTCGTCATTAGGTTTGCAAAGAATTGCATGTCCTGGCGCTACGCGTGTACCAGATATACCAACTTTCTTCTGGACACTAAGGGCATACTCTATCGTTGGCGGTCGCCTGTCATCATAGAGAAAAGGGGCAAAGTTGAGGTCGAAGGTCATCTGATCGACCTCAAAAGAGTTGTGCTTGATGGTTCCGTGGCAACCCCTATAACCAGAGTTTCAGCGGAACAATGGGGTCGTCCTTAGATGACTTCTGTCGTGATAGCACGGCTCCACAAAAGGTGCTTTG

>CF4-baoyuzuzhi20251101

CCCCGCTTTTTGCCATTCTGCTGGCAATTTGAATGTTCAGGTATGTTGGGGAAATGCTTGACCGCGGGCTGCTGCTCGCAATTGCCTTTTTTGTGGTGTATCGTGCCGTTCTGTTTAGTTGCGCTCGTCAACGCCAACGACAACAGCAGCTCCCACTTACAATTGATTTATAACCTAACGATATGTGAGCTGAATGGCACAGATTGGCTGAAAGACCACTTTCCTTGGGCGGTGGAGACTTTCGTTATTTTTCCTGTGTTAACTCATATTGTCTCTTACGGTGCCCTCACCACTAGCCATTTTCTTGACACGGTCGGTCTTATCACTGTGTCCACCGCCGGGTATTATCACAGGCGGTATGTATTGAGTAGCATCTACGCTGTCTGCGCCCTAGCAGCGCTTGTTTGCTTCGCCATTAGGTTGGTAAAGAATTGCATGTCCTGGCGCTACTCTTGCACCAGATATACTAATTTTCTTCTGGACACTAAGGGAAAACTCTATCGTTGGCGGTCACCCGTCATCATAGAGAAGGGGGGCAAGGTGGATGTTGGTGGTCATTTAATCGACCTAAAGAGAGTTGTGCTTGAAGGTTCCGCGGCAACCCCTGTAACCAAGATTTCAGCGGAACAATGGGGTCGTCCATAGACAACTTCTGCAATGACAGCACGGCTGTACAAAAGGTGTTG

>CF18-erqubaoyuxueqing20251101

CCCGTCTTTTTGCCATTCTGTTGGCAATTTGAATGTTTAGATATGTTGGGGAAATGCTTGACCGCGGGTTACTGCTCATCATTGCTTTTTTTGTGGTGTATCGTGCTATCCTGTTCTGTTGCGCTCGTCAACGCCAACAGCAACACCAGCTCTCATTTACAGTTGATTTATAACCTGACGATATGTGAGCTGAATGGCACGGATTGGTTGAATAAAAGATTTGACTGGGCAGTGGAGACTTTCGTCATTTTTCCTGTTTTGACCCATATTGTCTCGTACGGTGCCCTCACCACTAGCCATTTTCTTGACACGGTCGGCCTAATCACTGTGTCCACCGCTGGGTATTACCATAAGCGGTATGTATTGAGTAGCATCTATGCTGTCTGTGCCCTAGCCGCGTTGGCTTGCTTCGCCATCAGGTTGGCGAAAAATTGCATGTCCTGGCGCTACTCATGCACCAGATACACCAACTTTCTCCTTGACACTAAAGGCAAACTCTACCGCTGGCGGTCACCCGTCATCATAGAGAAGGGGGGCAAAGTTGATGTGGGGGGTCATTTAATTGACCTCAAGAGAGTCGTGCTTGATGGTTCCGCGGCGACCCCTATAACCAAGATTTCAGCGGAACAATGGGGTCGTCCATAGACGACTTCTGCAATGACAGCACGGCCGTACAAAAGGTGCTTTGGGCGGTTTTTCA

>CF20-erqubaoyuxueqing20251102

TGTTTTAGGCCCGTCTTTTTGCCATTCTGCTGGCAATTTGAATGTTCAGGTATGTTGGGGAGATGCTTGACCGCGGGCTGTTGCTCGCAATTGCCTTTTTTGTGGTATATCGTGCCGTTCTGTTTTGTTGCGCTCGTCAACGCCAACAACAGCAGCAGCTCCCATTTACAGTTGATTTATAACCTGACGATATGTGAGCTGAATGGCACAGATTGGCTAAATAAAAGTTTTGATTGGGCAGTGGAGACCTTTGTTATTTTCCCTGTGTTGACTCATATTGTCTCCTATGGCGCCCTAACCACCAGCCATTTCCTTGACACAGTTGGCCTGATCACCGTGTCTACCGCTGGGTATTACCACGGGCGGTATGTCCTGAGTAGCATTTACGCCGTCTGCGCCCTAGCTGCGTTAACTTGCTTTGTCATCAGGCTAACAAAAAATTGCATGTCCTGGCGTTACTCATGCACCAGATACACTAACTTTCTTCTGGACACCAAGGGCAAACTCTATCGTTGGCGGTCTCCCGTCATCGTGGAGAAAGGGGGTAAAGTTGAGGTCGCAGGCCACCTGATCGACCTCAAGAGAGTTGTGCTTGACGGTTCCGCGGCAACCCCTATAACCAAAGTTTCAGCGGAACAATGGGGTCGTCCTTAGACGACTTCTGCTATGACAGCACAGCCCCGCTAAAGGTGCTTGGGG

>CF9-peihuaitaiyizuzhi20251101

TGTTTTAGCCCTGTCTTTTTGCCATTCTGCTGGCAATTTGAATGTTCAGGTATGTTGGGGAAATGCTTGACCGCGGGCTGCTGCTCGCAATTGCCTTTTTTGTGGTTTATCGTGCCGTTCTGTTTAGTTGCGCTCGTCAACGCCAACGACAACAGCAGCTCCCACTTACAATTGATTTATAACCTAACGATATGTGAGCTGAATGGCACAGATTGGCTGAAAGACCACTTTCCTTGGGCGGTGGAGACTTTCGTTATTTTTCCTGTGTTAACTCATATTGTCTCTTACGGTGCCCTCACCACTAGCCATTTTCTTGACACGGTCGGTCTTATCACTGTGTCCACCGCCGGGTATTATCACGGGCGGTATGTATTGAGTAGCATCTACGCTGTCTGCGCCCTAGCTGCGCTTGTTTGCTTCGCCATTAGGTTGGTAAAGAATTGCATGTCCTGGCGCTACTCTTGCACCAGATATACTAATTTTCTTCTGGACACTAAGGGAAAACTCTATCGTTGGCGGTCACCCGTCATCATAGAGAAGGGGGGCAAGGTGGATGTTGGCGGTCATTTAATCGACCTAAAGAGAGTTGTGCTTGAAGGTTCCGCGGCAACCCCTGTAACCAAGATTTCAGCGGAACAATGGGGTCGTCCATAGACAACTTCTGCAATGACAGCACGGCTGTACAAAAGGTGCTTTGGGCC

>CF17-erqupeihuaitaiyixu20251106

CCCG-CTTTTTGCCATTCTGCTGGCAATTTGAATGTTCAGGTATGTTGGGGAGATGCTTGATCGCGGGCTGTTGCTCGCAATTGCCTTTTTTGTGGTATATCGTGCCGTTCTGTTTTGTTGCGCTCGTCAACGCCAACAACAGCAGCAGCTCCCATTTACAGTTGATTTATAACCTGACGATATGTGAGCTGAATGGCACAGATTGGCTAAATAAAAGTTTTGATTGGGCAGTGGAGACCTTTGTTATTTTCCCTGTGTTGACTCATATTGTCTCCTATGGCGCCCTAACCACCAGCCATTTCCTTGACACAGTTGGCCTGATCACCGTGTCTACCGCTGGGTATTACCACGGGCGGTATGTCCTGAGTAGCATTTACGCCGTCTGCGCCCTAGCTGCGTTAACTTGCTTTGTCATCAGGCTAACAAAAAATTGCATGTCCTGGCGTTACTCATGCACCAGATACACTAACTTTCTTCTGGACACCAAGGGCAAACTCTATCGTTGGCGGTCTCCCGTCATCGTTGAGAAAGGGGGTAAAGTTGAGGTCGCAGGCCACCTGATCGACCTCAAGAGAGTTGTGCTTGACGGTTCCGCGGCAACCCCTATAACCAAAGTTTCAGCGGAACAATGGGGTCGTCCTTAGACGACTTCTGCTATGACAGCACAGCCCCGCTAAAGGTGCTTGGG

>CF6-baoyuxueqing20251110

TGTTTTAGCCCGTCTTTTTGCCATTCTGCTGGCAATTTGAATGTTCAGGTATGTCGGGGAAATGCTTGACCGCGGGCTGTTGCTCGCAATTGCCTTTTTTGTGGTATATCGTGCCGTTCTGTTTTGTTGTGCTCGTCAACGCCAACAACAGCAGCAGCTCCCATTTACAGTTGATTTATAACCTGACGATATGTGAGCTGAATGGCACAGATTGGCTAAATAAGAGTTTTGATTGGGCAGTGGAGACCTTTGTTATCTTCCCTGTGTTGACTCATATTGTCTCCTATGGCGCCCTAACCACCAGCCATTTCCTTGACACAGTTGGCCTGATCACCGTGTCTACCGCTGGGTATTGCCACGGGCGGTATGTCCTGAGCAGCATTTACGCCGTCTGCGCCCTAGCTGCGTTAACTTGCTTTGTCATCAGGCTAACAAAAAATTGCATGTCCTGGCGTTACTCATGCACCAGATACACTAACTTTCTTCTGGACACCAAGGGCAGACTCTATCGTTGGCGGTCTCCCGTCATCGTGGAGAAAGGGGGTAAAGTTGAGGTCGCAGGCCACCTGATCGACCTCAAGAGAGTTGTGCTTGACGGTTCCGCGGCAACCCCTATAACCAAAGTTTCAGCGGAGCAATGGGGTCGTCCTTAGACGACTTCTGCTATGACAGCAAAGCCCCGCAAAAGGTGCTTTTGGGC

>CF9-peihuaitaiyixue20251108

TGTTTTAGCC-TGTCTTTTTGCCATTCTGCTGGCAATTTGAATGTTCAGGTATGTTGGGGAAATGCTTGACCGCGGGCTGCTGCTCGCAATTGCCTTTTTTGTGGTTTATCGTGCCGTTCTGTTTAGTTGCGCTCGTCAACGCCAACGACAACAGCAGCTCCCACTTACAATTGATTTATAACCTAACGATATGTGAGCTGAATGGCACAGATTGGCTGAAAGACCACTTTCCTTGGGCGGTGGAGACTTTCGTTATTTTTCCTGTGTTAACTCATATTGTCTCTTACGGTGCCCTCACCACTAGCCATTTTCTTGACACGGTCGGTCTTATCACTGTGTCCACCGCCGGGTATTATCACGGGCGGTATGTATTGAGTAGCATCTACGCTGTCTGCGCCCTAGCTGCGCTTGTTTGCTTCGCCATTAGGTTGGTAAAGAATTGCATGTCCTGGCGCTACTCTTGCACCAGATATACTAATTTTCTTCTGGACACTAAGGGAAAACTCTATCGTTGGCGGTCACCCGTCATCATAGAGAAGGGGGGCAAGGTGGATGTTGGCGGTCATTTAATCGACCTAAAGAGAGTTGTGCTTGAAGGTTCCGCGGCAACCCCTGTAACCAAGATTTCAGCGGAACAATGGGGTCGTCCATAGACAACTTCTGCAATGACAGCACGGCTGTACAAAAGGTGCTTTTGGGCGTTTTC

>CF10-baoyuxueqing20251110

CCCCGCTTTTTGCCATTCTGCTGGCAATTTGAATGTTCAGGTATGTTGGGGAAATGCTTGACCGCGGGCTGCTGCTCGCAATTGCTTTTTTTGTGGTGTATCGTGCCGTTCTGTTTAGTTGCGCTCGTCAACGCCAACGAAAACAGCAGCTCCCACTTACAATTGATTTATAACCTAACGATATGTGAGCTGAATGGCACAGATTGGCTGAATGAACACTTTTCTTGGGCGGTGGAGACTTTCGTTATTTTTCCTGTGTTAACTCATATTGTCTCTTACGGTGCCCTCACCACTAGCCATTTTCTTGACACGGTCGGTCTTATCACTGTGTCCACCGCCGGGTATTATCACGGGCGGTATGTATTGAGTAGCATCTACGCTGTCTGCGCCTTAGCTGCGCTTGGTTGCTTCACCATTAGGTTAGTAAAAAATTGCATGTCCTGGCGCTACTCTTGCACCAGATATACTAATTTTCTTCTGGACACTAAGGGAAAACTCTATCGTTGGCGGTCACCCGTCATCATAGAGAAGGGGGGCAAGGTGGATGTTGGTGGGCATTTAATCGACCTAAAGAGAGTTGTGCTTGAAGGTTCCGCGGCAACCCCTGTAACCAAGATTTCAGCGGAACAATGGGGTCGTCCATAGATGACTTCTGCAATGACAGCACGGCTGTACAAAAGGTGCTTGGGG

>CF25-GDUzuzhi20251103

TTGGCAATTTGAATGTTCAAGTATGTTGGGGAAATGCTTGACCGCGGGCTGCTGCTCGCAATTACTTTTTTTGTGGTGTATCGTGCCGTTCTGTTTAGTTGCGTTCGTCAACGCCAACAACAGCAGCAGCTCCCACTTACAGTTGATTTATAACCTAACGATATGTGAGCTGAATGGCACAGACTGGCTGAAAGAACATTTTTCTTGGGCAGTGGAGACTTTCGTTATTTTTCCTGTGTTAACCCACATTGTCTCTTACGGTGCCCTCACCACTAGCCATTTTCTTGACACGGTCGGCCTAATCACTGTGTCCACCGCCGGGTATTATCACGGGCGGTATGTACTGAGTAGCATCTATGCTGTCTGCGCCCTAGCTGCGCTTGTTTGCTTCGCCATTAGGTTGGTAAAAAATTGCATGTCCTGGCGCTACTCTTGCACCAGATATACTAATTTTCTTCTGGACACTAAGGGAAAACTCTATCGTTGGCGGTCACCCGTCATCATAGAGAAAGGGGGCAAGGTGGATGTTGGGGGTCATTTAATTGACCTAAAGAGAGTTGTGCTTGAAGGTTCCGCGGCGACTCCTGTAACCAAGATTTCAGCGGAACAATGGGGACATCCATAGACGACTTCTGCAATGATAGCACAG

>CF19-pwihuai6jiantaiyi20251114

TAGCCCTGTCTTTTTGCCATTCTGTTGGCAATTTGAATGTTTAGATATGTTGGGGAAATGCTTGACCGCGGGTTACTGCTCATCATTGCTTTTTTTGTGGTGTATCGTGCTATCCTGTTCTGTTGCGCTCGTCAACGCCAACAGCAACACCAGCTCTCATTTACAGTTGATTTATAACCTGACGATATGTGAGCTGAATGGCACGGATTGGTTGGATAAAAGATTTGACTGGGCAGTAGAGACTTTCGTCATTTTTCCTGTTCTGACCCATATTGTCTCGTACGGTGCCCTCACCACTAGCCATTTTCTTGACACGGTCGGCCTAATCACTGTGTCCACCGCTGGGTATTACCATAAGCGGTATGTATTGAGTAGCATCTATGCTGTCTGTGCCCTGGCCGCGTTGGCTTGCTTCGCCATCAGGTTGGCGAAAAATTGCATGTCCTGGCGCTACTCATGCACCAGATACACCAACTTCCTCCTTGACACTAAAGGCAAACTCTACCGCTGGCGGTCACCCGTCATCATAGAGAAGGGGGGCAAAGTTGATGTGGGGGGTCATTTAATTGACCTCAAGAGAGTCGTGCTTGATGGTTCCGCGGCGACCCCTATAACCAAGATTTCAGCGGAACAATGGGGTCGTCCATAGACGACTTCTGCAATGACAGCACGGCCGTACAAAAGGTGCTTTTGGGCG

>CF25-GDUzuzhi20251118

CCCCGCTTTTTGCCATTCTGCTGGCAATTTAAATGTTCAGGTATGTTGGGGAAATGCTTGACCGCGGGCTGCTGCTCGCAATTGCTTTTTTTGTGGTGTATCGTGCCGTTCTGTTTAGCTTCGCTCGTCAGCGCCAGCAACAGCAACAGCTCCCATTTACAGTTGATTTATAACCTGACGATATGTGAGCTGAATGGCACAGATTGGCTAAATCGCACTTTTGATTGGGCAGTGGAGACTTTCGTTATTTTTCCTGTGTTGACCCATATCGTCTCCTATGGCGCCCTGACCACCAGCCACTTCCTTGACACAGTCGGCCTGATCACCGTGTCTACTGCTGGATATTGCCACGGGCGGTATGTCTTGAGCAGTATTTACGCCGTCTGCGCCTTAGCTGCGCTAATTTGCTTCGTCATCAGGCTAACAAAAAATTGCATGTCCTGGCGTTACTCATGCACCAGATACACTAATTTTCTTCTGGACACCAAGGGAAAACTCTATCGTTGGCGATCTCCCGTCATCGTGGAAAAAGGGGGCAAAGTTGAGGTCGAAGGCCACCTCATCGACCTCAAGAAAGTTGTGCTTGATGGTTCCGCGGCGACCCCTATAACCAAAGTTTCAGCGGAACAATGGGGTCGTCCTTAGACGACTTCTGCTATGACAGCACAGCCCCGCAAAAGGTGCTGGG

>CF3-chanfangyanshizi20251118

-TGTTTTAAGCCCGTCTTTTTGCCATTCTGCTGGCAATTTGAATGTTCAGGTATGTTGGGGAAATGCTTGACCGCGGGCTGCTGCTCGCAATTGCCTTTTTTATGGTGTATCGTGCCGTTCTGTTTGGTTGCGCTCGTCAACGCCACCGACAACAGCAGCTCCCACTTACAATTGATTTATAACCTAACGATATGTGAGCTGAATGGCACAGATTGGCTGAAAGAACACTTTTCTTGGGCGGTGGAGACTTTCGTTATTTTTCCTGTGTTAACTCATGTTGTCTCTTACGGTGCCCTCACCACTAGCCATTTTCTTGACACGGTCGGTCTTATCACTGTGTCCACCGCCGGGTATTATCACGGGCGGTATGTATTGAGTAGCATCTATGCTGTCTGCGCCCTAGCTGCGCTTGTTTGCTTCGCCATTAGGTTGGCAAAGAATTGCATGTCCTGGCGCTACTCTTGCACCAGATATACTAATTTTCTTCTGGACACTAAGGGAAAACTCTATCGTTGGCGGTCACCCGTCATCATAGAGAAGGGGGGCAAGGTGGATGTCGGGGGTCATTTAATCGACCTAAAGAGAGTTGTGCTTGAAGGTTCCGCGGCAACCCCTGTAACCAAGATTTCAGCGGAACAATGGGGTCGTCCATAGACGACTTCTGCAATGACAGCACGGCTGCACAAAAGGTGCTTT-GGGCGTTTTTCA

>CF26-baoyuxueqing20251122

CCCGCTTTTTGCCATTCTGTTGGCAATTTGAATGTTTAGATATGTTGGGGAAATGCTTGACCGCGGGTTGCTGCTCATCATTGCTTTTTTTGTGGTGTATCGTGCTATCTTGTTCTGTTGCGCTCGTCAACGCCGACAGCAACACCAGCTCTCATTTACAGTTGATTTATAACCTGACGATATGTGAGCTGAATGGCACGGATTGGTTGGACAAAAAATTTGACTGGGCAGTAGAGACTTTCGTCATTTTTCCTGTTCTAACCCATATTGTCTCTTACGGCGCCCTCACCACTAGCCATTTTCTTGACATGGTCGGCCTAATTACTGTGTCCACCGCTGGGTATTACCATAAGCGGTATGTATTGAGTAGCATCTATGCTGTCTGTGCCCTGGCCGCGTTGGCTTGCTTCGCCATCAGGTTGGCGAAAAATTGCATGTCCTGGCGCTACTCATGCACCAGATACACCAACTTTCTCCTGGACACTAAAGGCAGACTCTACCGCTGGCGGTCACCCGTCATCATTGAGAAGGGGGGCAAAGTTGATGTGGGGGGTCATTTAATTGACCTCAAGAGAGTCGTGCTTGATGGTTCCGCGGCGACCCCTATAACCAAGATTTCAGCGGAACAATGGGGTCGTCCATAGACGACTTCTGCAATGACAGCACGGCTGTACAAAAGGTGCTTGGG

>CF13-fenmianxueqing20251122

TTTTTTTAGCCCCTTCTTTTTGCCATTCTGCTGGCAATTTGAATGTTCAGGTATGTTGGGGAAATGCTTGACCGCGGGCTGCTGCTCGCAATTGCCTTTTTTGTGGTGTATCGTGCCGTTCTGTTTAGTTGCGCTCGCCAACGCCAACAACGACAGCAGCTCCCACTTACAATTGATTTATAACCTAACGATATGTGAGCTGAATGGCACAGATTGGCTGAACAAACACTTTCCTTGGGCGGTGGAGACTTTCGTTATTTTTCCTGTGTTAACGCATGTTGTCTCTTACGGTGCCCTCACCACTAGCCATTTTCTTGACACGGTCGGTCTTATCACTGTGTCCACCGCCGGGTATTATCACGGGCGGTATGTATTGAGTAGCATCTACGCTGTCTGCGCCCTAGCTGCGCTTGTTTGCTTCGCCATTAGGTTGGTAAAGAATTGCATGTCCTGGCGCTACTCTTGCACCAGATATACTAATTTTCTTCTGGACACAAAGGGAAAACTCTATCGTTGGCGGTCACCCGTCATCATAGAGAAGGGGGGCAAGGTGGATGTTGGGGGTCATTTAATCGACCTAAAGAGAGTTGTGCTTGAAGGTTCCGCGGCAACCCCTGTAACCAAGATTTCAGCGGAACAATGGGGTCGTCCATAGACGACTTCTGCAATGACAGCACGGCTGTACAAAAGGTGCTTGGGG

>CF13-peihuaizuzhi20251124

CCCCCGCTTTTTGCCATTCTGCTGGCAATTTGAATGTTCAGGTATGTTGGGGAAATGCTTGACCGCGGGCTGCTGCTCGCAATTGCCTTTTTTGTGGTGTATCGTGCCGTTCTGTTTAGTTGCGCTCGCCAACGCCAACAACGACAGCAGCTCCCACTTACAATTGATTTATAACCTAACGATATGTGAGCTGAATGGCACAGATTGGCTGAACAAACACTTTCCTTGGGCGGTGGAGACTTTCGTTATTTTTCCTGTGTTAACGCATGTTGTCTCTTACGGTGCCCTCACCACTAGCCATTTTCTTGACACGGTCGGTCTTATCACTGTGTCCACCGCCGGGTATTATCACGGGCGGTATGTATTGAGTAGCATCTACGCTGTCTGCGCCCTAGCTGCGCTTGTTTGCTTCGCCATTAGGTTGGTAAAGAATTGCATGTCCTGGCGCTACTCTTGCACCAGATATACTAATTTTCTTCTGGACACAAAGGGAAAACTCTATCGTTGGCGGTCACCCGTCATCATAGAGAAGGGGGGCAAGGTGGATGTTGGGGGTCATTTAATCGACCTAAAGAAAGTTGTGCTTGAAGGTTCCGCGGCAACCCCTGTAACCAAGATTTCAGCGGAACAATGGGGTCGTCCATAGACGACTTCTGCAATGACAGCACGGCTGTACAAAAGGTGCTTTGGGG

>CF-7-yanshizi20251123-5

CCCG-CTTTTTGCCATTCTGCTGGCAATTTGAATGTTCAGGTATGTTGGGGAAATGCTTGACCGCGGGCTGCTGCTCGCAATTGCCTTTTTTGTGGTGTATCGTGCCGTTCTGTTTGGTTGCGCTCGTCAACGCCAACGACAACAGCAGCTCCCACTTACAATTGATTTATAACCTAACGATATGTGAGCTGAATGGCACAGATTGGCTGAAAGAACACTTTTCTTGGGCGGTGGAGACTTTCGTTATTTTTCCTGTGTTAACTCATGTTGTCTCTTACGGTGCCCTCACCACTAGCCATTTTCTTGACACGGTCGGTCTTATCACTGTGTCCACCGCCGGGTATTATCACGGGCGGTATGTATTGAGTAGCATCTACGCTGTCTGCGCCCTAGCTGCGCTTGTTTGCTTCGCCATTAGGTTGGCAAAGAATTGCATGTCCTGGCGCTACTCTTGCACCAGATATACTAATTTTCTTCTGGACACTAAGGGAAAACTCTATCGTTGGCGGTCACCCGTCATCATAGAGAAAGGGGGCAAGGTGGATGTCGGGGGTCATTTAATCGACCTAAAGAGAGTTGTGCTTGAAGGTTCCGCGGCAACTCCTGTAACCAAGATTTCAGCGGAACAATGGGGTCGTCCATAGACGACTTCTGCAATGACAGCACGGCTGTACAAAAGGTGCTTTGGG

>CF-7-yanshizi20251124-6

TGTTTTAGCCCTGTCTTTTTGCCATTCTGCTGGCAATTTGAATGTTCAGGTATGTTGGGGAAATGCTTGACCGCGGGCTGCTGCTCGCAATTGCCTTTTTTGTGGTGTATCGTGCCGTTCTGTTTGGTTGCGCTCGTCAACGCCAACGACAACAGCAGCTCCCACTTACAATTGATTTATAACCTAACGATATGTGAGCTGAATGGCACAGATTGGCTGAAAGAACACTTTTCTTGGGCGGTGGAGACTTTCGTTATTTTTCCTGTGTTAACTCATGTTGTCTCTTACGGTGCCCTCACCACTAGCCATTTTCTTGACACGGTCGGTCTTATCACTGTGTCCACCGCCGGGTATTATCACGGGCGGTATGTATTGAGTAGCATCTACGCTGTCTGCGCCCTAGCTGCGCTTGTTTGCTTCGCCATTAGGTTGGCAAAGAATTGCATGTCCTGGCGCTACTCTTGCACCAGATATACTAATTTTCTTCTGGACACTAAGGGAAAACTCTATCGTTGGCGGTCACCCGTCATCATAGAGAAAGGGGGCAAGGTGGATGTCGGGGGTCATTTAATCGACCTAAAGAGAGTTGTGCTTGAAGGTTCCGCGGCAACTCCTGTAACCAAGATTTCAGCGGAACAATGGGGTCGTCCATAGACGACTTCTGCAATGACAGCACGGCTGTACAAAAGGTGTTTG

>CF21-peihuaimuzhu20251126-7

TTTTTTTAGGCCTGTCTTTTTGCCATTCTGCTGGCAATTTGAATGTTCAGGTATGTTGGGGAGATGCTTGACCGCGGGCTGTTGCTCGCAATTGCCTTTTTTGTGGTATATCGTGCCGTTCTGTTTTGTTGCGCTCGTCAACGCCAACAACAGCAGCAGCTCCCATTTACAGTTGATTTATAACCTGACGATATGTGAGCTGAATGGCACAGATTGGCTAAATAAAAGTTTTGATTGGGCAGTGGAGACCTTTGTTATTTTCCCTGTGTTGACTCATATTGTCTCCTATGGCGCCCTAACCACCAGCCATTTCCTTGACACAGTTGGCCTGATCACCGTGTCTACCGCTGGGTATTACCACGGGCGGTATGTCCTGAGTAGCATTTACGCCGTCTGCGCCCTAGCTGCGTTAACTTGCTTTGTCATCAGGCTAACAAAAAATTGCATGTCCTGGCGTTACTCATGCACCAGATACACTAACTTTCTTCTGGACACCAAGGGCAAACTCTATCGTTGGCGGTCTCCCGTCATCGTGGAGAAAGGGGGTAAAGTTGAGGTCGCAGGCCACCTGATCGACCTCAAGAGAGTTGTGCTTGACGGTTCCGCGGCAACCCCTATAACCAAAGTTTCAGCGGAACAATGGGGTCGTCCTTAGACGACTTCTGCTATGACAGCACAGCCCCGCTAAAGGTGCTTTTGGGCGTTTTCA

>CF11-peihuaimuzhu20251127-8

TGTTTTAGCCCTGTCTTTTTGCCATTCTGCTGGCAATTTGAATGTTCAGGTATGTTGGGGAAATGCTTGACCGCGGGCTGCTGCTCGCAATTGCCTTTTTTATGGTTTATCGTGCCGTTCTGTTTAGTTGCGCTCGTCAACGCCAACGACAACAGCAGCTCCCACTTACAATTGATTTATAACCTAACGATATGTGAGCTGAATGGCACAGATTGGCTGAAAGACCACTTTTCTTGGGCGGTGGAGACTTTCGTTATTTTTCCTGTGTTAACTCATATTGTCTCTTACGGTGCCCTCACCACTAGCCATTTTCTTGACACGGTCGGCCTTATCACTGTGTCCACCGCCGGGTATTATCACGGGCGGTATGTATTGAGTAGCATCTACGCTGTCTGCGCCCTAGCTGCGCTTGTTTGCTTCGCCATTAGGTTGGTAAAGAATTGCATGTCCTGGCGCTACTCTTGCACCAGATATACTAATTTTCTTCTGGACACTAAGGGAAAACTCTATCGTTGGCGGTCACCCGTCATCATTGAGAAGGGGGGCAAGGTGGATGTTGGTGGTCATTTAATCGACCTAAAGAGAGTTGTGCTTGAAGGTTCCGCGGCAACCCCTGTAACCAAGATTTCAGCGGAACAATGGGGTCGTCCATAGACAACTTCTGCAATGACAGCACGGCTGTACAAAAGGTGCTTGGG

>CF-8-yanshizi20251128

TTGTTTTTAGCCCGTCTTTTTGCCATTCTGCTGGCAGTTTAAATGTTCAGGTATGTTGGGGAAATGCTTGACCGCGGGCTGCTGCTCGCAATTGCTTTTTTTGTGGTGTATCGTGCCGTTCTGTTTAGTTTCGCTCGTCAACGCCAGCAACAGCAGCAGCTCCCATTTACAGTTGATTTATAACCTGACGATATGTGAGCTGAATGGCACAGATTGGCTAAATCGCACTTTTGATTGGGCAGTGGAGACCTTCGTTATTTTTCCTGTGTTGACTCATATCGTCTCCTATGGCGCCCTAACCACCAGCCACTTCCTTGACACAGTCGGCCTGATCACCGTGTCTACTGCTGGATATTACCACGGGCGGTATGTCTTGAGCAGTATTTACGCCGTCTGCGCCTTAGCTGCGCTAATTTGCTTCGTCATCAGGCTAACAAAAAATTGCATGTCCTGGCGTTACTCATGCACCAGATACACTAATTTTCTTCTGGACACCAAGGGAAAACTCTATCGTTGGCGGTCTCCCGTCATCGTGGAAAAAGGGGGTAAAGTTGAGGTCGAAGGCCACCTCATCGACCTCAAGAAAGTTGTGCTTGATGGTTCCGCGGCGACCCCTATAACCAAAGTTACAGCGGAACAATGGGGTCGTCCTTAGACGACTTCTGCTATGACAGCACAGCCCCGCAAAAGGTGCTTTGGGCCGTTTTTC

>CF-8-yanshizi20251128

TGTTTTAGCCCTGTCTTTTTGCCATTCTGCTGGCAGTTTAAATGTTCAGGTATGTTGGGGAAATGCTTGACCGCGGGCTGCTGCTCGCAATTGCTTTTTTTGTGGTGTATCGTGCCGTTCTGTTTAGTTTCGCTCGTCAACGCCAGCAACAGCAGCAGCTCCCATTTACAGTTGATTTATAACCTGACGATATGTGAGCTGAATGGCACAGATTGGCTAAATCGCACTTTTGATTGGGCAGTGGAGACCTTCGTTATTTTTCCTGTGTTGACTCATATCGTCTCCTATGGCGCCCTAACCACCAGCCACTTCCTTGACACAGTCGGCCTGATCACCGTGTCTACTGCTGGATATTACCACGGGCGGTATGTCTTGAGCAGTATTTACGCCGTCTGCGCCTTAGCTGCGCTAATTTGCTTCGTCATCAGGCTAACAAAAAATTGCATGTCCTGGCGTTACTCATGCACCAGATACACTAATTTTCTTCTGGACACCAAGGGAAAACTCTATCGTTGGCGGTCTCCCGTCATCGTGGAAAAAGGGGGTAAAGTTGAGGTCGAAGGCCACCTCATCGACCTCAAGAAAGTTGTGCTTGATGGTTCCGCGGCGACCCCTATAACCAAAGTTACAGCGGAACAATGGGGTCGTCCTTAGACGACTTCTGCTATGACAGCACAGCCCCGCAAAAGGTGCTTTTGGCGTTTTCA

>CF7-houbeiweigenxue20251130

TGTTTTTAGGCCCGTCTTTTTGCCATTCTGCTGGCAATTTGAATGTTCAGGTATGTTGGGGAAATGCTTGACCGCGGGCTGCTGCTCGCAATTGCCTTTTTTGTGGTGTATCGTGCCGTTCTGTTTGGTTGCGCTCGTCAACGCCAACGACAACAGCAGCTCCCACTTACAATTGATTTATAACCTAACGATATGTGAGCTGAATGGCACAGATTGGCTGGAAAAACACTTTTCTTGGGCGGTGGAGACTTTCGTTATTTTTCCTGTGTTAACTCATGTTGTCTCTTACGGTGCCCTCACCACTAGCCATTTTCTTGACACGGTCGGTCTTATCACTGTGTCCACCGCCGGGTATTATCACGGGCGGTATGTATTGAGTAGCATCTACGCTGTCTGCGCCCTAGCTGCGCTTGTTTGCTTCGCCATTAGGTTGGCAAAGAATTGCATGTCCTGGCGCTACTCTTGCACCAGATATACTAATTTTCTTCTGGACACTAAGGGAAAACTCTATCGTTGGCGGTCACCCGTCATCATAGAGAAGGGGGGCAAGGTGGATGTTGGGGGTCATTTAATCGACCTAAAGAGAGTTGTGCTTGAAGGTTCCGCGGCAACCCCTGTAACCAAGATTTCAGCGGAACAATGGGGTCGTCCATAGACGACTTCTGCAATGACAGCACGGCTGTACAAAAGGTGCTTTTGGGCGTTTTCA

>CF9-peihuaitaiyixue20251130

TGTTTTAAGCCCGTCTTTTTGCCATTCTGCTGGCAATTTGAATGTTCAGGTATGTTGGGGAAATGCTTGACCGCGGGCTGCTGCTCGCAATTGCTTTTTTTGTGGTTTATCGTGCCGTTCTGTTTAGTTGCGCTCGTCAACGCCAACGACAACAGCAGCTCCCACTTACAATTGATTTATAAACTAACGATATGTGAGCTGAATGGCACAGATTGGCTGAAAGACCACTTTCCTTGGGCGGTGGAGACTTTCGTTATTTTTCCTGTGTTAACTCATATTGTCTCTTACGGTGCCCTCACCACTAGCCATTTTCTTGACACGGTCGGTCTTATCACTGTGTCCACCGCCGGGTATTATCACGGGCGGTATGTGTTGAGTAGCATCTACGCTGTCTGCGCCCTAGCTGCGCTTGTTTGCTTCGCCATTAGGTTGGTAAAGAATTGCATGTCCTGGCGCTACTCTTGCACCAGATATACTAATTTTCTTCTGGACACTAAGGGAAAACTCTATCGTTGGCGGTCACCCGTCATCATAGAGAAGGGGGGCAAGGTGGATGTTGGTGGTCATTTAATCGACCTAAAGAGAGTTGTGCTTGAAGGTTCCGCGGCAACCCCTGTAACCAAAATTTCAGCGGAACAATGGGGTCGTCCATAGACAACTTCTGCAATGACAGCACGGCTGTACAAAAGGTGCTTTGGG

>CF13-fenmiantaiyixue20251130

TTGTTTTAGCCCTGTCTTTTTGCCATTCTGCTGGCAATTTGAATGTTCAGGTATGTTGGGGAAATGCTTGACCGCGGGCTGCTGCTCGCAATTGCCTTTTTTGTGGTGTATCGTGCCGTTCTGTTTAGTTGCGCTCGCCAACGCCAACAACGACAGCAGCTCCCACTTACAATTGATTTATAACCTAACGATATGTGAGCTGAATGGCACAGATTGGCTGAACAAACACTTTCCTTGGGCGGTGGAGACTTTCGTTATTTTTCCTGTGTTAACGCATGTTGTCTCTTACGGTGCCCTCACCACTAGCCATTTTCTTGACACGGTCGGTCTTATCACTGTGTCCACCGCCGGGTATTATCACGGGCGGTATGTATTGAGTAGCATCTACGCTGTCTGCGCCCTAGCTGCGCTTGTTTGCTTCGCCATTAGGTTGGTAAAGAATTGCATGTCCTGGCGCTACTCTTGCACCAGATATACTAATTTTCTTCTGGACACAAAGGGAAAACTCTATCGTTGGCGGTCACCCGTCATCATAGAGAAGGGGGGCAAGGTGGATGTTGGGGGTCATTTAATCGACCTAAAGAAAGTTGTGCTTGAAGGTTCCGCGGCAACCCCTGTAACCAAGATTTCAGCGGAACAATGGGGTCGTCCATAGACGACTTCTGCAATGACAGCACGGCTGTACAAAAGGTGCTTTGGGCGTTTTTCA

>CF3-fenmianduanweiye20251210

TTGTTTTAGCCCTGTCTTTTTGCCATTCTGCTGGCAATTTGAATGTTCAGGTATGTTGGGGAAATGCTTGACCGCGGGCTGCTGCTCGCAATTGCCTTTTTTATGGTGTATCGTGCCGTTCTGTTTGGTTGCGCTCGTCAACGCCACCGACAACAGCAGCTCCCACTTACAATTGATTTATAACCTAACGATATGTGAGCTGAATGGCACAGATTGGCTGAAAGAACACTTTTCTTGGGCGGTGGAGACTTTCGTTATTTTTCCTGTGTTAACTCATGTTGTCTCTTACGGTGCCCTCACCACTAGCCATTTTCTTGACACGGTCGGTCTTATCACTGTGTCCACCGCCGGGTATTATCACGGGCGGTATGTATTGAGTAGCATCTATGCTGTCTGCGCCCTAGCTGCGCTTGTTTGCTTCGCCATTAGGTTGGCAAAGAATTGCATGTCCTGGCGCTACTCTTGCACCAGATATACTAATTTTCTTCTGGACACTAAGGGAAAACTCTATCGTTGGCGGTCACCCGTCATCATAGAGAAGGGGGGCAAGGTGGATGTCGGGGGTCATTTAATCGACCTAAAGAGAGTTGTGCTTGAAGGTTCCGCGGCAACCCCTGTAACCAAGATTTCAGCGGAACAATGGGGTCGTCCATAGACGACTTCTGCAATGACAGCACGGCTGCACAAAAGGTGCTTTTGGGC

>CF13-fenmianduanweiye20251210

TGTTTTAGCCTGTCTTTTTGCCATTCTGCTGGCAATTTGAATGTTCAGGTATGTTGGGGAAATGCTTGACCGCGGGCTGCTGCTCGCAATTGCCTTTTTTGTGGTGTATCGTGCCGTTCTGTTTAGTTGCGCTCGCCAACGCCAACAACGACAGCAGCTCCCACTTACAATTGATTTATAACCTAACGATATGTGAGCTGAATGGCACAGATTGGCTGAACAAACACTTTCCTTGGGCGGTGGAGACTTTCGTTATTTTTCCTGTGTTAACGCATGTTGTCTCTTACGGTGCCCTCACCACTAGCCATTTTCTTGACACGGTCGGTCTTATCACTGTGTCCACCGCCGGGTATTATCACGGGCGGTATGTATTGAGTAGCATCTACGCTGTCTGCGCCCTAGCTGCGCTTGTTTGCTTCGCCATTAGGTTGGTAAAGAATTGCATGTCCTGGCGCTACTCTTGCACCAGATATACTAATTTTCTTCTGGACACAAAGGGAAAACTCTATCGTTGGCGGTCACCCGTCATCATAGAGAAGGGGGGCAAGGTGGATGTTGGGGGTCATTTAATCGACCTAAAGAGAGTTGTGCTTGAAGGTTCCGCGGCAACCCCTGTAACCAAGATTTCAGCGGAACAATGGGGTCGTCCATAGACGACTCTGCAATACTGACAGGGCCCA

>CF8-yufeiyanshizi20251210

TGTTTTAGCCC-GTCTTTTTGCCATTCTGCTGGCAATTTGAATGTTCAGGTATGTTGGGGAAATGCTTGACCGCGGGCTGCTGCTCGCAATTGCTTTTTTTGTGGTGTATCGTGCCGTTCTGTTTAGTTGCGCTCGTCAACGCCAACGACAACAGCAGCTCCCACTTACAGTTGATTTATAACCTAACAATATGTGAGCTGAATGGCACAGATTGGCTGAAAGAACACTTTTCTTGGGCGGTGGAGACTTTCGTTATTTTCCCTGTGTTAACTCATGTTGTCTCTTACGGTGCCCTCACCACTAGCCATTTTCTTGACACGGTCGGTCTTATCACTGTGTCCACCGCCGGGTATTATCACGGGCGGTATGTACTGAGTAGTATCTACGCTGTCTGCGCCCTAGCTGCGCTTGTTTGCTTCGCCATTAGGTTGGTAAAGAATTGCATGTCCTGGCGCTACTCTTGCACCAGATATACTAATTTTCTTCTGGACACTAAGGGAAAACTCTATCGTTGGCGGTCACCCGTCATCATAGAGAAGGGGGGCAAGGTGGATGTCGGGGGTCATTTAATCGACCTAAAGAGAGTTGTGCTTGAAGGTTCCGCGGCAACCCCTGTAACCAAGATTTCAGCGGAACAATGGGGTTGTCCATAGACGACTTCTGCAATGACAGCACGGCTGTACAAAAGGTGCTTTGGG

>CF8-yufeiyanshizi20251210-21

TGTTTTAGCCCTGTCTTTTTGCCATTCTGCTGGCAATTTGAATGTTCAGGTATGTTGGGGAAATGCTTGACCGCGGGCTGCTGCTCGCAATTGCTTTTTTTGTGGTGTATCGTGCCGTTCTGTTTAGTTGCGCTCGTCAACGCCAACGACAACAGCAGCTCCCACTTACAGTTGATTTATAACCTAACAATATGTGAGCTGAATGGCACAGATTGGCTGAAAGAACACTTTTCTTGGGCGGTGGAGACTTTCGTTATTTTCCCTGTGTTAACTCATGTTGTCTCTTACGGTGCCCTCACCACTAGCCATTTTCTTGACACGGTCGGTCTTATCACTGTGTCTACCGCCGGGTATTATCACGGGCGGTATGTACTGAGTAGTATCTACGCTGTCTGCGCCCTAGCTGCGCTTGTTTGCTTCGCCATTAGGTTGGTAAAGAATTGCATGTCCTGGCGCTACTCTTGCACCAGATATACTAATTTTCTTCTGGACACTAAGGGAAAACTCTATCGTTGGCGGTCACCCGTCATCATAGAGAAGGGGGGCAAGGTGGATGTCGGGGGTCATTTAATCGACCTAAAGAGAGTTGTGCTTGAAGGTTCCGCGGCAACCCCTGTAACCAAGATTTCAGCGGAACAATGGGGTTGTCCATAGACGACTTCTGCAATGACAGCACGGCTGTACAAAAGGTGCTTTTGGGCGTTTTCA

>CF1-houbeiyanshizi20251213-12

TGTTTTTAGCCTGTCTTTTTGCCATTCTGCTGGCAATTTGAATGTTCAGGTATGTTGGGGAAATGCTTGACCGCGGGCTGTTGCTCGCAATTGCCTTTTTTGTGGTGTATCGTGCCGTTCTGTTTAGTTGCGCTCGTCAACGCCAACAACGACAGCAGCTCCCACTTACAATTGATTTATAACCTAACGATATGTGAGCTGAATGGCACAGATTGGCTGAACAAACACTTTCCTTGGGCGGTGGAGACTTTCGTTATTTTTCCTGTGTTAACGCATGTTGTCTCTTACGGTGCCCTCACCACTAGCCATTTTCTTGACACGGTCGGTCTTATCACTGTGTCCACCGCCGGGTATTATCACGGGCGGTACGTATTGAGTAGCATCTACGCTGTCTGCGCCCTAGCTGCGCTTGTTTGCTTCGCCATTAGGTTGGTAAAGAATTGCATGTCCTGGCGCTACTCTTGCACCAGATATACTAATTTTCTTCTGGACACAAAGGGAAAACTCTATCGTTGGCGGTCACCCGTCATCATAGAGAAGGGGGGCAAGGTGGATGTTGGGGGTCATTTAATCGACCTAAAGAGAGTTGTGCTTGAAGGTTCCGCGGCAACCCCTGTAACCAAGATTTCAGCGGAACAATGGGGTCGTCCATAGACGACTTCTGCAATGACAGCACGGCTGTACAAAAGGTGCTTTTGGCGTTTTCA

>CF7-houbeibiantaoti20251215

GTTTTAGCCTGTCTTTTTGCCATTCTGCTGGCAATTTGAATGTTCAGGTATGTTGGGGAAATGCTTGACCGCGGGCTGCTGCTCGCAATTGCCTTTTTTGTGGTGTATCGTGCCGTTCTGTTTGGTTGCGCTCGTCAACGCCAACGACAACAGCAGCTCCCACTTACAATTGATTTATAACCTAACGATATGTGAGCTGAATGGCACAGATTGGCTGGAAAAACACTTTTCTTGGGCGGTGGAGACTTTCGTTATTTTTCCTGTGTTAACTCATGTTGTCTCTTACGGTGCCCTCACCACTAGCCATTTTCTTGACACGGTCGGTCTTATCACTGTGTCCACCGCCGGGTATTATCACGGGCGGTATGTATTGAGTAGCATCTACGCTGTCTGCGCCCTAGCTGCGCTTGTTTGCTTCGCCATTAGGTTGGCAAAGAATTGCATGTCCTGGCGCTACTCTTGCACCAGATATACTAATTTTCTTCTGGACACTAAGGGAAAACTCTATCGTTGGCGGTCACCCGTCATCATAGAGAAGGGGGGCAAGGTGGATGTTGGGGGTCATTTAATCGACCTAAAGAGAGTTGTGCTTGAAGGTTCCGCGGCAACCCCTGTAACCAAGATTTCAGCGGAACAATGGGGTCGTCCATAGACGACTTCTGCAATGACAGCACGGCTGTACAAAAGGTGCTTTTGG-CGTTTTCA

>CF11-peihuaizuzhi20251216

TGTTTTTAGGCCCGTCTTTTTGCCATTCTGCTGGCAATTTGAATGTTCAGGTATGTCGGGGAAATGCTTGACCGCGGGCTGTTGCTCGCAATTGCCTTTTTTGTGGTATATCGTGCCGTTCTGTTTTGTTGTGCTCGTCAACGCCAACAACAGCAGCAGCTCCCATTTACAGTTGATTTACAACCTGACGATATGTGAGCTGAATGGCACAGATTGGCTAAATAAAAGTTTTGATTGGGCAGTGGAGACCTTTGTTATTTTCCCTGTGTTGACTCATATTGTCTCCTATGGCGCCCTAACCACCAGCCATTTTCTTGACACAGTTGGCCTGATCACCGTGTCTACCGCTGGGTATTACCACGGGCGGTATGTCCTGAGCAGCATTTACGCCGTCTGCGCCCTAGCTGCGTTAACTTGCTTTGTCATCAGGCTAACAAAAAATTGCATGTCCTGGCGTTACTCATGCACCAGATACACTAACTTTCTTCTGGACACCAAGGGCAAACTCTATCGTTGGCGGTCTCCCGTCATCGTGGAGAAAGGGGGTAAAGTTGAGGTCGCAGGCCACCTGATCGACCTCAAAAGAGTTGTGCTTGACGGTTCCGCGGCAACCCCTATAACCAAAGTTTCAGCGGAACAATGGGGTCGTCCTTAGACGACTTCTGCTATGACAGCAAAGCCCCGCAAAAGGTGCTTTGGG

>CF-GC-baoyu4jianzuzhi-20251228-ORF5

ATGTTGGGGAAATGCTTGACCGCGGGCTGCTGCTCGCAATTGCTTTTTTTGTGGTGTATCGTGCCGTTCTGTTTAGTTTCGCTCGTCAACGCCAGCAACAACAGCAGCTCCCATTTACAGTTGATTTATAACCTGACGATATGTGAGCTGAATGGCACAGATTGGCTAAATAGCAGGTTTGATTGGGCAGTGGAGACCTTCGTCATTTTTCCTGTGTTGACTCATATCGTCTCCTATGGCGCCCTAACCACCAGCCACTTCCTTGACACAGTCGGCCTGATCACCGTGTCTACTGCTGGGTATTGCCACGGGCGGTATGTCTTGAGCAGTATTTACGCCGTCTGCGCCTTAGCTGCGCTAATTTGCTTCGTCATCAGGCTAACAAAAAATTGCATGTCCTGGCGTTACTCATGCACCAGATACACTAATTTTCTTCTGGACACCAAAGGAAAACTCTATCGTTGGCGGTCTCCCGTCATCGTAGAGAAAGGGGGCAAAGTTGAGGTCGAAGGCCACCTCATCGACCTTAAGAAAGTTGTGCTTGACGGTTCCGCGGCGACCCCTATAACCAAAGTTTCAGCGGAACAATGGGGTCGTCCTTAG

>CF3-peihuaiweigenxue20251219

TTGTTTTAGCCCTGTCTTTTTGCCATTCTGCTGGCAATTTGAATGTTCAGGTATGTTGGG

GAAATGCTTGACCGCGGGCTGCTGCTCGCAATTGCCTTTTTTATGGTGTATCGTGCCGT

TCTGTTTGGTTGCGCTCGTCAACGCCACCGACAACAGCAGCTCCCACTTACAATTGATT

TATAACCTAACGATATGTGAGCTGAATGGCACAGATTGGCTGAAAGAACACTTTTCTTG

GGCGGTGGAGACTTTCGTTATTTTTCCTGTGTTAACTCATGTTGTCTCTTACGGTGCCCT

CACCACTAGCCATTTTCTTGACACGGTCGGTCTTATCACTGTGTCCACCGCCGGGTATTA

TCACGGGCGGTATGTATTGAGTAGCATCTATGCTGTCTGCGCCCTAGCTGCGCTTGTTTG

CTTCGCCATTAGGTTGGCAAAGAATTGCATGTCCTGGCGCTACTCTTGCACCAGATATA

CTAATTTTCTTCTGGACACTAAGGGAAAACTCTATCGTTGGCGGTCACCCGTCATCATA

GAGAAGGGGGGCAAGGTGGATGTTGGGGGTCATTTAATCGACCTAAAGAGAGTTGTG

CTTGAAGGTTCCGCGGCAACCCCTGTAACCAAGATTTCAGCGGAACAATGGGGTCGTC

CATAGACGACTTCTGCAATGACAGCACGGCTGCACAAAAGGTGCTTTTGGGCGTTTTT

CA

>CF9-peihuailiuchan20251219

TGTTTTAGCCCTGTCTTTTTGCCATTCTGCTGGCAATTTGAATGTTCAGGTATGTTGGGG

AAATGCTTGACCGCGGGCTGCTGCTCGCAATTGCCTTTTTTGTGGTTTATCGTGCCGTT

CTGTTTAGTTGCGCTCGTCAACGCCAACGACAACAGCAGCTCCCACTTACAATTGATTT

ATAACCTAACGATATGTGAGCTGAATGGCACAGATTGGCTGAAAGACCACTTTCCTTGG

GCGGTGGAGACTTTCGTTATTTTTCCTGTGTTAACTCATATTGTCTCTTACGGTGCCCTC

ACCACTAGCCATTTTCTTGACACGGTCGGTCTTATCACTGTGTCCACCGCCGGGTATTAT

CACGGGCGGTATGTATTGAGTAGCATCTACGCTGTCTGCGCCCTAGCTGCGCTTGTTTG

CTTCGCCATTAGGCTGGTAAAGAATTGCATGTCCTGGCGCTACTCTTGCACCAGATATA

CTAATTTTCTTCTGGACACTAAGGGAAAACTCTATCGTTGGCGGTCACCCGTCATCATA

GAGAAGGGGGGCAAGGTGGATGTTGGCGGTCATTTAATCGACCTAAAGAGAGTTGTG

CTTGAAGGTTCCGCGGCAACCCCTGTAACCAAGATTTCAGCGGAACAATGGGGTCGTC

CATAGACAACTTCTGCAATGACAGCACGGCTGTACAAAAGGTGCTTTGGGGCGTTTTT

CAA

>CF11-houbeizuzhi20251219

GTTTTAGGCCTGTCTTTTTGCCATTCTGCTGGCAATTTGAATGTTCAGGTATGTTGGGGA

AATGCTTGACCGCGGGCTGCTGCTCGCAATTGCCTTTTTTGTGGTGTATCGTGCCGTTC

TGTTTAGTTGCGCTCGTCAACGCCAACAACGACAGCAGCTCCCACTTACAATTGATTTA

TAACCTAACGATATGTGAGCTGAATGGCACAGATTGGCTGAACAACCACTTTTCTTGGG

CGGTGGAGACCTTCGTCATTTTTCCTGTGTTGACTCATATCGTCTCCTATGGCGCCCTAA

CCACCAGCCACTTCCTTGACACAGTCGGCCTGATCACCGTGTCTACTGCTGGATATTAC

CACGGGCGGTATGTCTTGAGCAGTATTTACGCCGTCTGCGCCTTAGCTGCGCTAATTTG

CTTCGTCATCAGGCTAACAAAAAATTGCATGTCCTGGCGCTACTCTTGCACCAGATATA

CTAATTTTCTTCTGGACACAAAGGGAAATCTCTATCGTTGGCGGTCACCCGTCATCATA

GAGAAGGGGGGCAAGGTGGATGTTGGGGGTCATTTAATCGACCTAAAGAGAGTTGTG

CTTGAAGGTTCCGCGGCAACCCCTGTAACCAAGATTTCAGCGGAACAATGGGGTCGTC

CATAGACGACTTCTGCAATGACAGCACGGCTGTACAAAAGGTGCTTTTGGGCCGTTTT

TCAA

>CF17-peihuaiweigenxue20251219

TGTTTTAGCCCTGTCTTTTTGCCATTCTGCTGGCAATTTGAATGTTCAGGTATGTTGGGG

AGATGCTTGATCGCGGGCTGTTGCTCGCAATTGCCTTTTTTGTGGTATATCGTGCCGTTC

TGTTTTGTTGCGCTCGTCAACGCCAACAACAGCAGCAGCTCCCATTTACAGTTGATTTA

TAACCTGACGATATGTGAGCTGAATGGCACAGATTGGCTAAATAAAAGTTTTGATTGGG

CAGTGGAGACCTTTGTTATTTTCCCTGTGTTGACTCATATTGTCTCCTATGGCGCCCTAA

CCACCAGCCATTTCCTTGACACAGTTGGCCTGATCACCGTGTCTACCGCTGGGTATTAC

CACGGGCGGTATGTCCTGAGTAGCATTTACGCCGTCTGCGCCCTAGCTGCGTTAACTTG

CTTTGTCATCAGGCTAACAAAAAATTGCATGTCCTGGCGTTACTCGTGCACCAGATACA

CTAACTTTCTTCTGGACACCAAGGGCAAACTCTATCGTTGGCGGTCTCCCGTCATCGTT

GAGAAAGGGGGTAAAGTTGAGGTCGCAGGCCACCTGATCGACCTCAAGAGAGTTGTG

CTTGACGGTTCCGCGGCAACCCCTATAACCAAAGTTTCAGCGGAACAATGGGGTCGTC

CTTAGACGACTTCTGCTATGACAGCACAGCCCCGCTAAAGGTGCTTTTGGGCGTTTTCA

>CF-8-houbeiyanshizi20251219

TGTTTTAGCCTGTCTTTTTGCCATTCTGCTGGCGATTTGAATGTTCAGGTATGTTGGGGA

AATGCTTGACCGCGGGCTGCTGCTCGCAATTGCCTTTTTTTTGGTGTATCGTGCCGTTCT

GTTTAGTTGCGCTCGTCAACGCCAACGACAACAGCAGCTCCCACTTGCAATTGATTTAT

AACCTAACGATATGTGAGCTGAATGGCACAGATTGGCTGAAAGAACACTTTTCTTGGG

CGGTGGAGACCTTCGTTATTTTTCCTGTGTTAACTCATATTGTCTCCTATGGCGCCCTAA

CCACCAGTCACTTCCTTGACACAGTCGGCCTGATCACCGTGTCTACTGCTGGATATTAC

CACGGGCGGTATGTCTTGAGCAGTATTTACGCCGTCTGCGCCTTAGCTGCGCTAATTTG

CTTCGTCATCAGGCTAACAAAAAATTGCATGTCCTGGCGTTACTCATGCACCAGATACA

CTAATTTTCTCCTGGACACCAAGGGAAAACTCTATCGTTGGCGGTCACCCGTCATCATA

GAGAAGGGGGGCAAGGTGGATGTTGGGGGTCATTTAATCGACCTAAAGAGAGTTGTG

CTTGAAGGTTCCGCGGCAACCCCTGTAACCAAGATTTCAGCGGAACAATGGGGTCGTC

CATAGACGACTTCTGCAATGACAGCACGGCTGTACAAAAGGTGCTTTGGGC

>CF13-fenmiantaiyixue20251229

TTGTTTTAGCCTGTCTTTTTGCCATTCTGCTGGCGATTTGAATGTTCAGGTATGTTGGGG

AAATGCTTGACCGCGGGCTGCTGCTCGCAATTGCCTTTTTTTTGGTGTATCGTGCCGTT

CTGTTTAGTTGCGCTCGTCAACGCCAACGACAACAGCAGCTCCCACTTGCAATTGATTT

ATAACCTAACGATATGTGAGCTGAATGGCACAGATTGGCTGAAAGAACACTTTTCTTGG

GCGGTGGAGACTTTCGTTATTTTTCCTGTGTTAACTCATATTGTCTCCTATGGCGCCCTA

ACCACCAGTCACTTCCTTGACACAGTCGGCCTGATCACCGTGTCTACTGCTGGATACTA

CCACGGGCGGTATGTCTTGAGCAGTATTTACGCCGTCTGCGCCTTAGCTGCGCTAATTT

GCTTCGTCATCAGGCTAACAAAAAATTGCATGTCCTGGCGTTACTCATGCACCAGATAC

ACTAATTTTCTCCTGGACACCAAGGGAAAACTCTATCGTTGGCGGTCACCCGTCATCAT

AGAGAAGGGGGGCAAGGTGGATGTTGGGGGTCATTTAATCGACCTAAAGAGAGTTGT

GCTTGAAGGTTCCGCGGCAACCCCTGTAACCAAGATTTCAGCGGAACAATGGGGTCGT

CCATAGACGACTTCTGCAATGACAGCACGGCTGTACAAAAGGTGCTTTGGGCCGTTTT

TC

>CF5-houbeiyanshizi20251229

TGTTTTAGCCCTGTCTTTTTGCCATTCTGCTGGCAGTTTAAATGTTCAGGTATGTTGGGG

AAATGCTTGACCGCGGGCTGCTGCTCGCAATTGCTTTTTTTGTGGTGTATCGTGCCGTT

CTGTTTAGTTTCGCTCGTCAACGCCAGCAACAGCAGCAGCTCCCATTTACAGTTGATTT

ATAACCTGACGATATGTGAGCTGAATGGCACAGATTGGCTAAATCGCACTTTTGATTGG

GCAGTGGAGACCTTCGTTATTTTTCCTGTGTTGACTCATATCGTCTCCTATGGCGCCCTA

ACCACCAGCCACTTCCTTGACACAGTCGGCCTGATCACCGTGTCTACTGCTGGATATTA

CCACGGGCGGTATGTCTTGAGCAGTATTTACGCCGTCTGCGCCTTAGCTGCGCTAATTT

GCTTCGTCATCAGGCTAACAAAAAATTGCATGTCCTGGCGTTACTCATGCACCAGATAC

ACTAATTTTCTTCTGGACACCAAGGGAAAACTCTATCGTTGGCGGTCTCCCGTCATCGT

GGAAAAAGGGGGTAAAGTTGAGGTCGAAGGCCACCTCATCGACCTCAAGAAAGTTGT

GCTTGATGGTTCCGCGGCGACCCCTATAACCAAAGTTACAGCGGAACAATGGGGTCGT

CCTTAGACGACTTCTGCTATGACAGCACAGCCCCGCAAAAGGTGCTTTTGGG

>CF15-peihuailiuchan20260102

GTTTTAGCCTGTCTTTTTGCCATTCTGCTGGCAATTTGAATGTTCAGGTATGTTGGGGAA

ATGCTTGACCGCGGGCTGCTGCTCGCAATTGCCTTTTTTGTGGTGTATCGTGCCGTTCT

GTTTAGTTGCGCTCGTCAACGCCAACAACGACAGCAGCTCCCACTTGCAATTGATTTAT

AACCTAACGATATGTGAGCTGAATGGCACAGATTGGCTGAACAAACACTTTCCTTGGG

CGGTGGAGACTTTCGTTATTTTTCCTGTGTTAACGCATGTTGTCTCTTGTGGTGCCCTCA

CCACTAGCCATTTTCTTGACACGGTCGGTCTTATCACTGTGTCCACCGCCGGGTATTATC

ACGGGCGGTATGTATTGAGTAGCATCTACGCTGTCTGCGCCCTAGCTGCGCTCGTTTGC

TTCGCCATTAGGTTGGTAAAGAATTGCATGTCCTGGCGCTACTCTTGCACCAGACATAC

TAATTTTCTTCTGGACACAAAGGGAAAACTCTATCGTTGGCGGTCACCCGTCATCATAG

AGAAGGGGGGCAAGGTGGATGTTGGGGGTCATTTAATCGACCTAAAGAGAGTTGTGCT

TGAAGGTTCCGCGGCAACCCCTGTAACCAAGATTTCAGCGGAACAATGGGGTCGTCCA

TAGACGACTTCTGCAATGACAGCACGGCTGTACAAAAGGTGCTTTTGGGCGTTTTCA

>CF-7-zuzhi20260101

TGTTTTAGCCCTGTCTTTTTGCCATTCTGCTGGCAATTTGAATGTTCAGGTATGTTGGGG

AAATGCTTGACCGCGGGCTGCTGCTCGCAATTGCTTTTTTTGTGGTGTATCGTGCCGTT

CTGTTTAGTTGCGCTCGTCAACGCCAACGACAACAGCAGCTCCCACTTACAATTGATTT

ATAACCTAACGATATGTGAGCTGAATGGCACAGATTGGCTGAAAGAACACTTTTCTTGG

GCGGTGGAGACTTTCGTTATTTTTCCTGTGTTAACTCATGTTGTCTCTTACGGTGCCCTC

ACCACTAGCCATTTTCTTGACACGGTCGGTCTTATCACTGTGTCCACCGCCGGGTATTAT

CACAGGCGGTATGTATTGAGTAGCATCTACGCTGTCTGCGCCCTAGCTGCGCTTGTTTG

CTTCACCATTAGGTTGGTAAAGAATTGCATGTCTTGGCGCTACTCTTGCACCAGATATAC

TAATTTTCTTCTGGACACTAAGGGAAAACTCTATCGTTGGCGGTCACCCGTCATCATAG

AGAAGGGGGGCAAGGTGGATGTTGGGGGTCATTTAATCGACCTAAAGAGAGTTGTGCT

TGAAGGTTCCGCGGCAACCCCTGTAACCAAGATTTCAGCGGAACAATGGGGTCGTCCA

TAGACGACTTCTGCAATGACAGCACGGCTGTACAAAAGGTGCTTTTGGGCGTTTTCA

>CF-7-zuzhi20260101

TGTTTTAGGCCCCGTCTTTTTGCCATTCTGCTGGCAATTTGAATGTTCAGGTATGTTGGG

GAAATGCTTGACCGCGGGCTGCTGCTCGCAATTGCTTTTTTTGTGGTGTATCGTGCCGT

TCTGTTTAGTTGCGCTCGTCAACGCCAACGACAACAGCAGCTCCCACTTACAATTGATT

TATAACCTAACGATATGTGAGCTGAATGGCACAGATTGGCTGAAAGAACACTTTTCTTG

GGCGGTGGAGACTTTCGTTATTTTTCCTGTGTTAACTCATGTTGTCTCTTATGGTGCCCT

CACCACTAGCCATTTTCTTGACACGGTCGGTCTTATCACTGTGTCCACCGCCGGGTATTA

TCACGGGCGGTATGTATTGAGTAGCATCTACGCTGTCTGCGCCCTAGCTGCGCTTGTTT

GCTTCACCATTAGGTTGGTAAAGAATTGCATGTCCTGGCGCTACTCTTGCACCAGATAT

ACAAATTTTCTTCTGGACACTAAGGGAAAACTCTATCGTTGGCGGTCACCCGTCATCAT

AGAGAAGGGGGGCAAGGTGGATGTTGGGGGTCATTTAATCGACCTAAAGAGAGTTGT

GCTTGAAGGTTCCGCGGCAACCCCTGTAACCAAGATTTCAGCGGAACAATGGGGTCGT

CCATAGACGACTTCTGCAATGACAGCACGGCTGTACAAAAGGTGCTTGGGG

>CF23-chanfangzizhuxueqing20260106

GTTTTAGCCCTGTCTTTTTGCCATTCTGCTGGCAATTTGAATGTTCAGGTATGTTGGGGA

AATGCTTGACCGCGGGCTGCTGCTCGCAATTGCCTTTTTTGTGGTGTATCGTGCCGTTC

TGTTTAGTTGCGCTCGTCAACGCCAACAACGACAGCAGCTCCCACTTACAATTGATTTA

TAACCTAACGATATGTGAGCTGAATGGCACAGATTGGCTGAAAGAACACTTTTCTTGG

GCGGTGGAGACTTTCGTTATTTTTCCTGTGTTGACTCATATCGTCTCCTATGGCGCCCTA

ACCACCAGCCACTTCCTTGACACAGTCGGCCTGATCACCGTGTCTACTGCTGGATATTA

CCACGGGCGGTATGTCTTGAGCAGCATTTACGCCGTCTGCGCCTTAGCTGCGCTAATTT

GCTTCGTCATCAGGCTAACAAAAAATTGCATGTCCTGGCGCTACTCTTGCACCAGATAT

ACTAATTTTCTTCTGGACACTAAGGGAAAACTCTATCGCTGGCGGTCACCCGTCATCAT

AGAGAAGGAGGGCAAGGTGGACGTTGGGGGTCATTTAATCGACTTAAAGAGAGTTGT

GCTTGAAGGTTCCGCGGCAACCCCTGTAACCAAGATTTCAGCGGAACAATGGGGCCGT

CCATAGACGACTTCTGCAATGACAGCACGGCTGTACAAAAGGTGCTTTTGGGCGTTTT

CA

>CF12-baoyuB3zuzhi20260106

CCCCGCTTTTTGCCATTCTGCTGGCAATTTGAATGTTCAGGTATGTCGGGGAAATGCTT

GACCGCGGGCTGTTGCTCGCAATTGCCTTTTTTGTGGTATATCGTGCCGTTCTGTTTTGT

TGTGCTCGTCAACGCCAACAACAGCAGCAGCTCCCATTTACAGTTGATTTACAACCTG

ACGATATGTGAGCTGAATGGCACAGATTGGCTAAATAAAAGTTTTGATTGGGCAGTGG

AGACCTTTGTTATTTTCCCTGTGTTGACTCATATTGTCTCCTATGGCGCCCTAACCACCA

GCCATTTTCTTGACACAGTTGGCCTGATCACCGTGTCTACCGCTGGGTATCACCACGGG

CGGTATGTCCTGAGCAGCATTTACGCCGTCTGCGCCCTAGCTGCGTTAACTTGCTTTGT

CATCAGGCTAACAAAAAATTGCATGTCCTGGCGTTACTCATGCACCAGATACACTAACT

TTCTTCTGGACACCAAGGGCAAACTCTATCGTTGGCGGTCTCCCGTCATCGTGGAGAA

AGGGGGTAAAGTTGAGGTCGCAGGCCACCTGATCGACCTCAAAAGAGTTGTGCTTGA

CGGTTCCGCGGCAACCCCTATAACCAAAGTTTCAGCGGAACAATGGGGTCGTCCTTAG

ACGACTTCTGCTATGACAGCAAAGCCCCGCAAAAGGTGCTTTGGG

>CF12-baoyuB2zuzhi20260106

CCCCGCTTTTTGCCATTCTGCTGGCAATTTGAATGTTCAGGTATGTTGGGGAAATGCTT

GACCGCGGGCTGCTGCTCGCAATTGCCTTTTTTATGGTTTATCGTGCCGTTCTGTTTAGT

TGCGCTCGTCAACGCCAACGACAACAGCAGCTCCCACTTACAATTGATTTATAACCTAA

CGATATGTGAGCTGAATGGCACAGATTGGCTGAAAGACCACTTTTCTTGGGCGGTGGA

GACTTTCGTTATTTTTCCTGTGTTAACTCATATTGTCTCTTACGGTGCCCTCACCACTAG

CCATTTTCTTGACACGGTCGGCCTTATCACTGTGTCCACCGCCGGGTATTATCACGGGC

GGTATGTATTGAGTAGCATCTACGCTGTCTGCGCCCTAGCTGCGCTTGTTTGCTTCGCCA

TTAGGTTGGTAAAGAATTGCATGTCCTGGCGCTACTCTTGCACCAGATATACTAATTTTC

TTCTGGACACTAAGGGAAAGCTCTATCGTTGGCGGTCACCCGTCATCATAGAGAAGGG

GGGCAAGGTGGATGTTGGTGGTCATTTAATCGACCTAAAGAGAGTTGTGCTTGAAGGT

TCCGCGGCAACCCCTGTAACCAAGATTTCAGCGGAACAATGGGGTCGTCCATAGACAA

CTTCTGCAATGACAGCACGGCTGTACAAAAGGTGCTTGGGGGGTTTCA

>CF-GC-Y15yanshizi20260108

CCCGCTTTTTGCCATTCTGCTGGCAATTTAAATGTTCAGGTATGTTGGGGAAATGCTTG

ACCGCGGGCTGCTGCTCGCAATTGCTTTTTTTGTGGTGTATCGTGCCGTTCTGTTTAGTT

TCGCTCGTCAACGCCAGCAACAACAGCAGCTCCCATTTACAGTTGATTTATAACCTGAC

GATATGTGAGCTGAATGGCACAGATTGGCTAAATAGCAGGTTTGATTGGGCAGTGGAG

ACCTTCGTCATTTTTCCTGTGTTGACTCATATCGTCTCCTATGGCGCCCTAACCACCAGC

CACTTCCTTGACACAGTCGGCCTGATCACCGTGTCTACTGCTGGGTATTGCCACGGGCG

GTATGTCTTGAGCAGTATTTACGCCGTCTGCGCCTTAGCTGCGCTAATTTGCTTCGTCAT

CAGGCTAACAAAAAATTGCATGTCCTGGCGTTACTCATGCACCAGATACACTAATTTTC

TTCTGGACACCAAAGGAAAACTCTATCGTTGGCGGTCTCCCGTCATCGTAGAGAAAGG

GGGCAAAGTTGAGGTCGAAGGCCACCTCATCGACCTTAAGAAAGTTGTGCTTGACGG

TTCCGCGGCGACCCCTATAACCAAAGTTTCAGCGGAACAATGGGGTCGTCCTTAGACG

ACTTCTGCTATGACAGCACAGCCCCGCTAAAGGTGTTG

>CF-CF1-20250712

ATGTTGGGGAAATGCTTGACCGCGGGCTGCTGCTCGCAATTGCTTTTTTTGTGGTGTATCGTGCCGTTCTGTTTAGTTGCGCTCGTCAACGCCGACAACAACAGCAGCTCCCACTTACAGTTGATTTATAACCTAACGATATGTGAGCTGAATGGCACAGATTGGCTGAAGGAACATTTTTCTTGGGCAGTAGAGACTTTCGTTATATTTCCTGTGTTAACTCATATTGTCTCTTACGGTGCCCTCACCACTAGCCATTTTCTTGACACGGTTGGCCTGATCACTGTGTCCACTGCCGGGTATTATCACAGGCGGTATGTACTGAGTAGCATCTACGCTGTCTGCGCCCTAGCTGCGCTTGTTTGCTTCACCATTAGGTTCGTAAAAAATTGCATGTCCTGGCGCTACTCTTGCACCAGATATACTAACTTTCTTCTGGACACTAAGGGAAAACTCTATCGTTGGCGGTCACCTGTCATCATAGAGAAGGGGGGCAAGGTGGATGTTGGTGGTCATTTAATTGACCTAAAGAGAGTTGTGCTTGAAGGTTCCGCGGCGACTCCTGTAACCAGGATTTCAGCGGAACAATGGGGTCGTCCATAG

>CF1-20251213-12

TGTTTTTAGCCTGTCTTTTTGCCATTCTGCTGGCAATTTGAATGTTCAGGTATGTTGGGGAAATGCTTGACCGCGGGCTGTTGCTCGCAATTGCCTTTTTTGTGGTGTATCGTGCCGTTCTGTTTAGTTGCGCTCGTCAACGCCAACAACGACAGCAGCTCCCACTTACAATTGATTTATAACCTAACGATATGTGAGCTGAATGGCACAGATTGGCTGAACAAACACTTTCCTTGGGCGGTGGAGACTTTCGTTATTTTTCCTGTGTTAACGCATGTTGTCTCTTACGGTGCCCTCACCACTAGCCATTTTCTTGACACGGTCGGTCTTATCACTGTGTCCACCGCCGGGTATTATCACGGGCGGTACGTATTGAGTAGCATCTACGCTGTCTGCGCCCTAGCTGCGCTTGTTTGCTTCGCCATTAGGTTGGTAAAGAATTGCATGTCCTGGCGCTACTCTTGCACCAGATATACTAATTTTCTTCTGGACACAAAGGGAAAACTCTATCGTTGGCGGTCACCCGTCATCATAGAGAAGGGGGGCAAGGTGGATGTTGGGGGTCATTTAATCGACCTAAAGAGAGTTGTGCTTGAAGGTTCCGCGGCAACCCCTGTAACCAAGATTTCAGCGGAACAATGGGGTCGTCCATAGACGACTTCTGCAATGACAGCACGGCTGTACAAAAGGTGCTTTTGGCGTTTTCA

>CF-2-20250812-83

ATGTTGGAGAAATGCTTGACCGCGGGCTGTTGCTCGCAATTGCTTTCTTTGTGGTGTATCGTGCCGTTCTGTTT

TGCTGTGCTCGCCAACGCCAGCAACGACAGCAGCTCCCATCTACAGCTGATTTACAACTTGACGCTATGTGAGC

TGAATGGCACAGATTGGCTAGCTAACAAATTTGATTGGGCAGTGGAGAGTTTTGTCATCTTTCCCGTTTTGACT

CACATTGTCTCCTATGGTGCCCTCACTACCAGCCATTTCCTTGACACAGTCGCTTTAGTCACTGTGTCTACCGC

CGGGTTTGTTCACGGGCGGTATGTCCTAAGTAGCATCTACGCGGTCTGTGCCCTGGCTGCGTTGACTTGCTTCG

TCATTAGGTTTGCAAAGAATTGCATGTCCTGGCGCTACGCGTGTACCAGATATACCAACTTTCTTCTGGACACT

AAGGGCATACTCTATCGTTGGCGGTCGCCTGTCATCATAGAGAAAAGGGGCAAAGTTGAGGTCGAAGGTCATCT

GATCGACCTCAAAAGAGTTGTGCTTGATGGTTCCGTGGCAACCCCTATAACCAGAGTTTCAGCGGAACAATGGG

GTCGTCCTTAG

>CF-2-20250812-84

ATGTTGGGGAAATGCTTGACCGCGGGCTGCTGCTCGCGATTGCTTTTTTTGTGGTGTATCGTGCCGTTCTGTTT

GGTTTCGCTCGTCAACGCCAGCAACAGCAGCAGCTCCCATTTACAGTTGATTTATAACCTGACGATATGTGAGC

TGAATGGCACAGATTGGCTAGATCGCAAATTTGATTGGGCAGTGGAGACCTTCGTTATTTTTCCTGTATTGACT

CATATCGTCTCCTATGGCGCCCTAACCACCAGCCACTTCCTTGACACAGTCGGCCTGATCACCGTGTCTACTGC

CGGATATTACCACGGGCGGTATGTCTTGAGCAGTATTTATGCCGTCTGCGCCTTAGCTGCGCTAATTTGCTTCG

TCATCAGGCTAACAAAAAATTGCATGTCCTGGCGTTACTCATGCACCAGATACACTAATTTTCTTCTGGACACC

AAGGGAAAACTCTATCGTTGGCGGTCTCCCGTCATCGTAGAAAAAGGGGGTAAAGTTGAGGTCGAAGGCCACCT

CATCGACCTCAAGAAAGTTGTGCTTGATGGTTCCGCGGCGACCCCTATAACCAAAGTTTCAGCGGAACAATGGG

GTCGTCCTTAG

>CF-CF2-20260120-1

atgttggggagatgcttgaccgcgggctgttgctcgcaattgccttttttgtggtatatcgtgccgttctgctttgttgtgctcgtcaacgccaacaacagcagcagctcccatttacaattgatttataacctgacgatatgtgagctgaatggcacagattggctaaataaaaattttgattgggcagtggagacctttgttattttccctgtgttgactcatattgtctcctatggcgccctaaccaccagccatttccttgacacagttggcctgatcaccgtgtctactgctggatattaccacgggcggtatgtctt-agcagtatttacgccgtctgcgccttagctgcgctaatttgcttcgtcatcaggctaacaaaaaattgcatgtcctggcgttactcatgcaccagatacactaattttcttctggacaccaagggaaaactctatcgttggcggtctcccgtcatcgtggaaaaagggggtaaagttgaggtcgaaggccacctcatcgacctcaagaaagttgtgcttgatggttccgcggcgacccctataaccaaagttacagcggaacaatggggtcgtccttag

>CF-CF3-20250604-125

ATGTTGGAGAAATGCTTGACCGCGGGCTGTTGCTCGCAATTGCTTTCTTTGTGGTGTATCGTGCCGTTCTGTTTTGCTGTGCTCGCCAACGCCAGCAACGACAGCAGCTCCCATCTACAGCTGATTTACAACTTGACGCTATGTGAGCTGAATGGCACAGATTGGCTAGCTAACAAATTTGATTGGGCAGTGGAGAGTTTTGTCATCTTTCCCGTTTTGACTCACATTGTCTCCTATGGTGCCCTCACTACCAGCCATTTCCTTGACACAGTCGCTTTAGTCACTGTGTCTACCGCCGGGTTTGTTCACGGGCGGTATGTCCTAAGTAGCATCTACGCGGTCTGTGCCCTGGCTGCGTTGACTTGCTTCGTCATTAGGTTTGCAAAGAATTGCATGTCCTGGCGCTACGCGTGTACCAGATATACCAACTTTCTTCTGGACACTAAGGGCGGACTCTATCGTTGGCGGTCGCCTGTCATCATAGAGAAAAGGGGCAAAGTTGAGGTCGAAGGTCATCTGATCGACCTCAAAAGAGTTGTGCTTGATGGTTCCGTGGCAACCCCTATAACCAGAGTTTCAGCGGAACAATGGGGTCGTCCTTAG

>CF-CF3-20250613-5

ATGTGGGGGAAATGCTTGACCGCGGGCTGCTGCTCGCAATTGCCTTTTTTGTGGTGTATCGTGCCGTTCTGTTTAGTTGCGCTCGTCAACGCCAACGACAACAGCAGCTCCCACTTACAATTGATTTATAACCTAACGATATGTGAGCTGAATGGCACAGATTGGCTGAAAGACCACTTTTCTTGGGCGGTGGAGACTTTCGTTATTTTTCCTGTGTTAACTCATATTGTCTCTTACGGTGCCCTCACCACTAGCCATTTTCTTGACACGGTCGGTCTTATCACTGTGTCCACCGCCGGGTATTATCACGGGCGGTATGTATTGAGTAGCATCTACGCTGTCTGCGCCCTAGCTGCGCTTGTTTGCTTCGCCATTAGGTTGGTAAAGAATTGCATGTCCTGGCGCTACTCTTGCACCAGATATACTAATTTTCTTCTGGACACTAAGGGAAAACTCTATCGTTGGCGGTCACCCGTCATCATAGAGAAGGGGGGCAAGGTGGATGTTGGAGGTCATTTAATCGACCTAAAGAGAGTTGTGCTTGAAGGTTCCGCGGCAACCCCTGTAACCAAGATTTCAGCGGAACAATGGGGTCGTCCATAG

>CF-CF3-20260129-3

atgttggggaaatgcttgaccgcgggctgctgctcgcaattgctttttttgtggtgtatcgtgccgttctgtttagtttcgctcgtcaacgccagcagcagcaacagctcccatttacagttgatttataacctgacgatatgtgagctgaatggcacagattggctaaatcgcacttttgattgggcagtggagaccttcgttatttttcctgtattgactcatatcgtctcctatggcgccctaaccaccagccacttccttgacacagtcggcctgatcaccgtgtctactgctggatattaccacaggcggtatgtcttgagcagtatttacgccgtctgcgccttagctgcgctaatttgcttcgccatcaggctaacaaaaaattgcatgtcctggcgttactcatgcaccagatacactaattttcttctggacaccaagggaaaactctatcgttggcggtctcccgtcatcgtagaaaaagggggtaaagttgaggtcgaaggccacctcatcgacctcaagaaagttgtgcttgatggttccgcggcgacccctataaccaaagtttcagcggaacaatggggtcgtccttag

>CF-CF4-20250809-70

ATGTTGGGGAAATGCTTGACCGCGGGTTATTGCTCGCAATCGCTTTTTTTGTGGTGTATCGTGCCATTCTGTTT

TGCTGTGCTCGTCAACGCCAGCAACGACAGCAGCTCCCACTTACAGTTGATCTATAACCTGACAATATGTGAGC

TGAATGGCACAGACTGGTTGAACAAAAAATTTGACTGGGCAGTAGAGACCTTCGTTATTTTTCCTGTCTTGACC

CATATTGTCTCTTACGGCGCCCTTACCACTAGCCATTTTCTTGACACGGTCGGCCTAATCACCGTATCCACCGC

TGGGTATTATCATAAGCGGTATGTATTGAGTAGCATCTACGCTGTCTGTGCCCTGGCCGCGTTGGTTTGTTTCG

CCATTAGGTTGGCAAAAAATTGTATGTCCTGGCGCTACTCATGCACCAGATATACCAATTTTCTTCTGGACTCT

AAGGGCAGACTCTACCGTTGGCGGTCACCCGTCATCATAGAGAAAGAGGGCAAAGTTGATGTGGGGGGTCAGTT

AATCGACCTCAAGAGAGTAGTGCTTGATGGTTCCGCGGCGACCCCTGTAACCAAGATTTCAGCGGAACAATGGG

GTCGTCCATAG

>CF-CF4-20250809-71

ATGTTGGGGAAATGCTTGACCGCGGGCTGCTGCTCGCAATTGCCTTTTTTGTGGTGTATCGTGCCGTTCTGTTT

AGTTGCGCTCGTCAACGCCAACGACAACAGCAGCTCCCACTTACAATTGATTTATAACCTAACGATATGTGAGC

TGAATGGCACAGATTGGCTGAAAGACCACTTTTCTTGGGCGGTGGAGACTTTCGTTATTTTTCCTGTGTTAACT

CATATTGTCTCTTACGGTGCCCTCACCACTAGCCATTTTCTTGACACGGTCGGTCTTATCACTGTGTCCACCGC

CGGGTATTATCACGGGCGGTATGTATTGAGTAGCATCTACGCTGTCTGCGCCCTAGCTGCGCTTGTTTGCTTCG

CCATTAGGTTGGTAAAGAATTGCATGTCCTGGCGCTACTCTTGCACCAGATATACTAATTTTCTTCTGGACACT

AAGGGAAAACTCTATCGTTGGCGGTCACCCGTCATCATAGAGAGGGGGGGCAAGGTGGATGTTGGTGGTCATTT

AATCGACCTAAAGAGAGTTGTGCTTGAAGGTTCCGCGGCAACCCCTGTAACCAAGATTTCAGCGGAACAATGGG

GTCGTCCATAG

>CF-CF4-20250809-72

ATGTTGGAGAAATGCTTGACCGCGGGCTGTTGCTCGCAATTGCTTTCTTTGTGGTGTATCGTGCCGTTCTGTTT

TGCTGTGCTCGCCAACGCCAGCAACGACAGCAGCTCCCATCTACAGCTGATTTACAACTTGACGCTATGTGAGC

TGAATGGCACAGATTGGCTAGCTAACAAATTTGATTGGGCAGTGGAGAGTTTTGTCATCTTTCCCGTTTTGACT

CACATTGTCTCCTATGGTGCCCTCACTACCAGCCATTTCCTTGACACAGTCGCTTTAGTCACTGTGTCTACCGC

CGGGTTTGTTCACGGGCGGTATGTCCTAAGTAGCATCTACGCGGTCTGTGCCCTGGCTGCGTTGACTTGCTTTG

TCATTAGGTTTGCAAAGAATTGCATGTCCTGGCGCTACGCGTGTACCAGATATACCAACTTTCTTCTGGACACT

AAGGGCATACTCTATCGTTGGCGGTCGCCTGTCATCATAGAGAAAAGGGGCAAAGTTGAGGTCGAAGGTCATCT

GATCGACCTCAAAAGAGTTGTGCTTGATGGTTCCGTGGCAACCCCTATAACCAGAGTTTCAGCGGAACAATGGG

GTCGTCCTTAG

>CF4-20251101-14

CCCCGCTTTTTGCCATTCTGCTGGCAATTTGAATGTTCAGGTATGTTGGGGAAATGCTTGACCGCGGGCTGCTGCTCGCAATTGCCTTTTTTGTGGTGTATCGTGCCGTTCTGTTTAGTTGCGCTCGTCAACGCCAACGACAACAGCAGCTCCCACTTACAATTGATTTATAACCTAACGATATGTGAGCTGAATGGCACAGATTGGCTGAAAGACCACTTTCCTTGGGCGGTGGAGACTTTCGTTATTTTTCCTGTGTTAACTCATATTGTCTCTTACGGTGCCCTCACCACTAGCCATTTTCTTGACACGGTCGGTCTTATCACTGTGTCCACCGCCGGGTATTATCACAGGCGGTATGTATTGAGTAGCATCTACGCTGTCTGCGCCCTAGCAGCGCTTGTTTGCTTCGCCATTAGGTTGGTAAAGAATTGCATGTCCTGGCGCTACTCTTGCACCAGATATACTAATTTTCTTCTGGACACTAAGGGAAAACTCTATCGTTGGCGGTCACCCGTCATCATAGAGAAGGGGGGCAAGGTGGATGTTGGTGGTCATTTAATCGACCTAAAGAGAGTTGTGCTTGAAGGTTCCGCGGCAACCCCTGTAACCAAGATTTCAGCGGAACAATGGGGTCGTCCATAGACAACTTCTGCAATGACAGCACGGCTGTACAAAAGGTGTTG

>CF-CF4-20260120-1

atgtcggggaaatgcttgaccgcgggctgatgctcgcaattgccttttttgtggtatatcgtgccgttctgttttgttgtgctcgtcaacgccaacaacagcagcagctcccatttacagttgatttataacctgacgatatgtgagctgaatggcacagattggctaaataagagttttgattgggcagtggagacctttgttatcttccctgtgttgactcatattgtctcctatggcgccctaaccaccagccatttccttgacacagttggcctgatcaccgtgtctgccgctgggtattgccacgggcggtatgtcctgagcagcatttacgccgtctgcgccctagctgcgttaacttgctttgtcatcaggctaacaaaaaattgcatgtcctggcgttactcatgcaccagatacactaactttcttctggacaccaagggcaaactctttcgttggcggtctcccgtcatcgtggagaaagggggtaaagttgaggtcgcaggccacctgatcgacctcaagagagttgtgcttgacggttccgcggcaacccctaaaaccaaagtttcagcggaacaatggggtcgtccttag

>CF-CF4-20260120-2

atgttggggaaatgcttgaccgcgggctgctgctcgcaattgctttttttgtg--gtatcgtgccgttctgtttagttgcgctcgtcaacgccaacgacaacagcagctcccacttacaattgatttataacctaacgatatgtgagctgaatggcacagattggctgaaagaccacttttcttgggcggtggagactttcgttatctttcctgtgttaactcatattgtctcttacggtgccctcaccaccagccattttcttgacacggtcggtcttatcactgtgtccaccgccgggtattatcacgggcggtatgtattgagtagcatctacgctgtctgcgccctagctgcgcttgtttgcttcgccattaggttggtaaagaattgcatgtcctggcgctactcttgcaccagatatactaattttcttctggacactaagggaaaactctatcgttggcggtcacccgtcatcatagagaaggggggcagggtggatgttggtggtcatttaatcgacctaaagagagttgtgcttgaaggttccgcggcaacccctgtaaccaagatttcagcggaacaatggggtcgtccatag

>CF-CF4-20260120-3

atgttggggaaatgcttgaccgcgggctgctgctcgcaattgctttttttgtggtgtatcgtgccgttctgtttagttgcgctcgtcaacgccaacgacaacagcagctcccacttacaattgatttatgacctaacgatatgtgagctgaatggcacagattggctgaaagaccacttttcttgggcggtggagactttcgttatctttcctgtgttaactcatattgtctcttacggtgccctcaccaccagccattttcttgacacggtcggtcttatcactgtgtccaccgccgggtattatcacgggcggtatgtattgagtagcatctacgctgtctgcgccctagctgcgcttgcttgcttcgccattaggttggtaaagaattgcatgtcctggcgctactcttgcaccagatatactaattttcttctggacactaagggaaaactctatcgttggcggtcacccgtcatcatagagaaggggggcaaggtggatgttggtggtcatttgatcgacctaaagagagttgtgcttgaaggttccgcggcaacccctgtaaccaagatttcagcggaacaatggggtcgtccatag

>CF-CF4-20260120-4

atgtcggggaaatgcttgaccgcgggctgttgctcgcaattgccttttttgtggtatatcgtgccgttctgttttgttgtgctcgtcaacgccaacaacagcagcagctcccatttacagttgatttataacctgacgatatgtgagctgaatggcacagattggctaaataagagttttgattgggcagtggagacctttgttatcttccctgtgttgactcatattgtctcctatggcgccctaaccaccagccatttccttgacacagttggcctgatcaccgtgtctaccgctgggtattgccacgggcggtatgtcctgagcagcatttacgccgtctgcgctctagctgcgttaacttgctttgtcatcaggctaacaaaaaattgcatgtcctggcgttactcatgcaccagatacactaactttcttctggacaccaagggcaaactctatcgttggcggtctcccgtcatcgtggagaaagggggtaaagttgaggtcgcaggccacctgatcgacctcaagagagttgtgcttgacggttccgcggcaacccctataaccaaagtttcagcggaacaatggggtcgtccttag

>CF-CF4-20260120-5

atgtcggggaaatgcttgaccgcgggctgttgctcgcaattgccttttttgtggtatatcgtgccgttctgttttgttgtgctcgtcaacgccaacaacagcagcagctcccatttacagttgatttataacctgacgatatgtgagctgaatggcacagattggctaaataagagttttgattgggcagtggagacctttgttatcttccctgtgttgactcatattgtctcctatggcgccctaaccaccagccatttccttgacacagttggcctgatcaccgtgtctaccgctgggtattgccacgggcggtatgtcctgagcagcatttacgccgtctgcgctctagctgcgttaacttgctttgtcatcaggctaacaaaaaattgcatgtcctggcgttactcatgcaccagatacactaactttcttctggacaccaagggcaaactctatcgttggcggtcccccgtcatcgtggagaaagggggtaaagttgaggtcgcaggccacctgatcgacctcaagagagttgtgcttgacggttccgcggcaacccctataaccaaagtttcagcggaacaatggggtcgtccttag

>CF-CF4-20260120-6

atgttggggaaatgcttgaccgcgggctgctgctcgcaattgccttttttatggtgtatcgtgccgttctgtttggttgcgctcgtcaacgccaccgacaacagcagctcccacttacaattgatttataacctaacgatatgtgagctgaatggcacagattggctgaaagaacacttttcttgggcggtggagactttcgttatttttcctgtgttaactcatgttgtctcttacggtgccctcaccactagcca-tttcttgacacggtcggtcttatcactgtgtccaccgccgggtattatcacggggggtatgtattgagtagcatctatgctgtctgcgccctagctgcgcttgtttgcttcgccattaggttggcaaagaattgcatgtcctggcgctactcttgcaccagatatactaattttcttctggacactaagggaaaactctatcgttggcggtcacccgtcatcatgaggaaggggggcaaggtggatgttgggggtcatttaatcgacctaaagagagttgtgcttgaaggttccgcggcaacccctgtaaccaaga-ttcagcggaacaatggggtcgtccatag

>CF-CF4-20260120-7

atgttggggaaatgcttgaccgcgggctgctgctcgcaattgccttttttatggtgtatcgtgccgttctgtttggttgcgctcgtcaacgccaccgacaacagcagctcccacttacaattgatttataacctaacgatatgtgagctgaatggcacagattggctgaaagaacacttttcttgggcggtggagactttcgttatttttcctgtgttaactcatgttgtctcttacggtgccctcaccactagccattttcttgacacggtcggtcttatcactgtgtccaccgccgggtattatcacgggcggtatgtattgagtagcatctatgctgtctgcgccctagctgcgcttgtttgcttcgccattaggttggcaaagaattgcatgtcctggcgctactcttgcaccagatatactaattttcttttggacactaagggaaaactctatcgttggcggtcacccgtcatcatagagaaggggggcaaggtggatgttgggggtcatttaatcgacctaaagagagttgtgcttgaaggttccgcggcaacccctgtaaccaagatttcagcggaacaatggggtcgtccatag

>CF-CF4-20260120-8

atgttggggaaatgcttgac--cgggctgctgctcgcaattgcc-tttttatggtgtatcgtgccgttctg-tttgttgcgctcgtcaacgccaccgacaacagcagctcccacttacaattgatttataacctaacgatatgtgagctgaatggcacagattggctgaaaggacacttttcttgggcggtggagactttcgttatttttcctgtgttaactcatgttgtctcttacggtgccctcaccactagccattttcttgacacggtcggtcttatcactgtgtccaccgccgggta-tatcacgggcggtatgtattgagtagcatctatgctgtctgcgccctagctgcgcttgtttgcttcgccattaggttggcaaagaattgcatgtcctggcgctactcttgcaccagatatactaattttcttctggacactaagggaaaactctatcgttggcggtcacccgtcatcatagagaaggggggcaaggtggatgtcgggggtcatttaatcgacctaaagagagttgtgcttgaaggttccgcggcaacccctgtaaccaagatttcagcggaacaatggggtcgtccatag

>CF-CF4-20260120-9

atgttggggaaatgcttgaccgcgggctgctgctcgcaattgccttttttatggtgtatcgtgccgttctgtttggttgcgctcgtcaacgccaccgacaacagcagctcccacttacaattgatttataacctaacgatatgtgagctgaatggcacagattggctgaaagaacacttttcttgggcggtggagactttcgttatttttcctgtgttaactcatgttgtctcttacggtgccctcaccactagccattttcttgacacggtcggtcttatcactgtgtccaccgccgggtattatcacgggcggtatgtattgagtagcatctatgctgtctgcgccctagctgcgcttgtttgcttcgccattaggttggcaaagaattgcatgtcctggcgctactcttgcaccagatatactaattttcttctggacactaagggaaaactctatcgttggcggtcacccgtcatcatagagaaggggggcaaggtggatgtcgggggtcatttaatcgacctaaagagagttgtgcttgaaggttccgcggcaacccctgtaaccaagatttcagcggaacaatggggtcgtccatag

>CF-CF5-20250512-72

ATGTTGGGGAAATGCTTGACCGCGGGCTGCTGCTCGCAATTGCCTTTTTTGTGGTGTATCGTGCCGTTCTGTTTAGTTGCGCTCGTCAACGCCAGCGACTACAGCAGCTCCCACTTACAATTGATTTATAACCTAACGATATGTGAGCTGAATGGCACAGATTGGCTGAAAGACCACTTTTCTTGGGCGGTGGAGACTTTCGTTATTTTTCCTGTGTTAACTCATATTGTCTCTTACGGTGCCCTCACCACTAGCCATTTTCTTGACACGGTCGGTCTTATCACTGTGTCCACCGCCGGGTATTATCACGGGCGGTATGTATTGAGTAGCATCTACGCTGTCTGCGCCCTAGCTGCGCTTGTTTGCTTCGCCATTAGGTTGGTAAAGAATTGCATGTCCTGGCGCTACTCTTGCACCAGATATACTAATTTTCTTCTGGACACTAAGGGAAAACTCTATCGTTGGCGGTCACCCGTCATCATAGAGAAGGGGGGCAAGGTGGATGTTGGTGGTCATTTAATCGACCTAAAGAGAGTTGTGCTTGAAGGTTCCGCGGCAACCCCTGTAACCAAGATTTCGGCGGAACAATGGGGTCGTCCATAG

>CF-5-20250807-66

ATGTTGGGGAAATGCTTGACCGCGGGTTATTGCTCACAATCGGTTTTTTTGTGGTGTATCGTGCCATTCTGTTTTGCTGTGCTCGTCAACGCCAGCGGCAACAGCAGCTCCCGCTTACAATTGATCTATAACCTGACGATATGTGAGCTGAATGGCACAGACTGGTTGAACCAAAGATTTGACTGGGCAGTGGAGACTTTCGTCATTTTTCCTGTCTTGACCCATATCGTCTCTTACGGCGCTCTTACCACTAGCCATTTTCTTGACACGGTCGGCCTAATCACCGTGTCCACTGCTGGGTATTATCATAAGCGGTATGTATTGAGTAGCATCTACGCTGTCTGTGCCCTAGCCGCGTTGGTTTGCTTCGCCATTAGGTTGGCAAAAAATTGTATGTCCTGGCGCTACTCATTCACCAGATATACTAATTTTCTTCTGGACTCTAAGGGCAAACTCTACCGTTGGCGGTCACCCGTCATCATAGAGAAAGAGGGCAAAGTTGATGTAAGGGGTCAGTTAATCGACCTCAAGAGAGTTGTGCTTGATGGTTCCGCGGCGACCCCTGTAACCAAGATTTCAGCGGAACAATGGGGTCGTCCATAG

>CF5-20251229-8

TGTTTTAGCCCTGTCTTTTTGCCATTCTGCTGGCAGTTTAAATGTTCAGGTATGTTGGGGAAATGCTTGACCGCGGGCTGCTGCTCGCAATTGCTTTTTTTGTGGTGTATCGTGCCGTTCTGTTTAGTTTCGCTCGTCAACGCCAGCAACAGCAGCAGCTCCCATTTACAGTTGATTTATAACCTGACGATATGTGAGCTGAATGGCACAGATTGGCTAAATCGCACTTTTGATTGGGCAGTGGAGACCTTCGTTATTTTTCCTGTGTTGACTCATATCGTCTCCTATGGCGCCCTAACCACCAGCCACTTCCTTGACACAGTCGGCCTGATCACCGTGTCTACTGCTGGATATTACCACGGGCGGTATGTCTTGAGCAGTATTTACGCCGTCTGCGCCTTAGCTGCGCTAATTTGCTTCGTCATCAGGCTAACAAAAAATTGCATGTCCTGGCGTTACTCATGCACCAGATACACTAATTTTCTTCTGGACACCAAGGGAAAACTCTATCGTTGGCGGTCTCCCGTCATCGTGGAAAAAGGGGGTAAAGTTGAGGTCGAAGGCCACCTCATCGACCTCAAGAAAGTTGTGCTTGATGGTTCCGCGGCGACCCCTATAACCAAAGTTACAGCGGAACAATGGGGTCGTCCTTAGACGACTTCTGCTATGACAGCACAGCCCCGCAAAAGGTGCTTTTGGG

>CF-CF6-20250530-116

ATGTTGGGGAAATGCTTGATCGCGGGTTATTGCTCGCAATTGCTTTTTTTGTGGTGTATCGTGCCATTCTGTTTTGTTGCACCCGTCAACGCCAACAACAGCAGCAGCTCCCATTTCCAGTTGATTTATAACTTAACGATATGTGAGCTGAATGGCACAGATTGGCTGACCAAAAATTTTTCTTGGCCAGTAGAGACTTTGGTCATCTTTCCGGTGTTAACTCATATTGTTTCTTACGGTGCCCTCACCACTAGTCATTTTCTTGACACGGCCGCCTTGATCACTGTGTCCCCCGCCGGGTATTTTCACGGGCGGTATGTATTGAGTAGCATCTATGCGGTCTGTGCCCTAGCTGCGTTGGTTTGCTTCGCCATTAGATTGGCAAAAAATTGCATGTCTTGGCGCTACTCCTGCACCAGATATACCAATTTTCTTCTGGACACTAAGGGCAAACTCTATCGTTGGCGGTCACCCGTCATCATAGAGAAGGGGGGCAAAGTGGACGTTGGAGGTCATTTAATCGACCTCAAGAAAGTTGTGCTTGAAGGTTCCGCGGCAACCCCTGTAACCAAAATTTCAGCGGAACTATGGGGTCGTCCATAG

>CF-CF6-20250820-90

ATGTCGGGGAAATGCTTGACCGCGGGCTGTTGCTCGCAATTGCCTTTTTTGTGGTATATCGTGCCGTTCTGTTTTGTTGTGCCCGTCAACGCCAACAACAGCAGCAGCTCCCATTTACAGTTGATTTATAACCTGACGATATGTGAGCTGAATGGCACAGATTGGCTAAATAAGAGTTTTGATTGGGCAGTGGAGACCTTTGTTATCTTCCCTGTGTTGACTCATATTGTCTCCTATGGCGCCCTAACCACCAGCCATTTCCTTGACACAGTTGGCCTGATCACCGTGTCTACCGCTGGGTATTGCCACGGGCGGTATGTCCTGAGCAGCATTTACGCCGTCTGCGCCCTAGCTGCGTTAACTTGCTTTGTCATCAGGCTAACAAAAAATTGCATGTCCTGGCGTTACTCATGCACCAGATACACCAACTTTCTTCTGGACACCAAGGGCAAACTCTATCGTTGGCGGTCTCCCGTCATCGTGGAGAAAGGGGGTAAAGTTGAGGTCGCAGGCCACCTGATCGACCTCAAGAGAGTTGTGCTTGACGGTTCCGCGGCAACCCCTATAACCAAAGTTTCAGCGGAACAATGGGGTCGTCTTTAG

>CF-CF6-20250820-91

ATGTCGGGGAAATGCTTGACCGCGGGCTGTTGCTCGCAATTGCCTTTTTTGTGGTATATCGTGCCGTTCTGTTTTGTTGTGCTCGTCAACGCCAACAACAGCAGCAGCTCCCATTTACAGTTGATTTATAACCTGACGATATGTGAGCTGAATGGCACAGATTGGCTAAATAAGAGTTTTGATTGGGCAGTGGAGACCTTTGTTATCTTCCCTGTGTTGACTCATATTGTCTCCTATGGCGCCCTAACCACCAGCCATTTCCTTGACACAGTTGGCCTGATCACCGTGTCTACCGCTGGGTATTGCCACGGGCGGTATGTCCTGAGCAGCATTTACGCCGTCTGCGCCCTAGCTGCGTTAACTTGCTTTGTCATCAGGCTAACAAAAAATTGCATGTCCTGGCGTTACTCATGCACCAGATACACTAACTTTCTTCTGGACACCAAGGGCAAACTCTATCGTTGGCGGTCTCCCGTCATCGTGGAGAAAGGGGGTAAAGTTGAGGTCGCAGGCCACCTGATCGACCTCAAGAGAGTTGTGCTTGACGGTTCCGCGGCAACCCCTATAACCAAAGTTTCAGCGGAACAATGGGGTCGTCCTTAG

>CF6-20251110-4

TGTTTTAGCCCGTCTTTTTGCCATTCTGCTGGCAATTTGAATGTTCAGGTATGTCGGGGAAATGCTTGACCGCGGGCTGTTGCTCGCAATTGCCTTTTTTGTGGTATATCGTGCCGTTCTGTTTTGTTGTGCTCGTCAACGCCAACAACAGCAGCAGCTCCCATTTACAGTTGATTTATAACCTGACGATATGTGAGCTGAATGGCACAGATTGGCTAAATAAGAGTTTTGATTGGGCAGTGGAGACCTTTGTTATCTTCCCTGTGTTGACTCATATTGTCTCCTATGGCGCCCTAACCACCAGCCATTTCCTTGACACAGTTGGCCTGATCACCGTGTCTACCGCTGGGTATTGCCACGGGCGGTATGTCCTGAGCAGCATTTACGCCGTCTGCGCCCTAGCTGCGTTAACTTGCTTTGTCATCAGGCTAACAAAAAATTGCATGTCCTGGCGTTACTCATGCACCAGATACACTAACTTTCTTCTGGACACCAAGGGCAGACTCTATCGTTGGCGGTCTCCCGTCATCGTGGAGAAAGGGGGTAAAGTTGAGGTCGCAGGCCACCTGATCGACCTCAAGAGAGTTGTGCTTGACGGTTCCGCGGCAACCCCTATAACCAAAGTTTCAGCGGAGCAATGGGGTCGTCCTTAGACGACTTCTGCTATGACAGCAAAGCCCCGCAAAAGGTGCTTTTGGGC

>CF-CF6-20260120-1

atgtcggggaaatgcttgaccgcgggctgttgctcgcaattgccttttttgtggtatatcgtgccgttctgttttgttgtgctcgtcaacgccaacaacagcagcagctcccatttacagttgatttataatctgacgatatgtgagctgaatggcacagattggctaaataagagttttgattgggcagtggagacctttgttatcttccctgtgttgactcatattgtctcctatggcgccctaaccaccagccatttccttgacacagttggcctgatcaccgtgtctaccgctgggtattgccacgggcggtatgtcctgagcagcatttacgccgtctgcgctctagctgcgttaacttgctttgtcatcaggctaacaaaaaattgcatgtcctggcgttactcatgcaccagatacactaactttcttctggacaccaagggcaaactctatcgttggcggtcccccgtcatcgtggagaaagggggtaaagttgaggtcgcaggccacctgatcgacctcaagagagttgtgcttgacggttccgcggcaacccctataaccaaagtttcagcggaacaatggggtcgtccttag

>CF-CF6-20260120-1

atgtcggggaaatgcttgaccgcgggctgttgctcgcaattgctttttttgtggtatatcgtgccgttctgttttgttgtgctcgtcaacgccaacaacagcagcagctcccatttacagttgatttataacctgacgatatgtgagctgaatggcacagattggctaaataagagttttgattgggcagtggagacctttgttatcttccctgtgttgactcatattgtctcctatggcgccctaaccaccagccacttccttgacacagttggcctgatcaccgtgtctgccgctgggtattgccacgggcggtatgtcctgagcagcatttacgccgtctgcgccctagctgcgttaacttgctttgtcatcaggctaacaaaaaattgcatgtcctggcgttactcatgcaccagatacactaactttcttctggacaccaagggcaaactctatcgttggcggtctcccgtcatcgtggagaaagggggtaaagttgaggtcgcaggccacctgatcgacctcaagagagttgtgcttgacggttccgcggcaacccctataaccaaagtttcagcggaacaatggggtcgtccttag

>CF-CF6-20260124-1

atgtcggggaaatgcttgaccgcgggctgttgctcgcaattgccttttttgtggtatatcgtgccgttctgttttgttgtgctcgtcaacgccaacaacagcagcagctcccatttacagttgatttataacctgacgatatgtgagctgaatggcacagattggctaaataagagttttgattgggcagtggagacctttgttatcttccctgtgttgactcatattgtctcctatggcgccctaaccaccagccatttccttgacacagttggcctgatcaccgtgtctaccgctgggtattgccacgggcggtatgtcctgagcagcatttacgccgtctgcgctctagctgcgttaacttgctttgtcatcaggctaacaaaaaattgcatgtcctggcgttactcatgcaccagatacactaactttcttctggacaccaagggcaaactctatcgttggcggtcccccgtcatcgtggagaaagggggtaaagttgaggtcgcaggccacctgatcgacctcaagagagttgtgcttgacggttccgcggcaacccctataaccaaagtttcagcggaacaatggggtcgtccttag

>CF-CF6-20260124-2

atgttggggaaatgcttgaccgcgggctgctgctcgcaattgctttttttgtggtgtatcgtgccgttctgtttagttgcgctcgtcaacgccaacgacaacagcagctcccacttacaattgatttatgacctaacgatatgtgagctgaatggcacagattggctgaaagaccacttttcttgggcggtggagactttcgttatctttcctgtgttaactcatattgtctcttacggtgccctcaccaccagccatcttcttgacacggtcggtcttatcactgtgtccaccgccgggtattatcacgggcggtatgtattgagtagcatctacgctgtctgcgccctagctgcgcttgcttgcttcgccattaggttggtaaagaattgcatgtcctggcgctactcttgcaccagatatacaaattttcttctggacactaagggaaaactctatcgttggcggtcacccgtcatcatagagaaggggggcaaggtggatgttggtggtcatttgatcgacctaaagagagttgtgcttgaaggttccgcggcaacccctgtaaccaagatttcagcggaacaatggggtcgtccatag

>CF-CF6-20260124-3

atgttggggaaatgcttgaccgcgggctgctgctcgcaattgctttttttgtggtgtatcgtgccgttctgtttagttgcgctcgtcaacgccaacgacaacagcagctcccacttacaattgatttataacctaacgatatgtgagctgaatggcacagattggctggaaaaccacttttcttgggcggtggagactttcgttatctttcctgtgttaactcatattgtctcttacggtgccctcaccaccagccatcttcttgacacggtcggtcttatcactgtgtccaccgccgggtattatcacgggcggtatgtattgagtagcatctacgctgtctgcgccctagctgcgcttgcttgcttcgccattaggttggtaaagaattgcatgtcctggcgctactcttgcaccagatata-caaatttcttctggacactaagggaaaactctatcgttggcggtcacccgtcatcatagagaaggggggcaaggtggatgttggtggtcatttaatcgacctaaagagagttgtgcttgaaggttccgcggcaacccctgtaaccaagatttcagcggaacaatggggtcgtccatag

>CF-CF6-20260124-4

atgtcggggaaatgcttgaccgcgggctgttgctcgcaattgccttttttgtggtatatcgtgccgttctgttttgttgtgctcgtcaacgccaacaacagcagcagctcccatttacagttgatttataacctgacgatatgtgagctgaatggcacagattggctaaataagagttttgattgggcagtggagacctttgttatcttccctgtgttgactcatattgtctcctatggcgccctaaccaccagccacttccttgacacagttggcctgatcaccgtgtctgccgctgggtattgccacgggcggtatgtcctgagcagcatttacgccgtctgcgccctagctgcgttaacttgctttgtcatcaggctaacaaaaaattgcatgtcctggcgttactcatgcaccagatacactaactttcttctggacaccaagggcaaactctatcgttggcggtctcccgtcatcgtggagaaagggggtaaagttgaggtcgcaggccacctgatcgacctcaagagagttgtgcttgacggttccgcggcaacccctataaccaaagtttcagcggaacaatggggtcgtccttag

>CF-CF6-20260129-1

atgttggggaaatgcttgaccgcgggctgctgctcgcaattgctttttttgtggtgtatcgtgccgttctgtttagttgcgctcgtcaacgccaacgacaacagcagctcccacttacaattgatttataacctaacgatatgtgagctgaatggcacagattggctgaaagaccacttttcttgggcggtggagactttcgttatctttcctgtgttaactcatattgtctcttacggtgccctcaccaccagccattttcttgacacggtcggtcttatcactgtgtccaccgccgggtattatcacgggcggtatgtattgagtagcatctacgctgtctgcgccctagctgcgcttgtttgcttcgccattaggttggtaaagaattgcatgtcctggcgctactcttgcaccagatatactaattttcttctggacactaagggaaaactctatcgttggcggtcacccgtcatcatagagaaggggggcagggtggatgttggtggtcatttaatcgacctaaagagagttgtgcttgaaggttccgcggcaacccctgtaaccaagatttcagcggaacaatggggtcgtccatag

>CF-CF6-20260129-2

atgttggggaaatgcttgaccgcgggctgctgctcgcaattgctttttttgtggtgtatcgtgccgttctgtttagttgcgctcgtcaacgccaacgacaacagcagctcccacttacaattgatttataacctaacgatatgtgagctgaatggcacagattggctgaaagaccacttttcttgggcggtggagactttcgttatctttcctgtgttaactcatattgtctcttacggtgccctcaccaccagccattttcttgacacggtcggtcttatcactgtgtccaccgccgggtattatcacgggcggtatgtattgagtagcatctacgctgtctgcgccctagctgcgcttgtttgcttcgccattaggttggtaaagaattgcatgtcctggcgctactcttgcaccagatatactaattttcttctggacactaagggaaaactctatcgttggcggtcacccgtcatcatagagaaggggggcagggtgga-gttggtggtcatttaatcgacctaaagagagttgtgcttgaaggttccgcggcaacccctgtaaccaagatttcagcggaacaatggggtcgtccatag

>CF-CF7-20250730-21

ATGTTGGTGAAATGCTTGACCGCGGGCTGCTGCTCGCAATTGCTTTTTTTGTGGTGTATCGTGCCGTTCTGTTTAGTTTCGCTCGTCAACGCCAGCGACAGCAGCAGCTCCCATTTACAGTTGATTTATAACCTGACGATATGTGAGCTGAATGGCACAGATTGGCTAAATCACACTTTTGATTGGGCAGTGGAGACCTTCGTTATTTTTCCTGTGTTGACTCATATCGTCTCCTATGGCGCCCTGACCACCAGCCACTTCCTTGACACAGTCGGCCTGATCACCGTGTCCACTGCTGGATATTACCACGGGCGGTATGTTTTGAGCAGTATTTACGCCGTCTGCGCCTTGGCTGCGCTAATTTGCTTCATCATCAGGCTAACAAAAAATTGCATGTCCTGGCGTTACTCGTGCACCAGATACACTAATTTTCTTCTGGACACCAAGGGAAAACTCTATCGTTGGCGGTCTCCCGTCATCGTGGAAAAAGGGGGTAAAGTTGAGGTCGAAGGCCACCTCATTGACCTCAAGAAAGTTGTGCTTGATGGTTCCGCGGCGACCCCTATAACCAAAGTTACAGCGGAACAATGGGGTCGTCCTTAG

>CF-CF7-20250730-22

ATGTTGGGGAAGTGCTTGACCGCGTGCTGTTGCTCGCGATTGCTTTTTTTGTGGTGTATCGTGCCGTTCTATCTTGCTGTGCTCGTCAACGCCAGCAACAACAACAGCTCTCATATTCAGTTGATTTATAACTTGACGCTATGTGAGCTGAATGGCACAGATTGGCTGGCACAAAATTTTGACTGGGCAGTGGAGACTTTTGTCATCTTCCCCGTGTTGACTCACATTGTTTCCTATGGGGCACTCACCACCAGCCATTTCCTTGACACAGTTGGTCTGGCCACTGTGTCCACCGCCGGATATTATCACGGGCGGTATGTCTTGAGTAGCATTTACGCAGTCTGTGCTCTGGCTGCGCTGATTTGCTTTGTCATTAGGCTTGCGAAGAACTGCATGTCCTGGCGCTACTCTTGTACCAGATATACCAACTTCCTTCTGGACACTAAGGGCAGACTCTATCGTTGGCGGTCGCCCGTCATTGTGGAGAAAAGGGGTAAGGTTGAGGTCGAAGGTCACCTGATCGACCTCAAGAGAGTTGTGCTTGATGGTTCCGCGGCAACCCCTTTAACCAGAGTTTCAGCGGAACTATGGGGTCGTCTCTAG

>CF-7-20250730

ATGTTGGGGAAATGCTTGACCGCGGGCTGCTGCTCGCAATTCCCTTTTTTGTGGTGTATCGTGCCGTTCTGTTTAGTTGCGCTCGTCAACGCCAACGACAACAGCAGCTCCCACTTACAATCGATTTATAACCTAACGATATGTGAGCTGAATGGCACAGATTGGCTGAAAGAACACTTTTCTTGGGCGGTGGAGACTTTCGTTATTTTTCCTGTGTTAACTCATGTTGTCTCTTACGGTGCCCTCACCACTAGCCATTTTCTTGATACGGTCGGTCTTATCACTGTGTCCACCGCCGGGTATTATCACGGGCGGTATGTATTGAGTAGCATCTACGCTGTCTGCGCCCTAGCTGCGCTTGTTTGCTTCGCCATTAGGTTGGTAAAGAATTGCATGTCCTGGCGCTACTCTTGCACCAGATATACTAATTTTCTTCTGGACACTAAGGGAAAACTCTATCGTTGGCGGTCGCCCGTCATCATAGAGAAGGGGGGCAAGGTGGATGTTGGGGGTCATTTAATCGACCTAAAGAGAGTTGTGCTTGAAGGTTCCGCGGCAACCCCTGTAACCAAGATTTCAGCGGAACAATGGGGTCGTCCATAG

>CF-CF7-20260120-1

atgttggggaaatgcttgaccgcgggctgctgctcgcaattgccttttttgtggtgtatcgtgccgttctgtttggttgcgctcgtcaacgccaacgacaacagcagctcccacttacaattgatttataacctaacgatatgtgagctgaatggcacagattggctggaaaaacacttttcttgggcggtggagactttcgttatttttcctgtgttaactcatgttgtctcttacggtgccctcaccactagccattttcttgacacggtcggtcttatcactgtgtccaccgccgggtattatcacgggcggtatgtattgagtagcatctacgctgtctgcgccctagctgcgcttgtttgcttcgccattaggttggcaaagaattgcatgtcctggcgctactcttgcaccagatatactaattttcttctggacactaagggaaaactctatcgttggcggtcacccgtcatcatagagaaggggggcaaggtggatgttgggggtcatttaatcgacctaaagagagttgtgcttgaaggttccgcggcgacccctataaccaaagtttcagcggaacaatggggtcgtccttag

>CF-CF7-20260120-2

atgttggggagatgcttgatcgcgggctgttgctcgcaattgccttttttgtggtatatcgtgccgttctgctttgttgtgctcgtcaacgccaacaacagcagcagctcccatttacagttgatttataacctgacgatatgtgagctgaatggcacagattggctaaataaaagttttgattgggcagtggagacctttgttattttccctgtgttgactcatattgtctcctatggcgccctaaccaccagccatttccttgacacagttggcctgatcaccgtgtctaccgctggatattatcacgggcggtatgtcttgagcagcatttacgccgcctgcgccctagctgcgttaacttgctttgtcatcaggctaacaaaaaattgcatgtcctggcgttactcatgcaccagatacactaactttcttctggacaccaagggcaaactc-atcgttggcggtctcccgtcatcgtggagaaagggggtaaagttgaggtcgaaggccacctgatcgacctcaagaaagttgtgcttgacggttccgcggcgacccctataaccaaagtttcagcggaacaatggggtcgtccttag

>CF-CF7-20260120-13

atgttggggaaatgcttgaccgcgggctgctgctcgcaattgccttttttgtggtgtatcgtgccgttctgtttagttgcgctcgtcaacgccaacaacgacagcagctcccacttacaattgatttataacctaacgatatgtgagctgaa-ggcacagattggctgaaagaacacttttcttgggcagtggagacctttgttattttccctgtgttgactcatattgtctcctatggcgccctaaccaccagccatttccttgacacagttggcctgatcaccgtgtctaccgctggatattatcacgggcggtatgtcctgagcagcatttacgccgcctgcgccctagctgcgttaacttgctttgtcatcaggctaacaaaaaattgcatgtcctggcgttactcatgcaccagatacactaactttcttctggacaccaagggcaaactctatcgttggcggtctcccgtcatcgtggagaaagggggtaaagttgaggtcgaaggccacctgatcgacctcaagagagttgtgcttgacggttccgcggcgacccctataaccaaagtttcagcggaacaatggggtcgtccttag

>CF-CF7-20260120-14

atgttggggaaatgcttgaccgcgggctgctgctcgcaattgctttttttgtggtgtatcgtgccgttctgtttagtttcgctcgtcaacgccagcaacagcagcagctcccatttacagttgatttataacctgacgatatgtgagctgaatggcacagattggctaaatcgcacttttgattgggcagtggagaccttcgttatttttcctgtgttgactcatatcgtctcctatggcgccctaaccaccagccacttccttgacacagtcggcctgatcaccgtgtctactgctggatattaccacgggcggtatgtcttgagcagtatttacgccgtctgcgccttagctgcgctgatttgcttcgtcatcaggctaacaaaaaattgcatgtcctggcgttactcctgcaccagatacactaattttcttctggacaccaaaggaaaactctatcgttggcggtctcccgtcatcgtggaaaaagggggtaaagttgaggtcgaaggccacctcatcgacctcaagaaagttgtgcttgatggttccgcggcgacccctataaccaaagttacagcggaacaatggggtcgtccttag

>CF-CF7-20260120-15

atgttggggaaatgcttgaccgcgggctgctgctcgcaattgctttttttgtggtgtatcgtgccgttctgtttagttgcgctcgtcaacgccagcaacagcagcagctcccatttacagttgatttataacctgacgatatgtgagctgaatggcacagattggctaaatcgcacttttgattgggcggtggagactttcgttatttttcctgtgttaactcatgttgtctcttacggcgccctcaccactagccattttcttgacacggtcggtcttatcactgtgtccaccgccgggtattgtcacgggcggtatgtattgagtagcatctacgctgtctgcgccctagctgcgcttgtttgcttcgccattaggttggtaaagaattgcatgtcctggcgctactcttgcaccagatatactaattttcttctggacactaagggaaaactctatcgttggcggtcacccgtcatcatagagaaggggggcaaggtggacgttgggggtcatttaatcgacctaaagagagttgtgcttgaaggttccgcggcaacccctgtaaccaagatttcagcggaacaatggggccgtccatag

>CF-CF7-20260120-18

atgttggggaaatgcttgaccgcgggctgctgctcgcaattgctttttttgtggtgtatcgtgccgttctgtttagttgcgctcgtcaacgccaacaacgacagcagctcccacttacaattgatttataacctacagatatgtgagctgaatggcacagattggctgaaagaacacttttcttgggcggtggagactttcgttatttttcctgtgttaactcatgttgtctcttacggcgccctcaccactagccattttcttgacacggtcggtcttatcactgtgtccaccgccgggtattgtcacgggcggtatgtattgagtagcatctacgctgtctgcgccctagctgcgcttgtttgcttcgccattaggttggtaaagaattgcatgtcctggcgctactcttgcaccagatattattggcgtgtaggtgatagagaacgccaacaacaccttttgcggggctttgctgtcataacagaagtcgtc-----------taaggacgaccccattgttccgctgaaactttggttataggggtcgccgcggaaccgtcaagcacaactctcttgaggtcgatcaggtggccttcga

>CF-CF7-20260120-19

atgttggggagatgcttgatcgcgggctgttgctcgcaattgccttttttgtggtatatcgtgccgttctgctttgttgtgctcgtcaacgccaacaacagcagcagctcccatttacagttgatttataacctgacgatatgtgagctgaatggcacagattggctaaataaaagttttgattgggcagtggagacctttgttattttccctgtgttgactcatattgtctcctatggcgccctaaccaccagccatttccttgacacagttggcctgatcaccgtgtctaccgctggatattatcacgggcggtatgtcctgagcagcatttacgccgcctgcgccctagctgcgttaacttgctttgtcatcaggctaacaaaaaattgcatgtcctggcgttactcatgcaccagatacactaattttcttctggacactaagggaaaactctatcgttggcggtcacccgtcatcatagagaaggggggcaaggtggacgttgggggtcatttaatcgacctaaagagagttgtgcttgaaggttccgcggcaacccctgtaaccaagatttcagcggaacaatggggccgtccatag

>CF-CF7-20260120-30

ATGTTGGGGAAATGCTTGACCGCGGGCTGCTGCTCGCAATTGCCTTTTTTGTGGTGTATAGTGCCGTTCTGTTTAGTTGCGCTCGTCAACGCCAACAACAACAGCAGCTCCCACTTACAATTGATTTATAACCTAACGATATGTGAGCTGAATGGCACAGATTGGCTGAAAGAACACTTTTCTTGGGCGGTGGAGACTTTCGTTATTTTTCCTGTGTTAACTCATGTTGTCTCTTACGGCGCCCTCACCACTAGCCATTTTCTTGACACGGTCGGTCTTATCACTGTGTCCACCGCCGGGTATTGTCACGGGCGGTATGTATTCGGTGGCCTACA-------TGCGCCCTAGCTGCGCTTGTTTGCTTCGCCATTAGGTTGGTAAAGAATTGCATGTCCTGGCGCTACTCTTGCACCAGATATACTAATTTTCTTCTGGACACTAAGGGAAAACTCTATCGTTGGCGGTCACCCGTCATCATAGAGAAGGGGGGCAAGGTGGACGTTGGGGGTCATTTAATCGACCTAAAGAGAGTTGTGCTTGAAGGTTCCGCGGCAACCCCTGTAACCAAGATTTCAGCGGAACAATGGGGCCGTCCATAG

>CF-CF7-20260120-31

ATGTTGGGGAGATGCTTGATCGCGGGCTGTTGCTCGCAATTGCCTTTTTTGTGGTATATCGTGCCGTTCTGCTTTGTTGTGCTCGTCAACGCCAACAACAGCAGCAGCTCCCATTTACAGTTGATTTATAACCTGACGATATGTGAGCTGAATGGCACAGATTGGCTAAATAAAAGTTTTGATTGGGCAGTGGAGACCTTTGTCATTTTCCCTGTGTTGACTCATATTGTCTCCTATGGCGCCCTAACCACCAGCCATTTCCTTGATACAGTTGGCCTGATCACCGTGTCTACCGCTGGATATTATCACGGGCGGTATGTCCTGAGCAGCATTTACGCCGCCTGCGCCCTAGCTGCGTTAACTTGCTTTGTCATCAGGCTAACAAAAAATTGCATGTCCTAGTATCATTC----ACCAGATACACTAACTTTCTTCTGGACACCAAGGGCAAACTCTATCGTTGGCGGTCTCCCGTCATCGTGGAGAAAGGGGGTAAAGTTGAGGTCGAAGGCCACCTGATCGACCTCAAGAGAGTTGTGCTTGACGGTTCCGCGGCGACCCCTATAACCAAAGTTTCAGCGGAACAATGGGGTCGTCCTTAG

>CF-CF7-20250604-128

ATGTTGGGGAAATGCTTGACCGCGGGCTGCTGCTCGCAATTGCTTTTTTTGTGGTGTATCGTGCCGTTCTGTTTAGTTTCGCTCGTCAACGCCAACAACAACAGCAGCTCCCATTTACAGTTGATTTATAACCTGACGATATGTGAGCTGAATGGCACAGATTGGCTAAATCACACTTTTGATTGGGCGGTGGAGACCTTCGTTATTTTTCCTGTGTTGACCCATATCGTCTCCTATGGCGCCCTAACCACCAGCCATTTCCTTGACACAGTCGGCCTGATCACCGTGTCTATCGCTGGATATTACCACGGGCGGTATGTCTTGAGCAGCATTTACGCCGTCTGCGCCCTAGCTGCGCTAATTTGCTTCGTCATCAGGCTAACAAAAAATTGCATGTCCTGGCGTTACTCATGCACCAGATACACTAACTTTCTTCTGGACACCAAGGGAAAACTCTATCGTTGGCGGTCTCCCGTCATCGTGGAAAAAGGGGGTAAAGTTGAGGTCGAAGGCCACCTCATCGACCTCAAGAAAGTTGTGCTTGACGGCTCCGCGGCGACCCCTATAACCAAAGTTACAGCGGAACAATGGGGTCGTCCTTAG

>CF-CF8-20250614-6

ATGTTGGAGAAATGCTTGACCGCGGGCTGTTGCTCGCAATTGCTTTCTTTGTGGTGTATCGTGCCGTTCTGTTTTGCTGTGCTCGCCAACGCCAGCAACGACAACAGCTCCCATCTACAGCTGATTTACAACTTGACGCTATGTGAGCTGAATGGCACAGATTGGCTAGCTAACAAATTTGATTGGGCAGTGGAGAGTTTTGTCATCTTTCCCGTTTTGACTCACATTGTCTCCTATGGTGCCCTCACTACCAGCCATTTCCTTGACACAGTCGCTTTAGTCACTGTGTCTACCGCCGGGTTTGTTCACGGGCGGTATGTCCTAAGTAGCATCTACGCGGTCTGTGCCCTGGCTGCGTTGACTTGCTTCGTCATTAGGTTTGCAAAGAATTGCATGTCCTGGCGCTACGCGTGTACCAGATATACCAACTTTCTTCTGGACACTAAGGGCAGACTCTATCGTTGGCGGTCGCCTGTCATCATAGAGAAAAGGGGCAAAGTTGAGGTCGAAGGTCATCTGATCGACCTCAAAAGAGTTGTGCTTGATGGTTCCGTGGCAACCCCTATAACCAGAGTTTCAGCGGAACAATGGGGTCGTCCTTAG

>CF8-20251210-20

TGTTTTAGCCCGTCTTTTTGCCATTCTGCTGGCAATTTGAATGTTCAGGTATGTTGGGGAAATGCTTGACCGCGGGCTGCTGCTCGCAATTGCTTTTTTTGTGGTGTATCGTGCCGTTCTGTTTAGTTGCGCTCGTCAACGCCAACGACAACAGCAGCTCCCACTTACAGTTGATTTATAACCTAACAATATGTGAGCTGAATGGCACAGATTGGCTGAAAGAACACTTTTCTTGGGCGGTGGAGACTTTCGTTATTTTCCCTGTGTTAACTCATGTTGTCTCTTACGGTGCCCTCACCACTAGCCATTTTCTTGACACGGTCGGTCTTATCACTGTGTCCACCGCCGGGTATTATCACGGGCGGTATGTACTGAGTAGTATCTACGCTGTCTGCGCCCTAGCTGCGCTTGTTTGCTTCGCCATTAGGTTGGTAAAGAATTGCATGTCCTGGCGCTACTCTTGCACCAGATATACTAATTTTCTTCTGGACACTAAGGGAAAACTCTATCGTTGGCGGTCACCCGTCATCATAGAGAAGGGGGGCAAGGTGGATGTCGGGGGTCATTTAATCGACCTAAAGAGAGTTGTGCTTGAAGGTTCCGCGGCAACCCCTGTAACCAAGATTTCAGCGGAACAATGGGGTTGTCCATAGACGACTTCTGCAATGACAGCACGGCTGTACAAAAGGTGCTTTGGG

>CF-CF8-20260129-1

atgttggggaaatgcttgaccgcgggctgctgctcgcaattgccttttttgtggtgtatcgtgccgttctgtttagttgcgctcgtcaacgccaacaacgacagcagctcccacttacaattgatttataacctaacgatatgtgagctgaatggcacagattggctgaaagaacacttttcttgggcggtggagactttcgttatttttcctgtgttaactcatgttgtctcttacggcgccctcaccactagccattttcttgacacggtcggtcttatcactgtgtccaccgccgggtattgtcacgggcggtatgtattgagtagcatctacgctgtctgcgccctagctgcgcttgtttgcttcgccattaggttggtaaagaattgcatgtcctggcgctactcttgcaccagatatactaattttcttctggacactaagggaaaactctatcgttggcggtcacccgtcatcatagagaaggggggcaaggtggacgttgggggtcatttaatcgacctaaagagagttgtgcttgaaggttccgcggcaacccctgtaaccaagatttcagcggaacaatggggccgtccatag

>CF-CF8-20260129-3

atgttggggagatgcttgatcgcgggctgttgctcgcaattgccttttttgtggtatatcgtgccgttctgctttgttgtgctcgtcaacgccaacaacagcagcagctcccatttacagttgatttataacctgacgatatgtgagctgaatggcacagattggctaaataaaagttttgattgggcagtggagacctt--gttttttccctgtgttgactcatattgtctcctatggcgccctaaccaccagccatttccttgacacagttggcctgatcaccgtgtctaccgctggatattatcacgggcggtatgtcctgagcagcatttacgccgcctgcgccctagctgcgttaacttgctttgtcatcaggctaac-aaaaattgcatgtcctggcgttactcatgcaccagatacactaactttcttctggacaccaagggcaaactctatcgttggcggtctcccgtcatcgtggagaaagggggtaaagttgaggtcgaaggccacctgatcgacctcaagagagttgtgcttgacggttccgcggcgacccctataaccaaagtttcagcggaacaatggggtcgtccttag

>CF-CF9-20250528-113

ATGTTGGGGAAATGCTTGACCGCGGGCTGCTGCTCGCAATTGCCTTTTTTGTGGTGTATCGTGCCGTTCTGTTTAGTTGCGCTCGTCAACGCCAACGAAAACAGCAGCTCCCACTTACAATTGATTTATAACCTAACGATATGTGAGCTGAATGGCACAGATTGGCTGAATGGACACTTTTCTTGGGCGGTGGAGACTTTCGTTATTTTTCCTGTGTTAACTCATATTGTCTCTTACGGTGCCCTCACCACTAGCCATTTTCTTGACACGGTCGGTCTTATCACTGTGTCCACCGCCGGGTATTGTCACGGGCGGTATGTATTGAGTAGCATCTACGCTGTCTGCGCCCTAGCTGCGCTTGGTTGCTTCGCCATTAGGTTGGTAAAAAATTGCATGTCCTGGCGCTACTCTTGCACCAGATATACTAATTTTCTTCTGGACACTAAGGGAAAACTCTATCGTTGGCGGTCACCCGTCATCATAGAGAAGGGGGGCAAGGTGGATGTTGGTGGTCATTTAATCGACCTAAAGAGAGTTGTGCTTGAAGGTTCCGCGGCAACCCCTGTAACCAAGATTTCAGCGGAACAATGGGGTCGTCCATAG

>CF-CF9-20250811-73

ATGTTGGGGAAATGCTTGACCGCGGGTTATTGCTCACAATCGCTTTTTTTGTGGTGTATCGTGCCATTCTGTTT

TGCTGTGCTCGTCAACGCCAGCGACAACAACAGCTCCCACTTACAGTTGATCTATAACCTGACGATATGTGAGC

TGAATGGCACAGACTGGTTGAAACAAAAATTTGACTGGGCAGTAGAGACTTTCGTCATTTTTCCTGTCTTGACC

CATATTGTCTCTTACGGCGCCCTTACCACTAGCCATTTTCTTGACACGGTCGGCCTAATCACCGTATCCGCCGC

TGGGTATTATCATAAGCGGTATGTGTTGAGTAGCATCTACGCTGTCTGTGCCCTGGCCGCGTTGGTTTGCTTCG

CCATTAGGTTGGCAAAAAATTGCATGTCCTGGCGCTACTCATTCACCAGATATACTAATTTTCTTCTGGACTCT

AAGGGCAAACTCTACCGTTGGCGGTCACCCGTCATCATAGAGAAAGAGGGCAAAGTTGATGTGGGGGGTCAGTT

AATCGAACTCAAGAGAGTTGTGCTTGATGGTTCCGCGGCGACCCCTGTAACCAAGATTTCAGCGGAACAATGGG

GTCGTCCATAG

>CF-CF9-20260129-1

agtttgaggacatgctaaaggtccagcccctgattgtctactcggatgatcttgtgctgtatgccgaatccccctccatgccaaattaccactggtgggtcgagcatcttaatttgatgctggggtttcagctttctgggctgtaggataattaacgggcgtcagctggtcccaaaccgtgacaggattctcgcggctcttgcctatcacatgaaggcgaataatgtttctgagtattacgcctccgcggccactggcttcttcaacaggttcaggacggtgatgttatctaccggcgggtggtgtccctgaaagcgcatcgtggattctctccgcgccatttgtgcttcacctgatttgacacagttcgccaaactcccggcagaacttcgcgtgcatcggtgccggctacatggtgggtccttcagttttttgttaggggcgaggctcaaatgcttccagagacggtcttcagcaccggtcgaattgagggacccagtccaaatgctggaaaatgttgttggacttcaaggaagtccgactgatggtctggcggccaactttttgtggatgctttcac-gagttcttggtgtctattgttg

>CF-CF9-20260129-7

atgttggggaaatgcttgaccgccggctgctgctcgcaattgccttttttgtggt-tatcgtgccgttctgtttagttgcgctcgtcaacgccaacgacaacagcagctcccacttacaattgatttataacctaacgatatgtgagctgaatggcacagattggctgaa-agacactttctctgggcggtggagactttcgttatttttcctgtgttaactcatattgtctcttacggtgccctcaccactagcca-tttcttgacacggtcggtcttatcactgtgtccaccgccgggtattatcacgggcggtatgtattgagtagcatctacgctgtctgcgccctagctgcgcttatttgcttcgccattaggttggtaaagaattgcatgtcctggcgctactcttgcaccagatatatcaattttcttctggacactaagggaaaactctatcgttggcggtcacccgtcatcatagagaaggggggcaaggtggatgttggtggtcatttaatcgacctaaagagagttgtgcttgaaggttccgcggcaacccctgtaaccaagatttcagcggaacaatggggtcgtccatag

>CF-CF10-20250821-96

ATGTTGGGGAAATGCTTGACCGCGGGCTGCTGCTCGCAATTGCCTTTTTTGTGGTGTATCGTGCCGTTCTGTTTAGTTGCGCTCGTCAACGCCAACGAAAACAGCAGCTCCCACTTACAATTGATTTATAACCTAACGATATGTGAGCTGAATGGCACAGATTGGCTGAATGAACACTTTTCTTGGGCGGTGGAGACTTTCGTCATTTTTCCCGTGTTGACTCATATTGTCTCTTACGGTGCCCTCACCACTAGCCATTTTCTTGACACGGTCGGTCTTATCACTGTGTCCACCGCCGGGTATTACCACGGGCGGTATGTATTGAGTAGCATCTACGCTGTCTGCGCCCTAGCTGCGCTTGTTTGCTTCGCCATTAGGTTGGTAAAAAATTGCATGTCCTGGCGCTACTCTTGCACCAGATATACTAATTTTCTTCTGGACACTAAGGGAAAACTCTATCGTTGGCGGTCACCCGTCATCATAGAGAAGGGGGGCAAGGTGGATGTTGGTGGTCATTTAATCGACCTAAAGAGAGTTGTGCTTGAAGGTTCCGCGGCAACCCCTGTAACCAAAATTTCAGCGGAACAATGGGGTCGTCCATAG

>CF10-20251110-6

CCCCGCTTTTTGCCATTCTGCTGGCAATTTGAATGTTCAGGTATGTTGGGGAAATGCTTGACCGCGGGCTGCTGCTCGCAATTGCTTTTTTTGTGGTGTATCGTGCCGTTCTGTTTAGTTGCGCTCGTCAACGCCAACGAAAACAGCAGCTCCCACTTACAATTGATTTATAACCTAACGATATGTGAGCTGAATGGCACAGATTGGCTGAATGAACACTTTTCTTGGGCGGTGGAGACTTTCGTTATTTTTCCTGTGTTAACTCATATTGTCTCTTACGGTGCCCTCACCACTAGCCATTTTCTTGACACGGTCGGTCTTATCACTGTGTCCACCGCCGGGTATTATCACGGGCGGTATGTATTGAGTAGCATCTACGCTGTCTGCGCCTTAGCTGCGCTTGGTTGCTTCACCATTAGGTTAGTAAAAAATTGCATGTCCTGGCGCTACTCTTGCACCAGATATACTAATTTTCTTCTGGACACTAAGGGAAAACTCTATCGTTGGCGGTCACCCGTCATCATAGAGAAGGGGGGCAAGGTGGATGTTGGTGGGCATTTAATCGACCTAAAGAGAGTTGTGCTTGAAGGTTCCGCGGCAACCCCTGTAACCAAGATTTCAGCGGAACAATGGGGTCGTCCATAGATGACTTCTGCAATGACAGCACGGCTGTACAAAAGGTGCTTGGGG

>CF-CF10-20260129-1

atgttggggaaatgcttgaccgcgggctgctgctcgcaattgccttttttgtggtttatcgtgccgttctgttttgttgcgctcgtcaacgccaacgacaacagcagctcccacttacaattgatttataacctaacgatatgtgagctgaatggcacagattggctgaaagaccactttccttgggcggtggagactttcgttatttttcctgtgttaactcatattgtctcttacggtgccctcaccactagccattttcttgacacggtcggtcttatcactgtgtccaccgccgggtattatcacgggcggtatgtattgagtagcatctacgctgtctgcgccctagctgcgcttgtttgcttcgccattaggttggtaaagaattgtatgtcctggcgctactcttgcaccagatatactaattttcttctggacactaagggaaaactctatcgttggcggtcacccgtcatcatagagaaggggggcaaggtggatgttggcggtcatttaatcgacctaaagagagttgtgcttgaaggttccgcggcaacccctgtaaccaagatttcagcggaacaatggggtcgtccatag

>CF-CF10-20260129-2

atgttggggaaatgcttgaccgcgggctgctgctcgcaattgccttttttgtggtgtatcgtgccgttctgtttagttgcgctcgtcaacgccgacgacaacagcagctcccacttacaattgatttataacctaacgatatgtgagctgaatggcacagattggctgaatgaacacttttcttgggcggtggagactttcgttatttttcctgtgttaactcatattgtctcttacggtgccctcaccactagccattttcttgacacggtcggtcttatcactgtgtccaccgccgggtattatcacgggcggtatgtattgagtagcatctacgctgtctgcgccctagctgcgcttagttgcttcgccattaggttggtaaaaaattgcatgtcctggcgctactcttgtaccagatatactaattttcttctggacactaagggaaaactctatcgttggcggtcacccgtcatcatagagaaggggggcaaggtggatgttggtggtcatttaatcgacctaaagagagttgtgcttgaaggttccgcggcaacccctgtaaccaagatttcagcggaacaatggggtcgtccatag

>CF-CF10-20260129-3

atgttggggaaatgcttgaccgcgggctgctgctcgcaattgcc-tttttgtggtttatcgtgccgttctgtttagttgcgctcgtcaacgccaacgacaacagcagctcccacttacaattgatttataacctaacgatatgtgagctgaatggcacagattggctgaaagaccactttccttgggcggtggagactttcgttatttttcctgtgttaactcatattgtctcttacggtgccctcaccactagccattttcttgacacggtcggtcttatcactgtgtccaccgccgggtattatcacgggcggtatgtattgagtagcatctacgctgtctgcgccctagctgcgcttgtttgcttcgccattaggttggtaaagaattgcatgtcctggcgctactcttgcaccagatatactaattttcttctggacactaagggaaaactctatcgttggcggtcacccgtcatcatagagaaggggggcaaggtggatgttggcggtcatttaatcgacctaaagagagttgtgcttgaaggttccgcggcaacccctgtaaccaagatttcagcggaacaatggggtcgtccatag

>CF-CF10-20260129-4

atgttggggaaatgcttgaccgcgggctgctgctcgcaattgccttttttgtggtgtatcgtgccgttctgtttagttgcgctcgtcaacgccgacgacaacagcagctcccacttacaattgatttataacctaacgatatgtgagctgaatggcacagattggctgaatgaacacttttcttgggcggtggagactttcgttatttttcctgtgttaactcatattgtctcttacggtgccctcaccactagccattttcttgacacggtcggtcttatcactgtgtccaccgccgggtattatcacgggcggtatgtattgagtagcatctacgctgtctgcgccctagctgcgcttgtttgcttcgccattaggttggtaaaaaattgtatgtcctggcgctactcttgtaccagatatactaattttcttctggacactaagggaaaactctatcgttggcggtcacccgtcatcatagagaaggggggcaaggtggatgttggcggtcatttaatcgacctaaagagagttgtgcttgaaggttccgcggcaacccctgtaaccaagatttcagcggagcaatggggtcgtccatag

>CF-CF10-20260129-5

atgttggggaaatgcttgaccgcgggctgctgctcgcaattgccttttttgtggtttatcgtgccgttctgtttagttgcgctcgtcaacgccaacgacaacagcagctcccacttacaattgatttataacctaacgatatgtgagctgaatggcacagattggctgaagaacacttttccttgggcggtggagactttcgttatttttcctgtgttaactcatattgtctcttacggtgccctcaccactagccattttcttgacacggtcggtcttatcactgtgtccaccgccgggtattatcacgggcggtatgtattgagtagcatctacgctgtctgcgccctagctgcgcttgtttgcttcgccattaggttggtaaagaattgcatgtcctggcgctactcttgcaccagatatactaattttcttctggacactaagggaaaactctatcgttggcggtcacccgtcatcatagagaaggggggcaaggtggatgttggtggtcatttaatcgacctaaagagagttgtgcttgaaggttccgcggcaacccctgtaaccaagatttcagcggaacaatggggtcgtccatag

>CF-CF11-20250602-123

ATGTTGGGGAAATGCTTGACCGCGGGCTGCTGCTCGCAATTGCCTTTTTTGTGGTTTATCGTGCCGTTCTGTTTAGTTGCGCTCGTCAACGCCAACGACAACAGCAGCTCCCACTTACAATTGATTTATAACCTAACGATATGTGAGCTGAATGGCACAGATTGGCTGAAAGACCACTTTTCTTGGGCGGTGGAGACTTTCGTTATTTTTCCTGTGTTAACTCATATTGTCTCTTACGGTGCCCTCACCACTAGCCATTTTCTTGACACGGTCGGTCTTATCACTGTGTCCACCGCCGGGTATTATCACGGGCGGTATGTATTGAGTAGCATCTACGCTGTCTGCGCCCTAGCTGCGCTTGTTTGCTTCGCCATTAGGTTGGTAAAGAATTGCATGTCCTGGCGCTACTCTTGCACCAGATATACTAATTTTCTTCTGGACACTAAGGGAAAACTCTATCGTTGGCGGTCACCCGTCATCATAGAGAAGGGGGGCAAGGTGGATGTTGGTGGTCATTTAATCGACCTAAAGAGAGTTGTGCTTGAAGGTTCCGCGGCAACCCCTGTAACCAAGATTTCAGCGGAACAATGGGGTCGTCCATAG

>CF-11-20250917-1

ATGTCGGGGAAATGCTTGACCGCGGGCTGTTGCTCGCAATTGCCTTTTTTGTGGTATATCGTGCCGTTCTGTTTTGTTGTGCTCGTCAACGCCAACAACAGCAGCAGCTCCCATTTACAGTTGATTTACAACCTGACGATATGTGAGCTGAATGGCACAGATTGGCTAAATAGAAGTTTTGATTGGGCAGTGGAGACCTTTGTTATTTTCCCTGTGTTGACTCATATTGTCTCCTATGGCGCCCTAACCACCAGCCATTTTCTTGACACAGTTGGCCTGATCACCGTGTCTACCGCTGGGTATTACCACGGGCGGTATGTCCTGAGCAGCATTTACGCCGTCTGCGCCCTAGCTGCGTTAACTTGCTTTGTCATCAGGCTAACAAAAAATTGCATGTCCTGGCGTTACTCATGCACCAGATACACTAACTTTCTTCTGGACACCAAGGGCAAACTCTATCGTTGGCGGTCTCCCGTCATCGTGGAGAAAGGGGGTAAAGTTGAGGTCGCAGGCCACCTGATCGACCTCAAGAGAGTTGTGCTTGACGGTTCCGCGGCAACCCCTATAACCAAAGTTTCAGCGGAACAATGGGGTCGTCCTTAG

>CF-11-20250917-2

ATGTTGGGGAAATGCTTGACCGCGGGTTATTGCTCACAATCGGTTTTTTTGTGGTGTATCGTGCCATTCTGTTTTGCTGTGCTCGTCAACGCCAGCGGCAACAGCAGCTCTCACTTACAATTGATCTATAACCTGACGATATGTGAGCTGAATGGCACAGACTGGTTGAACCAAAGATTTGACTGGGCAGTGGAGACTTTCGTCATTTTTCCTGTCTTGACCCATATCGTCTCTTACGGCGCCCTTACCACTAGCCATTTTCTTGACACGGTCGGCCTAATCACCGTGTCCACCGCTGGGTATTATCATAAGCGGTATGTATTGAGTAGCATCTACGCTGTCTGTGCCCTAGCCGCGTTGGTTTGCTTCGCCATTAGGTTGGCAAAAAATTGTATGTCCTGGCGCTACTCATTCACCAGATATACTAATTTTCTTCTGGACTCTAAGGGCAAACTCTACCGTTGGCGGTCACCCGTCATCATAGAGAAAGAGGGCAAAGTTGATGTGGGGGGTCAGTTAATCGACCTCAAGAGAGTTGTGCTTGATGGTTCCGCGGCGACCCCTGTAACCAAGATTTCAGCGGAACAATGGGGTCGTCCATAG

>CF-11-20251024-31

CCCCGCTTTTTGCCATTCTGCTGGCAATTTGAATGTTCAGGTATGTCGGGGAAATGCTTGACCGCGGGCTGTTGCTCGCAATTGCCTTTTTTGTGGTATATCGTGCCGTTCTGTTTTGTTGTGCTCGTCAACGCCAACAACAGCAACAGCTCCCATTTACAGTTGATTTATAACCTGACGATATGTGAGCTGAATGGCACAGATTGGCTAGATAAAAATTTTGATTGGGCAGTGGAGACCTTTGTTATTTTCCCCGTGTTGACTCATATTGTCTCCTATGGCGCCCTAACCACCAGCCATTTCCTTGACACAGTTGGCCTGATCACCGTGTCTACCGCTGGGTATTACCACGGGCGGTATGTCCTGAGCAGCATTTACGCCGTCTGCGCCCTAGCTGCGTTAACTTGCTTTGTCATCAGGCTAACAAAAAATTGCATGTCCTGGCGCTACTCTTGCACCAGATATACTAATTTTCTTCTGGACACTAAGGGAAAACTCTATCGCTGGCGGTCACCCGTCATCATAGAGAAGGGGGGCAAGGTGGATGTTGGGGGTCACTTAATTGACCTAAAGAGAGTTGTGCTTGAAGGTTCCGCGGCGACTCCTGTAACCAAGATTTCAGCGGAACAATGGGGTCGTCCATAGACGACTTCTGCAATGACAGCACGGCTGTACAAAAGGTGCTTGGGG

>CF11-20251216-16

TGTTTTTAGGCCCGTCTTTTTGCCATTCTGCTGGCAATTTGAATGTTCAGGTATGTCGGGGAAATGCTTGACCGCGGGCTGTTGCTCGCAATTGCCTTTTTTGTGGTATATCGTGCCGTTCTGTTTTGTTGTGCTCGTCAACGCCAACAACAGCAGCAGCTCCCATTTACAGTTGATTTACAACCTGACGATATGTGAGCTGAATGGCACAGATTGGCTAAATAAAAGTTTTGATTGGGCAGTGGAGACCTTTGTTATTTTCCCTGTGTTGACTCATATTGTCTCCTATGGCGCCCTAACCACCAGCCATTTTCTTGACACAGTTGGCCTGATCACCGTGTCTACCGCTGGGTATTACCACGGGCGGTATGTCCTGAGCAGCATTTACGCCGTCTGCGCCCTAGCTGCGTTAACTTGCTTTGTCATCAGGCTAACAAAAAATTGCATGTCCTGGCGTTACTCATGCACCAGATACACTAACTTTCTTCTGGACACCAAGGGCAAACTCTATCGTTGGCGGTCTCCCGTCATCGTGGAGAAAGGGGGTAAAGTTGAGGTCGCAGGCCACCTGATCGACCTCAAAAGAGTTGTGCTTGACGGTTCCGCGGCAACCCCTATAACCAAAGTTTCAGCGGAACAATGGGGTCGTCCTTAGACGACTTCTGCTATGACAGCAAAGCCCCGCAAAAGGTGCTTTGGG

>CF11-20251219-18

GTTTTAGGCCTGTCTTTTTGCCATTCTGCTGGCAATTTGAATGTTCAGGTATGTTGGGGA

AATGCTTGACCGCGGGCTGCTGCTCGCAATTGCCTTTTTTGTGGTGTATCGTGCCGTTC

TGTTTAGTTGCGCTCGTCAACGCCAACAACGACAGCAGCTCCCACTTACAATTGATTTA

TAACCTAACGATATGTGAGCTGAATGGCACAGATTGGCTGAACAACCACTTTTCTTGGG

CGGTGGAGACCTTCGTCATTTTTCCTGTGTTGACTCATATCGTCTCCTATGGCGCCCTAA

CCACCAGCCACTTCCTTGACACAGTCGGCCTGATCACCGTGTCTACTGCTGGATATTAC

CACGGGCGGTATGTCTTGAGCAGTATTTACGCCGTCTGCGCCTTAGCTGCGCTAATTTG

CTTCGTCATCAGGCTAACAAAAAATTGCATGTCCTGGCGCTACTCTTGCACCAGATATA

CTAATTTTCTTCTGGACACAAAGGGAAATCTCTATCGTTGGCGGTCACCCGTCATCATA

GAGAAGGGGGGCAAGGTGGATGTTGGGGGTCATTTAATCGACCTAAAGAGAGTTGTG

CTTGAAGGTTCCGCGGCAACCCCTGTAACCAAGATTTCAGCGGAACAATGGGGTCGTC

CATAGACGACTTCTGCAATGACAGCACGGCTGTACAAAAGGTGCTTTTGGGCCGTTTT

TCAA

>CF-CF12-20250527-103

ATGTCGGGGAAATGCTTGACCGCGGGCTGTTGCTCGCAATTGCCTTTTTTGTGGTATATCGTGCCGTTCTGTTTTGTTGTGCTCGTCAACGCCAACAACAGCAGCAGCTCCCATTTACAGTTGATTTATAACCTGACGATATGTGAGCTGAATGGCACAGATTGGCTAAATAAAAGTTTTGATTGGGCAGTGGAGACCTTTGTTATTTTCCCTGTGTTGACTCATATTGTCTCCTATGGCGCCCTAACCACCAGCCATTTCCTTGACACAGTTGGCCTGATCACCGTGTCTACCGCTGGGTATTACCACGGGCGGTATGTCCTGAGCAGCATTTACGCCGTCTGCGCCCTAGCTGCGTTAACTTGCTTTGTCATCAGGCTAACAAAAAATTGCATGTCCTGGCGTTACTCATGCACCAGATACACTAACTTTCTTCTGGACACCAAGGGCAAACTCTATCGTTGGCGGTCTCCCGTCATCGTTGAGAAAGGGGGTAAAGTTGAGGTCGCAGGCCACCTGATCGACCTCAAGAGAGTTGTGCTTGACGGTTCCGCGGCAACCCCTATAACCAAAGTTTCAGCGGAACAATGGGGTCGTCCTTAG

>CF-CF12-20250809-94

ATGTTGGGGAAATGCTTGACCGCGGGCTGCTGCTCGCAATTGCCTTTTTTGTGGTTTATCGTGCCGTTCTGTTTAGTTGCGCTCGTCAACGCCAACGACAACAGCAGCTCCCACTTACAATTGATTTATAACCTAACGATATGTGAGCTGAATGGCACAGATTGGCTGAAAGACCACTTTTCTTGGGCGGTGGAGACTTTCGTTATTTTTCCTGTGTTAACTCATATTGTCTCTTACGGTGCCCTCACCACTAGCCATTTTCTTGACACGGTCGGCCTTATCACTGTGTCCACCGCCGGGTATTATCACGGGCGGTATGTATTAAGTAGCATCTACGCTGTCTGCGCCCTAGCTGCGCTTGTTTGCTTCGCCATTAGGTTGGTAAAGAATTGCATGTCCTGGCGCTACTCTTGCACCAGATATACTAATTTTCTTCTGGACACTAAGGGAAAACTCTATCGTTGGCGGTCACCCGTCATCATAGAGAAGGGGGGCAAGGTGGATGTTGGTGGTCATCTAATCGACCTAAAGAGAGTTGTGCTTGAAGGTTCCGCGGCAACCCCTGTAACCAAGATTTCAGCGGAACAATGGGGTCGTCCATAG

>CF-CF12-20250809-95

ATGTCGGGGAAATGCTTGACCGCGGGCTGTTGCTCGCAATTGCTTTTTTTGTGGTATATCGTGCCGTTCTGTTTTGTTGTGCCCGTCAACGCCAACAACAGCAGCAGCTCCCATTTACAGTTGATTTATAACCTGACGATATGTGAGCTGAATGGCACAGATTGGCTAAATAAAAGTTTTGATTGGGCAGTGGAGACCTTTGTTATTTTCCCTGTGTTGACTCATATTGTCTCCTATGGCGCCCTAACCACCAGCCATTTCCTTGACACAGTTGGCCTGATCACCGTGTCTACCGCTGGGTATTACCACGGGCGGTATGTCCTGAGCAGCATCTACGCCGTCTGCGCCCTAGCTGCGTTAACTTGCTTTGTCATCAGGCTAACAAAAAATTGCATGTCCTGGCGTTACTCATGCACCAGATACACTAACTTTCTTCTGGACACCAAGGGCAAACTCTATCGTTGGCGGTCTCCCGTCATCGTGGAGAAAGGGGGTAAAGTTGAGGTCGCAGGCCACCTGATCGACCTCAAGAGAGTTGTGCTTGACGGTTCCGCGGCAACCCCTATAACCAAAGTTTCAGCGGAACAATGGGGTCGTCCTTAG

>CF12-20260106-9

CCCCGCTTTTTGCCATTCTGCTGGCAATTTGAATGTTCAGGTATGTCGGGGAAATGCTTGACCGCGGGCTGTTGCTCGCAATTGCCTTTTTTGTGGTATATCGTGCCGTTCTGTTTTGTTGTGCTCGTCAACGCCAACAACAGCAGCAGCTCCCATTTACAGTTGATTTACAACCTGACGATATGTGAGCTGAATGGCACAGATTGGCTAAATAAAAGTTTTGATTGGGCAGTGGAGACCTTTGTTATTTTCCCTGTGTTGACTCATATTGTCTCCTATGGCGCCCTAACCACCAGCCATTTTCTTGACACAGTTGGCCTGATCACCGTGTCTACCGCTGGGTATCACCACGGGCGGTATGTCCTGAGCAGCATTTACGCCGTCTGCGCCCTAGCTGCGTTAACTTGCTTTGTCATCAGGCTAACAAAAAATTGCATGTCCTGGCGTTACTCATGCACCAGATACACTAACTTTCTTCTGGACACCAAGGGCAAACTCTATCGTTGGCGGTCTCCCGTCATCGTGGAGAAAGGGGGTAAAGTTGAGGTCGCAGGCCACCTGATCGACCTCAAAAGAGTTGTGCTTGACGGTTCCGCGGCAACCCCTATAACCAAAGTTTCAGCGGAACAATGGGGTCGTCCTTAGACGACTTCTGCTATGACAGCAAAGCCCCGCAAAAGGTGCTTTGGG

>CF12-20260106-10

CCCCGCTTTTTGCCATTCTGCTGGCAATTTGAATGTTCAGGTATGTTGGGGAAATGCTTGACCGCGGGCTGCTGCTCGCAATTGCCTTTTTTATGGTTTATCGTGCCGTTCTGTTTAGTTGCGCTCGTCAACGCCAACGACAACAGCAGCTCCCACTTACAATTGATTTATAACCTAACGATATGTGAGCTGAATGGCACAGATTGGCTGAAAGACCACTTTTCTTGGGCGGTGGAGACTTTCGTTATTTTTCCTGTGTTAACTCATATTGTCTCTTACGGTGCCCTCACCACTAGCCATTTTCTTGACACGGTCGGCCTTATCACTGTGTCCACCGCCGGGTATTATCACGGGCGGTATGTATTGAGTAGCATCTACGCTGTCTGCGCCCTAGCTGCGCTTGTTTGCTTCGCCATTAGGTTGGTAAAGAATTGCATGTCCTGGCGCTACTCTTGCACCAGATATACTAATTTTCTTCTGGACACTAAGGGAAAGCTCTATCGTTGGCGGTCACCCGTCATCATAGAGAAGGGGGGCAAGGTGGATGTTGGTGGTCATTTAATCGACCTAAAGAGAGTTGTGCTTGAAGGTTCCGCGGCAACCCCTGTAACCAAGATTTCAGCGGAACAATGGGGTCGTCCATAGACAACTTCTGCAATGACAGCACGGCTGTACAAAAGGTGCTTGGGGGGTTTCA

>CF-CF12-20260120-1

atgtcggggaaatgcttgaccgcgggctgttgctcgcaattgccttttttttggtatatcgtgccgttctgttttgttgtgctcgtcaacgccaacaacagcagcagctcccatttacagttgatttacaacctgacgatatgtgagctgaacggcacagattggctaaataaaagttttgattgggcagtg--gacctttgttattttccctgtgttgactcatattgtctcctatggcgccctaaccaccagccattttcttgacacagttggcctgatcaccgtgtctaccgctgggtattaccacgggcggtatgtcctgagcagcatttacgccgtctgcgccctagctgcgtt-acttgctttgtcatcaggctaacaaaaaattgcatgtcctggcgttactcatgcaccagatacactaactttcttctggacaccaagggcaaactctatcgttggcggtctcccgtcatcgtggagaaagggggtaaagttgaggtcgcaggccacctgatcgacctcaaaagagttgtgcttgacggttccgcggcaacccctataaccaaagtttcagcggaacaatggggtcgtccttag

>CF-CF12-20260120-2

atgtcggggaaatgcttgaccgcgggctgttgctcgcaattgccttttttgtggtatatcgtgccgttctgttttgttgtgctcgtcaacgccaacaacagcagcagctcccatttacagttgatttacaacctgacgatatgtgagctgaatggcacagattggctaaataaaagttttgattgggcagtggagacctttgttattttccctgtgttgactcatattgtctcctatggcgccctaaccaccagccattttcttgacacagttggcctgatcaccgtgtctaccgctgggtattaccacgggcggtatgtcctgagcagcatttacgccgtctgcgccctagctgcgttaacttgctttgtcatcaggctaacaaaaaattgcatgtcctggcgttactcatgcaccagatacactaactttcttctggacaccaagggcaaactctatcgttggcggtctcccgtcatcgtggagaaagggggtaaagttgaggtcgcaggccacctgatcgacctcaaaagagttgtgcttgacggttccgcggcaacccctataaccaaagtttcagcggaacaatggggtcgtccttag

>CF-CF12-20260120-3

atgtcggggaaatgcttgaccgcgggctgttgctcgcaattgccttttttgtggtatatcgtgccgttctgttttgttgtgctcgtcaacgccaacaacagcagcagctcccatttacagttgatttacaacctgacgatatgtgagctgaatggcacagattggctaaataaaaggtttgattgggcagtggagacctttgtgattttccctgtgttgactcatattgtctcctatggcgccctaaccaccagccattttcttgacacagttggcctgatcaccgtgtctaccgctgggtattaccacgggcggtatgtcctgagcagcatttacgccgtctgcgccctagctgcgttaacttgctttgtcatcaggctaacaaaaaattgcatgtcctggcgttactcatgcaccagatacactaactttcttctggacaccaagggcaaactctatcgttggcggtctcccgtcatcgtggagaaagggggtaaagttgaggtcgcaggccacctgatcgacctcaaaagagttgtgcttgacggttccgcggcaacccctataaccaaagtttcagcggaacaatggggtcgtccttag

>CF-CF12-20260120-4

atgtcggggaaatgcttgaccgcgggctgttgctcgcaattgccttttttgtggtatatcgtgccgttctgttttgttgtgctcgtcaacgccaacaacagcagcagctcccatttacagttgatttacaacctgacgatatgtgagctgaacggcacagattggctaaataaaagttttgattgggcagtggagacctttgttattttccctgtgttgactcatattgtctcctatggcgccctaaccaccagccattttcttgacacagttggcctgatcaccgtgtctaccgctgggtattaccacgggcggtatgtcctgagcagcatttacgccgtctgcgccctagctgcgttaacttgctttgtcatcaggctaacaaaaaattgcatgtcctggcgttactcatgcaccagatacactaactttcttctggacaccaagggc-aactctatcgttggcggtctcccgtcatcgtggagaaagggggtaaag-tgaggtcgcaggccacctgatcgacctcaaaagagttgtgcttgacggttccgcggcaacccctataaccaaagtttcagcggaacaatggggtcgtccttag

>CF-CF12-20260120-5

atgttggggaaatgcttgaccgcgggctgctgctcgcaattgccttttttatggtttatcgtgccgtcctgtttagttgcgctcgtcaacgccaacgacaacagcagctcccacttacaattgatttataacctaacaatatgtgagctgaatggcacagattggctgaaagaccactttccttgggcggtggagactttcgttatttttcctgtgttaactcatattgtctcttacggtgccctcaccactagccattttcttgacacggtcggccttatcactgtgtccaccgccgggtattatcacgggcggtatgtattgagtagcatctacgctgtctgcgccctagctgcgcttgtttgcttcgccattaggttggtaaagaattgcatgtcctggcgctactcttgcaccagatatactaattttcttctggacactaagggaaaactctatcgttggcggtcacccgtcatcatagagaaggggggcaaggtggatgttggtggtcatttaatcgacctaaagagagttgtgcttgaaggttccgcggcaacccctgtaaccaagatttcagcggaacaatggggtcgtccatag

>CF-CF12-20260129-1

atgttggggaaatgcttgaccgcgggctgctgctcgcaattgccttttttgtggtttatcgtgccgttctgtttagttgcactcgtcaacgccaacgacaacagcagctcccacttacaattgatttataacctaacaatatgtgagctgaatggcacagattggctgaaagaccacttttcttgggcggtggagactttcgttatttttcctgtgttaactcatattgtctcttacggtgccctcaccactagccattttcttgacacggtcggtcttatcactgtgtcctccgccgggtattatcacgggcggtatgtattgagtagcatctacgctgtctgcgccctagctgcgcttgtttgcttcgccattaggttggtaaagaattgcatgtcctggcgctactcttgcaccagatatactaattttcttctggacactaagggaaaactctatcgttggcggtcacccgtcatcatagagaaggggggcaaggtggatgttggtggtcatttaatcgacctaaaaagagttgtgcttgaaggttccgcggcaacccctgtaaccaagatttcagcggaacaatggggtcgtccatag

>CF-CF12-20260129-2

atgttggggaaatgcttgaccgcgggctgctgctcgcaattgccttttttgtggtttatcgtgccgttctgtttagttgcgctcgtcaacgccaacgacaacagcagctcccacttacaattgatttataacctaacaatatgtgagctgaatggcacagattggctgaaagaccacttttcttgggcggtggagactttcgttatttttcctgtgttaactcatattgtctcttacggtgccctcaccactagccattttcttgacacggtcggtcttatcactgtgtcctccgccgggtattatcacgggcggtatgtattgagtagcatctacgctgtctgcgccctagctgcgcttgtttgcttcgccattaggttggtaaagaattgcatgtcctggcgctactcttgcaccagatatactaattttcttctggacactaagggaaaactctatcgttggcggtcacccgtcatcatagagaaggggggcaaggtggatgttggtggtcatttaatcgacctaaaaagagttgtgcttgaaggttccgcggcaacccctgtaaccaagatttcagcggaacaatggggtcgtccatag

>CF-CF12-20260129-3

atgtcggggaaatgcttgaccgcgggctgttgctcgcaattgccttttttgtggtatatcgtgccgttctgttttgttgtgctcgtcaacgccaacaacagcagcagctcccatttacagttgatttacaacctgacgatatgtgagctgaatggcacagattggctaaataaaagttttgattgggcagtggagacctttgttattttccctgtgttgactcatattgtctcctatggcgccctaaccaccagccattttcttgacacagttggcctgatcaccgtgtctaccgctgggtattaccacgggcggtatgtcctgagcagcatttacgccgtctgcgccctagctgcgttaacttgctttgtcatcaggctaacaaaaaattgcatgtcctggcgttactcatgcaccagatacactaactttcttctggacaccaagggcaaactctatcgttggcggtctcccgtcatcgtggagaaagggggtaaagttgaggtcgcaggccacctgatcgacctcaaaagagttgtgcttgacggttccgcggcaacccctataaccaaagtttcagcggaacaatggggtcgtccttag

>CF-CF12-20260130-1

atgttggggaaatgcttgaccgcgggctgctgctcgcaattgctttttttgtggtttatcgtgccgttctgtttagttgcgctcgtcaacgccaacgacaacagcagctcccacttacaattgatttataacctaacgatatgtgagctgaatggcacagattggctgaaagaccacttttcttgggcggtggagactttcgttatttttcctgtgttaactcatattgtctcttacggtgccctcaccactagccattttcttgacacggtcggtcttatcactgtgtccaccgccgggtattatcacgggcggtatgtattgagtagcatctacgctgtctgcgccctagctgcgcttgtttgcttcgccattaggttggtaaagaattgcatgtcctggcgctactcttgcaccagatatactaattttcttctggacactaagggaaagctctatcgttggcggtcacccgtcatcatagagaaggggggcaaggtggatgttggtggtcatttaatcgacctaaaaagagttgtgcttgaaggttccgcggcaacccctgtaaccaagatttcagcggaacaatggggtcgtccatag

>CF-CF12-20260130-2

atgttggggaaatgcttgaccgcgggctgctgctcgcaattgccttttttgtggtttatcgtgccgttctgtttagttgcgctcgtcaacgccaacgacaacagcagctcccacttacaattgatttataacctaacaatatgtgagctgaatggcacagattggctgaaagaccacttttcttggcggtggagactttcgttatttttcctgtgttaactcatattgtctcttacggtgccctcaccactagccattttcttgacacggtcggtcttatcactgtgtcctccgccgggtattatcacgggcggtatgtattgagtagcatctacgctgtctgcgccctagctgcgcttgtttgcttcgccattaggttggtaaagaattgcatgtcctggcgctactcttgcaccagatatactaattttcttctggacactaagggaaaactctatcgttggcggtcacccgtcatcatagagaaggggggcaaggtggatgttggtggtcatttaatcgacctaaaaagagttgtgcttgaaggttccgcggcaacccctgtaaccaagatttcagcggaacaatggggtcgtccatag

>CF-CF12-20260130-3

atgttggggaaatgcttgaccgcgggctgctgctcgcaattgccttttttgtggtttatcgtgccgttctgtttagttgcgctcgtcaacgccaacgacaacagcagctcccacttacaattgatttataacctaacaatatgtgagctgaatggcacagattggctgaaagaccacttttcttgggcggtggagactttcgttatttttcctgtgttaactcatattgtctcttacggtgcactcaccactagccattttcttgacacggtcggtcttatcactgtgtcctccgccgggtattatcacgggcggtatgtattgagtagcatctacgctgtctgcgcctagctgcgcttgtttgcttcgccattaggttggtaaagaattgcatgtcctggcgctactcttgcaccagatatactaattttcttctggacactaagggaaagctctatcgttggcggtcacccgtcatcatagagaaggggggcaaggtggatgttggtggtcatttaatcgacctaaagagagttgtgcttgaaggttccgcggcaacccctgtaaccaagatttcagcggaacaatggggtcgtccatag

>CF-13-20250827

ATGTTGGGGAAATGCTTGACCGCGGGCTGCTGCTCGCAATTGCCTTTTTTGTGGTGTATCGTGCCGTTCTGTTT

AGTTGCGCTCGTCAACGCCAACAACGACAGCAGCTCCCACTTACAATTGATTTATAACCTAACGATATGTGAGC

TGAATGGCACAGATTGGCTGAACAAACACTTTCCTTGGGCGGTGGAGACTTTCGTTATTTTTCCTGTGTTAACG

CATGTTGTCTCTTACGGTGCCCTCACCACTAGCCATTTTCTTGACACGGTCGGTCTTATCACTGTGTCCACCGC

CGGGTATTATCACGGGCGGTATGTATTGAGTAGCATCTACGCTGTCTGCGCCCTAGCTGCGCTTGTTTGCTTCG

CCATTAGGTTGGTAAAGAATTGCATGTCCTGGCGCTACTCTTGCACCAGATATACTAATTTTCTTCTGGACACA

AAGGGAAAACTCTATCGTTGGCGGTCACCCGTCATCATAGAGAAGGGGGGCAAGGTGGATGTTGGGGGTCATTT

AATCGACCTAAAGAGAGTTGTGCTTGAAGGTTCCGCGGCAACCCCTGTAACCAAGATTTCAGCGGAACAATGGG

GTCGTCCATAG

>CF13-20251229-6

TTGTTTTAGCCTGTCTTTTTGCCATTCTGCTGGCGATTTGAATGTTCAGGTATGTTGGGGAAATGCTTGACCGCGGGCTGCTGCTCGCAATTGCCTTTTTTTTGGTGTATCGTGCCGTTCTGTTTAGTTGCGCTCGTCAACGCCAACGACAACAGCAGCTCCCACTTGCAATTGATTTATAACCTAACGATATGTGAGCTGAATGGCACAGATTGGCTGAAAGAACACTTTTCTTGGGCGGTGGAGACTTTCGTTATTTTTCCTGTGTTAACTCATATTGTCTCCTATGGCGCCCTAACCACCAGTCACTTCCTTGACACAGTCGGCCTGATCACCGTGTCTACTGCTGGATACTACCACGGGCGGTATGTCTTGAGCAGTATTTACGCCGTCTGCGCCTTAGCTGCGCTAATTTGCTTCGTCATCAGGCTAACAAAAAATTGCATGTCCTGGCGTTACTCATGCACCAGATACACTAATTTTCTCCTGGACACCAAGGGAAAACTCTATCGTTGGCGGTCACCCGTCATCATAGAGAAGGGGGGCAAGGTGGATGTTGGGGGTCATTTAATCGACCTAAAGAGAGTTGTGCTTGAAGGTTCCGCGGCAACCCCTGTAACCAAGATTTCAGCGGAACAATGGGGTCGTCCATAGACGACTTCTGCAATGACAGCACGGCTGTACAAAAGGTGCTTTGGGCCGTTTTTC

>CF-CF15-20260120-1

atgttggggaaatgcttgaccgcgggctgctgctcgcaattgctttttttgtggtgtatcgtgccgttctgtttagttgcgctcgtcaacgccaacaacgacagcagctcccacttacaattgatttataacctaacgatatgtgagctgaatggcacagattggctgaacaaacactttccttgggcggtggagactttcgttatttttcctgtgttaacgcatgttgtctcttgtggtgccctcaccactagccattttcttgacacggtcggtcttatcactgtgtccaccgccgggtattatcacgggcggtatgtgttgagtagcatctacgctgtctgcgccctagctgcgcttgtttgcttcgccattaggttggtaaagaattgcatgtcctggcgctactcttgcaccagatatactaattttcttctggacacaaagggaaaactctatcgttggcggtcacccgtcatcatagagaaggggggcaaggtggatgttgggggtcatttaatcgacctaaagagagttgtgcttgaaggttccgcggcaacccctgtaaccaagatttcagcggaacaatggggttgtccatag

>CF-CF15-20260120-5

atgttggggaaatgcttgaccgcgggctgctgctcgcaattgctttttttgtggtgtatcgtgccgttctgtttagttgcgctcgtcaacgccaacaacgacagcagctcccacttacaattgatttataacctaacgatatgtgagctgaatggcacagattggctgaacaaacactttccttgggcggtggagactttcgttatttttcctgtgttaacgcatgttgtctcttgtggtgccctcaccactagccattttcttgacacggtcggtcttatcactgtgtccaccgccgggtattatcacgggcggtatgtgttgagtagcatctacgctgtctgcgccctagctgcgcttgtttgcttcgccattaggttggtaaagaattgcatgtcctggcg---cctgttcaccagatatactaattttcttctggacacaaagggaaaactctatcgttggcggtcacccgtcatcgtcgag-aggggggcaaggtggatgttgggggtcatttaatcgacctggagtta-----tactgaaggttccgcggcaacccctgtaaccaagatttcagcggaacaatggggttgtccatag

>CF-CF15-20260120-9

gtatgttgggaacgcttgaccgcgggctgctgcttgcaattgccttttttgtggtgtatcgtgccgttctgtttagttgcgctcgtcaacgccaacaacgacagcagctcccacttacaattgatttataacctaacgatatgtgagccgaatggcacagattggctgaacaaacactttccttgggcggtggagactttcgttatctttcctgtgttaacgcatgttgtc---tgcggcgccctcgccactagccattttcttgacacggtcggtcttatcactgtgtccaccgccggg---gatcacgggcggtatgtattgagtagcatctacgctgtctgcgcccagcctcacc--gtttgcttcgccattaggttggtaaagaattgcatgtcctggcgctactcttgcaccagatatactaattttcttctggacacaaagggaaaactctatcgttggcggtcacccgtcatcatagagaaggggggcaaggtggatgttgggggtcatttaatcgacctaaagagagttgtgcttgaaggttccgcggcaacccctgtaaccaagatttcagcggaacaatggggtcgtccatag

>CF-CF15-20260120-12

atgttggggagatgcttgaccgcgggctgctgctcgcaattgctttttttgtggtgtatcgtgccgttttgttttgttgcgctcgtcaacgccaacaacagcaacagctcccatttacaattgatttataacctgacgatatgtgagctgaatggcacagaatggctaaatagaagttttgattgggccgtggagacctttgtcattttccctgtgttgactcatattgtctcctatggcgccctaaccaccagccatttccttgacacagttggcctgatcatcgtttctaccgctggatattatcatgggcggtatgtcttgagtagcatttacgccgtctgtgccctagctgcgttgacttgcttcgtcatcaggctaacaaaaaatttcatgtcctggcgttattcatgcaccagatacactaactttcttctggacaccaagggcaaactctatcgttggcggtctcccgtcatcatagagaaagggggtaaagttgaggtcgaaggccacctgatcgacctcaagagagttgtgcttgacggttccgcggcgacccctataaccaaagtttcagcggaacaatggggtcgtccttag

>CF-CF15-20260120-19

atgttggggaaatgcttgaccgcgggctgctgctcgcaattgctttttttgtggtgtatcgtgccgttctgtttagtctcgctcgtcaacgccagcaacaacagcagctcccatttacagttgatttataacctgacgatatgtgagctgaatggcacggattggctgaatcacaattttgattgggcagtggagaccttcgttatttttcctgtgttgactcatatcgtctcctacggcgccctaaccactagccacttccttgacacagtcggcctgatcaccgtgtctactgctggatattaccacgggcggtgtgtcttgagcagtatttacgccgtctgcgccttagctgcgctaatttgcttcgtcatcaggctaacaaaaaattgcatgtcctggcgttactcatgcaccagatacactaattttcttctggacaccaagggaaaactctatcgttggcggtctcccgtcatcgtggaaaaagggggaaaagttgaggtcgaaggccacctcttcgacctcaagaaagttgtgcttgatggttccgcagcgacccctgtaaccaaagtcacagcggaacaatggggtcgtccttag

>CF-CF15-20260120-29

atgttggggaaatgcttgaccgcgggctgctgctcgcaattgccttttttgtggtgtatcgtgccgttctgtttagttgcgctcgtcaacgccaacaacgacagcagctcccacttacaattgatttataacctaacgatatgtgagctgaatggcacagattggctgaacaaacactttccttgggcggtggagactttcgttatttttcctgtgttaacgcatgttgtctcttgtggtgccctcaccactagccattttcttgacacggtcggtcttatcactgtgtccaccgccgggtattatcacgggcggtatgtattgagtagcatctacgctgtctgcgccctagctgcgcttgtttgcttcgccattaggttggtaa------gcatgtcctggcgctactcttgcaccagatatactaattttcttctggacacaaaggg-aaactctatcgttggcggtcacccgtcatcatagagaagggggcaaaggtggatgttgggggtcatttaatcgacctaaagagagttgtgcttgaaggttccgcggcaacccctgtaaccaaga-ttcagcggaacaatggggtcgtccatag

>CF-CF15-20250604-127

ATGTTGGGGAAATGCTTGACCGCGGGCTGCTGCTCGCAATTGCCTTTTTTGTGGTGTATCGTGCCGTTCTGTTTGGTTGCGCTCGTCAACGCCAACGACAACAGCAGCTCCCACTTACAATTGATTTATAACCTAACGATATGTGAGCTGAATGGCACAGATTGGCTGAAAGAACACTTTTCTTGGGCGGTGGAGACTTTCGTTATTTTTCCTGTGTTAACTCATGTTGTCTCTTACGGTGCCCTCACCACTAGCCATTTTCTTGACACGGTCGGTCTTATCACTGTGTCCACCGCCGGGTATTATCACGGGCGGTATGTATTGAGTAGCATCTACGCTGTCTGCGCCCTAGCTGCGCTTGTTTGCTTCGCCATTAGGTTGGCAAAGAATTGCATGTCCTGGCGCTACTCTTGCACCAGATATACTAATTTTCTTCTGGACACTAAGGGAAAACTCTATCGTTGGCGGTCACCCGTCATCATAGAGAAGGGGGGCAAGGTGGATGTTGGGGGTCATTTAATCGACCTAAAGAGAGTTGTGCTTGAAGGTTCCGCGGCAACCCCTGTAACCAAGATTTCAGCGGAACAATGGGGTCGTCCATAG

>CF-CF15-20260120-11

atgttggggaaatgcttgaccgcgggctgctgctcgcaattgctttttttgtggtgtatcgtgccgttctgtttagtttcgctcgtcaacgccagcaacagcagcagctcccatttacagttgatttataacctgacgatatgtgagctgaatggcacggattggctgaatcgcacttttgattgggcagtggagaccttcgttatttttcctgtgttgactcatatcgtctcctacggcgccctaaccaccagccacttccttgacacagtcggcctgatcaccgtgtctactgctggatattaccacgggcggtatgtcttgagcagtatttacgccgtctgcgccttagctgcgctaatttgcttcgtcatcaggctaacaaaaaattgcatgtcctggcgttactcatgcaccagatacactaattttcttctggacaccaagggaaaactctatcgttggcggtctcccgtcatcgtggaaaaagggggtaaagttgaggtcgaaggccacctcatcgacctcaagaaagttgtgcttgatggttccgcggcgactcctataaccaaagttacagcggaacaatggggtcgtccttag

>CF-CF16-20260124-1

atgttggggaaatgcttgaccgcgggctgctgctcgcaattgccttttttgtggtgtatcgtgccgttctgtttagttgcgctcgtcaacgccaacaacgacagcagctcccacttacagttgatttataacctaacgatatgtgagctgaatggcacagactggctgaaagaacatttttcttgggcagtggagactttcgttatctttcctgtgttaactcatattgtctcttacggtgccctcaccactagccattttcttgacacggtcggcctaatcgctgtgtccaccgccgggtatcgtcacgggcggtatgtactgagtagcatctatgctgtctgcgccctggctgcgcttgtttgcttcgccattaggttggtaaaaaattgcatgtcctggcgctactcttgcaccagatatactaattttcttctggacactaaggggaaactctatcgctggcggtcacccgtcatcatagagaaggggggcaaggtggatgttgggggtcatttaattgacctaaagagagttgtgcttgaaggttccgcggcgactcctgtaaccaggatttcagcggaacaatggggtcgtccatag

>CF-CF16-20260124-2

atgttggggaaatgcttgaccgcgggctgctgctcgcaattgccttttttgtggtgtatcgtgccgttctgtttagttgcgctcgtcaacgccaacgacgacaacagctcccacttacaattgatttataacctaacgatatgtgagctgaatggcacagattggttgaacgaacactttccttgggcggtggagactttcgttatttttcctgtgttaacgcatgttgtctcttgtggtgccctcaccactagccattttcttgacacggtcggtcttatcactgtgtccaccgccgggtattatcacgggcggtatgtattgagtagcatctacgctgtctgcgccctagctgcgcttgtttgcttcgccattaggttggtaaagaattgcatgtcctggcgctactcttgcaccagatatactaattttcttctggacacaaagggaaaactctatcgttggcggtcacccgtcatcatagagaaggggggcaaggtggatgttgggggtcatttaatcgacctaaagagagttgtgcttgaaggttccgcggcaacccctgtaaccaagatttcagcggaacaatggggtcgtccatag

>CF-CF16-20260129-2

atgttggggaaatgcttgaccgcgggctgctgctcgcaattgctttttttgtggtgtatcgtgccgttctgtttagtttcgctcgtcaacgccagcaacagcagcagctcccatttacagttgatttataacctgacgatatgtgagctgaatggcacggattggctgaatcgcaattttgattgggcagtggagaccttcgttatttttcctgtgttgactcatatcgtctcctacggtgccctaaccaccagccacttccttgacacagtcggcctgatcaccgtgtctactgctggatattgccacgggcggtatatcttgagcagtatttacgccgtctgcgccttagctgcgctaatttgcttcgtcatcaggctaacaaaaaattgcatgtcctggcgttactcatgcaccagatacactaattttcttctggacaccaagggaaaactctatcgttggcggtctcccgtcatcgtggaaaaagggggtaaagttgaggtcgaaggccacctcatcgacctcaagaaagttgtgcttgatggttccgcggcgacccctataaccaaagttacagcggaacaatggggtcgtccttag

>CF-CF16-20260129-5

atgttggagaaatgcttgaccgcgggctgttgctcgcaattgctttttttgtggtgtatcgtgccgttctgttttgctgtgctcgccaacgccagcaacgacagcagctcccatctacagctgatttacaacttgacgctatgtgagctgaatggcacagattggctagctaacaaatttgattgggcagtggagagttttgtcatctttcccgttttgactcacattgtctcctatggtgccctcactactagccatttccttgacacagtcgctttagtcactgtgtctaccgccgggtttgttcacgggcggtatgtcctaagtagcatctacgcggtctgtgccctggctgcgttgacttgcttcgtcattaggtttgcaaagaattgcatgtcctggcgctacgcgtgtaccagatataccaactttcttctggacactaagggcatactctatcgttggcggtcgcctgtcatcatagagaaaaggggcaaagttgaggtcgaaggtcatctgatcgacctcaaaagagttgtgcttgatggttccgtggcaacccctataaccagagtttcagcggaacaatggggtcgtccttag

>CF-CF17-20250605-130

ATGTTGGGGAAATGCTTGACCGCGGGCTGCTGCTCGCAATTGCTTTTTTTGTGGTGTATCGTGCCGTTCTGTTTAGTTGCGTTCGTCAACGCCAACAACAGCAACAGCTCCCACTTACAGTTGATTTATAGCCTAACGATATGTGAGCTGAATGGCACAGAGTGGCTGAATAAACATTTTTCTTGGGCAGTGGAGACTTTCGTTATTTTTCCTGTGTTAACCCACATTGTCTCTTACGGTGCCCTCACCACTAGCCATTTTCTTGACACGGTCGGCCTAATCACTGTGTCCACCGCCGGGTATTGTCACGGGCGGCATGTACTGAGTAGCATCTACGCTGTCTGCGCCCTAGCTGCGCTTGTTTGCTTCGCCATTAGGTTGGTAAAAAATTGCATGTCCTGGCGCTACTCTTGCACCAGACACACTAATTTCCTTCTGGACACTAAGGGAAAACTCTATCGTTGGCGGTCACCCGTCATCATAGAGAAAGGGGGCAAGGTGGATGTTGGGGGTCATTTAATTGACCTGAAGAGAGTTGTGCTTGAAGGTTCCGCGGCGACTCCTGTAACCAAGATTTCAGCGGAACAATGGGGTCGTCCATAG

>CF-17-20250825

ATGTTGGAGAAATGCTTGACCGCGGGCTGTTGCTCGCAATTGCTTTCTTTGTGGTGTATCGTGCCGTTCTGTTT

TGCTGTGCTCGCCAACGCCAGCAACGACAGCAGCTCCCATCTACAGCTGATTTACAACTTGACGCTATGTGAGC

TGAATGGCACAGATTGGCTAGCTAACAAATTTGATTGGGCAGTGGAGAGTTTTGTCATCTTTCCCGTTTTGACT

CACATTGTCTCCTATGGTGCCCTCACTACCAGCCATTTCCTTGACACAGTCGCTTTAGTCACTGTGTCTACCGC

CGGGTTTGTTCACGGGCGGTATGTCCTAAGTAGCATCTACGCGGTCTGTGCCCTGGCTGCGTTGACTTGCTTCG

TCATTAGGTTTGCAAAGAATTGCATGTCCTGGCGCTACGCGTGTACCAGATATACCAACTTTCTTCTGGACACT

AAGGGCGGACTCTATCGTTGGCGGTCGCCTGTCATCATAGAGAAAAGGGGCAAAGTTGAGGTCGAAGGTCATCT

GATCGACCTCAAAAGAGTTGTGCTTGATGGTTCCGTGGCAACCCCTATAACCAGAGTTTCAGCGGAACAATGGG

GTCGTCCTTAG

>CF-17-20251001-38

ATGTTGGGGAAATGCTTGACCGCGGGTTACTGCTCATCATTGCTTTTTTTGTGGTGTATCGTGCTATCCTGTTC

TGTTGCGCTCGTCAACGCCAACAGCAACACCAGCTCTCATTTACAGTTGATTTATAACCTGACGATATGTGAGC

TGAATGGCACGGATTGGTTGAATGAAAGATTTGACTGGGCAGTGGAGACTTTCGTCATTTTTCCTGTTCTGACC

CATATTGTCTCGTACGGTGCCCTCACCACTAGCCATTTTCTTGACACGGTCGGTCTAATCACTGTGTCCACCGC

TGGGTATTACCATAAGCGGTATGTATTGAGTAGCATCTATGCTGTCTGTGCCCTAGCCGCGCTGGCTTGCTTCG

CCATCAGGTTGGCGAAAAATTGCATGTCCTGGCGCTACTCATGCACCAGATACACCAACTTTCTCCTTGACACT

AAAGGCAAACTCTACCGCTGGCGGTCACCCGTCATCATAGAGAAGGGGGGCAAAGTTGATGTGGGGGGTCATTT

AATTGACCTCAAGAGAGTCGTGCTTGATGGTTCCGCGGCGACCCCTATAACCAAGATTTCAGCGGAACAATGGG

GTCGTCCATAG

>CF17-20251106-23

CCCGCTTTTTGCCATTCTGCTGGCAATTTGAATGTTCAGGTATGTTGGGGAGATGCTTGATCGCGGGCTGTTGCTCGCAATTGCCTTTTTTGTGGTATATCGTGCCGTTCTGTTTTGTTGCGCTCGTCAACGCCAACAACAGCAGCAGCTCCCATTTACAGTTGATTTATAACCTGACGATATGTGAGCTGAATGGCACAGATTGGCTAAATAAAAGTTTTGATTGGGCAGTGGAGACCTTTGTTATTTTCCCTGTGTTGACTCATATTGTCTCCTATGGCGCCCTAACCACCAGCCATTTCCTTGACACAGTTGGCCTGATCACCGTGTCTACCGCTGGGTATTACCACGGGCGGTATGTCCTGAGTAGCATTTACGCCGTCTGCGCCCTAGCTGCGTTAACTTGCTTTGTCATCAGGCTAACAAAAAATTGCATGTCCTGGCGTTACTCATGCACCAGATACACTAACTTTCTTCTGGACACCAAGGGCAAACTCTATCGTTGGCGGTCTCCCGTCATCGTTGAGAAAGGGGGTAAAGTTGAGGTCGCAGGCCACCTGATCGACCTCAAGAGAGTTGTGCTTGACGGTTCCGCGGCAACCCCTATAACCAAAGTTTCAGCGGAACAATGGGGTCGTCCTTAGACGACTTCTGCTATGACAGCACAGCCCCGCTAAAGGTGCTTGGG

>CF-CF18-20250520-80

ATGTTGGGGAAATGCTTGACCGCGGGTTACTGCTCATCATTGCTTTTTTTGTGGTGTATCGTGCTATCCTGTTCTGTTGCGCTCGTCAACGCCAACAGCAACACCAGCTCTCATTTACAGTTGATTTATAACCTGACGATATGTGAGCTGAATGGCACGGATTGGTTGAACAAAAGATTTGACTGGGCAGTAGAGACTTTCGTCATTTTTCCTGTTCTGACCCATATTGTCTCGTACGGTGCCCTCACCACTAGCCATTTTCTTGACACGGTCGGCCTAATCACTGTGTCCACCGCTGGGTATTACCATAAGCGGTATGTATTGAGCAGCATCTATGCTGTCTGTGCCCTGGCTGCGTTGGCTTGCTTCGCCATCAGGTTGGCGAAAAATTGCATGTCCTGGCGCTACTCATGCACCAGATACACCAACTTTCTCCTTGACACTAAAGGCAAACTCTACCGCTGGCGGTCACCCGTCATCATAGAGAAGGGGGGCAAAGTTGATGTGGGGGGTCATTTAATTGACCTCAAGAGAGTCGTGCTTGATGGTTCCGCAGCGACCCCTATAACCAAGATTTCAGCGGAACAATGGGGTCGTCCATAG

>CF-CF18-20250808-68

ATGTTGGGGAAATGCTTGACCGCGGGCTGCTGCTCGCAATTGCTTTTTTTGTGGTGTATCGTGCCGTTCTGTTT

AGTTGCGTTCGTCAACGCCAACGACAACAACAGCTCCCACTTACAGTTGATTTATAACCTAACGATATGTGAGC

TGAATGGCACAGAGTGGCTGAATAAACATTTTTCTTGGGCAGTGGAGACTTTCGTTATTTTTCCTGTGTTAACC

CACATTGTCTCTTACGGTGCCCTCACCACTAGCCATTTTCTTGACACGGTCGGCCTAATCACTGTGTCCACCGC

CGGGTATTGTCACGGGCGGTATGTACTGAGTAGCATCTACGCTGTCTGCGCCCTAGCTGCGCTTGTTTGCTTCG

CCATTAGGTTGGTAAAAAATTGCATGTCCTGGCGCTACTCTTGCACCAGACACACTAATTTCCTTCTGGACACT

AAGGGAAAACTCTATCGTTGGCGGTCACCCGTCATCATAGAGAAAGGGGGCAAGGTGGATGTTGGGGGTCATTT

AATTGACCTGAAGAGAGTTGTGCTTGAAGGTTCCGCGGCGACTCCTGTAACCAAGATTTCAGCGGAACAATGGG

GTCGTCTATAG

>CF-CF18-20250808-69

ATGTTGGGGAAATGCTTGACCGCGGGCTACTGCTCGCCATTGCTTTTTTTGTGGTGTATCGTGCCGTTCTGTTT

AGTTGCGTTCGTCAACGCCAACAACAGCACCAGCTCCCACTTACAGTTGATTTATAACCTAACGATATGTGAGC

TGAATGGCACAGATTGGCTGAATGAACATTTTTCTTGGGCAGTGGAGACTTTTGTTATTTTTCCTGTGTTAACT

CACATTGTCTCTTACGGTGCCCTCACCACCAGCCATTTTCTTGACACGGTCGGCCTAATCACTGTGTCCACCGC

CGGGTATTGCCACGGGCGGTATGTATTGAGTAGCATCTACGCTGTCTGCGCCCTAGCTGCGCTTGTTTGCTTCG

CCATTAGGTTGGTAAAAAATTGCATGTCCTGGCGCTACTCTTGCACCAGACATACTAATTTCCTTCTGGACACT

AAGGGAAAACTCTATCGTTGGCGGTCACCCGTCATCATAGAGAAAGGGGGCAAGGTGGATGTTGGGGGCCATTT

AATTGACCTGAAGAGAGTTGTGCTTGAAGGTTCCGCGGCGACTCCTGTAACCAAGATTTCAGCGGAACAATGGG

GTCGTCCATAG

>CF-18-20250927-2-5

ATGTTGGGGAAATGCTTGACCGCGGGCTGCTGCTCGCAATTGCTTTTTTTGTGGTGTATCGTGCCGTTCTGTTT

AGTTGCGTTCGTCAACGCCAACAACAGCAGCAGCTCCCACTTACAGTTGATTTATAACCTAACGATATGTGAGC

TGAATGGCACAGATTGGCTGAATGAACATTTTTCTTGGGCAGTGGAGACTTTCGTTATTTTTCCTGTGTTAACT

CATATTGTCTCTTACGGTGCCCTCACCACTAGCCATTTTCTTGACACGGTCGGCCTAATCACTGTGTCCACCGC

CGGGTATTATCACGGGCGGTATGTATTGAGTAGCATCTACGCTGTCTGCGCCCTAGCTGCGCTTGTTTGCTTCG

CCATTAGGTTGGTAAAAAATTGCATGTCCTGGCGCTACTCTTGCACCAGACATACTAATTTCCTTCTGGACACT

AAGGGAAAACTCTATCGTTGGCGGTCACCCGTCATCATAGAGAAAGGGGGCAAGGTGGATGTTGGGGGCCATTT

AATTGACCTGAAGAGAGTTGTGCTTGAAGGTTCCGCGGCGACTCCTGTAACCAAGATTTCAGCGGAACAATGGG

GTCGTCCATAG

>CF18-20251101-15

CCCGTCTTTTTGCCATTCTGTTGGCAATTTGAATGTTTAGATATGTTGGGGAAATGCTTGACCGCGGGTTACTGCTCATCATTGCTTTTTTTGTGGTGTATCGTGCTATCCTGTTCTGTTGCGCTCGTCAACGCCAACAGCAACACCAGCTCTCATTTACAGTTGATTTATAACCTGACGATATGTGAGCTGAATGGCACGGATTGGTTGAATAAAAGATTTGACTGGGCAGTGGAGACTTTCGTCATTTTTCCTGTTTTGACCCATATTGTCTCGTACGGTGCCCTCACCACTAGCCATTTTCTTGACACGGTCGGCCTAATCACTGTGTCCACCGCTGGGTATTACCATAAGCGGTATGTATTGAGTAGCATCTATGCTGTCTGTGCCCTAGCCGCGTTGGCTTGCTTCGCCATCAGGTTGGCGAAAAATTGCATGTCCTGGCGCTACTCATGCACCAGATACACCAACTTTCTCCTTGACACTAAAGGCAAACTCTACCGCTGGCGGTCACCCGTCATCATAGAGAAGGGGGGCAAAGTTGATGTGGGGGGTCATTTAATTGACCTCAAGAGAGTCGTGCTTGATGGTTCCGCGGCGACCCCTATAACCAAGATTTCAGCGGAACAATGGGGTCGTCCATAGACGACTTCTGCAATGACAGCACGGCCGTACAAAAGGTGCTTTGGGCGGTTTTTCA

>CF19-20251114-2

TAGCCCTGTCTTTTTGCCATTCTGTTGGCAATTTGAATGTTTAGATATGTTGGGGAAATGCTTGACCGCGGGTTACTGCTCATCATTGCTTTTTTTGTGGTGTATCGTGCTATCCTGTTCTGTTGCGCTCGTCAACGCCAACAGCAACACCAGCTCTCATTTACAGTTGATTTATAACCTGACGATATGTGAGCTGAATGGCACGGATTGGTTGGATAAAAGATTTGACTGGGCAGTAGAGACTTTCGTCATTTTTCCTGTTCTGACCCATATTGTCTCGTACGGTGCCCTCACCACTAGCCATTTTCTTGACACGGTCGGCCTAATCACTGTGTCCACCGCTGGGTATTACCATAAGCGGTATGTATTGAGTAGCATCTATGCTGTCTGTGCCCTGGCCGCGTTGGCTTGCTTCGCCATCAGGTTGGCGAAAAATTGCATGTCCTGGCGCTACTCATGCACCAGATACACCAACTTCCTCCTTGACACTAAAGGCAAACTCTACCGCTGGCGGTCACCCGTCATCATAGAGAAGGGGGGCAAAGTTGATGTGGGGGGTCATTTAATTGACCTCAAGAGAGTCGTGCTTGATGGTTCCGCGGCGACCCCTATAACCAAGATTTCAGCGGAACAATGGGGTCGTCCATAGACGACTTCTGCAATGACAGCACGGCCGTACAAAAGGTGCTTTTGGGCG

>CF-CF19-20260120-2

atgttggggagatgcttgaccgcgggctgttgctcgcaattgccttttttgtggtatatcgtgccgttctgttttgttgtgctcgtcaacgccaacaacagcagcagctcccatttacagttgatttataacctgacgatatgtgagctgagtggcacagattggctaaataaaagttttgattgggcagtggagacctttgttattttccctgtgttgactcatattgtctcctatggtgccctaaccaccagccacttccttgacacagttggcctgatcaccgtgtctaccgctgggtattaccacgggcggtatgtcctgagtagcatttacgccgtctgcgccctagctgcgttaacttgctttgtcatcaggctaacaaaaaattgcatgtcctggcgttactcatgcaccagatacactaactttcttctggacaccaagggcaaactctatcgttggcggtctcccgtcatcgtggagaaagggggtagagttgaggtcgcaggccacctgatcgacctcaagagagttgtgcttgacggttccgcggcaacccctataaccaaagtttcagcggaacaatggggtcgtccttag

>CF-CF20-20250528-109

ATGTTGGGGAGATGCTTGACCGCGGGCTGTTGCTCGCAATTGCCTTTTTTGTGGTATATCGTGCCGTTCTGTTTTGTTGTGCTCGTCAACGCCAACAACGACAGCAGCTCCCATTTACAGTTGATTTATAACCTGACGATATGTGAGCTGAATGGCACAGATTGGCTAAATAAAAGTTTTGATTGGGCCGTGGAGACCTTTGTTATTTTCCCTGTGTTGACTCATATTGTCTCCTATGGTGCCCTAACCACCAGCCATTTCCTTGACACAGTTGGCCTGGTCACCGTGTCTACCGCTGGATATTGTCACGGGCGGTATGTCCTGAGCAGCATTTACGCCGTCTGCGCCCTGGCTGCGTTAACTTGCTTTGTAATCAGGCTAACAAAAAATTGCATGTCCTGGCGTTACTCTTGCACCAGATACACTAGCTTTCTTTTGGACACCAAGGGCAAACTCTATCGTTGGCGATCTTCCGTCATCGTGGAGAAAGGGGGTAAAGTTGAGGTCGAAGGCCACCTGATCGACCTCAAGAGAGTTGTGCTTGATGGTTCCGCGGCGACCCCTGTAACCAAAATTTCAGCGGAACAATGGGGTCGTCCTTAG

>CF-20-20250801-58

ATGTTGGGGAGATGCTTGACCGCGGGCTGTTGCTCGCAATTGCTTTTTTTGTGGTATATCGTGCCGTTCTGTTTTGTTGCGCTCGTCAACGCCAACAACGGCAGCAGCTCCCATTTACAGTTGATTTATAACCTGACGATATGTGAGCTGAATGGCACAGATTGGCTAAATAAAAGTTTCGATTGGGCAGTGGAGACCTTTGTTATTTTCCCTGTGTTGACTCATATTGTCTCCTATGGCGCCCTAACCACCAGCCATTTCCTTGACACAGTTGGCCTGATCACCGTGTCTACCGCTGGATATTATCACGGGCGGTATGTCCTGAGCAGCATTTACGCCGTCTGCGCCCTAGCTGCGTTGACTTGCTTTGTCATCAGGCTAACAAAAAATTGCATGTCCTGGCGTTACTCATGCACCAGACACACTAACTTTCTTCTGGACACCAAGGGCAAACTCTATCGTTGGCGGTCTCCCGTCATCGTAGAGAAAGGGGGTAAAGTTGAGGTCGAAGGCCACCTGATCGACCTCAAGAGAGTTGTGCTTGATGGTTCCGCGGCGACCCCTATAACCAAAGTTTCAGCGGAACAATGGGGTCGTCCTTAG

>CF-20-20250801-59

ATGTTGGAGAAATGCTTGACCGCGGGCTGTTGCTCGCAATTGCTTTCTTTGTGGTGTATCGTGCCGTTCTGTTTTGCTGTGCCCGCCAACGCCAGCAACGACAGCAGCTCCCATCTACAGCTGATTTACAACTTGACGCTATGTGAGCTGAATGGCACAGATTGGCTAGCTAACAAATTTGATTGGGCAGTGGAGAGTTTTGTCATCTTTCCCGTTTTGACTCACATTGTCTCCTATGGTGCCCTCACTACCAGCCATTTCCTTGACACAGTCGCTTTAGTCACTGTGTCTACCGCCGGGTTTGTTCACGGGCGGTATGTCCTAAGTAGCATCTACGCGGTCTGTGCCCTGGCTGCGTTGACTTGCTTCGTCATTAGGTTTGCAAAGAATTGCATGTCCTGGCGCTACGCGTGTACCAGATATACCAACTTTCTTCTGGACACTAAGGGCAGACTCTATCGTTGGCGGTCGCCTGTCATCATAGAGAAAAGGGGCAAAGTTGAGGTCGAAGGTCATCTGATCGACCTCAAAAGAGTTGTGCTTGATGGTTCCGTGGCAACCCCTATAACCAGAGTTTCAGCGGAACAATGGGGTCGTCCTTAG

>CF-20-20250801-60

ATGTTGGGGAAATGCTTGACCGCGGGCTGCTGCTCGCAATTGCTTTTTTTGTGGTGTATCGTGCCGTTCTGTTTAGTTGCGTTCGTCAACGCCAACAACAGCAGCAGCTCCCACTTACAGTTGATTTATAACCTAACGATATGTGAGCTGAATGGCACAGATTGGCTGAATGAACATTTTTCTTGGGCAGTGGAGACTTTCGTTATTTTTCCTGTGTTAACTCACATTGTCTCTTACGGTGCTCTCACCACTAGCCATTTTCTTGACACGGTCGGCCTAATCACTGTGTCCACCGCCGGGTATTGTCACGGGCGGTATGTACTGAGTAGCATCTACGCTGTCTGCGCCCTAGCTGCACTTGTTTGCTTCGCCATTAGGTTGGTAAAAAATTGCATGTCCTGGCGCTACTCTTGCACCAGATATACTAATTTCCTTCTGGACACTAAGGGAAAACTCTATCGTTGGCGGTCACCCGTCATCATAGAGAAAGGGGGCAAGGTGGATGTTGGGGGTCATTTAATTGACCTGAAGAGAGTTGTGCTTGAAGGTTCCGCGGCGACTCCTGTAACCAAGATTTCAGCGGAACAATGGGGTCGTCCATAG

>CF-20-20250801-61

ATGTTGGGGAAATGCTTGACCGCGGGCTGCTGCTCGCAATTGCTTTTTTTGTGGTGTATCGTGCCGTTCTGTTTAGTTGCGTTCGTCAACGCCAACAACAGCAGCAGCTCCCACTTACAGTCGATTTATAACCTAACGATATGTGAGCTGAATGGCACAGATTGGCTGAATGAACATTTTTCTTGGGCAGTGGAGACTTTCGTTATTTTTCCTGTGTTAACTCACATTGTCTCTTACGGTGCCCTCACCACTAGCCATTTTCTTGACACGGTCGGCCTAATCACTGTGTCCACCGCCGGGTATCATCACAGGCGGTATGTACTGAGTAGCATCTACGCTGTCTGCGCCCTAGCTGCGCTTGTTTGCTTCGCCATTAGGTTGGTGAAAAATTGCATGTCCTGGCGCTACTCTTGCACCAGATATACTAACTTCCTTCTGGACACTAAGGGAAAACTCTATCGTTGGCGGTCACCCGTCATCATAGAGAAAGGGGGCAAGGTGGATGTTGGGGGTCATTTAATTGACCTGAAGAGAGTTGTGCTTGAAGGTTCCGCGGCGACTCCTGTAACCAAGATTTCAGCGGAACAATGGGGTCGTCCATAG

>CF-20-20250801-62

TGTTTTAGGCCCGTCTTTTTGCCATTCTGCTGGCAATTTGAATGTTCAGGTATGTTGGGGAGATGCTTGACCGCGGGCTGTTGCTCGCAATTGCCTTTTTTGTGGTATATCGTGCCGTTCTGTTTTGTTGCGCTCGTCAACGCCAACAACAGCAGCAGCTCCCATTTACAGTTGATTTATAACCTGACGATATGTGAGCTGAATGGCACAGATTGGCTAAATAAAAGTTTTGATTGGGCAGTGGAGACCTTTGTTATTTTCCCTGTGTTGACTCATATTGTCTCCTATGGCGCCCTAACCACCAGCCATTTCCTTGACACAGTTGGCCTGATCACCGTGTCTACCGCTGGGTATTACCACGGGCGGTATGTCCTGAGTAGCATTTACGCCGTCTGCGCCCTAGCTGCGTTAACTTGCTTTGTCATCAGGCTAACAAAAAATTGCATGTCCTGGCGTTACTCATGCACCAGATACACTAACTTTCTTCTGGACACCAAGGGCAAACTCTATCGTTGGCGGTCTCCCGTCATCGTGGAGAAAGGGGGTAAAGTTGAGGTCGCAGGCCACCTGATCGACCTCAAGAGAGTTGTGCTTGACGGTTCCGCGGCAACCCCTATAACCAAAGTTTCAGCGGAACAATGGGGTCGTCCTTAGACGACTTCTGCTATGACAGCACAGCCCCGCTAAAGGTGCTTGGGG

>CF-CF20-20260120-1

atgttggggagatgcttgaccgcgggctgttgctcgcaattgccttttttgtggtatatcgtgccgttctgttttgttgtgctcgtcaacgccaacaacagcagcagctcccatttacagttgatttataacctgacgatatgtgagctgaatggcacagattggctaaataaaagttttgattgggcagtggagacctttgttattttccctgtgttgactcatattgtctcctatggtgccctaaccaccagccacttccttgacacagttggcctgatcaccgtgtctattgctgggtattaccacgggcggtatgtcctgagtagcatttacgccgtctgcgccctagctgcgttaacttgctttgtcatcaggctaacaaaaaattgcatgtcctggcgttactcatgcaccagatacactaactttcttctggacaccaagggcaaactctatcgttggcggtctcccgtcatcgtggagaaagggggtagagttgaggtcgcaggccacctgatcgacctcaagagagttgtgcttgacggttccgcggcaacccctataaccaaagtttcagcggaacaatggggtcgtccttag

>CF-CF20-20260120-2

atgttggggagatgcttgaccgcgggctgttgctcgcaattgccttttttgtggtatatcgtgccgttctgttttgttgtgctcgtcaacgccaacaacagcagcagctcccatttacagttgatttataacctgacgatatgtgagctgaatggcacagattggctaaataaaagttttgattgggcagtggagacctttgttattttccctgtgttgactcatattgtctcctatggtgccctaaccaccagccatttccttgacacagttggcctgatcaccgtgtctaccgctgggtattaccacgggcggtatgtcctgagtagcatttacgccgtctgcgccctagctgcgttaacttgctttgtcatcaggctaacaaaaaattgcatgtcctggcgttactcatgcaccagatacactaactttcttctggacaccaaaggcaaactctatcgttggcggtctcccgtcatcgtggagaaagggggtagagttgaggtcgcaggccacctgatcgacctcaagagagttgtgcttgacggttccgcggcaacccctataaccaaagtttcagcggaacaatggggtcgtccttag

>CF-CF20-20260120-3

atgttggggagatgcttgaccgcgggctgttgctcgcaattgccttttttgtggtatatcgtgccgttctgttttgttgtgctcgtcaacgccaacaacagcagcagctcccatttacagttgatttataacctgacgatatgtgagctgaatggcacagattggctaaataaaagttttgattgggcagtggagacctttgttattttccctgtgttgactcatattgtctcctatggtgccctaaccaccagccatttccttgacacagttggcctgatcaccgtgtctaccgctgggtattaccacgggcggtatgtcctgagtagcatttacgccgtctgcgccctagctgcgttaacttgctttgtcatcaggctaacaaaaaattgcatgtcctggcgttactcatgcaccagatacactaactttcttctggacaccaagggcaaactctatcgttggcggtctcccgtcatcgtggagaaagggggtagagttgaggtcgcaggccacctgatcgacctcaagagagttgtgcttgacggttccgcggcaacccctataaccaaagtttcagcggaacaatggggtcgtccttag

>CF-CF20-20260120-4

atgttggggagatgcttgaccgcgggctgttgctcgcaattgccttttttgtggtatatcgtgccgttctgttttgttgtgctcgtcaacgccaacaacagcagcagctcccatttacagttgatttataacctgacgatatgtgagctgaatggcacagattggctaaataaaagttttgattgggcagtggagacctttgttattttccctgtgttgactcatattgtctcctatggtgccctaaccaccagccatttccttgacacagttggcctgatcaccgtgtctaccgctgggtattaccacgggcggtatgtcctgagtagcatttacgccgtctgcgccttagctgcgttaacttgctttgtcatcaggctaacaaaaaattgcatgtcctggcgttactcatgcaccagatacactaactttcttctggacaccaagggcaaactctatcgttggcggtctcccgtcatcgtggagaaagggggtagagttgaggtcgcaggccacctgatcgacctcaagagagttgtgcttgacggttccgcggcaacccctataaccaaagtttcagcggaacaatggggtcgtccttag

>CF-CF20-20260129-1

agctaaaggacgcgcttgagcatggccaacattctgcagttccctcgcttgtggacaataatacaggcttgtgacattaggcgcggcgatgcacctaacatcggcctctcacgttggcaagtgatttatagcttggtcatttatgcgctaattaacggacgtcagctggtcccaaaccgtgacaggattcttgcggctcttgcctatcacatgaaggcgaataatgtttctgagtattacgcctctgcggctgcaatactcatggacaagatcaacatggttgctgttgcttctaacgtgttgcgcagcagatggcgcatcgtggattctctccgcgccatttgtgctctggaagggtcgagctccccgctccccaaagtcgcacacaactgcgtgcatcggtgccggctacatggtgggtccctcagtgttttatcaggggcgaggctcaaatgcttccggagacggtcttcagcaccggtcgaattgaggtagatagtctaaatgctggaaaatgttgttggacttcaaggaagtccgactgatggtctggagggacaaggcgtgtgcggagttctcgtttgacgatccggtgagatacaa

>CF-CF20-20260129-2

agctaaaggacacgcttgagcacaaccaacattctgcagttccctcgcttgtggacaataatacaggcttgtgacattaggcgcggcgatgcacctaacatcggcctctcacgttggcaagtgatttatagcttggtcatttatgcgctaattaacggacgtcagctggtcccaaaccgtgacaggattcttgcggctcttgcctatcacatgaaggcgaataatgtttctgagtattacgcctctgcggctgcaatactcatggacaagatcaacatggttgctgttgcttctaacgtgttgcgcagcagatggcgcatcgtggattctctccgcgccatttgtgctctggaagggtcgagctccccgctccccaaagtcgcacacaactgcgtgcatcggtgccggctacatggtgggtccctcagtgttttgttaggggcgaggctcaaatgcttccagagacggtcttcagcaccggtcgaattgaggtagatagtctaaatgctggaaaatgttgttggacttcaaggaagtccgactgatggtctggagggacaaggcgtgtgcggagttctcgtttgatgatccggtgagatacaa

>CF-CF20-20260129-3

agctaaaggacgcgcttgagcacaaccaacactctgcagttccctcgcttgtggataataatacaggcttgtgacattaggcgcggcgatgcacctaacatcggcctctcacgttggcaagtgatttatagcttggtcatttatgcgctaattaacgggcgtcagctggtcccaaaccgtgacaggattctcgctgctcttgcctatcacatgaaggcgaataatgtttctgagtattatgcctccgcggctgcaatactcatggacaagatcaacatggttgctgttgcttctaacgtgttgcgcagcagatggcgcatcgtggattctctccgcgccgtttgtgctctggaagggtcgagctccccgctccccaaagtcgcacacaattgcgtgcatcggtgccggctacatggtgggtccttcagttttttgttaagggcgaggctcaaatgcttccagagacggtcttcagcaccggtcgaattgaggtagatagtccaaatgctggaaaatgttgttggacttcaaggaagtccgactgatggtctggagggacaaggcgtgtgcggagttctcgtttgacgatccggtgagatacaa

>CF-CF20-20260129-4

agctaaaggacgcgcttgagcacaaccaacattctgcagttccctcacttgtggacaataatacaggcttgtgacattaggcgcggcgatgcacctaacatcggcctcccacgttggcaagtgatttatagcttggtcatttatgcgctaattaacgggcgtcagctggtcccaaaccgtgacaggattctcgctgctcttgcctatcacatgaaggcgaataatgtttctgagtattatgcctccgcggctgcaatactcatggacaagatcaacatggttgctgttgcttctaacgtgttgcgcagcagatggcgcatcgtggattctctccgcgccgtttgtgctctggaagggtcgagctccccgctccccaaagtcgcacacaattgcgtgcatcggtgccggctacatggtgggtccttcagttttttgttaagggcgaggctcaaatgcttccagagacggtcttcagcaccggtcgaattgaggtagatagtccaaatgctggaaaatgttgttggacttcaaggaagtccgactgatggtctggagggacaaggcgtgtgcggagttctcgtttgacgatccggtgagatacaa

>CF-CF20-20260129-5

atgttggggagatgcttgaccgcgggctgttgctcgcaattgccttttttgtggtatatcgtgccgttctgttttgttgtgctcgtcaacgccaacaacagcagcagctcccatttacagttgatttataacctgacgatatgtgagctgaatggcacagattggctaaataaaagttttgattgggcagtggagacctttgttattttccctgtgttgactcatattgtctcctatggtgccctaaccaccagccatttccttgacacagttggcctgatcaccgtgtctaccgctgggtattaccacgggcggtatgtcctgagtagcatttacgccgtctgcgccctagccgcgttaacttgctttgtcatcaggctaacaaaaaattgcatgtcctggcgttactcatgcaccagatacactaactttcttctggacaccaagggcaaactctatcgttggcggtctcccgtcatcgtggagaaagggggtagagttgaggtcgcaggccacctgatcgacctcaagagagttgtgcttgacggttccgcggcaacccctataaccaaagtttcagcggaacaatggggtcgtccttag

>CF-CF20-20260129-6

atgttggggaaatgcttgaccgcgggttactgctcatcattgctttttttgtggtgtatcgtgctatcctgttctgttgcgctcgtcaacgccaacaacaacaccagctctcatttacagttgatttataacctgacgatatgtgagctgaatggcacggattggttggataaaagatttgactgggcagtagagactttcgtcatttttcctgttctgacccatattgtctcgtacggtgccctcaccactagccattttcttgacacggtcggcctaatcactgtgtccaccgctgggtattgccataagcggtatgttttgagtagcatctatgctgtctgtgccctggccgcgttggcttgcttcgccatcaggttggcgaaaaattgcatgtcctggcgctactcatgcaccagatacaccaacttcctccttgacactaaaggcaaactctaccgctggcggtcacccgtcatcatagagaaagggggcaaagttgatgtggggggtcatttaattgacctcaagagagtcgtgcttgatggttccgcggcgacccctataaccaagatttcagcggaacaatggggtcgtccatag

>CF-CF20-20260129-7

atgttggggaaatgcttgaccgcgggttactgctcatcattgctttttttgtggtgtatcgtgctatcctgttctgttgcgctcgtcaacgccaacaacaacaccagctctcatttacagttgatttataacctgacgatatgtgagctgaatggcacggattggttggataaaagatttgactgggcagtagagactttcgtcatttttcctgttctgacccatattgtctcgtacggtgccctcaccactagccattttcttgacacggtcggcctaatcactgtgtccaccgctgggtattgccataagcggtatgttttgagtagcatctatgctgtctgtgccctggccgcgttggcttgcttcgccatcaggttggcgaaaaattgcatgtcctggcgctactcatgcaccagatacaccaacttcctccttgacactaaaggcaaactctaccgctggcggtcacccgtcatcatagagaaagggggcaaagttgatgtggggggtcatttaattgacctcaagagagtcgtgcttgatggttccgcggcgacccctataaccaagatttcagcggaacaatggggtcgtccatag

>CF-CF20-20260129-8

atgttggggagatgcttgaccgcgggctgttactcgcaattgccttttttgtggtatatcgtgccgttctgttttgttgcgctcgtcaacgccaacaacagcagcagctcccatttacagttgatttataacctgacgatatgtgagctgaatggcacagattggctaaataaaagttttgattgggcagtggagacctttgttattttccctgtgttgactcatattgtctcctatggcgccctaaccaccagccatttccttgacacagttggcctgatcaccgtgtctaccgctgggtattaccacgggcggtatgtcctgagtagcatttacgccgtctgcgccctagctgcgttaacttgctttgtcatcaggctaacaaaaaattgcatgtcctggcgttactcatgcaccagatacactaactttcttctggacaccaagggcaaactctatcgttggcggtctcccgtcatcgtggagaaagggggtaaagttgaggtctcaggccacctgatcgacctcaagagagttgtgcttgacggttccgcggcaacccctataaccaaagtttcagcggaacaatggggtcgtccttag

>CF-CF20-20260130-1

atgttggggaaatgcttgaccgcgggttactgctcatcattgctttttttgtggtgtatcgtgctatcctgttctgttgcgctcgtcaacgccaacagcaacaccagctctcatttacagttgatttataacctgacgatatgtgagctgaatggcacggattggttggataaaagatttgactgggcagtggagactttcgtcatttttcctgttctgacccatattgtctcgtacggtgccctcaccactagccattttcttgacacggtcggcctaatcactgtgtccaccgctgggtattgccataagcggtatgtattgagtagcatctatgctgtctgtgccctagccgcgttggcttgcttcgccatcaggttggcgaaaaattgcatgtcctggcgctactcatgcaccagatacaccaactttctccttgacactaaaggcaaactctaccgctggcggtcacccgtcatcatagagaaggggggcaaagttgatgtggggggtcatttaattgacctcaagagagtcgtgcttgatggttccgcggcgacccctataaccaagatttcagcggaacaatggggtcgtccatag

>CF-CF20-20260130-2

atgttggggagatgcttgaccgcgggctgttgctcgcaattgccttttttgtggtatatcgtgccgttctgttttgttgtgctcgtcaacgccaacaacagcagcagctcccatttacagttgatctataacctgacgatatgtgagctgaatggcacagattggctaaataaaagttttgattgggcagtggagacctttgttattttccctgtgttgactcatattgtctcctatggtgccctaaccaccagccatttccttgacacagttggcctgatcaccgtgtctaccgctgggtattaccacgggcggtatgtcctgagtagcatttacgccgtctgcgccctagccgcgttaacttgctttgtcatcaggctaacaaaaaattgcatgtcctggcgttactcatgcaccagatacactaactttcttctggacaccaagggcaaactctatcgttggcggtctcccgtcatcgtggagaaagggggtagagttgaggtcgcaggccacctgatcgacctcaagagagttgtgcttgacggttccgcggcaacccctataaccaaagtttcagcggaacaatggggtcgtccttag

>CF-CF20-20260130-3

atgttggggagatgcttgaccgcgggctgttgctcgcaattgccttttttgtggtatatcgtgccgttctgttttgttgtgctcgtcaacgccaacaacagcagcagctcccatttacagttgatttataacctgacgatatgtgagctgaatggcacagattggctaaataaaagttttgattgggcagtggagacctttgttattttccctgtgttgactcatattgtctcctatggtgccctaaccaccagccatttccttgacacagttggcctgatcaccgtgtctaccgctgggtattaccacgggcggtatgtcctgagtagcatttacgccgtctgcgccctagctgcgttaacttgctttgtcatcaggctaacaaaaaattgcatgtcctggcgttactcatgcaccagatacactaactttcttctggacaccaagggcaaactctatcgttggcggtctcccgtcatcgtggagaaagggggtagagttgaggtcgcaggccacctgatcgacctcaagagagttgtgcttgacggttccgcggcaacccctataaccaaagtttcagcggaacaatggggtcgtccttag

>CF-CF21-20250811-75

ATGTTGGGGAAATGCTTGACCGCGGGCTGCTGCTCGCAATTGCTTTTTTTGTGGTGTATCGTGCCGTTCTGTTT

AGTTGCGTTCGTCAACGCCAACAACAGCAGCAGCTCCCACTTACAGTTGATTTATAACCTAACGATATGTGAGC

TGAATGGCACAGATTGGCTGAATGAACATTTTTCTTGGGCAGTGGAGACTTTCGTTATTTTTCCTGTGTTAACT

CACATTGTCTCTTACGGTGCCCTCACCACTAGCCATTTTCTTGACACGGTCGGCCTAATCACTGTGTCCACCGC

CGGGTATTGTCGCGGGCGGTATGTATTGAGTAGCATCTACGCTGTCTGCGCCCTAGCTGCACTTGTTTGCTTCG

CCATTAGGTTGGTAAAAAATTGCATGTCCTGGCGCTACTCTTGCACCAGACATACTAATTTCCTTCTGGACACT

AAGGGAAAACTCTATCGTTGGCGGTCACCCGTCATCATAGAGAAAGGGGGCAAGGTGGATGTTGGGGGCCATTT

AATTGACCTGAAGAGAGTTGTGCTTGAAGGTTCCGCGGCGACTCCTGTAACCAAGATTTCAGCGGAACAATGGG

GTCGTCCATAG

>CF-21-20250906

ATGTTGGGGAAATGCTTGACCGCGGGCTGCTGCTCGCAATTGCCTTCTTTGTGGTGTATCGTGCCGTTCTGTTT

AGTTGCGCTCGTCAACGCCAACCACGACAGCAGCTCCCACTTACAATTGATTTATAACCTAACGATATGTGAGC

TGAATGGCACAGATTGGCTGAAAGAACACTTTCCTTGGGCGGTGGAGACTTTCGTTATTTTTCCTGTGTTAACT

CATGTTGTCTCTTACGGTGCCCTCACCACTAGCCATTTTCTTGACACGGTCGGTCTTATCACTGTGTCCACCGC

CGGGTATTATCACGGGCGGTATGTACTGAGTAGCATCTACGCTGTCTGCGCCCTAGCTGCGCTTGCTTGCTTCG

CCATTAGGTTGGTAAAGAATTGCATGTCCTGGCGCTACTCTTGCACCAGATATACTAATTTTCTTCTGGACACC

AAGGGAAAACTCTATCGTTGGCGGTCACCCGTCATCATAGAGAAGGGGGGCAAGGTGGATGTTGGGGGTCATTT

AATCGACCTAAAGAGAGTTGTGCTTGAAGGTTCCGCGGCAACCCCTGTAACCAAGATTTCAGCGGAACAATGGG

GTCGTCCATAG

>CF-21-20251024-35

TGTTTTAGCCTGTCTTTTTGCCATTCTGTTGGCAATTTGAATGTTTAGATATGTTGGGGAAATGCTTGACCGCGGGTTATTGCTCACAATTGCTTTTTTTGTGGTGTATCGTGCCATTCTGTTCTGCTGTGCTCGTCAACGCCAGCGGCAACAGCAGCTCCCACTTACAGTTGATCTATAACCTGACGATATGTGAGCTGAATGGCACAGACTGGTTGAACCAAAAATTTGACTGGGCAGTAGAGACTTTCGTCATTTTTCCTGTCTTGACCCATATTGTCTCTTACGGCGCCCTTACCACTAGCCATTTTCTTGACACGGTCGGCCTAATCACCGTATCCACCGCTGGGTATTATCATAAGCGGTATGTGTTGAGTAGCATCTACGCTGTCTGTGCCCTGGCCGCGTTGGTTTGCTTCGCCATTAGGTTGGCAAAAAATTGCATGTCCTGGCGCTACTCATTCACCAGATATACTAATTTTCTTCTGGACTCTAAGGGCAAACTCTACCGTTGGCGGTCACCCGTCATCATAGAGAAAGAGGGCAAAGTTGATGTGGGGGGTCAGTTAATCGACCTAAAGAGAGTTGTGCTTGAAGGTTCCGCGGCAACCCCTGTAACCAAGATTTCAGCGGAACAATGGGGTCGTCCATAGACGACTTCTGCAATGACAGCACGGCTGTACAAAAGGTGCTTTTGGCGTTTTCA

>CF-CF22-20250512-76

ATGTTGGGGAAATGCTTGACCGCGGGCTGCTGCTCGCAATCGCTTTTTTTGTGGTGTATCGTGCCGTTCTGTTTAGTTGCGTTCGTCAACGCCAACAACAGCAGCAGCTCCCACTTACAGTTGATTTATGACCTAACGATATGTGAGCTGAATGGCACAGATTGGCTGGATAAACATTTTTCTTGGGCAGTGGAGACTTTCGTTATTTTTCCTGTATTAACTCACATTGTCTCTTACGGTGCCCTCACCACTAGCCATTTTCTTGACACGGTCGGCCTAATCACTGTGTCCACCGCCGGGTATTGTCACGGGCGGTATGTACTGAGTAGCATCTACGCTGTCTGCGCCCTAGCTGCGCTTGTTTGCTTCGCCATTAGGTTGGTAAAAAATTGCATGTCCTGGCGCTACTCTTGCACTAGATATACTAATTTCCTTCTGGACACTAAGGGAAAACTCTATCGTTGGCGGTCACCCGTCATCATAGAGAAAGGGGGCAAGGTGGATGTTGGGGGTCATTTAATTGACCTGAAGAGAGTTGTGCTTGAAGGTTCCGCGGCGACCCCTGTAACCAAGATTTCAGCGGAACAATGGGGTCGTCCATAG

>CF-CF22-20250512-78

ATGTTGGGGAAATGCTTGACCGCGGGCTGCTGCTCGCAATCGCTTTTTTTGTGGTGTATCGTGCCGTTCTGTTTAGTTGCGTTCGTCAACGCCAACAACAGCAGCAGCTCCCACTTACAGTTGATTTATAACCTAACGATATGTGAGCTGAATGGCACAGATTGGCTGAATAAACATTTTTCTTGGGCAGTGGAGACTTTCGTTATTTTTCCTGTGTTAACTCACATTGTCTCTTACGGTGCCCTCACCACTAGCCATTTTCTTGACACGGTCGGCCTAATCACTGTGTCCACCGCCGGGTATTGTCACGGGCGGTATGTACTGAGTAGCATCTACGCTGTCTGCGCCCTAGCTGCGCTTGTTTGCTTCGCCATTAGGTTGGTAAAAAATTGCATGTCCTGGCGCTACTCTTGCACTAGATATACTAATTTCCTTCTGGACACTAAGGGAAAACTCTATCGTTGGCGGTCACCCGTCATCATAGAGAAAGGGGGCAAGGTGGATGTTGGGGGTCATTTAATTGACCTGAAGAGAGTTGTGCTTGAAGGTTCCGCGGCGACCCCTGTAACCAAGATTTCAGCGGAACAATGGGGTCGTCCATAG

>CF-22-20250727

ATGTTGGGGAGATGCTTGACCGCGGGCTGTTGCTCGCAATTGCCTTTTTTGTGGTATATCGTGCCGTTCTGTTTTGTTGTGCTCGTCAACGCCAACAACAGCAGCAGCTCCCATTTACAGTTGATTTATAACCTGACGATATGTGAGCTGAATGGCACAGATTGGCTAAATAAAAGTTTTGATTGGGCAGTGGAGACCTTTGTTATTTTCCCTGTGTTGACTCATATTGTCTCCCATGGCGCCCTAACCACCAGCCATTTCCTTGACACAGTTGGCCTGATCACCGTGTCTACCGCTGGGTATTACCACGGGCGGTATGTCCTGAGCAGCATTTACGCCGTCTGCGCCCTAGCTGCGTTAACTTGCTTTGTCATCAGGCTAACAAAAAATTGCATGTCCTGGCGTTACTCATGCACCAGATACACTAACTTTCTTCTGGACACCAAGGGCAAACTCTATCGTTGGCGGTCTCCCGTCATCGTGGAGAAAGGGGGTAAAGTTGAGGTCGCAGGCCACCTGATCGACCTCAAGAGAGTTGTGCTTGACGGTTCCGCGGCAACCCCTATAACCAAAGTTTCAGCGGAACAATGGGGTCGTCCTTAG

>CF-22-20251020-37

TGTTTTAGGCCTGTCTTTTTGCCATTCTGCTAGCAATTTGAATGTTCAGGTATGTTGGGGAAATGCTTGACCGCGGGCTGCTGCTCGCAATTGCTTTTTTTGTGGTGTATCGTGCCGTTCTGTTTAGTTGCGTTCGTCAACGCCAACAACAGCAGCAGCTCCCACTTACAGTTGATTTATAACCTAACGATATGTGAGCTGAATGGCACAGATTGGCTGAATGAACATTTTTCTTGGGCAGTGGAGACTTTCGTTATTTTTCCTGTGTTAACTCACATTGTCTCTTACGGTGCCCTCACCACTAGCCATTTTCTTGACATGGTCGGCCTAATCACTGTGTCCACCGCCGGGTATTGTCACGGGCGGTATGTACTGAGTAGCATCTACGCTGTCTGCGCCCTAGCTGCGCTTGTTTGCTTCGCCATTAGGTTGGTAAAAAATTGCATGTCCTGGCGCTACTCTTGCACCAGATATACTAATTTCCTTCTGGACACTAAGGGAAAACTCTATCGTTGGCGGTCACCCGTCATCATAGAGAAAGGGGGCAAGGTGGATGTTGGGGGTCATTTAATTGACCTGAAGAGAGTTGTGCTTGAAGGTTCCGCGGCGACTCCTGTAACCAAGATTTCAGCGGAACAATGGGGTCGTCCATAGACGACTTCTGCAATGACAGCACGGCTGTACAAAAGGTGCTTTTGGCGTTTTCA

>CF-CF22-20260120-1

atgttggggagatgcttgaccgcgggctgttgctcgcaattgccttttttgtggtatatcgtgccgttctgttttgttgcgctcgtcaacgccaacaacagcagcagctcccatttacagttgatttataacctgacgatatgtgagctgaatggcacagattggctaaataaaagttttgattgggcagtggagacctttgttattttccctgtgttgactcatattgtctcctatggcgccctaaccaccagccatttccttgacacagttggcctgatcaccgtgtctaccgctgggtattaccacgggcggtatgtcctgagtagcatttacgccgtctgcgccctagctgcgttaacttgctttgtcatcaggctaacaaaaaattgcatgtcctggcgttactcatgcaccagatacactaactttcttctggacaccaagggcaaactctatcgttggcggtctcccgtcatcgtggagaaagggggtaaagttgaggtcgcaggccacctgatcgacctcaagagagttgtgcttgacggttccgcggcaacccctataaccaaagtttcagcggaacaatggggtcatccttag

>CF-CF22-20260120-2

atgttggggagatgcttgaccgcgggctgttgctcgcaattgccttttttgtggtatatcgtgccgttctgttttgttgcgctcgtcaacgccaacaacagcagcagctcccatttacagttgatttataacctgacgatatgtgagctgaatggcacagattggctaaataaaagttttgattgggcagtggagacctttgttattttccctgtgttgactcatattgtctcctatggcgccctaaccaccagccatttccttgacacagttggcctgatcaccgtgtctaccgctgggtattaccacgggcggtatgtcctgagtagcatttacgccgtctgcgccctagctgcgttaacttgctttgtcatcaggctaacaaaaaattgcatgtcctggcgttactcatgcaccagatacactaactttcttctggacaccaagggcaaactctatcgttggcggtctcccgtcatcgtggagaaagggggtaaagttgaggtcgcaggccacctgatcgacctcaagagagttgtgcttgacggttccgcggcaacccctataaccaaagtttcagcggaacaatggggtcgtccttag

>CF-CF22-20260120-3

atgttggggagatgcttgaccgcgggctgttgctcgcaattgccttttttgtggtatatcgtgccgttctgttttgttgcgctcgtcaacgccaacaacagcagcagctcccatttacagttgatttataacctgacgatatgtgagctgaatggcacagattggctaaataaaagttttgattgggcagtggagacctttgttattttccctgtgttgactcatattgtctcctatggcgccctaaccaccagccatttccttgacacagttggcctgatcaccgtgtctaccgctgggtattaccacgggcggtatgtcctgagtagcatttacgccgtctgcgccctagctgcgttaacttgctttgtcatcaggctaacaaaaaattgcatgtcctggcgttactcatgcaccagatacactaactttcttctggacaccaagggcaaactctatcgttggcggtctcccgtcatcgtggagaaagggggtaaagttgaggtcgcaggccacctgatcgacctcaagagagttgtgcttgacggttccgcggcaacccctataaccaaagtttcagcggaacaatggggtcgtccttag

>CF-CF22-20260120-4

atgttggggagatgcttgaccgcgggctgttactcgcaattgccttttttgtggtatatcgtgccgttctgttttgttgtgctcgtcaacgccaacaacagcagcagctcccatttacagttgatttataacctgacgatatgtgagctgaatggcacagattggctaaataaaagttttgattgggcagtggagacctttgttattttccctgtgttgactcatattgtctcctatggcgccctaaccaccagccatttccttgacacagttggcctgatcaccgtgtctaccgctgggtattaccacgggcggtatgtcctgagtagcatttacgccgtctgcgccctagctgcgttaacttgctttgtcatcaggctaacaaaaaattgcatgtcctggcgttactcatgcaccagatacactaactttcttctggacaccaagggcaaactctatcgttggcggtctcccgtcatcgtggagaaagggggtaaagttgaggtcgcaggccacctgatcgacctcaagagagttgtgcttgacggttccgcggcaacccctataaccaaagtttcagcggaacaatggggtcgtccttag

>CF-CF22-20260120-5

atgttggggaaatgcttgaccgcgggttactgctcatcattgctttttttgtggtgtatcgtgctatcctgttctgttgcgctcgtcaacgccaacagcaacaccagctctcatttacagttgatttataacctgacgatatgtgagctgaatggcacggattggttgaataaaagatttgactgggcagtggagactttcgtcatttttcctgttctaacccatattgtctcgtacggtgccctcaccactagccattttcttgacacggtcggcctaatcactgtgtccaccgctgggtattaccataagcggtatgtattgagtagcatctatgctgtctgtgccctagccgcgttggcttgcttcgccatcaggttggcgaaaaattgcatgtcctggcgctactcatgcaccagatacaccaactttctccttgacactaaaggcaaactctaccgctggcggtcacccgtcatcatagagaaggggagcaaagttgatgtggggggtcatttgattgacctcaagagagtcgtgcttgatggttccgcggcgacccctataaccaagatttcagcggaacaatggggtcgtccttag

>CF-CF22-20260120-6

atgttggggaaatgcttgaccgcgggttactgctcatcattgctttttttgtggtgtatcgtgctatcctgttctgttgcgctcgtcaacgccaacagcaacaccagctctcatttacagttgatttataacctgacgatatgtgagctgaatggcacggattggttgaataaaagatttgactgggcagtggagactttcgtcatttttcctgttctaacccatattgtctcgtacggtgccctcaccactagccattttcttgacacggtcggcctaatcactgtgtccaccgctgggtattaccataagcggtatgtattgagtagcatctatgctgtctgtgccctagccgcgttggcttgcttcgccatcaggttggcgaaaaattgcatgtcctggcgctactcatgcaccagatacaccaactttctccttgacactaaaggcaaactctaccgctggcggtcacccgtcatcatagagaaggggggcaaagttgatgtggggggtcatttgattgacctcaagagagtcgtgcttgatggttccgcggcgacccctataaccaagatttcagcggaacaatggggtcgtccatag

>CF-CF23-20250718-35

ATGTTGGGGAAGTGCTTGACCGCGTGCTGTTGCTCGCGATTGCTTTTTTTGTGGTGTATCGTGCCGTCCTATCTTGCTGTGCTCGTCAACGCCAGCAACAACAACAGCTCTCATATTCAGTTGATTTATAACTTAACGCTATGTGAGCTGAATGGCACAGATTGGCTGGCACAAAAATTTGACTGGACAGTGGAGACTTTCGTCATCTTCCCCGTGTTGACTCACATTGTTTCCTATGCGGCACTCACCACCAGCCATTTCCTTGACACAGTTGGTCTGGCCACTGTGTCCACCGCCGGATATTATCACGGGCGGTATGTCTTGAGTAGCATTTACGCAGTCTGTGCTCTGGCTGCGCTGATTTGCTTTGTCATTAGGCTTGCGAAGAACTGCATGTCCTGGCGCTACTCTTGTACCAGATATACCAACTTCCTTCTGGACACTAAGGGCAAACTCTATCGTTGGCGGTCGCCCGTCATTGTGGAGAAAGGGGGTAAGGTTGAGGTCGAAGGTCACCTGATCGACCTCAAGAGAGTTGTGCTTGATGGTTCCGCGGCAACCCCTTTAACCAGAGTTTCAGCGGAACGATGGGGTCGTCTCTAG

>CF-23-20250803-65

ATGTTGGGGAAATGCTTGACCGCGGGCTGCTGCTCGCAATTGCCTTTTTTGTGGTGTATCGTGCCGTTCTGTTTAGTTGCGCTCGTCAACGCCAACGACAGCAGCAGCTCCCACTTACAATTGATTTATAACCTAACGATATGTGAGCTGAATGGCACAGATTGGCTGAAAGAACACTTTCCTTGGGCGGTGGAGACTTTCGTTATTTTTCCTGTGTTAACTCATGTTGTCTCTTACGGTGCCCTCACCACTAGCCATTTTCTTGACACGGTCGGTCTTATCACTGTGTCCACCGCCGGGTATTATCACGGGCGGTATGTATTGAGTAGCATCTACGCTGTCTGCGCCCTAGCTGCGCTTGTCTGCTTTGCCATTAGGTTGGTAAAGAACTGCATGTCCTGGCGCTACTCTTGCACCAGATATACTAATTTTCTTCTGGACACTAAGGGAAAACTCTATCGTTGGCGGTCACCCGTCATCATAGAGAAGGGGGGCAAGGTGGACGTTGGGGGTCATTTAATCGACCTAAAGAGAGTTGTGCTTGAAGGTTCCGCGGCAGCCCCTGTAACCAAGATTTCAGCGGAACAATGGGGTCGTCCATAG

>CF-23-20250928-34

ATGTTGGGGAAATGCTTGACCGCGGGCTGCTGCTCGCAATTGCTTTTTTTGTGGTGTATCGTGCCGTTCTGTTT

AGTTTCGCTCGTCAACGCCAGCAACAGCAGCAGCTCCCATTTACAGTTGATTTATAACCTGACGATATGTGAGC

TGAATGGCACAGATTGGCTAAATCGCACTTTTGATTGGGCAGTGGAGACCTTCGTTATTTTTCCTGTGTTGACT

CATATCGTCTCCTATGGCGCCCTAACCACCAGCCACTTCCTTGACACAGTCGGCCTGATCACCGTGTCTACTGC

TGGATATTACCACGGGCGGTATGTCTTGAGCAGTATTTACGCCGTCTGCGCCTTAGCTGCGCTAATTTGCTTCG

TCATCAGGCTAACAAAAAATTGCATGTCCTGGCGTTACTCATGCACCAGATACACTAATTTTCTTCTGGACACC

AAGGGAAAACTCTATCGTTGGCGGTCTCCCGTCATCGTGGAAAAAGGGGGTAAAGTTGAGGTCGAAGGCCACCT

CATCGACCTCAAGAAAGTTGTGCTTGATGGTTCCGCGGCGACCCCTATAACCAAAGTTATAGCGGAACAATGGG

GTCGTCCTTAG

>CF-23-20251028-26

TGTTTTAGCCTGTCTTTTTGCCATTCTGCTGGCAATTTGAATGTTCAGGTATGTTGGGGAAATGCTTGACCGCGGGCTGCTGCTCGCAATTGCCTTTTTTGTGGTGTATCGTGCCGTTCTGTTTAGTTGCGCTCGTCAACGCCAACAACGACAGCAGCTCCCACTTACAATTGATTTATAACCTAACGATATGTGAGCTGAATGGCACAGATTGGCTGAAAGAACACTTTTCTTGGGCGGTGGAGACTTTCGTTATTTTTCCTGTGTTGACTCATATCGTCTCCTATGGCGCCCTAACCACCAGCCACTTCCTTGACACAGTCGGCCTGATCACCGTGTCTACTGCTGGATATTACCACGGGCGGTATGTCTTGAGCAGTATTTACGCCGTCTGCGCCTTAGCTGCGCTAATTTGCTTCGTCATCAGGCTAACAAAAAATTGCATGTCCTGGCGCTACTCTTGCACCAGATATACTAATTTTCTTCTGGACACTAAGGGAAAACTCTATCGCTGGCGGTCACCCGTCATCATAGAGAAGGAGGGCAAGGTGGACGTTGGGGGTCATTTAATCGACTTAAAGAGAGTTGTGCTTGAAGGTTCCGCGGCAACCCCTGTAACCAAGATTTCAGCGGAACAATGGGGCCGTCCATAGACGACTTCTGCAATGACAGCACGGCTGTACAAAAGGTGCTTTTGGCGTTTTCA

>CF-23-20251025-27

TGTTTTAGGCCTGTCTTTTTGCCATTCTGCTGGCAATTTGAATGTTCAGGTATGTTGGGGAAATGCTTGACCGCGGGCTGCTGCTCGCAATTGCCTTTTTTGTGGTGTATCGTGCCGTTCTGTTTAGTTGCGCTCGTCAACGCCAACGACAACAGCAGCTCCCACTTACAATTGATTTATAACCTAACGATATGTGAGCTGAATGGCACAGATTGGCTGAAAGACCACTTTTCTTGGGCGGTGGAGACTTTCGTTATTTTTCCTGTGTTAACTCATATTGTCTCTTACGGTGCCCTCACCACTAGCCATTTTCTTGACACGGTCGGTCTTATCACTGTGTCCACCGCCGGGTATTATCACGGGCGGTATGTATTGAGTAGCATTTACGCTGTCTGCGCCTTAGCTGCGCTTGTTTGCTTCGCCATTAGGTTGGTAAAGAATTGCATGTCCTGGCGCTACTCTTGCACCAGATATACCAATTTTCTTCTGGACACTAAGGGAAAACTCTATCGTTGGCGGTCATCCGTCATCATAGAGAAGGGGGGCAAGGTGGATGTTGGTGGTCATTTAATCGACCTAAAGAGAGTTGTGCTTGAAGGTTCCGCGGCAACCCCTGTAACCAAGATTTCAGCGGAACAATGGGGCCGTCCATAGACGACTTCTGCAATGACAGCACGGCTGTACAAAAGGTGCTTTGGCC

>CF-CF23-20260130-3

atgttggggagatgcttgaccgcgggttgctgctcatcattgctttttttgtggtgtatcgtgctatcttgttctgttgcgctcgtcaacgccgacagcaacaccagctctcatttacagttgatttataacctgacgatatgtgagctgaatggcacggattggttggacaaaagatttaactgggcagtagagactttcgtcatttttcctgttctaacccatattgtctcttacggcgccctcaccactagccattttcttgacacggtcggcctaattactgtgtccaccgctgggtattaccataagcggtatgtattgagtagcatctatgctgtctgtgccctggccgcgttggcttgcttcgccattaggttggcgaaaaattgcatgtcttggcgctactcatgcaccagatacaccaacttcctcctggacactaaaggcagactctaccgctggcggtcacccgtcatcatagagaaggggggcaaagttgatgtggggggtcatttaattgacctcaaaagagtcgtgcttgatggttccgcggcgacccctataaccaagatatcagcggaacaatggggtcgtccatag

>CF-CF24-20250721-39

ATGTTGGGGAAGTGCTTGACCGCGTGCTGTTGCTCGCGATTGCTTTTTTTGTGGTGTATCGTGCCGTCCTATCTTGCTGTGCTCGTCAACGCCAGCAACAACAACAGCTCTCATATTCAGTTGATTTATAACTTAACGCTATGTGAGCTGAATGGCACAGATTGGCTGGCACAAAAATTTGACTGGACAGTGGAGACTTTCGTCATCTTCCCCGTGTTGACTCACATTGTTTCCTATGCGGCACTCACCACCAGCCATTTCCTTGACACAGTTGGTCTGGCCACTGTGTCCACCGCCGGATATTATCACGGGCGGTATGTCTTGAGTAGCATTTACGCAGTCTGTGCTCTGGCTGCGCTGATTTGCTTTGTCATTAGGCTTGCGAAGAACTGCATGTCCTGGCGCTACTCTTGTACCAGATATACCAACTTCCTTCTGGACACTAAGGGCAAACTCTATCGTTGGCGGTCGCCCGTCGTTGTGGAGAAAGGGGGTAAGGTTGAGGTCGAAGGTCACCTGATCGACCTCAAGAGAGTTGTGCTTGATGGTTCCGCGGCAACCCCTTTAACCAGAGTTTCAGCGGAACGATGGGGTCGTCTCTAG

>CF-CF24-20250721-43

ATGTTGGGGAAATGCTTGACCGCGGGCTGCTGCTCGCAATTACTTTTTTTGTGGTGTATCGTGCCGTTCTGTTTAGTTGCGTTCGTCAACGCCAACAACAGCAGCAGCTCCCACTTACAGTTGATTTATAACCTAACGATATGTGAGCTGAATGGCACAGATTGGCTGAAAGAACATTTTTCTTGGGCAGTGGAGACTTTCGTTATTTTTCCTGTGTTAACCCACATTGTCTCTTACGGTGCCCTCACCACTAGCCATTTTCTTGACACGGTCGGCCTAATCACTGTGTCCACCGCCGGGTATTATCACGGGCGGTATGTACTGAGTAGCATCTATGCTGTCTGCGCCCTAGCTGCGCTTGTTTGCTTCGCCATTAGGTTGGTAAAAAATTGCATGTCCTGGCGCTACTCTTGCACCAGATATACTAATTTTCTTCTGGACACTAAGGGAAAACTCTATCGTTGGCGGTCACCCGTCATCATAGAGAAAGGGGGCAAGGTGGATGTTGGGGGTCATTTAATTGACCTGAAGAGAGTTGTGCTTGAAGGTTCCGCGGCGACTCCTGTAACCAAGATTTCAGCGGAACAATGGGGACATCCATAG

>CF-24-20251020-38

CCCTTTTTGCCATTCTGCTAGCAATTTGAATGTTCAGGTATGTTGGGGAAATGCTTGACCGCGGGCTGCTGCTCGCAATTACTTTTTTTGTGGTGTATCGTGCCGTTCTGTTTAGTTGCGTTCGTCAACGCCAACAACAGCAGCAGCTCCCACTTACAGTTGATTTATAACCTAACGATATGTGAGCTGAATGGCACAGACTGGCTGAAAGAACATTTTTCTTGGGCAGTGGAGACTTTCGTTATTTTTCCTGTGTTAACCCACATTGTCTCTTACGGTGCCCTCACCACTAGCCATTTTCTTGACACGGTCGGCCTAATCACTGTGTCCACCGCCGGGTATTATCACGGGCGGTATGTACTGAGTAGCATCTATGCTGTCTGCGCCCTAGCTGCGCTTGTTTGCTTCGCCATTAGGTTGGTAAAAAATTGCATGTCCTGGCGCTACTCTTGCACCAGATATACTAATTTTCTTCTGGACACTAAGGGAAAACTCTATCGTTGGCGGTCACCCGTCATCATAGAGAAAGGGGGCAAGGTGGATGTTGGGGGTCATTTAATTGACCTGAAGAGAGTTGTGCTTGAAGGTTCCGCGGCGACTCCTGTAACCAAGATTTCAGCGGAACAATGGGGACATCCATAGACGACTTCTGCAATGACAGCACGGCTGTGCAAAAGGTGCTGGG

>CF-CF24-20260129-1

atgttggagaaatgcttgaccgcgggctgttgctcgcaattgctttctttgtggtgtatcgtgccgttctgttttgctgtgctcgccaacgccagcaacgacagcagctcccatctacagctgatttacaacttgacgctatgtgagctgaatggcacagattggctagctaacaaatttgattgggcagtggagagttttgtcatctttcccgttttgactcacattgtctcctatggtgccctcactaccagccatttccttgacacagtcgctttagtcactgtgtctaccgccgggtttgttcacgggcggtatgtcctaagtagcatctacgcggtctgtgccctggctgcgttgacttgcttcgtcattaggtttgcaaagaattgcatgtcctggcgctacgcgtgtaccagatataccaactttcttctggacactaagggcatactctatcgttggcggtcgcctgtcatcatagagaaaaggggcaaagttgaggtcgaaggtcatctgatcgacctcaaaagagttgtgcttgatggttccgtggcaacccctataaccagagtttcagcggaacaatggggtcgtccttag

>CF-CF24-20260129-2

atgttggggaaatgcttgaccgcgggctgctgctcgcaattgccttttttgtggtgtatcgtgccgttctgtttagttgcgctcgtcaacgcctacaacgacagcagctcccacttacaattgatttataacctaacgatatgtgagctgaatggcacagattggctgaaagaacacttttcttgggcggtggagactttcgttatttttcctgtgttgactcatatcgtctcctatggcgccctaaccaccagccacttccttgacacagtcggcctgatcaccgtgtctactgctggatattaccacgggcggtatgtcttgagcagcatttacgccgtctgcgccttagctgcgctaatttgcttcgtcatcaggctaacaaaaaattgcatgtcctggcgctactcttgcaccagatatactaattttcttctggacactaagggaaaactctatcgctggcggtcacccgtcatcatagagaaggagggcaaggtggacgttgggggtcatttaatcgacttaaagagagttgtgcttgaaggttccgcggcaacccctgtaaccaagatttcagcggaacaatggggccgtccatag

>CF-CF24-20260129-3

atgttggggaaatgcttgaccgcgggctgctgctcgcaattgccttttttgtggtgtatcgtgccgttctgtttagttgcgctcgtcaacgcctacaacgacagcagctcccacttacaattgatttataacctaacgatatgtgagctgaatggcacagattggctgaaagaacacttttcttgggcggtggagactttcgttatttttcctgtgttgactcatatcgtctcctatggcgccctaaccaccagccacttccttgacacagtcggcctgatcaccgtgtctactgctggatattaccacgggcggtatgtcttgagcagcatttacgccgtctgcgccttagctgcgctaatttgcttcgtcatcaggctaacaaaaaattgcatgtcctggcgctactcttgcaccagatatactaattttcttctggacactaagggaaaactctatcgctggcggtcacccgtcatcatagagaaggagggcaaggtggacgttgggggtcatttaatcgacttaaagagagttgtgcttgaaggttccgcggcaacccctgtaaccaagatttcagcggaacaatggggccgtccatag

>CF-CF24-20260129-4

atgttggggaaatgcttgaccgcgggttgctgctcacaattgccttttttgtggtgtatcgtgctatcttgttctgttgcgctcgtcaacgccaacaacgacagcagctctcattcacagttgatttataacctgacgatatgtgagctgaatggcacgaattggttggacaaaaaatttgactgggcagtagagactttcgtcatatttcctgttctaacccatattgtctcttacggcgccctcaccactagccattttcttgacacggtcggtctaatcactgtgtccaccgctgggtattaccataagcggtatgtattgagtagcatctatgctgtctgtgccctggccgcgtcggcttgcttcgccatcaggttggcg-aaaattgcatgtcctggcgctactcatgcaccagatacaccaactttctcctggacactaaaggcagactctaccgctggcggtcacccgtcatcatagagaaggggggcaaagttgatgtggggggtcatttaattgacctcaagagagtcgtgcttgatggttccgcggcgacccctataaccaagatttcagcggaacaatggggtcgtccatag

>CF-CF24-20260129-5

atgttggggagatgcttgaccgcgggttgctgctcatcattgccttttttgtggtgtatcgtgctatcttgttctgttgcgctcgtcaacgccaacagcaacaccagctctcattcacagttgatttataacctgacgatatgtgagctgaatggcacgaattggttggacaaaaaatttgactgggcagtagagactttcgtcatttttcctgttctaacccatattgtctcttacggcgccctcaccactagccattttcttgacacggtcggtctaatcactgtgtccaccgctgggtattaccataagcggtatgtattgagtagcatctatgctgtctgtgccctggccgcgtcggcttgcttcgccatcaggttggcgaaaaattgcatgtcctggcgctactcatgcaccagatacaccaactttctcctggacactaaaggcagactctaccgctggcggtcacccgtcatcatagagaaggggggcaaagttgatgtggggggtcatttaattgacctcaagagagtcgtgcttgatggttccgcggcgacccctataaccaagatttcagcggaacaatggggtcgtccatag

>CF-CF24-20260129-6

atgttggagaaatgcttgaccgcgggctgttgctcgcaattgctttctttgtggtgtatcgtgccgttctgttttgctgtgctcgccaacgccagcaacgacagcagctcccatctacagctgatttacaacttgacgctatgtgagctgaatggcacagattggctagctaacaaatttgattgggcagtggagagttttgtcatctttcccgttttgactcacattgtctcctatggtgccctcactaccagccatttccttgacacagtcgctttagtcactgtgtctaccgccgggtttgttcacgggcggtatgtcctaagtagcatctacgcggtctgtgccctggctgcgttgacttgcttcgtcattaggtttgcaaagaattgcatgtcctggcgctacgcgtgtaccagatataccaactttcttctggacactaagggcagactctatcgttggcggtcgcctgtcatcatagagaaaaggggcaaagttgaggtcgaaggtcatctgatcgacctcaaaagagttgtgcttgatggttccgtggcaacccctataaccagagtttcagcggaacaatggggtcgtccttag

>CF-CF24-20260129-7

atgttggggagatgcttgaccgcgggttgctgctcatcattgccttttttgtggtgtatcgtgctatcttgttctgttgcgctcgtcaacgccaacagcaacaccagctctcatttacagttgatttataacctgacgatatgtgagctgaatggcacgaattggttggacaaaagatttaactgggcagtagagactttcgtcatttttcctgttctaacccatattgtctcttacggcgccctcaccactagccattttcttgacacggtcggcctaattactgtgtccaccgctgggtattaccataagcggtatgtattgagtagcatctatgctgtctgtgccctggccgcgtcggcctgcttcgccatcaggttggcgaaaaattgcatgtcctggcgctactcatgcaccagatacaccaactttctcctggacactaaaggcagactctaccgctggcggtcacccgtcatcatagagaaggggggcaaagttgatgtggggggtcatttaattgacctcaagagagtcgtgcttgatggttccgcggcgacccctataaccaagatttcagcggaacaatggggtcgtccatag

>CF-CF24-20260129-8

atgttggagaaatgcttgaccgcgggctgttgctcgcaattgctt--cttgtggtgtatcgtgccgttctgttttgctgtgctcgccaacgccagcaacgacagcagctcccatctacagctgatttacaacttgacgctatgtgagctgaatggcacagattggctagctaacaaatttgattgggcagtggagagttttgtcatctttcccgttttgactcacattgtctcctatggtgccctcactaccagccatttccttgacacagtcgctttagtcactgtgtctaccgccgggtttgttcacgggcggtatgtcctaagtagcatctacgcggtctgtgccctggctgcgttgacttgcttcgtcattaggtttgcaaagaattgcatgtcctggcgctacgcgtgtaccagatataccaactttcttctggacactaagggcatactctatcgttggcggtcgcctgtcatcatagagaaaaggggcaaagttgaggtcgaaggtcatctgatcgacctcaaaagagttgtgcttgatggttccgtggcaacccctataaccagagtttcagcggaacaatggggtcgtccttag

>CF-CF24-20260129-9

atgttggggaaatgcttgaccgcgggctgctgctcgcaattgccttttttgtggtgtatcgtgccgttctgtttagttgcgctcgtcaacgcctacaacgacagcagctcccacttacaattgatttataacctaacgatatgtgagctgaatggcacagattggctgaaagaacacttttcttgggcggtggagactttcgttatttttcctgtgttgactcatatcgtctcctatggcgccctaaccaccagccacttccttgacacagtcggcctgatcaccgtgtctactgctggatattaccacgggcggtatgtcttgagcagcatttacgccgtctgcgccttagctgcgctaatttgcttcgtcatcaggctaacaaaaaattgcatgtcctggcgctactcttgcaccagatatactaattttcttctggacactaagggaaaactctatcgctggcggtcacccgtcatcatagagaaggagggcaaggtggacgttgggggtcatttaatcgacttaaagagagttgtgcttgaaggttccgcggcaacccctgtaaccaagatttcagcggaacaatggggccgtccatag

>CF-CF24-20260129-10

atgttggggaaatgcttgaccgcgggctgctgctcgcaattgccttttttgtggtgtatcgtgccgttctgtttagttgcgctcgtcaacgcctacaacgacagcagctcccacttacaattgatttataacctaacgatatgtgagctgaatggcacagattggctgaaagaacacttttcttgggcggtggagactttcgttatttttcctgtgttgactcatatcgtctcctatggcgccctaaccaccagccacttccttgacacagtcggcctgatcaccgtgtctactgctggatattaccacgggcggtatgtcttgagcagcatttacgccgtctgcgccttagctgcgctaatttgcttcgtcatcaggctaacaaaaaattgcatgtcctggcgctactcttgcaccagatatactaattttcttctggacactaagggaaaactctatcgctggcggtcacccgtcatcatagagaaggagggcaaggtggacgttgggggtcatttaatcgacttaaagagagttgtgcttgaaggttccgcggcaacccctgtaaccaagatttcagcggaacaatggggccgtccatag

>CF-CF25-20250510-59

ATGTTGGGGAAATGCTTGACCGCGGGCTGCTGCTCGCAATTGCCTTTTTTGTGGTGTATCGTGCCGTTCTGTTTAGTTGCGCTCGTCAACGCCAACGACAACAGCAGCTCCCACTTACAATTGATTTATAACCTAACGATATGTGAGCTGAATGGCACAGATTGGCTGAAAGACCACTTTTCTTGGGCGGTGGAGACTTTCGTTATTTTTCCTGTGTTAACTCATATTGTCTCTTACGGTGCCCTCACCACTAGCCATTTTCTTGACACGGTCGGTCTTATCACTGTGTCCACCGCCGGGTATTATCACGGGCGGTATGTATTGAGTAGCATCTACGCTGTCTGCGCCCTAGCTGCGCTTGTTTGCTTCGCCATTAGGTTGGTAAAGAATTGCATGTCCTGGCGCTACTCTTGCACCAGATATACTAATTTTCTTCTGGACACTAAGGGAAAACTCTATCGTTGGCGGTCATCCGTCATCATAGAGAAGGGGGGCAAGGTGGATGTTGGTGGTCATTTAATCGACCTAAAGAGAGTTGTGCTTGAAGGTTCCGCGGCAACCCCTGTAACCAAGATTTCAGCGGAACAATGGGGTCGTCCATAG

>CF-25-20250814-87

ATGTTGGGGAGATGCTTGACCGCGGGCTGTTGCTCGCAATTGCCTTTTTTGTGGTATATCGTGCCGTTCTGCTT

TGTTGTGCTCGTCAACGCCAACAACAGCAGCAGCTCCCATTTACAGTTGATTTATAACCTAACGATATGTGAGC

TGAACGGCACAGATTGGCTAAATAAAAGTTTTGATTGGGCAGTGGAGACCTTTGTTTTTTTCCCTGTGTTGACT

CATATTGTTTCTTAGGGCGCCCTAACCACCAGCCATTTCCTTGACACAGTTGGCCTGATCACCGTGTCTACCGC

TGGATATTATCACGAGCGGTATGTCCTGAGCAGCATTTACGCCCCCTGCGCCCTAGCTGCGTTAACTTGCTTTG

TCATCAGGCTAACAAAAAATTGCATGTCCGGGCGTTACTCATGCACCAGATACACTAACTTTCTTCTGGACACC

AAGGGCAAACTCTATCGCTGGCGGTCTCCCGTCATCGTGGAGAAAGGGGGTAAAGTTGAGGTCGAAGGCCACCT

GATCGACCTCAAGAGAGTTGTGCTTGACGGTTCCGCGGCGACCCCTATAACCAAAGTTTCAGCGGAACAATGGG

GTCGTCCTTAG

>CF-25-20250913 ATGTTGGGGAAATGCTTGACCGCGGGTTGCTGCTCATCATTGCTTTTTTTGTGGTGTATCGTGCTATCTTGTTCTGTTGCGCTCGTCAACGCCGACAGCAACACCAGCTCTCATTTACAGTTGATTTATAACCTGACGATATGTGAGCTGAATGGCACGGATTGGTTGGACAAAAAATTTGACTGGGCAGTAGAGACTTTCGTCATTTTTCCTGTTCTAACCCATATTGTCTCTTACGGCGCCCTCACCACTAGCCATTTTCTTGACATGGTCGGCCTAATTACTGTGTCCACCGCTGGGTATTACCATGAGCGGTATGTATTGAGTAGCATCTATGCTGTCTGTGCCCTGGCCGCGTTGGCTTGCTTCGCCATCAGGTTGGCGAAAAATTGCATGTCCTGGCGCTACTCATGCACCAGATACACCAACTTTCTCCTGGACACTAAAGGCAGACTCTACCGCTGGCGGTCACCCGTCATCATAGAGAAGGGGGGCAAAGTTGATGTGGGGGGTCATTTAATTGACCTCAAGAGAGTCGTGCTTGATGGTTCCGCGGCGACCCCTATAACCAAGATTTCAGCGGAACAATGGGGTCGTCCATAG

>CF25-20251111-L22

TTGGCAATTTGAATGTTCAAGTATGTTGGGGAAATGCTTGACCGCGGGCTGCTGCTCGCAATTACTTTTTTTGTGGTGTATCGTGCCGTTCTGTTTAGTTGCGTTCGTCAACGCCAACAACAGCAGCAGCTCCCACTTACAGTTGATTTATAACCTAACGATATGTGAGCTGAATGGCACAGACTGGCTGAAAGAACATTTTTCTTGGGCAGTGGAGACTTTCGTTATTTTTCCTGTGTTAACCCACATTGTCTCTTACGGTGCCCTCACCACTAGCCATTTTCTTGACACGGTCGGCCTAATCACTGTGTCCACCGCCGGGTATTATCACGGGCGGTATGTACTGAGTAGCATCTATGCTGTCTGCGCCCTAGCTGCGCTTGTTTGCTTCGCCATTAGGTTGGTAAAAAATTGCATGTCCTGGCGCTACTCTTGCACCAGATATACTAATTTTCTTCTGGACACTAAGGGAAAACTCTATCGTTGGCGGTCACCCGTCATCATAGAGAAAGGGGGCAAGGTGGATGTTGGGGGTCATTTAATTGACCTAAAGAGAGTTGTGCTTGAAGGTTCCGCGGCGACTCCTGTAACCAAGATTTCAGCGGAACAATGGGGACATCCATAGACGACTTCTGCAATGATAGCACAG

>CF-CF25-20260120-10

ATGTTGGGGAAATGCTTGACCGCGGGTTGCTGCTCATCATTGCTTT-TTTGTGGTGTATCGTGCTATCTTGTTCTGTTGCGCTCGTCAACGCCGACAGCAACACCAGCTCTCATTTACAGTTGATTTATAACCTGACGATATGTGAGCTGAATGGCA-CGGATTGGTTGGACAAAAAATTTGACTGGGCAGTAGAGACTTTCGTCATTTTTCCTGTTCTAACCCATATTGTCTCTTACGGCGCCCTCACCACTAGCCATTTTCTTGACATGGTCGGCCTAATTACTGTGTCCACCGCTGGGTATTACCATAAGCGGTATGTATTGAGTAGCATCTATGCTGTCTGTGCCCTGGCCGCGTTGGCTTGCTTCGCCATCAGGTTGGCGAAAAATTGCATGTCCTGGCGCTACTCATGCACCAGATACACCAACTTTCTCCTGGACACTAAAGGCAGACTCTACCGCTGGCGGTCACCCGTCATCATAGAGAAGGGGGGCAAAGTTGATGTGGGGGGTCATTTAATTGACCTCAAAAGAGTCGTGCTTGATGGTTCCGCGGCGACCCCTATAACCAAGATTTCAGCGGAACAATGGGGTCGTCCATAG

>CF-CF25-20260120-12

ATGTTGGGGAGATGCTTGATCGCGGGCTGTTGCTCGCAATTGCCTTTTTTGTGGTATATCGTGCCGTTCTGCTTTGTTGTGCTCGTCAACGCCAACAACAGCAGCAGCTCCCATTTACAGTTGATTTATAACCTGACGATATGTGAGCTGAATGGCA-CAGATTGGCTAAATAAAAGTTTTGATTGGGCAGTGGAGACCTTTGTTATTTTCCCTGTGTTGACTCATATTGTCTCCTATGGCGCCCTAACCACCAGCCATTTCCTTGATACAGTTGGCCTGATCACCGTGTCTACCGCTGGATATTATCACGGGCGGTATGTCCTGAGCAGCATTTACGCCGCCTGCGCCCTAGCTGCGTTAACTTGCTTTGTCATCAGGCTAACAAAAAATTGCATGTCCTGGCGTTACTCATGCACCAGATACACTAACTTTCTTCTGGACACCAAGGGCAAACTCTATCGTTGGCGGTCTCCCGTCATCGTGGAGAAAGGGGGTAAAGTTGAGGTCGAAGGCCACCTGATCGACCTCAAGAGAGTTGTGCTTGACGGTTCCGCGGCGACCCCTATAACCAAAGTTTCAGCGGAACAATGGGGTCGTCCTTAG

>CF-CF25-20260120-1

atgttggggaaatgcttgaccgcgggctgctgctcgcaattgctttttttgtggtgtatcgtgccgttctgtttagttgcgctcgtcaacgccaacaacgacagcagctcccacttacaattgatttataacctaacgatatgtgagctgaatggcacagattggctgaacaaacactttccttgggcggtggagactttcgttatttttcctgtgttaacgcatgttgtctcttgtggtgccctcaccactagccattttcttgacacggtcggtcttatcactgtgtccaccgccgggtattatcacgggcggtatgtgttgagtagcatctacgctgtctgcgccctagctgcgcttgtttgcttcgccattaggttggtaaagaattgcatgtcctggcgctactcttgcaccagatatactaattttcttctggacacaaagggaaaactctatcgttggcggtcacccgtcatcatagagaaggggggcaaggtggatgttgggggtcatttaatcgacctaaagagagttgtgcttgaaggttccgcggcaacccctgtaaccaagatttcagcggaacaatggggttgtccatag

>CF-CF25-20260120-3

atgttggggaaatgcttgaccgcgggctgctgctcgcaattgctttttttgtggtgtatcgtgccgttctgtttagcttcgctcgtcaacgccagcaacagcagcagctcccatttacagttgatttataacctgacgatatgtgagctgaatggcacagattggctaaatcgtacttttgattgggcagtggagactttcgttatttttcctgtgttgactcacatcgtctcctatggcgccctgaccaccagccacttccttgacacagtcggcctgatcaccgtgtctactgctggatattgccacgggcggtatgtcttgagcagtatttacgccgtctgcgccttagctgcgctaatttgcttcgtcatcaggctaacaaaaaattgcatgtcctggcgttactcatgcaccagatacactaattttcttctggacaccaagggaaaactttatcgttggcggtctcccgtcatcgtggaaaaagggggtaaagttgaggtcgaaggccacctcatcgacctcaagaaagttgtgcttgatggttccgcggcgacccctataaccaaagtttcagcggaacaatggggtcgtccttag

>CF-CF26-20250528-111

ATGTTGGGGAAGTGCTTGACCGCGTGCTGTTGCTCGCGATTGCTTTTTTTGTGGTGTATCGTGCCGTCCTATCTTGTTGTGCTCGTCAACGCCAGCAACAACAACAGCTCTCATATTCAGTTGATTTATAACTTAACGCTATGTGAGCTGAATGGCACAGATTGGCTGGCACAAAAATTTGACTGGGCAGTGGAGACTTTCGTCATCTTCCCCGTGTTGACTCACATTGTTTCCTATGCGGCACTCACCACCAGCCATTTCCTTGACACAGTTGGTCTGGCCACTGTGTCCACCGCCGGATATTATCACGGGCGGTATGTCTTGAGTAGCATTTACGCAGTCTGTGCTCTGGCTGCGCTGATTTGCTTTGTCATTAGGCTTGCGAAGAACTGCATGTCCTGGCGCTACTCTTGTACCAGATATACCAACTTCCTTCTGGACACTAAGGGCAAACTCTATCGTTGGCGGTCGCCCGTCATTGTGGAGAAAGGGGGTAAGGTTGAGGTCGAAGGTCACCTGATCGACCTCAAGAGAGTTGTGCTTGATGGTTCCGCAGCAACCCCTTTAACCAGAGTTTCAGCGGAACGATGGGGTCGTCTCTAG

>CF-CF26-20250620

ATGTTGGAGAAATGCTTGACCGCGGGCTGTTGCTCGCAATTGCTTTCTTTGTGGTGTATCGTGCCGTTCTGTTTTGCTGTGCTCGCCAACGCCAGCAACGACAGCAGCTCCCATCTACAGCTGATTTACAACTTGACGCTATGTGAGCTGAATGGCACAGATTGGCTAGCTAACAAATTTGATTGGGCAGTGGAGAGTTTTGTCATCTTTCCCGTTTTGACTCACATTGTCTCCTATGGTGCCCTCACTACCAGCCATTTCCTTGACACAGTCGCTTTAGTCACTGTGTCTACCGCCGGGTTTGTTCACGGGCGGTATGTCCTAAGTAGCATCTACGCGGTCTGTGCCCTGGCTGCGTTGACTTGCTTCGTCATTAGGTTTGCAAAGAATTGCATGTCCTGGCGCTACGCGTGTACCAGATATACCAACTTTCTTCTGGACACTAAGGGCGGACTCTATCGTTGGCGGTCGCCTGTCATCATAGAGAAAAGGGGCAAAGTTGAGGTCGAAGGTCATCTGATCGACCTCAAAAGAGTTGTGCTTGATGGTTCCGTGGCAACCCCTATAACCAGAGTTTCAGCGGAACAATGGGGTCGTCCTTAG

>CF-26-20250727

ATGTTGGGGAAATGCTTGACCGCGGGCTGCTGCTCGCAATTGCCTTTTTTGTGGTGTATCGTGCCGTTCTGTTTAGTTGCGCTCGTCAACGCCAACGACAACAGCAGCTCCCACTTACAATTGATTTATAACCTAACGATATGTGAGCTGAATGGCACAGATTGGCTGAAAGACCACTTTTCTTGGGCGGTGGAGACTTTCGTTATTTTTCCTGTGTTAACTCATATTGTCTCTTACGGTGCCCTCACCACTAGCCATTTTCTTGACACGGTCGGTCTTATCACTGTGTCCACCGCCGGGTATTATCGCGGGCGGTATGTATTGAGTAGCATCTACGCTGTCTGCGCCCTAGCTGCGCTTGTTTGCTTCGCCATTAGGTTGGTAAAGAATTGCATGTCCTGGCGCTACTCTTGCACCAGATATACTAATTTTCTTCTGGACACTAAGGGAAAACTCTATCGTTGGCGGTCACCCGTCATCATAGAGAAGGGGGGCAAGGTGGATGTTGGTGGTCATTTAATCGACCTAAAGAGAGTTGTGCTTGAAGGTTCCGCGGCAACCCCTGTAACCAAGATTTCAGCGGAACAATGGGGTCGTCCATAG

>CF26-20251122-2

CCCGCTTTTTGCCATTCTGTTGGCAATTTGAATGTTTAGATATGTTGGGGAAATGCTTGACCGCGGGTTGCTGCTCATCATTGCTTTTTTTGTGGTGTATCGTGCTATCTTGTTCTGTTGCGCTCGTCAACGCCGACAGCAACACCAGCTCTCATTTACAGTTGATTTATAACCTGACGATATGTGAGCTGAATGGCACGGATTGGTTGGACAAAAAATTTGACTGGGCAGTAGAGACTTTCGTCATTTTTCCTGTTCTAACCCATATTGTCTCTTACGGCGCCCTCACCACTAGCCATTTTCTTGACATGGTCGGCCTAATTACTGTGTCCACCGCTGGGTATTACCATAAGCGGTATGTATTGAGTAGCATCTATGCTGTCTGTGCCCTGGCCGCGTTGGCTTGCTTCGCCATCAGGTTGGCGAAAAATTGCATGTCCTGGCGCTACTCATGCACCAGATACACCAACTTTCTCCTGGACACTAAAGGCAGACTCTACCGCTGGCGGTCACCCGTCATCATTGAGAAGGGGGGCAAAGTTGATGTGGGGGGTCATTTAATTGACCTCAAGAGAGTCGTGCTTGATGGTTCCGCGGCGACCCCTATAACCAAGATTTCAGCGGAACAATGGGGTCGTCCATAGACGACTTCTGCAATGACAGCACGGCTGTACAAAAGGTGCTTGGG

>CF-CF26-20260120-4

atgttggggaaatgcttgaccgcgggctgctgctcgcaattactttttttgtggtgtatcgtgccgttctgtttagttgcgttcgtcaacgccaacaacagcagcagctcccacttacagttgatttataacctaacgatatgtgagctgaatggcacagactggctgaaagaacatttttcttgggcagtggagactttcgttatttttcctgtgttaacccacattgtctcttacggtgccctcaccactagccattttcttgacacggtcggcctaatcactgtgtccaccgccgggtattatcacgggcggtgtgtactgagtagcatctatgctgtctgcgccctagctgcgcttgtttgcttcgccattaggttggtaaaaaattgcatgtcctggcgctactcttgcaccagacatactaattttcttctggacactaagggaaaactctatcgttggcggtcacccgtcatcatagagaaagggggcaaggtggatgttgggggtcatttaattgacctgaagagagttgtgcttgaaggttccgcggcgactcctgtaaccaagatttcagcggaacaatggggacatccatag

>CF-GC-20251227-26

CTGTCTTTTTGCCATCTGCTGGCAATTTAAATGTTCAGGTATGTTGGGGAAATGCTTGACCGCGGGCTGCTGCTCGCAATTGCTTTTTTTGTGGTGTATCGTGCCGTTCTGTTTAGTTTCGCTCGTCAACGCCAGCAACAACAGCAGCTCCCATTTACAGTTGATTTATAACCTGACGATATGTGAGCTGAATGGCACAGATTGGCTAAATAGCAGGTTTGATTGGGCAGTGGAGACCTTCGTCATTTTTCCTGTGTTGACTCATATCGTCTCCTATGGCGCCCTAACCACCAGCCACTTCCTTGACACAGTCGGCCTGATCACCGTGTCTACTGCTGGGTATTGCCACGGGCGGTATGTCTTGAGCAGTATTTACGCCGTCTGCGCCTTAGCTGCGCTAATTTGCTTCGTCATCAGGCTAACAAAAAATTGCATGTCCTGGCGTTACTCATGCACCAGATACACTAATTTTCTTCTGGACACCAAAGGAAAACTCTATCGTTGGCGGTCTCCCGTCATCGTAGAGAAAGGGGGCAAAGTTGAGGTCGAAGGCCACCTCATCGACCTTAAGAAAGTTGTGCTTGACGGTTCCGCGGCGACCCCTATAACCAAAGTTTCAGCGGAACAATGGGGTCGTCCTTAGACGACTTCTGCTATGACAGCACAGCCCCGCTAAAGGTGCTTTTGGCGTTTTCA

>CF-GC-20251227-27

CCCACTTTTTGCCATTCTGCTGGCAATTTAAATGTTCAGGTATGTTGGGGAAATGCTTGACCGCGGGCTGCTGCTCGCAATTGCTTTTTTTGTGGTGTATCGTGCCGTTCTGTTTAGTTTCGCTCGTCAACGCCAGCAACAACAGCAGCTCCCATTTACAGTTGATTTATAACCTGACGATATGTGAGCTGAATGGCACAGATTGGCTAAATAGCAGGTTTGATTGGGCAGTGGAGACCTTCGTCATTTTTCCTGTGTTGACTCATATCGTCTCCTATGGCGCCCTAACCACCAGCCACTTCCTTGACACAGTCGGCCTGATCACCGTGTCTACTGCTGGGTATTGCCACGGGCGGTATGTCTTGAGCAGTATTTACGCCGTCTGCGCCTTAGCTGCGCTAATTTGCTTCGTCATCAGGCTAACAAAAAATTGCATGTCCTGGCGTTACTCATGCACCAGATACACTAATTTTCTTCTGGACACCAAAGGAAAACTCTATCGTTGGCGGTCTCCCGTCATCGTAGAGAAAGGGGGCAAAGTTGAGGTCGAAGGCCACCTCATCGACCTTAAGAAAGTTGTGCTTGACGGTTCCGCGGCGACCCCTATAACCAAAGTTTCAGCGGAACAATGGGGTCGTCCTTAGACGACTTCTGCTATGACAGCACAGCCCCGCTAAAGTGTTTGAG

>CF-CFGC-20260120-3

atgttggggaaatgcttgaccgcgggctgctgctcgcaattgctttttttgtggtgtatcgtgccgttctgtttagtttcgctcgtcaacgccagcaacaacagcagctcccatttacagttgatttataacctgacgatatgtgagctgaatggcacagattggctaaatagcaggtttgattgg--agcggagaccttcgtcatttttcctgtgttgactcatatcgtctcctatggcgccctaaccaccagccacttccttgacacagtcggcctgatcaccgtgtctactgctgggtattgccacgggcggtatgtcttgagcagtatttacgccgtctgcgccttagctgcgctaatttgcttcgtcatcaggctaacaaaaaattgcatgtcctggcgttactcatgcaccagatacactaattttcttctggacaccaagg---aactctatcgttggcggtctcccgtcatcgt-agagaaaggggcaaagttgaggtcgaaggccacctcatcgaccttaagaaagttgtgcttgacggttccgcggcgacccctataaccaaagtttcagcggaacaatggggtcgtccttag

>CF-CFGC-20260120-7

atgttggggaaatgcttgaccgcgggctgctgctcgcaattgctttttttgtggtgtatcgtgccgttctgtttagtttcgctcgtcaacgccagcaacaacagcagctcccatttacagttgatttataacctgacgatatgtgagctgaatggcacagattggctaaatagcaggtttgattgggcagtggagaccttcgtcatttttcctgtgttgactcatatcgtctcctatggcgccctaaccaccagccacttccttgacacagtcggcctgatcaccgtgtctactgctgggtattgccacgggcggtatgtcttgagcagtatttacgccgtctgcgccttagctgcgctaatttgcttcgtcatcaggctaacaaaaaattgcatgtcctggcgttactcatgcaccagatacactaattttcttctggacaccaaaggaaaactctatcgttggcggtctcccgtcatc--agtagaagggggcaaagttgaggtcgaaggccacctcatcgaccttaag--agttgtgctcagcactttggcggtgacccctataaccaaagtttcagcggaacaacggggtcgtccttag

>CF-7-20251123-5

CCCGCTTTTTGCCATTCTGCTGGCAATTTGAATGTTCAGGTATGTTGGGGAAATGCTTGACCGCGGGCTGCTGCTCGCAATTGCCTTTTTTGTGGTGTATCGTGCCGTTCTGTTTGGTTGCGCTCGTCAACGCCAACGACAACAGCAGCTCCCACTTACAATTGATTTATAACCTAACGATATGTGAGCTGAATGGCACAGATTGGCTGAAAGAACACTTTTCTTGGGCGGTGGAGACTTTCGTTATTTTTCCTGTGTTAACTCATGTTGTCTCTTACGGTGCCCTCACCACTAGCCATTTTCTTGACACGGTCGGTCTTATCACTGTGTCCACCGCCGGGTATTATCACGGGCGGTATGTATTGAGTAGCATCTACGCTGTCTGCGCCCTAGCTGCGCTTGTTTGCTTCGCCATTAGGTTGGCAAAGAATTGCATGTCCTGGCGCTACTCTTGCACCAGATATACTAATTTTCTTCTGGACACTAAGGGAAAACTCTATCGTTGGCGGTCACCCGTCATCATAGAGAAAGGGGGCAAGGTGGATGTCGGGGGTCATTTAATCGACCTAAAGAGAGTTGTGCTTGAAGGTTCCGCGGCAACTCCTGTAACCAAGATTTCAGCGGAACAATGGGGTCGTCCATAGACGACTTCTGCAATGACAGCACGGCTGTACAAAAGGTGCTTTGGG

>CF-7-20251124-6

TGTTTTAGCCCTGTCTTTTTGCCATTCTGCTGGCAATTTGAATGTTCAGGTATGTTGGGGAAATGCTTGACCGCGGGCTGCTGCTCGCAATTGCCTTTTTTGTGGTGTATCGTGCCGTTCTGTTTGGTTGCGCTCGTCAACGCCAACGACAACAGCAGCTCCCACTTACAATTGATTTATAACCTAACGATATGTGAGCTGAATGGCACAGATTGGCTGAAAGAACACTTTTCTTGGGCGGTGGAGACTTTCGTTATTTTTCCTGTGTTAACTCATGTTGTCTCTTACGGTGCCCTCACCACTAGCCATTTTCTTGACACGGTCGGTCTTATCACTGTGTCCACCGCCGGGTATTATCACGGGCGGTATGTATTGAGTAGCATCTACGCTGTCTGCGCCCTAGCTGCGCTTGTTTGCTTCGCCATTAGGTTGGCAAAGAATTGCATGTCCTGGCGCTACTCTTGCACCAGATATACTAATTTTCTTCTGGACACTAAGGGAAAACTCTATCGTTGGCGGTCACCCGTCATCATAGAGAAAGGGGGCAAGGTGGATGTCGGGGGTCATTTAATCGACCTAAAGAGAGTTGTGCTTGAAGGTTCCGCGGCAACTCCTGTAACCAAGATTTCAGCGGAACAATGGGGTCGTCCATAGACGACTTCTGCAATGACAGCACGGCTGTACAAAAGGTGTTTG

>CF-8-20251128-30

TTGTTTTTAGCCCGTCTTTTTGCCATTCTGCTGGCAGTTTAAATGTTCAGGTATGTTGGGGAAATGCTTGACCGCGGGCTGCTGCTCGCAATTGCTTTTTTTGTGGTGTATCGTGCCGTTCTGTTTAGTTTCGCTCGTCAACGCCAGCAACAGCAGCAGCTCCCATTTACAGTTGATTTATAACCTGACGATATGTGAGCTGAATGGCACAGATTGGCTAAATCGCACTTTTGATTGGGCAGTGGAGACCTTCGTTATTTTTCCTGTGTTGACTCATATCGTCTCCTATGGCGCCCTAACCACCAGCCACTTCCTTGACACAGTCGGCCTGATCACCGTGTCTACTGCTGGATATTACCACGGGCGGTATGTCTTGAGCAGTATTTACGCCGTCTGCGCCTTAGCTGCGCTAATTTGCTTCGTCATCAGGCTAACAAAAAATTGCATGTCCTGGCGTTACTCATGCACCAGATACACTAATTTTCTTCTGGACACCAAGGGAAAACTCTATCGTTGGCGGTCTCCCGTCATCGTGGAAAAAGGGGGTAAAGTTGAGGTCGAAGGCCACCTCATCGACCTCAAGAAAGTTGTGCTTGATGGTTCCGCGGCGACCCCTATAACCAAAGTTACAGCGGAACAATGGGGTCGTCCTTAGACGACTTCTGCTATGACAGCACAGCCCCGCAAAAGGTGCTTTGGGCCGTTTTTC

>CF-8-20251128-31

TGTTTTAGCCCTGTCTTTTTGCCATTCTGCTGGCAGTTTAAATGTTCAGGTATGTTGGGGAAATGCTTGACCGCGGGCTGCTGCTCGCAATTGCTTTTTTTGTGGTGTATCGTGCCGTTCTGTTTAGTTTCGCTCGTCAACGCCAGCAACAGCAGCAGCTCCCATTTACAGTTGATTTATAACCTGACGATATGTGAGCTGAATGGCACAGATTGGCTAAATCGCACTTTTGATTGGGCAGTGGAGACCTTCGTTATTTTTCCTGTGTTGACTCATATCGTCTCCTATGGCGCCCTAACCACCAGCCACTTCCTTGACACAGTCGGCCTGATCACCGTGTCTACTGCTGGATATTACCACGGGCGGTATGTCTTGAGCAGTATTTACGCCGTCTGCGCCTTAGCTGCGCTAATTTGCTTCGTCATCAGGCTAACAAAAAATTGCATGTCCTGGCGTTACTCATGCACCAGATACACTAATTTTCTTCTGGACACCAAGGGAAAACTCTATCGTTGGCGGTCTCCCGTCATCGTGGAAAAAGGGGGTAAAGTTGAGGTCGAAGGCCACCTCATCGACCTCAAGAAAGTTGTGCTTGATGGTTCCGCGGCGACCCCTATAACCAAAGTTACAGCGGAACAATGGGGTCGTCCTTAGACGACTTCTGCTATGACAGCACAGCCCCGCAAAAGGTGCTTTTGGCGTTTTCA

>CF-8-20251219-21

TGTTTTAGCCTGTCTTTTTGCCATTCTGCTGGCGATTTGAATGTTCAGGTATGTTGGGGAAATGCTTGACCGCGGGCTGCTGCTCGCAATTGCCTTTTTTTTGGTGTATCGTGCCGTTCTGTTTAGTTGCGCTCGTCAACGCCAACGACAACAGCAGCTCCCACTTGCAATTGATTTATAACCTAACGATATGTGAGCTGAATGGCACAGATTGGCTGAAAGAACACTTTTCTTGGGCGGTGGAGACCTTCGTTATTTTTCCTGTGTTAACTCATATTGTCTCCTATGGCGCCCTAACCACCAGTCACTTCCTTGACACAGTCGGCCTGATCACCGTGTCTACTGCTGGATATTACCACGGGCGGTATGTCTTGAGCAGTATTTACGCCGTCTGCGCCTTAGCTGCGCTAATTTGCTTCGTCATCAGGCTAACAAAAAATTGCATGTCCTGGCGTTACTCATGCACCAGATACACTAATTTTCTCCTGGACACCAAGGGAAAACTCTATCGTTGGCGGTCACCCGTCATCATAGAGAAGGGGGGCAAGGTGGATGTTGGGGGTCATTTAATCGACCTAAAGAGAGTTGTGCTTGAAGGTTCCGCGGCAACCCCTGTAACCAAGATTTCAGCGGAACAATGGGGTCGTCCATAGACGACTTCTGCAATGACAGCACGGCTGTACAAAAGGTGCTTTGGGC

>CF-7-20260101-3

TGTTTTAGCCCTGTCTTTTTGCCATTCTGCTGGCAATTTGAATGTTCAGGTATGTTGGGGAAATGCTTGACCGCGGGCTGCTGCTCGCAATTGCTTTTTTTGTGGTGTATCGTGCCGTTCTGTTTAGTTGCGCTCGTCAACGCCAACGACAACAGCAGCTCCCACTTACAATTGATTTATAACCTAACGATATGTGAGCTGAATGGCACAGATTGGCTGAAAGAACACTTTTCTTGGGCGGTGGAGACTTTCGTTATTTTTCCTGTGTTAACTCATGTTGTCTCTTACGGTGCCCTCACCACTAGCCATTTTCTTGACACGGTCGGTCTTATCACTGTGTCCACCGCCGGGTATTATCACAGGCGGTATGTATTGAGTAGCATCTACGCTGTCTGCGCCCTAGCTGCGCTTGTTTGCTTCACCATTAGGTTGGTAAAGAATTGCATGTCTTGGCGCTACTCTTGCACCAGATATACTAATTTTCTTCTGGACACTAAGGGAAAACTCTATCGTTGGCGGTCACCCGTCATCATAGAGAAGGGGGGCAAGGTGGATGTTGGGGGTCATTTAATCGACCTAAAGAGAGTTGTGCTTGAAGGTTCCGCGGCAACCCCTGTAACCAAGATTTCAGCGGAACAATGGGGTCGTCCATAGACGACTTCTGCAATGACAGCACGGCTGTACAAAAGGTGCTTTTGGGCGTTTTCA

>CF-7-20260101-5

TGTTTTAGGCCCCGTCTTTTTGCCATTCTGCTGGCAATTTGAATGTTCAGGTATGTTGGGGAAATGCTTGACCGCGGGCTGCTGCTCGCAATTGCTTTTTTTGTGGTGTATCGTGCCGTTCTGTTTAGTTGCGCTCGTCAACGCCAACGACAACAGCAGCTCCCACTTACAATTGATTTATAACCTAACGATATGTGAGCTGAATGGCACAGATTGGCTGAAAGAACACTTTTCTTGGGCGGTGGAGACTTTCGTTATTTTTCCTGTGTTAACTCATGTTGTCTCTTATGGTGCCCTCACCACTAGCCATTTTCTTGACACGGTCGGTCTTATCACTGTGTCCACCGCCGGGTATTATCACGGGCGGTATGTATTGAGTAGCATCTACGCTGTCTGCGCCCTAGCTGCGCTTGTTTGCTTCACCATTAGGTTGGTAAAGAATTGCATGTCCTGGCGCTACTCTTGCACCAGATATACAAATTTTCTTCTGGACACTAAGGGAAAACTCTATCGTTGGCGGTCACCCGTCATCATAGAGAAGGGGGGCAAGGTGGATGTTGGGGGTCATTTAATCGACCTAAAGAGAGTTGTGCTTGAAGGTTCCGCGGCAACCCCTGTAACCAAGATTTCAGCGGAACAATGGGGTCGTCCATAGACGACTTCTGCAATGACAGCACGGCTGTACAAAAGGTGCTTGGGG

>Yigong-20260115-4

TGTTTTAGGCCTGTCTTTTTGCCATTCTGCTGGCAATTTGAATGTTCAGGTATGTTGGGG

AAATGCTTGACCGCGGGCTGCTGCTCGCAATTGCCTTTTTTGTGGTGTATCGTGCCGTT

CTGTTTGGTTGCGCTCGTCAACGCCAACGACAACAGCAGCTCCCACTTACAATTGATTT

ATAACCTAACGATATGTGAGCTGAATGGCACAGATTGGCTGGAAAAACACTTTTCTTGG

GCGGTGGAGACTTTCGTTATTTTTCCTGTGTTAACTCATGTTGTCTCTTACGGTGCCCTT

ACCACTAGCCATTTTCTTGACACGGTCGGTCTTATCACTGTGTCCACCGCCGGGTATTAT

CACGGGCGGTATGTATTGAGTAGCATCTACGCTGTCTGCGCCCTAGCTGCGCTTGTTTG

CTTCGCCATTAGGTTGGCAAAGAATTGCATGTCCTGGCGCTACTCTTGCACCAGATATA

CTAATTTTCTTCTGGACACTAAGGGAAAACTCTATCGTTGGCGGTCACCCGTCATCATA

GAGAAGGGGGGCAAGGTGGATGTTGGGGGTCATTTAATCGACCTAAAGAGAGTTGTGCTTGAAGGTTCCGCGGCAACCCCTGTAACCAAGATTTCAGCGGAACAATGGGGTCGTC

CATAGACGACTTCTGCAATGACAGCACGGCTGTACAAAAGGTGCTTTTGGGCCGTTTT

CA

>CF-9-20260122-2

TGTTTTAGCCCTGTCTTTTTGCCATCCTGCTGGCAATTTGAATGTTCAGGTATGTTGGGG

AAATGCTTGACCGCGGGCTGCTGCTCGCAATTGCCTTTTTTGTGGTGTATCGTGCCGTT

CTGTTTAGTTGCGCTCGTCAACGCCAACGAAAACAGCAGCTCCCACTTGCAATCGATTTATAACCTAACGATATGTGAGCTGAATGGCACAGATTGGCTGAAAGAACACTTTCCTTG

GGCGGTGGAGATTTTCGTTATTTTTCCTGTGTTAACTCATGTTGTCTCTTATGGTGCCCT

CACCACTAGCCATTTTCTTGACACGGTCGGTCTTATCACTGTGTCCACCGCCGGGTATTA

TCACGGGCGGTATGTATTGAGTAGCATCTACGCTGTTTGCGCCCTAGCTGCGCTTGTTT

GCTTCGCCATTAGGTTGGTAAAGAATTGCATGTCCTGGCGCTTCTCTTGCACCAGATAT

ACTAATTTTCTTCTGGACACTAAGGGGAAACTCTATCGTTGGCGGTCACCCGTCATCAT

AGAGAAGGGGGGCAAGGTGGATGTTGGGGGTCATTTAATCGACCTAAAGAGAGTTGT

GCTTGAAGGTTCCGCGGCAACCCCTGTAACCAAGATTTCAGCGGAACAATGGGGTCGT

CCATAGACGACTTCTGCAATGACAGCACGGCTGTACAAAAGGTGCTTTTGGCGTTTTC

A

>CF-8-20260123-4

TGTTTTAGCCTGTCTTTTTGCCATTATGCTGGCAATTTGAATGTTCAGGTATGTTGGGGA

AATGCTTGACCGCGGGCTGCTGCTCACAATTGCTTTTTTTGTGGTGTATCGTGCCGTTC

TGTTTAGTTGCGCTCGTCAACGCAAACGAAAACAGCAGCTCCCACTTACAATTGATTTA

TAACCTAACGATATGTGAGCTGAATGGCACAGATTGGCTGAAAGAACACTTTTCTTGG

GCGGTGGAGACTTTCGTTATTTTTCCTGTGTTAACTCATGTTGTCTCTTACGGTGCCCTC

ACCACTAGCCATTTTCTTGACACGGTCGGTCTTACCACTGTGTCCACCGCCGGGTATTA

TCACGGGCGGTATGTATTAAGTAGCATCTACGCTGTCTGCGCCCTAGCTGCGCTTGTTT

GCTTCGCCATTAGGTTGGTAAAGAATTGCATGTCCTGGCGCTACTCTTGCACCAGATAT

ACTAATTTTCTTCTGGACACTAAGGGAAAACTCTATCGTTGGCGGTCACCCGTCATCAT

AGAGAAGGGGGGCAAGGTGGATGTTGGGGGTCATTTAATCGACCTAAAGAGAGTTGT

GCTTGAAGGTTCCGCGGCAACCCCTGTAACCAAGATTTCAGCGGAACAATGGGGTCGT

CCATAGACGACTTCTGCAATGACAGCACGGCTATACAAAAGGTGCTTTTGGGCGTTTTC

A

>CF-7-20260128-1

atgttggggaaatgcttgaccgcgggctgctgctcgcaattgctttttttgtggtgtatcgtgccgttctgtttagttgcgctcgtcaacgccaacgacaacagcagctcccacttacaattgatttataacctaacgatatgtgagctgaatggcacagattggctgaaagaacacttttcttgggcggtggagactttcgttatttttcctgtgttaactcatgttgtctcttatggtgccctcaccactagccattttcttgacacggtcggtcttatcactgtgtccaccgccgggtattatcacgggcggtatgtattgagtagcatctacgctgtctgcgccctagctgcgcttgtttgcttcaccattaggttggtaaagaattgcatgtcctggcgctactcttgtaccagatatacaaattttcttctggacactaagggaaaactctatcgttggcggtcacccgtcatcatagagaaggggggcaaggtggatgttgggggtcatttaatcgacctaaagagagttgtgcttgaaggttccgcggcaacccctgtaaccaagatttcagcggaacaatggggtcgtccatag

>CF-7-20260128-2

atgttggggaaatgcttgaccgcgggctgctgctcgcaattgctttttttgtggtgtatcgtgccattctgtttagttgcgctcgtcaacgccaacgacaacagcagctcccacttacaattgatttataacctaacgatatgtgagctgaatggcacagattggctgaaagaacacttttcttgggcggtggagactttcgttatttttcctgtgttaactcatgttgtctcttacggtgccctcaccactagccattttcttgacacggtcggtcttatcactgtgtccaccgccgggtattatcacgggcggtatgtattgagtagcatctacgctgtctgcgccctagctgcgcttgtttgcttcaccattaggttggtaaagaattgcatgtcctggcgctactcttgcaccagatatactaattttcttctggacactaagggaaaactctatcgttggcggtcacccgtcatcatagagaaggggggcaaggtggatgttgggggtcatttaatcgacctaaagagagttgtgcttgaaggttccgcggcaacccctgtaaccaagatttcagcggaacaatggggtcgtccatag

>CF-7-20260128-3

atgttggagaaatgcttgaccgcgggctgttgctcgcgattgctttctttgtggtgtatcgtgccgttctgttttgctgtgctcgccaacgccagcaacgacagcagctcccatctacagctgatttacaacttgacgctatgtgagctgaatggcacagattggctagctaacaaatttgattgggcagtggagagttttgtcatctttcccgttttgactcacattgtctcctatggtgccctcactaccagccatttccttgacacagtcgctttagtcactgtgtctaccgccgggtttgttcacgggcggtatgtcctaagtagcatctacgcggtctgtgccctggctgcgttgatttgcttcgtcattaggtttgcaaagaattgcatgtcctggcgctacgcgtgtaccagatacaccaactttcttctggacactaagggcatactctatcgttggcggtcgcctgtcatcatagagaaaaggggcaaagttgaggtcgaaggtcatctgatcgacctcaaaagagttgtgcttgatggttccgtggcaacccctataaccagagtttcagcggaacaatggggtcgtccttag

>CF-7-20260128-4

atgttggggaaatgcttgaccgcgggctgctgctcgcaattgctttttttgtggtgtatcgtgccgttctgtttagttgcgctcgtcaacgccaacgacaacagcagctcccacttacaattgatttataacctaacgatatgtgagctgaatggcacagattggctgaaagaacacttttcttgggcggtggagactttcgttatttttcctgtgttaactcatgttgtctcttatggtgccctcaccactagccattttcttgacacggtcggtcttatcactgtgtccaccgccgggtattatcacgggcggtatgtattgagtagcatctacgctgtctgcgccctagctgcgcttgtttgcttcaccattaggttggtaaagaattgcatgtcctggcgctactcttgcaccagatatacaaattttcttctggacactaagggaaaactctatcgttggcggtcacccgtcatcatagagaaggggggcaaggtggatgttgggggtcatttaatcgacctaaagagagttgtgcttgaaggttccgcggcaacccctgtaaccaagatttcagcggaacaatggggtcgtccatag

>CF-7-20260128-5

atgttggagaaatgcttgaccgcgggctgttgctcgcgattgctttctttgtggtgtatcgtgccgttctgttttgctgtgctcgccaacgccagcaacaacagcagctcccatctacagctgatttacaacttgacgctatgtgagctgaatggcacagattggctagctaacaaatttgattgggcagtggagagttttgtcatctttcccgttttgactcacattgtctcctatggtgccctcactaccagccatttccttgacacagtcgctttagtcactgtgtctaccgccgggtttgttcacgggcggtatgtcctaagtagcatctacgcggtctgtgccctggctgcgttgatttgcttcgtcattaggtttgcaaagaattgcatgtcctggcgctacgcgtgtaccagatacaccaactttcttctggacactaagggcatactctatcgttggcggtcgcctgtcatcatagagaagaggggcaaagttgaggtcgaaggtcatctgatcgacctcaaaagagttgtgcttgatggttccgtggcgacccctataaccagagtttcagcggaacaatggggtcgtccttag

>CF-7-20260128-6

tatgttggggaatgcttgaccgcgggctgctgctcgcaattgctttttttgtggtgtatcgtgccgttctgtttagttgcgctcgtcaacgccaacgaaaacagcagctcccacttacaattgatttataacctaacgatatgtgagctgaatggcacagattggctgaaagaacacttttcttgggcggt-gagactttcgttatttttcctgtgttaactcatgttgtctcttatggtgccctcaccactagccattttcttgacacggtcg-tcttatcactgtgtccaccgccgggtattatcacgggcggtatgtattgagtagcatctacgctgtctgcgccctagctgcgcttgtttgcttcaccattaggttggtaaagaattgcatgtcctggcgctactcttgcaccagatatacaaattttcttctggacactaagggaaaactctatcgttggcggtcacccgtcatcatagagaaggggggcaaggtggatgttgggggtcatttaatcgacctaaagagagttgtgcttgaaggttccgcggc-acccctgtaaccaagatttcagcgg-acaatggggtcgtccatag

>CF-7-20260128-7

atgttggggaaatgcttgaccgcgggctgctgctcgcaattgctttttttgtggtgtatcgtgccattctgtttagttgcgctcgtcaacgccaacgacaacagcagctcccacttacaattgatttataacctaacgatatgtgagctgaatggcacagattggctgaaagaacacttttcttgggcggtggagactttcgttatttttcctgtgttaactcatgttgtctcttacggtgccctcaccactagccattttcttgacacggtcggtcttatcactgtgtccaccgccgggtattatcacgggcggtatgtattgagtagcatttacgctgtctgcgccctagctgcgcttgtctgcttcaccattaggttggtaaagaattgcatgtcctggcgctactcttgcaccagatatactaattttcttctggacactaagggaaaactctatcgttggcggtcacccgtcatcatagagaaggggggcaaggtggatgttgggggtcatttaatcgacctaaagagagttgtgcttgaaggttccgcggcaacccctgtaaccaagatttcagcggaacaatggggtcgtccatag

>CF-7-20260128-8

atgttggggaaatgc-tgaccgcgggctgctgctcgcaattgctttttttgtggtgtatcgtgccgttctgtttagttgcgctcgtcaacgccaacgacaacagcagct-ccacttacaattgatttataacctaacgatatgtgagctgaatggcacagattggctgaaagaacacttttcttgggcggtggagactttcgttatttttcctgtgttaactcatgttgtctc-tacggtgccctcaccactagccattttcttgacacggtcggtcttatcactgtgtcca-cgccgggtattatcacaggcggtatgtattgagtagcatctacgctgtctgcgccctagctgcgcttgtttgcttcaccattaggttggtaaagaattgcatgtcttgtcgctactcttgcaccagatatactaattttcttctggacactaagggaaaactctatcgttggcggtcacccgtcatcatagagaaggggggcaaggtggatgttgggggtcatttaatcgacctaaagagagttgtgcttgaaggttccgcggcaacccctgtaaccaagatttcagcggaacaatggggtcgtccatag

>CF-7-20260128-9

atgttggggaaatgcttgaccgcgggctgctgctcgcaattgctttttttgtggtgtatcgtgccgttctgtttagttgcgctcgtcaacgccaacgacaacagcagctcccacttacaattgatttataacctaacgatatgtgagctgaatggcacagattggctgaaagaacacttttcttgggcggtggagactttcgttatttttcctgtgttaactcatgttgtctcttacggtgccctcaccactagccattttcttgacacggtcggtcttatcactgtgtccaccgccgggtattatcacaggcggtatgtattgagtagcatctacgctgtctgcgccctagctgcgcttgtttgcttcaccattaggttggtaaagaattgcatgtcttggcgctactcttgcaccagatatactaattttcttctggacactaagggaaaactctatcgttggcggtcacccgtcatcatagagaaggggggcaaggtggatgttgggggtcatttaatcgacctaaagagagttgtgcttgaaggttccgcggcaacccctgtaaccaagatttcagcggaacaatggggtcgtccatag

>CF-7-20260128-10

atgttggggaaatgcttgaccgcgggctgctgctcgcaattgctttttttgtggtgtatcgtgccattctgtttagttgcgctcgtcaacgccaacgacaacagcagctcccacttacaattgatttataacctaacgatatgtgagctgaatggcacagattggctgaaagaacacttttcttgggcggtggagactttcgttatttttcctgtgttaactcatgttgtctcttacggtgccctcaccactagccattttcttgacacggtcggtcttatcactgtgtccaccgccgggtattatcacgggcggtatgtattgagtagcatctacgctgtctgcgccctagctgcgcttgtctgcttcaccattaggttggtaaagaattgcatgtcctggcgctactcttgcaccagatatactaattttcttctggacactaagggaaaactctatcgttggcggtcacccgtcatcatagagaaggggggcaaggtggatgttgggggtcatttaatcgacctaaagagagttgtgcttgaaggttccgcggcaacccctgtaaccaagatttcagcggaacaatggggtcgtccatag

>CF-7-20260128-11

atgttggggaaatgcttgaccgcgggctgctgctcgcaattgctttttttgtggtgtatcgtgccattctgtttagttgcgctcgtcaacgccaacgacaacagcagctcccacttacaattgatttataacctaacgatatgtgagctgaatggcacagattggctgaaagaacacttttcttgggcggtggagactttcgttatttttcctgtgttaactcatgttgtctcttacggtgccctcaccactagccattttcttgacacggtcggtcttatcactgtgtccaccgccgggtattatcacgggcggtatgtattgagtagcatctacgctgtctgcgccctagctgcgcttgtctgcttcaccattaggttggtaaagaattgcatgtcctggcgctactcttgcaccagatatactaattttcttctggacactaagggaaaactctatcgttggcggtcacccgtcatcatagagaaggggggcaaggtggatgttgggggtcatttaatcgacctaaagagagttgtgcttgaaggttccgcggcaacccctgtaaccaagatttcagcggaacaatggggtcgtccatag
